# Two-carbon ring expansion of bicyclic aziridines to oxazocines *via* aryne insertion into a σ C–N bond

**DOI:** 10.1039/d5sc04998a

**Published:** 2025-09-17

**Authors:** Daniel S. Rampon, Tuan Anh Trinh, Yekun Pan, Sierra Thein, Jacob W. Kailing, Ilia A. Guzei, Israel Fernández, Jennifer M. Schomaker

**Affiliations:** a Department of Chemistry, University of Wisconsin Madison Wisconsin 53706 USA schomakerj@chem.wisc.edu; b Departamento de Química Orgánica I and Centro de Innovación en Química Avanzada (ORFEO-CINQA), Facultad de Ciencias Químicas, Universidad Complutense de Madrid Madrid 28040 Spain israel@quim.ucm.es

## Abstract

Oxazocines are medium-sized N,O-heterocycles that are motifs in reported bioactive compounds; thus, methods for their rapid preparation and functionalization are of significant interest, particularly to increase their representation in current drug libraries. In this work, a mild method to access oxazocines through aryne insertion into the σ C–N bond of carbamate-tethered bicyclic aziridines is described. This work unlocks a complementary reactivity mode for bicyclic aziridines *via* a two-carbon ring expansion, which preserves both the strained ring and its stereochemical information for further modifications. Mechanistic studies of the reaction pathway using Density Functional Theory computations indicate that oxazocine formation *via* nucleophilic acyl substitution of the carbonyl group of the carbamate is kinetically preferred over alternative products arising from aziridine ring-opening pathways.

## Abstract 2

We have unlocked practical access to oxazocines through aryne insertion into the σ C–N bond of bicyclic aziridines. This two-carbon ring expansion preserves the aziridine ring, which was leveraged for oxazocine functionalizations.

## Graphical abstract

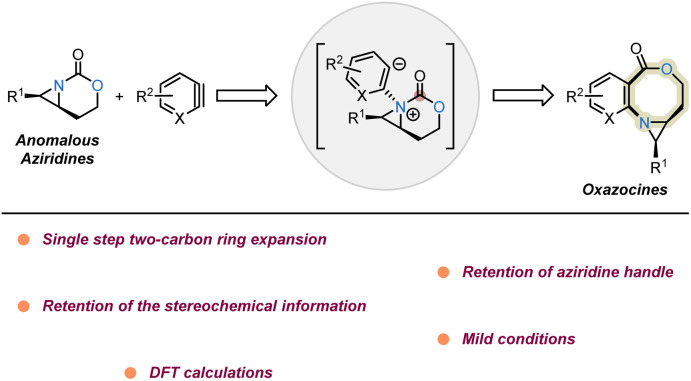

## Introduction

**Scheme 1:**
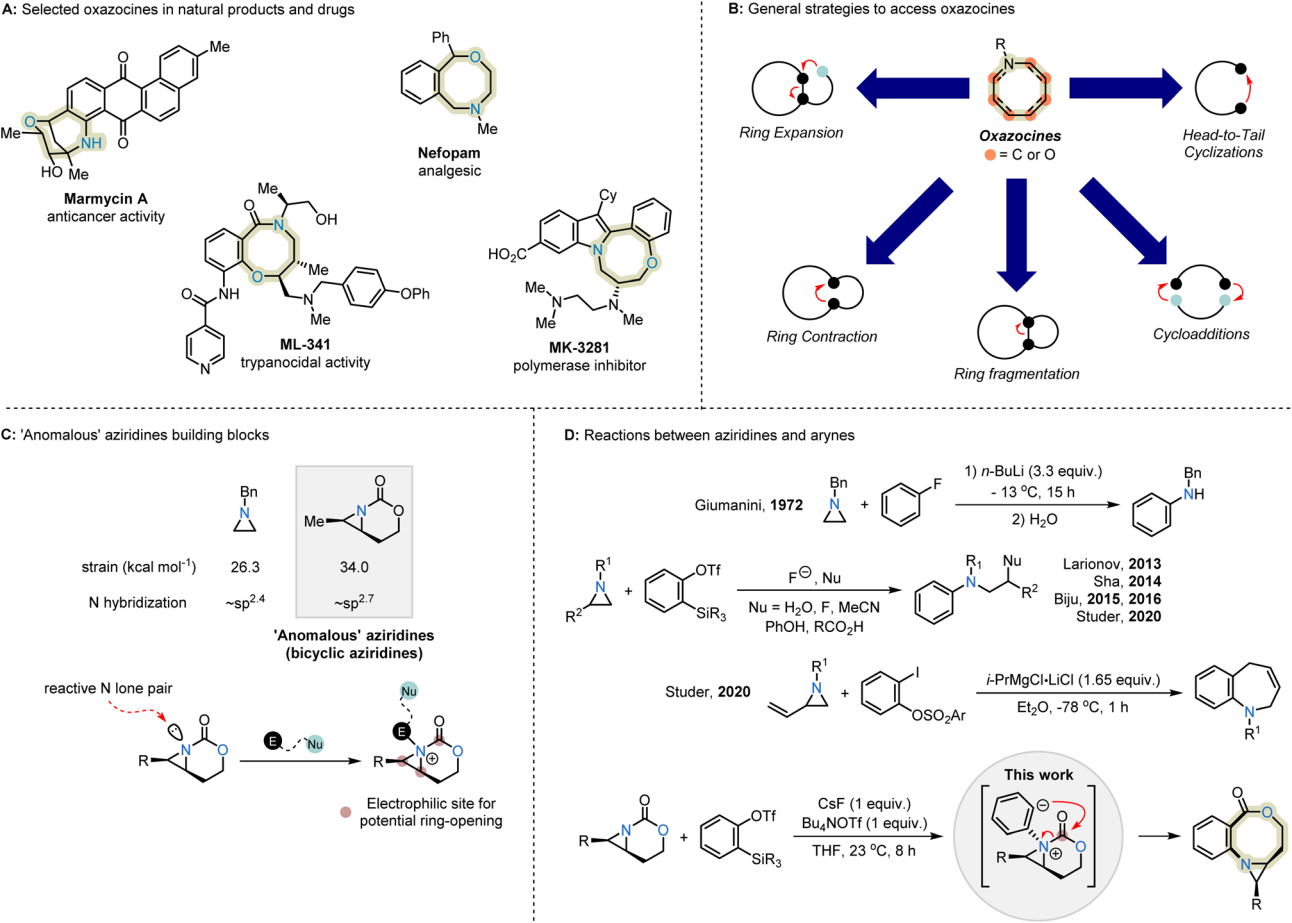
(A) Selected oxazocines in natural products and drugs. (B) General strategies to access oxazocines. (C) ‘Anomalous’ aziridine building blocks. (D) Reactions of aziridines and arynes.

N-Heterocycles are common motifs in pharmaceuticals, natural products, and fine chemicals, and continue to inspire the development of innovative strategies for their synthesis from versatile precursors. In fact, N-heterocycles are found as structural components in ∼82% of recent U.S. FDA-approved small-molecule drugs (January 2013–December 2023), with the majority of these comprised of 5- and 6-membered rings.^1^ In contrast, medium-sized rings (8–11-membered) are underrepresented in current drug screening libraries,^1,2^ despite the attractive interplay between their rigidity and broad conformational space that may lead to improved binding affinity to biological receptors, oral bioavailability, and cell permeability.^3^ In particular, natural and synthetic eight-membered N,O-heterocycles, including oxazocines, show valuable bioactivities that remain underexplored in compound screening collections, largely due to challenges inherent in their syntheses (Scheme 1A).^4^

Common strategies for the syntheses of substituted oxazocines include head-to-tail cyclizations, cycloadditions, ring fragmentations, ring contractions, and ring expansions (Scheme 1B).^5^ The kinetic barriers associated with head-to-tail cyclizations are typically high due to unfavorable entropy and enthalpy contributions to the transition state in the formation of medium-sized rings.^6^ These systems require careful optimization to prevent unwanted intermolecular reactions, such as dimerization and oligomerization. In addition, control over the relative and absolute stereochemistry has not been addressed in the preparation of oxazocines, leading to a need for new methods to prepare the heterocycles from readily available building blocks.

The construction of eight-membered cyclic frameworks *via* ring contraction or expansion strategies circumvents many of the common challenges associated with medium-sized ring formation.^7^ In recent years, our group has explored ring expansions of unusual aziridinium ylides, generated by the nucleophilic attack of bicyclic aziridines on metal-supported carbenes.^8,9^ The nitrogen of the carbamate-derived bicyclic aziridine precursor displays near-sp^3^ hybridization, which increases the nucleophilicity of the aziridine's nitrogen lone pair. Reaction with an electrophile bearing a latent nucleophile gives an intermediate with three electrophilic sites that may undergo strain-release ring-opening to enlarge the ring (Scheme 1C).^8^ Judicious choice of substrate, catalyst and reaction conditions enable us to successfully harness the reactivity of aziridinium ylides to furnish densely substituted, stereochemically complex N-heterocycles.

Arynes are highly reactive intermediates that function as polarized two-carbon synthons for the 1,2-difunctionalization of arenes.^10^ The low-lying LUMO resulting from the strained nature of the C≡C triple bond makes arynes susceptible to attack even by weak nucleophiles,^11^ such as non-activated aziridines. Reported examples of the addition of aziridines to arynes, generated from *o*-silylaryl triflates, typically yield products from fragmentation or ring-opening of the aziridinium ion by external nucleophiles (Scheme 1D).^12^ An exception was reported by the Studer group showing intramolecular ring expansion of vinyl aziridines in the absence of fluoride ion.^12*g*^ Given the unusual reactivity of carbamate-derived bicyclic aziridines, we proposed they might serve as building blocks to construct oxazocines *via* aziridinium intermediates generated from arynes. Attack of the aryne by the bicyclic aziridine generates a highly basic, nucleophilic aryl anion intermediate that undergoes rapid intramolecular nucleophilic acyl substitution instead of the expected competing aziridine ring-opening. The insertion of the aryne into a C–N σ-bond preserves the aziridine as a useful handle for further transformations (Scheme 1D). Herein, we report the successful demonstration of this formal cut-and-sew strategy to furnish oxazocines bearing a useful fused aziridine ring. No competing intermolecular nucleophilic opening of the aziridinium ion intermediate is observed, while full transfer of stereochemical information from the precursor to the product can be harnessed for subsequent stereoselective oxazocine functionalizations.

## Results and discussion

**Scheme 2:**
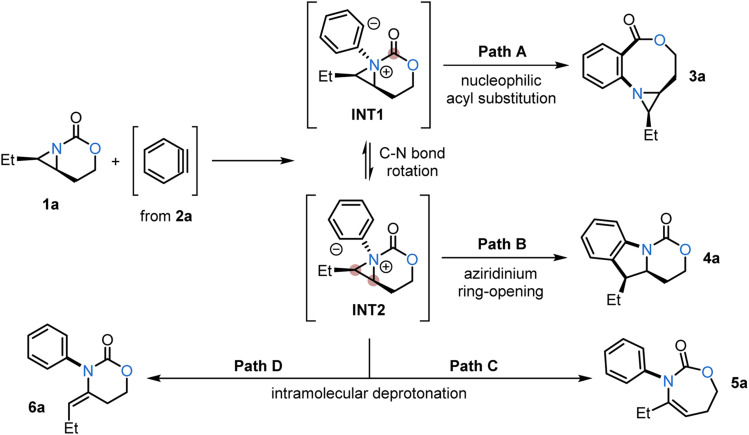
Two-carbon ring expansion of 1a with benzyne to furnish 3a (Path A) and an alternative pathway to 4a (Path B), 5a (Path C) and 6a (Path D).

Investigations were initiated by first assessing whether the aziridine 1a^9^ undergoes nucleophilic addition to a benzyne formed from 2-(trimethylsilyl)phenyl triflate (2a). The resulting aziridinium ion intermediate (INT1) could furnish oxazocine 3a (Scheme 2, Path A) by ring-opening of the carbamate. Alternatively, INT1 could undergo C–N bond rotation to furnish conformer INT2; opening of the aziridine by the aryl anion would give 4a (Scheme 2, Path B). Based on previous studies,^12*a*^ intramolecular deprotonations leading to 5a or 6a are also plausible outcomes for this reaction (Scheme 2, Paths C and D).

**Scheme 3:**
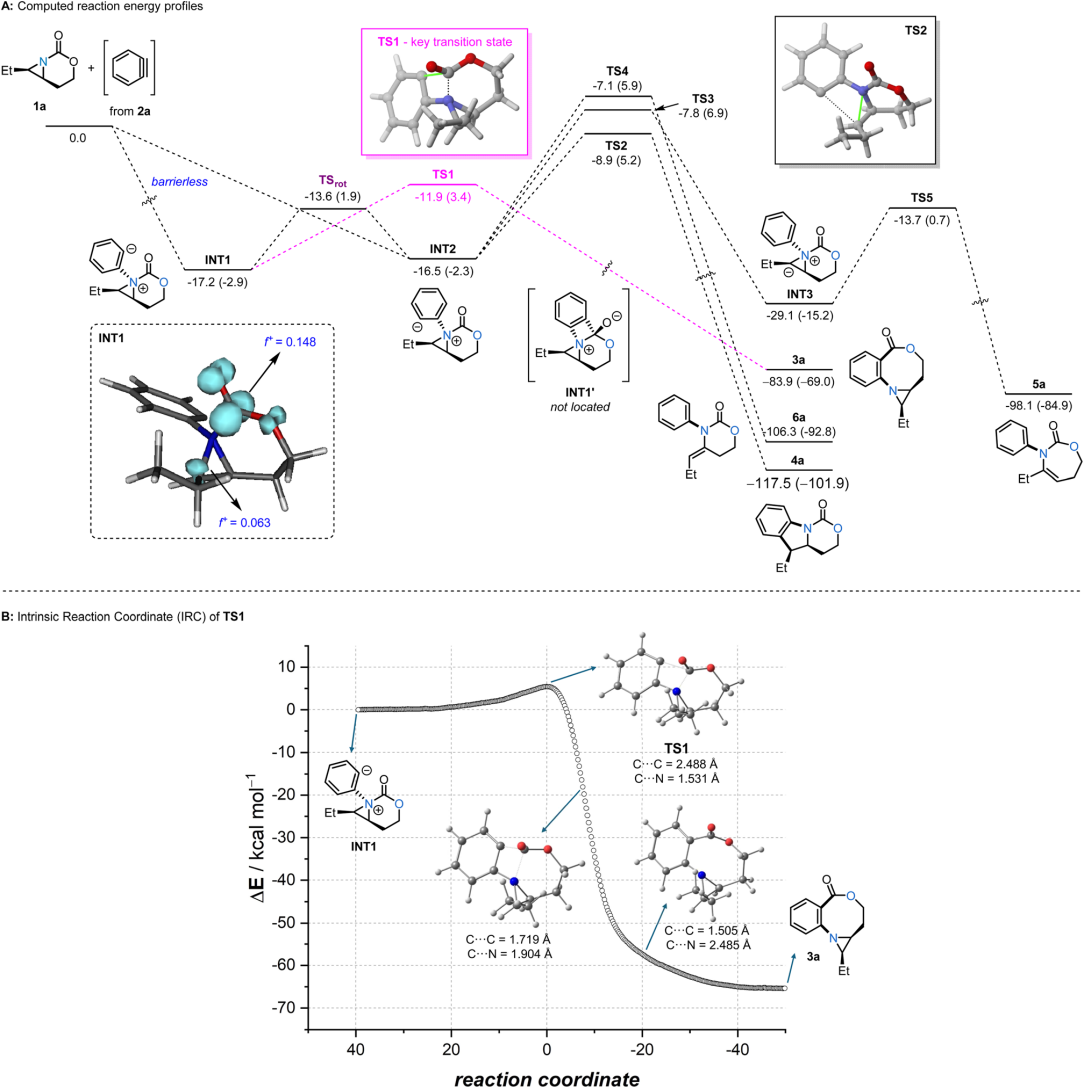
(A) Computed reaction profile for the reaction between benzyne and aziridine 1a. Relative enthalpies (Δ*H*) and free energies (Δ*G*) (within parentheses, at 298 K) are given in kcal mol^−1^. (inset): Computed condensed Fukui functions (*f*^+^) in INT1. (B) Intrinsic reaction coordinate computed for the transformation of INT1 into 3a. All data have been computed at the SMD(THF)-B3LYP-D3/def2-TZVPP//SMD(THF)-B3LYP-D3/def2-SVP level.

To this end, we first computationally explored the reaction between aziridine 1a and a benzyne using Density Functional Theory (DFT) calculations at the dispersion corrected SMD(THF)-B3LYP-D3/def2-TZVPP//SMD(THF)-B3LYP-D3/def2-SVP level (Scheme 3A). Similar to related nucleophilic additions to benzyne,^13^ the process begins with a barrierless, highly exothermic formation of INT1 or its isomer INT2 (Δ*H* ≈ −17 kcal mol^−1^ from the separate reactants).^14^ Both initial intermediates are nearly degenerate (ΔΔ*H* = 0.7 kcal mol^−1^), with INT1 being slightly more stable; conformers are easily interconverted through transition state TS_rot_ by simple rotation along the newly formed Csp^2^–N bond with a low activation barrier of 3.6 kcal mol^−1^ (ΔΔ*H*). Interestingly, INT1 can be directly transformed into oxazocine 3a through transition state TS1, a saddle point which is mainly associated with the formation of the new Csp^2^–C(=O) bond with a low barrier of 5.3 kcal mol^−1^ (ΔΔ*H*) in a highly exothermic (Δ*H*_R_ = −66.7 kcal mol^−1^) reaction. Instead of forming the four-membered ring intermediate INT1′, intrinsic reaction coordinate (IRC) calculations confirm that this intermediate is unstable on the potential energy surface and rapidly evolves to the oxazocine 3a (Scheme 3B), supporting that the formation of the new Csp^2^–C(=O) bond is also associated with the concomitant (O=)C–N bond rupture.

In contrast, INT2 can be transformed into tricyclic species 4a, which is thermodynamically more stable than 3a (ΔΔ*H* = −33.6 kcal mol^−1^). However, the formation of 4a (*via* transition state TS2) requires a higher barrier of 7.6 kcal mol^−1^ (ΔΔ*H*), indicating that the formation of the oxazocine is kinetically favored. Similarly, the alternative intramolecular deprotonation/aziridine ring-opening processes (involving TS3 and TS4) leading to species 5a and 6a, although thermodynamically favored over the formation of the oxazocine, also proceed with higher barriers (Δ*H*^≠^ = 8.7 and 9.4 kcal mol^−1^, respectively). Therefore, our calculations suggest that the formation of the oxazocine is kinetically preferred over other possible reaction products. This preference likely arises from the higher electrophilicity of the carbonyl carbon atom as compared to the carbon atom of the aziridine; this was confirmed by the corresponding condensed Fukui functions (*f*^+^) computed at the reactive intermediate INT1 (see inset in Scheme 3).

**Scheme 4:**
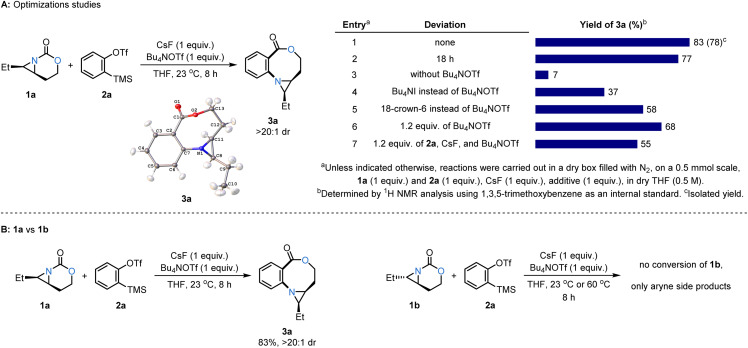
(A) Optimization studies. (B) Reaction of *cis*-1a*vs. trans*-1b.

The striking preference for formation of the oxazocine ring 3a was experimentally confirmed using 1a and the Kobayashi aryne precursor 2a, which afforded 3a as the only product in a high dr of >20 : 1 under several conditions (Scheme 4A). The structure of 3a was further supported by single-crystal X-ray diffraction (see Section 11 in the SI for details), where the *cis* stereochemistry of the aziridine ring was preserved during the course of the reaction. After extensive screening (see Table S1 in the SI for further details), the formation of the oxazocine 3a was found to proceed in optimal yield using CsF and Bu_4_NOTf as a fluoride-solubilizing agent^15^ in THF as the solvent at room temperature (Scheme 4A, entry 1). We found that diverse commercially available sources of fluoride promoted reactivity, while other aryne sources were less efficient replacements for *o*-silylaryl triflates (see Scheme S1 in the SI for details). The *o*-silylaryl triflates were ideal precursors, as only mild conditions are required to trigger *in situ* formation of the desired aryne. Representative optimization studies show a considerably lower yield of 3a in the absence of Bu_4_NOTf (entry 3); other common additives to enhance the solubility of fluoride salts proved inferior compared to standard conditions (entries 4, 5). Furthermore, the yield of 3a was not improved with extended reaction time (entry 2) and only moderate yields were observed using an excess of 2a, CsF, and Bu_4_NOTf (entries 6, 7). Conversely, even under forcing conditions, the *trans* isomer 1b showed no conversion to the desired oxazocine (Scheme 4B).^9*b*^ As noted earlier, the *cis* isomer 1a gives 3a in good yield, highlighting that ready access to the nitrogen lone pair is essential for efficient reaction with the aryne.

The scope of the two-carbon ring expansion of carbamate-tethered bicyclic aziridines with diverse aryne precursors was explored (Table 1, top). Reaction of the *cis*-aziridine 1a with 2a–p gave the oxazocines 3a–p, where the *cis* stereochemistry of the aziridine 1a was conserved in the product. No competing reactions of the *cis*-bicyclic aziridines were observed; the remaining mass balance consisted of remaining 1a and varying amounts of 2a–p. Bicyclic aziridine 1a was then reacted with a series of 3-substituted aryne precursors 2b–f; the resulting products 3b–f were obtained exclusively from the nucleophilic attack of the aziridine nitrogen at the less sterically hindered terminus on the aryne intermediate, which is also more distorted toward linearity.^16^ The high sensitivity of the reaction to steric effects is highlighted in reactions of 3,6-disubstituted *o*-silylaryl triflates 2g and 2h, which delivered low yields of the products 3g and 3h even under relatively harsh conditions. However, moderate-to-good yields were restored when the substituents on the Kobayashi aryne precursor were located distal to the approach trajectory between the aziridine nitrogen and the aryne, as observed for oxazocines 3i–m. The 4-substituted aryne precursor 2l, which bears an electron-withdrawing chlorine, provided greater regioselectivity in 3l resulting from nucleophilic attack of the aziridine at the *para*-position as compared to 3k (3l: 2.7 : 1 rr *vs.*3k: 1 : 1.3 rr).^17^ The reaction was also successful with ring-fused-arynes, producing 3n as the sole product in 54% yield. Benzo-fused five-membered heterocyclic aryne precursors were also compatible with this method, as exemplified by the use of 2o, a precursor of 4,5-benzofuranyne, which furnished a 70% yield of 3o in a regioselectivity consistent with the aryne distortion model.^18^ Given the importance of pyridines in medicinal chemistry, the compatibility of the reaction with a 2,3-pyridyne Kobayashi precursor 2p was assessed. Gratifyingly, the ring expansion smoothly produced oxazocine 3p in 44% yield after minor alterations to the standard reaction conditions;^19^ no trace of any other regioisomers were observed. Unfortunately, all attempts to translate this chemistry into the addition of bicyclic aziridines to strained alkynes and allenes proved unsuccessful (see Fig. S1 in the SI for further details). The higher LUMO energies of strained alkynes and allenes relative to arynes, together with distinct rates of intermediate formation under the optimized conditions,^20^ preclude productive use of other fleeting strained intermediates in this reaction.

**Table 1:** Scope of oxazocine formation with diverse *cis*-aziridines and aryne precursors

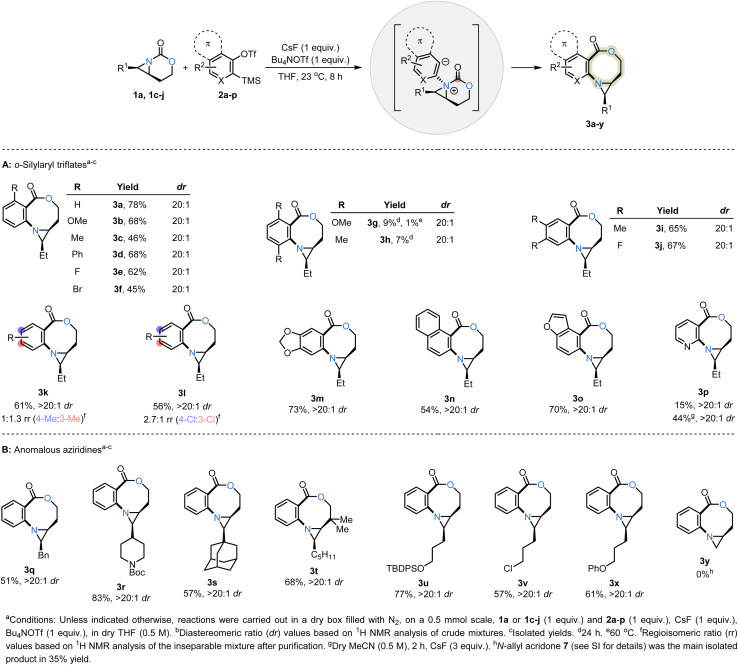

**Scheme 5:**
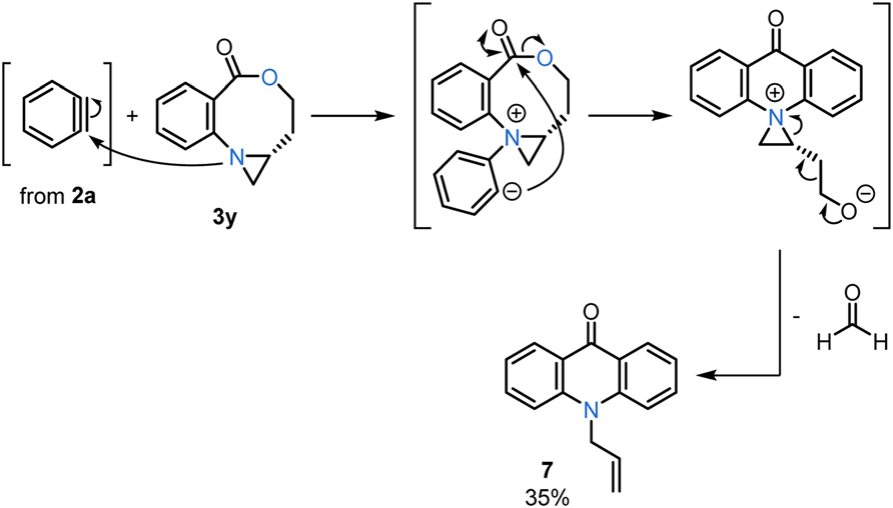
Proposed mechanism for *N*-allyl acridone (7) formation from 3y and 2a.

We next investigated the facility of the ring expansion with a series of *cis*-substituted bicyclic aziridines (1c–j) that display distinct side chains (Table 1, bottom). In general, the oxazocines 3q–y were obtained as the sole products in good-to-moderate yields and a high dr of >20 : 1. Secondary, tertiary, and quaternary carbon substituents were tolerated (3q–t) under these mild reaction conditions, and no side products were detected from alkyl migration, elimination, or fluoride-mediated aziridinium ring opening. A bicyclic aziridine 1d, containing an *N*-Boc protected amine, was also suitable, giving a good yield of 3r. Another interesting feature of this chemistry was its compatibility with a *tert*-butyldiphenylsilyl (TBDPS)-protected alcohol in 1g to furnish 3u in 77% yield, despite the well-known use of fluoride to deprotect silyl ethers. Additionally, aziridines substituted with primary alkyl chlorides (1h) or ethers (1i) were successful reaction partners with benzyne, providing 3v and 3x in 57% and 61% yields, respectively. Lastly, less-substituted bicyclic aziridines also undergo this ring expansion. However, the aziridine of 3y proved more nucleophilic than that of the 1j precursor, leading to full consumption of the oxazocine 3y through a second addition to benzyne. The major *N*-allyl acridone 7 product resulted from intramolecular nucleophilic acyl substitution, followed by formaldehyde extrusion (Table 1 and Scheme 5).^21^

**Scheme 6:**
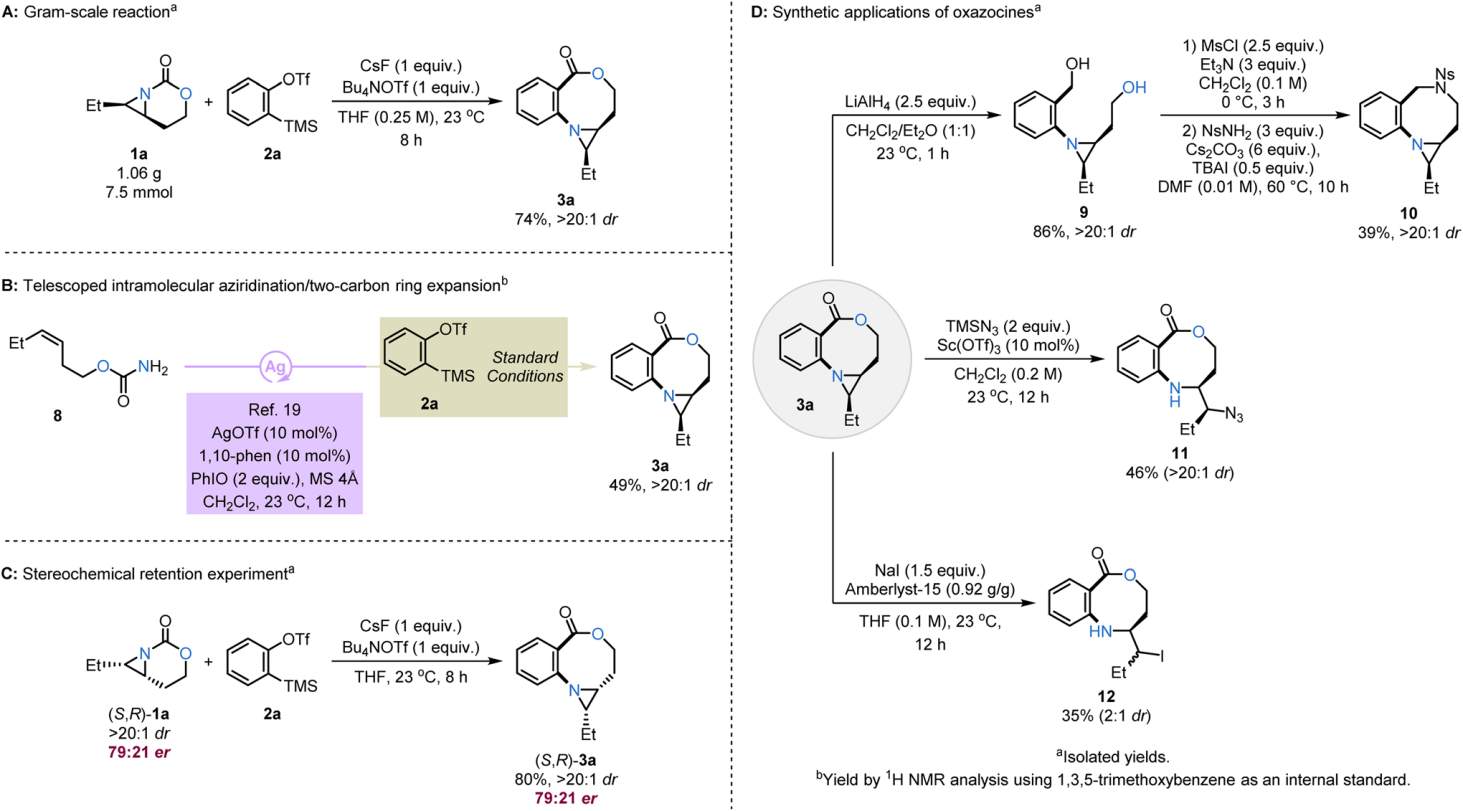
Synthetic utility of two-carbon ring expansion to oxazocines. (A) Gram-scale reaction. (B) Telescoped intramolecular aziridination/two-carbon ring expansion. (C) Stereochemical retention experiment. (D) Synthetic applications of oxazocines.

To highlight the usefulness of this transformation, a gram-scale reaction was conducted with 1a (1.06 g, 7.5 mmol) and 2a under slightly modified conditions, with no significant loss in efficiency (Scheme 6A). Another key feature of this transformation is the ability to telescope intramolecular Ag-catalyzed nitrene transfer (NT) of the homoallylic carbamates^22^ with the two-carbon ring expansion (Scheme 6B). After a short Celite pad and drying, the crude mixture from intramolecular aziridination of 8 was submitted to ring expansion, giving 49% of 3a after two steps. The synthetic utility of ring expansion using arynes was enhanced by employing enantioenriched anomalous aziridines (Scheme 6C). When the enantioenriched bicyclic aziridine (*S*,*R*)-1a (79 : 21 er) was treated with 2a under optimized conditions, complete retention of the stereochemical information at the aziridine stereocenters was observed in the resulting oxazocine 3a (79 : 21 er, (*S*,*R*)-3a), opening a valuable path for subsequent stereoselective oxazocine functionalizations.

The oxazocine ring fused to an aziridine moiety is synthetically quite versatile, enabling chemoselective functionalization of both the oxazocine and aziridine units under mild conditions (Scheme 6D). Reduction of the ester of 3a with LiAlH_4_ provided the corresponding diol 9 in 86% yield,^23^ delivering a pathway to convert the oxazocine ring into a diazocine 10 after dimesylation and dialkylation with NsNH_2_.^24^ We further demonstrated that the fused aziridine ring in 3a can be selectively opened using a combination of a Lewis or Brønsted–Lowry acid and a nucleophile. In the reaction, 3a was treated with Sc(OTf)_3_ and TMSN_3_ as the azide source to give 11 in 46% yield and >20 : 1 dr.^25^ Lastly, ring-opening with iodide and Amberlyst-15 furnished 12 in 35% yield and 2 : 1 dr.^26^ The reaction sequences in Scheme 6 demonstrate that under controlled conditions, the stereochemical information from the anomalous aziridines can be harnessed in the oxazocine fused aziridines for the synthesis of enantioenriched medium-sized N,O-heterocycles.

## Conclusions

In conclusion, we have reported practical access to oxazocines through aryne insertion into the σ C–N bond of our anomalous aziridines. This work unlocked a complementary reactivity mode for anomalous bicyclic aziridines through a two-carbon ring expansion that allows the retention of the aziridine ring and its stereochemical information. Subsequently, we have leveraged the synthetic potential of this fused aziridine structure for relevant oxazocine functionalizations. Finally, our DFT computations have uncovered key insights indicating that the formation of the observed oxazocines occurs under kinetic control in view of the lower activation barrier computed for the nucleophilic substitution on the carbamate moiety as compared to alternative pathways involving aziridine ring-opening reactions.

## Author contributions

The manuscript was written through contributions of all authors. All authors have given approval to the final version of the manuscript.

## Conflicts of interest

There are no conflicts to declare.

## Supplementary Material

SC-OLF-D5SC04998A-s001

## Data Availability

The data for 3a has been deposited with the CCDC number 2470550.^27^ The data supporting this article has been included as part of the SI. See DOI: https://doi.org/10.1039/d5sc04998a.
