# Supplementary material for: Two-carbon ring expansion of bicyclic aziridines to oxazocines via aryne insertion into a σ C–N bond
Source: Chem Sci. 2025 Sep 17;16(43):20368–75. doi: 10.1039/d5sc04998a (PMC12453046; doi:10.1039/d5sc04998a)
Supplement: SC-016-D5SC04998A-s001 [file SC-016-D5SC04998A-s001.pdf]

Supplementary Information (SI) for Chemical Science.
This journal is © The Royal Society of Chemistry 2025

Supporting information

Two-Carbon Ring Expansion of Bicyclic Aziridines to Oxazocines via Aryne Insertion into a σ C–N bond

Daniel S. Rampon,[a] Tuan Anh Trinh,[a] Yekun Pan,[a] Sierra Thein,[a] Jacob W. Kailing,[a] Ilia A. Guzei,[a] Israel Fernández,*[b] and Jennifer M. Schomaker*[a]

[a] Department of Chemistry, University of Wisconsin, Madison, Wisconsin 53706, United States. Email: schomakerj@chem.wisc.edu

[b] Departamento de Química Orgánica I and Centro de Innovación en Química Avanzada (ORFEO-CINQA), Facultad de Ciencias Químicas, Universidad Complutense de Madrid, Madrid 28040, Spain. Email: israel@quim.ucm.es

Table of Contents

1. General information S2
2. Reaction optimization S3
3. Additional aryne source screening S4
4. Other fleeting strained intermediates screening S4
5. Synthesis and characterization of starting materials S5
6. General procedures for two-carbon ring expansion S12
7. Gram-scale reaction procedure S24
8. Telescoped intramolecular aziridination/two-carbon ring expansion procedure S25
9. Stereochemical retention experiment procedure S26
10. Synthetic applications procedures and characterization S29
11. Crystal structure analysis of **3a** S32
12. Computational methods (Scheme 3) S38
13. References S55
14. NMR Spectra S57

1. General information

Unless otherwise specified, all reagents were purchased from commercial suppliers and used without further purification. Diethyl ether, tetrahydrofuran, dichloromethane, acetonitrile, and toluene were obtained from a PureSolv MD 5 solvent purification system. All other solvents were purified according to procedures from the sixth edition of "Purification of Laboratory Chemicals".[1] Before use, all glassware was either oven-dried overnight at 130 °C or flame-dried using a Bunsen burner, then cooled to room temperature in a desiccator containing Drierite™ or under a stream of dry nitrogen. The air- and moisture-sensitive reactions were performed using standard Schlenk techniques under an atmosphere of dried nitrogen. Analytical thin layer chromatography (TLC) was performed utilizing pre-coated silica gel 60 F_{254} plates containing a fluorescent indicator, while compounds without a UV signature were visualized using phosphomolybdic acid (PMA), potassium permanganate ($KMnO_4$), or ceric ammonium molybdate (CAM) staining solutions. Product purification was usually carried out by column chromatography using a Combiflash® RF (Teledyne ISCO) under a gradient method, equipped with RediSep® Silver silica gel disposable flash columns (silica gel pore size 60 Å, 230-400 mesh). In specific cases, the purification was done through regular column chromatography procedures using a gradient method in silica gel pore size 60 Å (230-400 mesh), beginning with 100% of the less polar eluent and gradually increasing the polarity with the other solvent. ^{1}H, ^{13}C{^{1}H}, and ^{19}F{^{1}H} spectra were recorded in $CDCl_3$ on a Bruker Avance-400 (400 MHz, 101 MHz, and 376 MHz) or Bruker Avance-500 (500 MHz and 126 MHz) NMR spectrometers. Chemical shifts are given in parts per million (ppm). For ^{1}H NMR, chemical shifts are reported relative to tetramethylsilane (TMS) (δ 0.00 in $CDCl_3$) or to residual protiated solvent peak (δ 7.26 for $CDCl_3$). ^{13}C{^{1}H} NMR chemical shifts are also reported relative to TMS (δ 0.00 in $CDCl_3$) or to residual protiated solvent peak (δ 77.16 for $CDCl_3$).[2] ^{19}F{^{1}H} NMR spectra were absolutely referenced to their respective solvent peaks in the ^{1}H NMR spectrum. The coupling constants (*J*) are reported in Hertz (Hz), and the multiplicities are described using the following abbreviations: singlet (s), doublet (d), doublet of doublets (dd), doublet of doublets of doublets (ddd), doublet of doublets of doublets of doublets (dddd), doublet of triplets (dt), doublet of doublet of triplets (ddt), doublet of triplet of doublets (dtd), triplet (t), triplet of doublets (td), triplet of doublet of doublets (tdd), doublet of quartets (dq), doublet of quartet of doublets (dqd), quartet (q), doublet of doublets of quartets (ddq), pentet (p), doublet of pentets (dp), pentet of doublets (pd) sextet (sext), sept (septet), broad singlet (bs) and multiplet (m). High-performance liquid chromatography (HPLC) analyses were performed using a Shimadzu LC-20AB instrument equipped with a CHIRALPAK® AD-H column (4.6 mm diameter x 25 mm length, particle size 5 µm), maintained at a temperature of 40 °C, using *i*-PrOH/hexanes mixtures as mobile phase. Accurate mass measurements were acquired at the University of Wisconsin-Madison using a Thermo Q Exactive™ Plus (electrospray ionization or atmospheric solids analysis probe (ASAP-MS) methods). The crystal evaluation and

data collection were performed on a Bruker D8 VENTURE Photon III four-circle diffractometer with Cu Kα (λ = 1.54178 Å) radiation and the detector to crystal distance of 50 mm and 70 mm. The spectrometers used for this work are from the Paul Bender Chemistry Instrumentation Center (NMR, mass spectrometry and X-ray crystallography) in the Department of Chemistry at UW-Madison. These facilities are funded by the NSF (CHE-1048642 and CHE-2017891), NIH (1S10 OD020022-1), the Bender Fund, UW2020, and the University of Wisconsin-Madison.

2. Reaction optimization

Table S1. Selected optimization screening results.[a,b]

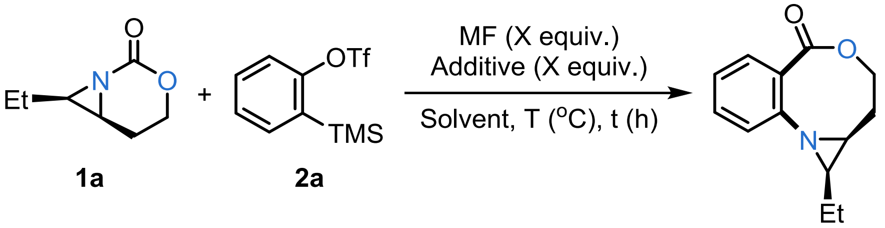

3a

Entry	MF (equiv.)	Additive (equiv.)	Solvent	T (°C)	t (h)	Yield of 3a (%)[c]	Recovered 1a (%)[c]
1	CsF (1)	Bu_4NOTf (1.5)	THF	23	8	64	39
2	CsF (1)	Bu_4NOTf (1)	THF	100	8	57[d]	-
3	CsF (1)	Bu_4NOTf (0.5)	THF	23	8	15	70
4	CsF (1)	Bu_4NOTf (1)	THF	23	4	51	30
5	KF (1)	18-crown-6 (1)	THF	23	8	59[d]	-
6	Bu_4NF in THF (1)	-	THF	23	8	43	43
7	TBAT (1)	-	THF	23	8	68[d]	-
8	CsF (1)	Bu_4NOTf (1)	MeCN	23	8	54	33
9	CsF (1)	Bu_4NOTf (1)	Toluene	23	8	77	22
10	CsF (1)	Bu_4NOTf (1)	1,2-DME	23	8	64	23
11	CsF (1)	Bu_4NOTf (1)	DMSO	23	8	7	43
12	CsF (1)	Bu_4NOTf (1)	DMF	23	8	0	83
13	CsF (1)	Bu_4NOTf (1)	DCM	23	8	45	37

[a]Unless indicated otherwise, reactions were carried out in a dry box filled with N_2, on a 0.5 mmol scale, **1a** (1 equiv.) and **2a** (1 equiv.), MF (equiv.), additive (equiv.), in dry THF (0.5 M). [b]The reactions were set up according to General Procedure A. [d]Determined by ^1H NMR analysis using 1,3,5-trimethoxybenzene as an internal standard. [d]Isolated yield.

3. Additional aryne source screening

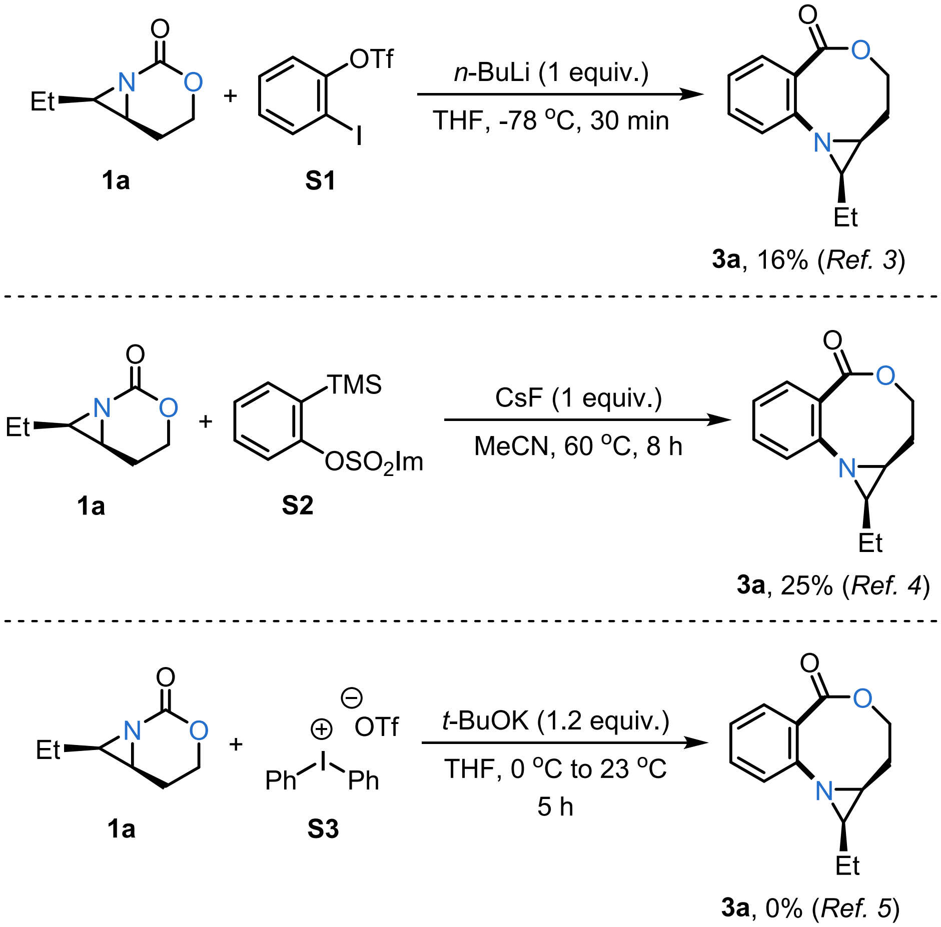

Scheme S1. Additional aryne source screening (^{1}H NMR yields using 1,3,5-trimethoxybenzene as an internal standard).

4. Other fleeting strained intermediates screening

Scheme S2 below shows the unsuccessful fleeting strained intermediates for the two-carbon ring expansion under optimized conditions.

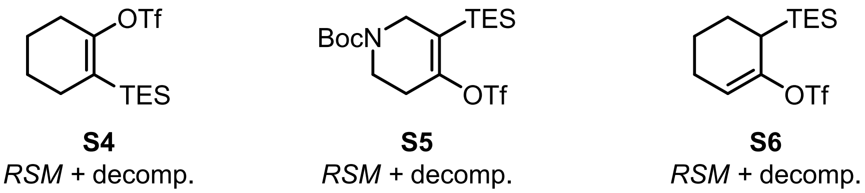

Figure S1. Unsuccessful fleeting strained intermediates.

5. Synthesis and characterization of starting materials

5.1 Synthesis of *o*-silylaryl triflates

Figure S2 shows the *o*-silyl triflates and other aryne precursors employed in this study. The *o*-silyl triflates **2a**, **2b**, **2i**, and **2j** were purchased from Ambeed and used without further purification. Substrate **S3** was purchased from Chem-Impex and used without further purification. The remaining aryne precursors **2c**,[6] **2d**, **2e**,[6] **2f**,[6] **2g**,[7] **2h**,[8] **2k**,[6] **2l**,[6] **2m**,[6] **2n**,[6] **2o**,[9] **2p**,[10] **S1**,[11] and **S2**,[4] were synthesized according to reported literature procedures. The strained alkyne and allene precursors **S4**,[12] **S5**,[13] and **S6**[12] were also prepared according to published literature procedures.

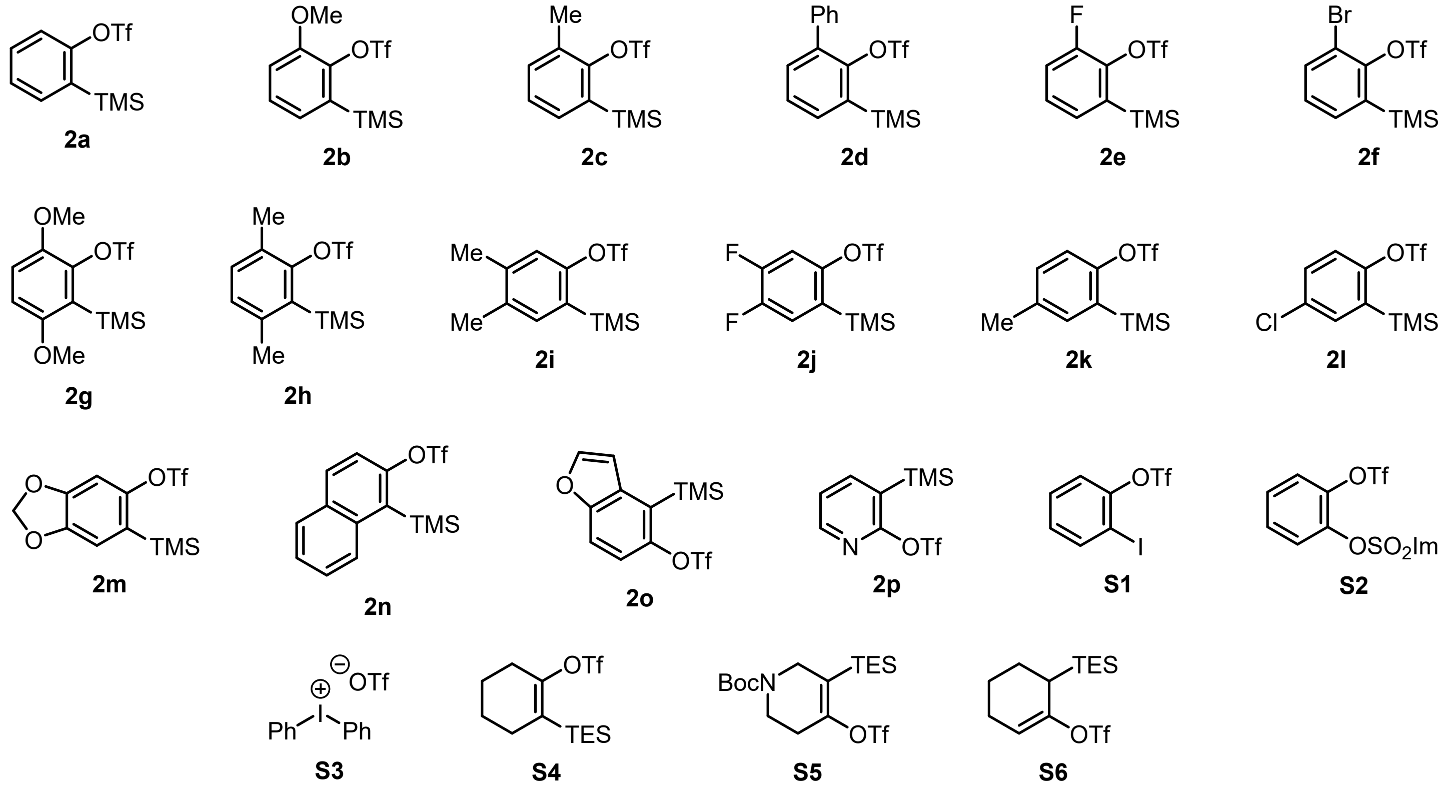

Figure S2. *O*-silyl triflates and other aryne precursors employed in this study.

5.2 Synthesis of bicyclic aziridines

The bicyclic aziridines (**1a–j**) were prepared according to literature procedures from the respective homoallylic carbamates (**8** and **S8**) (Scheme S2).[14] The homoallylic carbamates (**8** and **S8**) were also prepared from reported

procedures using commercially available homoallylic alcohols (**S7**) or prepared from the synthetic route below (Scheme S2).[14,15]

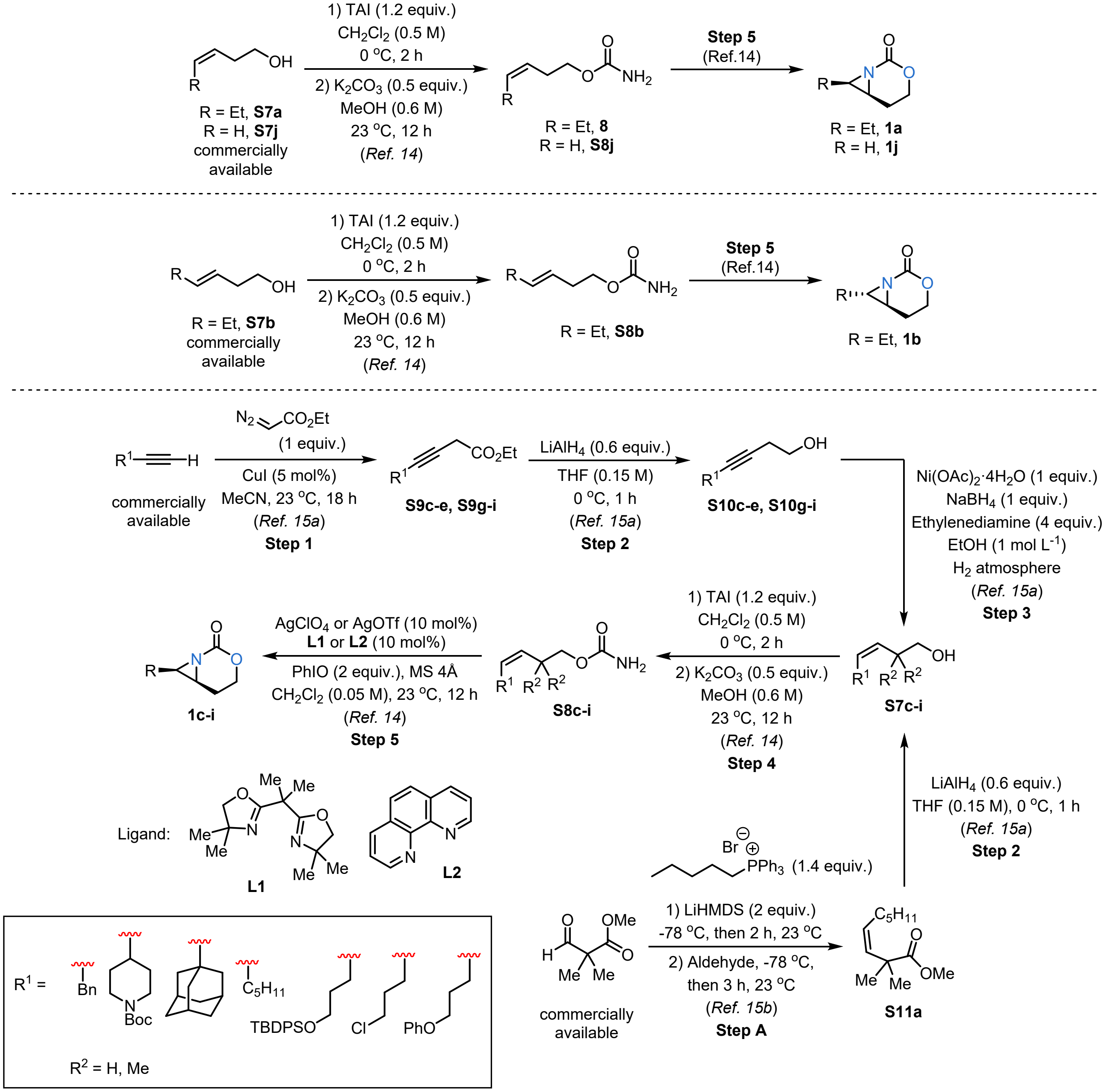

Scheme S2. Synthesis of the bicyclic aziridines (**1a–j**).

Step A:[15b] In a flame-dried two-neck round bottom flask under N_2 atmosphere, THF (0.1 M) was added, followed by (1-pentyl)triphenylphosphonium bromide (1.4 equiv.). The solution was cooled to -78 °C and then LHMDS (2 equiv., 1 M in THF) was added. After stirring for 2 h at room temperature, methyl 2,2-dimethyl-3-oxopropanoate (1 equiv.) was added to the mixture at –78 °C, which was further stirred at room temperature for

3 h. The reaction was quenched by the addition of saturated aqueous NH_4Cl solution, and the mixture was extracted with three portions of Et_2O. The extract was washed with brine (3x) and water (2x), dried, and concentrated. The crude product was then purified by column chromatography using a gradient method with mixtures of hexanes/EtOAc to afford the product **S11a** as a colorless liquid.

Step 1:[15a] Terminal alkyne (1 equiv.), dry MeCN (1 M), and CuI (5 mol%) were added sequentially to a dried Schlenk flask under N_2 atmosphere. Ethyl diazoacetate (87% wt in DCM, 1 equiv.) was then added dropwise, and the reaction mixture was stirred for 18 h at room temperature. The solvent was removed under reduced pressure, and the residue was dissolved in Et_2O and filtered through Celite. The filtrated was concentrated and purified by column chromatography using a gradient method with mixtures of hexanes/EtOAc to afford **S9**.

Step 2:[15a] In a flame-dried two-neck round bottom flask under N_2 atmosphere, dry THF (0.15 M) was added, followed by the ester **S9** (1 equiv.). The reaction mixture was cooled to 0 °C, and $LiAlH_4$ (0.6 equiv.) was added portion-wise over 30 min. The reaction was stirred for more 30 min at 0 °C and quenched using Fieser's work-up procedure: a mixture containing x g of $LiAlH_4$ at 0 °C was treated with slow addition of x mL of H_2O, 2x mL of a 10% aqueous NaOH solution, and 2x mL of H_2O, then diluted with Et_2O and dried over Na_2SO_4 with vigorous stirring for 15 min at room temperature. The resulting mixture was filtered through a pad of Na_2SO_4. The filtrate was concentrated under reduced pressure to afford the homopropargyl alcohol **S10**, which was used in the next step without purification.

Step 3:[15a] To a homogeneous solution of $Ni(OAc)_2{\cdot}4H_2O$ (1 equiv.) in EtOH (1 M) under H_2 atmosphere, $NaBH_4$ (1 equiv.) in EtOH (1 M) was slowly added at room temperature. After stirring for 30 min, an ethanolic solution (1 M) of the homopropargyl alcohol **S10** (1 equiv.) and ethylenediamine (4 equiv.) was added, and the reaction mixture was vigorously stirred for 3.5 h at room temperature (*NOTE: Check the consumption of the starting material by TLC every 30 minutes to prevent overreduction*). Once the reaction was complete, EtOH was removed under reduced pressure. The residue was diluted with CH_2Cl_2 (0.4 M) and filtered through a pad of Celite (2x). The crude product was then purified by column chromatography using a gradient method with mixtures of hexanes/EtOAc to afford the *Z*-homoallylic alcohol **S7**.

Step 4:[14] In a flame-dried two-neck round bottom flask under N_2 atmosphere, the homoallylic alcohol **S7** (1 equiv.) was dissolved in dry CH_2Cl_2 (0.5 M) and cooled to 0 ˚C. Trichloroacetylisocyanate (TAI) (1.2 equiv.) was then added dropwise to the cooled solution. The reaction was stirred at 0 ˚C until TLC indicated complete consumption of the starting material (usually between 1 h to 2 h). The solvent was then removed under reduced

pressure and the crude reaction mixture was dissolved in MeOH (0.6 M). K_2CO_3 (0.5 equiv.) was added in a single portion, and the mixture was stirred at room temperature for 12 h. Saturated aqueous NH_4Cl was added to quench the reaction, and the mixture was extracted with six portions of CH_2Cl_2. The combined organic phases were washed with brine. The crude product solution was then adjusted to 0.05 M in DCM, and 1 M aqueous NaOH solution (10 mL/mmol) was added. The biphasic solution was vigorously stirred for 30 min to remove the trichloroacetamide impurity (*NOTE: It is imperative that this step is done carefully. If not, it is likely that the aziridination will not proceed to completion).* The mixture was extracted with three portions of CH_2Cl_2. The combined organic phases were washed with brine again, dried over Na_2SO_4 or $MgSO_4$ and concentrated under reduced pressure. The crude product was purified by column chromatography using a gradient method with mixtures of hexanes/EtOAc to afford the *Z*-homoallylic carbamate **8** or **S8**.

Step 5:[14] A cooled, flame-dried round bottom flask was charged with $AgClO_4$ or AgOTf (10 mol%) and dimethyl bisoxazoline (dmBox) **L1** or 1,10-phenanthroline ligand **L2** (10 mol%). Dry CH_2Cl_2 (0.05 M) was added to the flask and the mixture was stirred vigorously for 20 min (*NOTE: Both **L1** and **L2** will furnish the aziridine product. **L2** may result in varying ratios of aziridine to C–H insertion product if the metal/ligand ratio is not carefully measured to be 1:1. **L1** is the preferable ligand, as the ratio of metal/ligand is not as important to the aziridine/CH insertion product ratio. However, **L2** is a good commercially available alternative if **L1** is unavailable*). After 20 min of pre-stirring, powdered 4Å molecular sieves (1g of sieves /mmol of substrate) were added (*NOTE: It is not necessary to use rigorously dried molecular sieves*). After 5 min, the homoallylic carbamate **8** or **S8** (1 equiv.) and recently prepared iodosobenzene (2 equiv.) were added. The reaction was then capped, covered in aluminum foil and allowed to stir at room temperature for 2-12 h. After TLC indicated complete consumption of the starting material, the reaction mixture was filtered over Celite and concentrated. The crude mixture was then purified by column chromatography – once with a gradient elution of 0–2.5% MeOH/CH_2Cl_2, followed by a second column with a gradient elution of 0–50% EtOAc in hexanes (*NOTE*: *Omitting the first plug with CH_2Cl_2 leaves silver byproducts in the mixture, which, upon concentration, can degrade the bicyclic aziridine over time*). The compounds (**1a–j**) were stored at -20 ˚C to prevent decomposition.

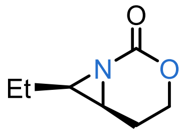

1a

Compound 1a:[14c] Following the aziridination reaction (step 5) on 5 mmol scale. The product was purified by column chromatography using a Combiflash® RF (Teledyne ISCO), equipped with a 24 g silica gel disposable flash column (0 – 2.5% MeOH/CH_2Cl_2, then 0% – 50% EtOAc in hexanes, 5% increments) to yield **1a** as a

colorless oil (550.6 mg, 3.9 mmol, 78%, >20:1 *dr*). R_f = 0.17 (40% EtOAc/hexanes). ^{1}H NMR (500 MHz, $CDCl_3$) δ 4.43 – 4.31 (m, 2H), 2.87 (ddd, J = 9.0, 7.0, 4.9 Hz, 1H), 2.60 (dt, J = 8.3, 5.1 Hz, 1H), 2.19 (ddt, J = 14.7, 7.0, 2.0 Hz, 1H), J = 14.7, 7.0, 2.0 Hz, 1H), 1.86 (dqd, J = 14.6, 7.4, 5.4 Hz, 1H), 1.48 (dddd, J = 14.6, 12.2, 9.0, 4.7 Hz, 1H), 1.30 – 1.18 (m, 1H), 1.11 (t, J = 7.4 Hz, 3H). ^{13}C NMR (126 MHz, $CDCl_3$) δ 158.7, 67.9, 44.1, 37.3, 19.1, 10.9. HRMS-ESI (*m/z*) calculated for $C_7H_{11}NO_2$ $[M+H]^+$ 142.0863; found, 142.0862.

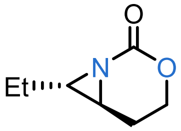

1b

Compound 1b:[16] Following the aziridination reaction (step 5), on a 9.3 mmol scale. The product was purified by column chromatography using a Combiflash® RF (Teledyne ISCO), equipped with a 40 g silica gel disposable flash column (0 – 2.5% MeOH/CH_2Cl_2, then 0% – 50% EtOAc in hexanes, 5% increments) to yield **1b** as a clear oil (1039.0 mg, 7.4 mmol, 79%, >20:1 *dr*). R_f = 0.43 (40% EtOAc/hexanes). ^{1}H NMR (500 MHz, $CDCl_3$) δ 4.40 (ddd, J = 12.4, 10.6, 2.0 Hz, 1H), 4.29 (ddd, J = 10.6, 4.1, 1.9 Hz, 1H), 2.60 (ddd, J = 8.7, 6.2, 3.2 Hz, 1H), 2.35 (ddt, J = 14.5, 6.2, 1.9 Hz, 1H), 2.23 (td, J = 6.0, 3.3 Hz, 1H), 1.67 – 1.59 (m, 2H), 1.39 (dddd, J = 14.6, 12.5, 8.7, 4.1 Hz, 1H), 1.06 (t, J = 7.5 Hz, 3H). ^{13}C NMR (126 MHz, $CDCl_3$) δ 161.1, 68.2, 50.1, 39.9, 25.2, 25.1, 10.6. HRMS-ESI (*m/z*) calculated for $C_7H_{11}NO_2$ $[M+H]^+$ 142.0863; found, 142.0864.

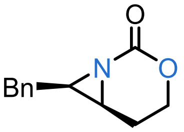

1c

Compound 1c:[14c] Following the aziridination reaction (step 5), on a 4.9 mmol scale. The product was purified by column chromatography using a Combiflash® RF (Teledyne ISCO), equipped with a 24 g silica gel disposable flash column (0 – 2.5% MeOH/CH_2Cl_2, then 0% – 60% EtOAc in hexanes, 5% increments) to yield **1c** as a white solid (691.0 mg, 3.4 mmol, 69%, >20:1 *dr*). R_f = 0.24 (30% EtOAc/hexanes). ^{1}H NMR (500 MHz, $CDCl_3$) δ 7.34 (t, J = 8.3, 2H), 7.30 – 7.21 (m, 3H), 4.50 – 4.38 (m, 2H), 3.33 (dd, J = 15.4, 3.9 Hz, 1H), 2.98 (ddd, J = 9.0, 6.9, 4.8 Hz, 1H), 2.90 (ddd, J = 10.0, 4.8, 3.9 Hz, 1H), 2.41 (dd, J = 15.3, 10.0 Hz, 1H), 2.28 (ddt, J = 14.6, 6.8, 1.9 Hz, 1H), 1.68 (dddd, J = 14.3, 12.1, 8.9, 4.7 Hz, 1H). ^{13}C NMR (126 MHz, $CDCl_3$) δ 158.6, 136.7, 129.0, 128.6, 127.2, 68.2, 42.8, 37.9, 31.2, 19.7. HRMS-ESI (*m/z*) calculated for $C_{12}H_{13}NO_2$ $[M+H]^+$ 204.1019; found, 204.1019.

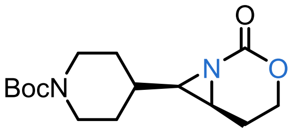

1d

Compound 1d: Following the aziridination reaction (step 5), on a 1.1 mmol scale. The product was purified by column chromatography using a Combiflash® RF (Teledyne ISCO), equipped with a 12 g silica gel disposable flash column (0 – 5% MeOH/CH_2Cl_2, then 0% – 80% EtOAc in hexanes, 5% increments) to yield **1d** as a white solid (127.4 mg, 0.43 mmol, 39%, >20:1 *dr*). R_f = 0.29 (30% EtOAc/hexanes). (The product **1d** was obtained as a mixture of rotamers. These data represent empirically observed chemical shifts from the ^{1}H and ^{13}C NMR spectra). ^{1}H NMR (500 MHz, $CDCl_3$) δ 4.44 – 4.32 (m, 2H), 4.12 (br, 2H), 2.84 (ddd, J = 9.1, 6.7, 4.9 Hz, 1H), 2.69 (m, 1H), 2.41 (dd, J = 9.8, 5.0 Hz, 2H), 2.23 (ddt, J = 14.5, 6.7, 1.9 Hz, 1H), 1.61 – 1.32 (m, 14H), 1.31 – 1.20 (m, 2H). ^{13}C NMR (126 MHz, $CDCl_3$) δ 158.5, 154.8, 79.7, 67.8, 46.7, 36.6, 34.4, 29.30, 29.0, 28.5, 19.8. HRMS-ESI (*m/z*) calculated for $C_{15}H_{24}N_2O_4$ $[M+H]^+$ 297.1809; found, 297.1806.

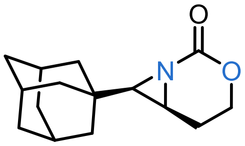

1e

Compound 1e: Following the aziridination reaction (step 5), on a 0.96 mmol scale. The product was purified by column chromatography using a Combiflash® RF (Teledyne ISCO), equipped with a 12 g silica gel disposable flash column (0 – 2.5% MeOH/CH_2Cl_2, then 0% – 50% EtOAc in hexanes, 5% increments) to yield **1e** as a white solid (98.9 mg, 0.40 mmol, 42%, >20:1 *dr*). R_f = 0.38 (40% EtOAc/hexanes). ^{1}H NMR (500 MHz, $CDCl_3$) δ 4.40 – 4.28 (m, 2H), 2.71 (ddd, J = 9.2, 7.4, 5.3 Hz, 1H), 2.27 (d, J = 5.2 Hz, 1H), 2.24 – 2.13 (m, 2H), 2.04 – 1.90 (m, 6H), 1.80 – 1.63 (m, 9H). ^{13}C NMR (126 MHz, $CDCl_3$) δ 158.6, 67.8, 53.2, 40.7, 37.8, 36.7, 35.7, 28.3, 20.7. HRMS-ESI (*m/z*) calculated for $C_{15}H_{21}NO_2$ $[M+H]^+$ 248.1645; found, 248.1645.

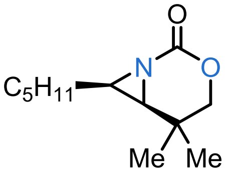

1f

Compound 1f: Following the aziridination reaction (step 5), on a 1.29 mmol scale. The product was purified by column chromatography using a Combiflash® RF (Teledyne ISCO), equipped with a 24 g silica gel disposable flash column (0 – 2.5% MeOH/CH_2Cl_2, then 0% – 60% EtOAc in hexanes, 5% increments) to yield **1f** as a pale-yellow oil (253.5 mg, 1.20 mmol, 93%, >20:1 *dr*). R_f = 0.20 (30% EtOAc/hexanes). ^{1}H NMR (500 MHz, $CDCl_3$) δ 4.15 (d, J = 10.4 Hz, 1H), 3.77 (d, J = 10.4 Hz, 1H), 2.60 (br q, J = 6.9, 5.2 Hz, 1H), 2.46 (d, J = 5.2 Hz, 1H), 1.83 (dddd, J = 14.0, 10.5, 6.9, 4.8 Hz, 1H), 1.77 – 1.66 (m, 1H), 1.66 – 1.57 (m, 1H), 1.52 – 1.40 (m, 1H), 1.40 – 1.29 (m, 4H), 1.27 (s, 3H), 1.06 (s, 3H), 0.95 – 0.86 (m, 3H). ^{13}C NMR (126 MHz, $CDCl_3$) δ 158.8, 77.6, 46.5,

46.4, 31.7, 30.8, 27.7, 27.2, 26.3, 22.5, 21.1, 14.0. HRMS-ESI (*m/z*) calculated for $C_{12}H_{21}NO_2$ $[M+H]^+$ 212.1645; found, 212.1643.

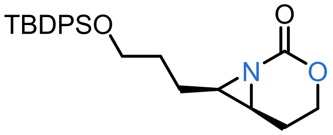

1g

Compound 1g: Following the aziridination reaction (step 5), on a 1.43 mmol scale. The product was purified by column chromatography using a Combiflash® RF (Teledyne ISCO), equipped with a 24 g silica gel disposable flash column (0 – 5% MeOH/CH_2Cl_2, then 0% – 60% EtOAc in hexanes, 5% increments) to yield **1g** as a white solid (364.5 mg, 0.89 mmol, 62%, >20:1 *dr*). R_f = 0.49 (50% EtOAc/hexanes). ^{1}H NMR (500 MHz, $CDCl_3$) δ 7.64 (d, *J* = 7.9 Hz, 4H), 7.46 – 7.35 (m, 6H), 4.41 – 4.27 (m, 2H), 3.76 – 3.63 (m, 2H), 2.83 (ddd, *J* = 8.9, 6.9, 4.9 Hz, 1H), 2.63 (dt, *J* = 8.7, 5.0 Hz, 1H), 2.10 (ddt, *J* = 14.6, 7.0, 1.9 Hz, 1H), 1.94 – 1.77 (m, 2H), 1.69 – 1.59 (m, 1H), 1.49 (dddd, *J* = 14.6, 12.2, 8.9, 4.6 Hz, 1H), 1.36 (dtd, *J* = 14.0, 8.6, 5.9 Hz, 1H), 1.04 (s, 9H). ^{13}C NMR (126 MHz, $CDCl_3$) δ 158.6, 135.7, 135.7, 133.9, 133.8, 129.9, 129.8, 127.9, 127.8, 67.9, 63.1, 42.6, 37.5, 29.6, 27.0, 22.2, 19.3, 19.2. HRMS-ESI (*m/z*) calculated for $C_{24}H_{31}NO_3Si$ $[M+H]^+$ 410.2146; found, 410.2140.

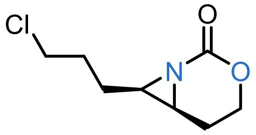

1h

Compound 1h:[14c] Following the aziridination reaction (step 5), on a 1.35 mmol scale. The product was purified by column chromatography using a Combiflash® RF (Teledyne ISCO), equipped with a 24 g silica gel disposable flash column (0 – 5% MeOH/CH_2Cl_2, then 0% – 60% EtOAc in hexanes, 5% increments) to yield **1h** as a white solid (138.4 mg, 0.73 mmol, 54%, >20:1 *dr*). R_f = 0.25 (50% EtOAc/hexanes). ^{1}H NMR (500 MHz, $CDCl_3$) δ 4.45 – 4.32 (m, 2H), 3.63 (ddd, *J* = 11.0, 6.8, 5.2 Hz, 1H), 3.56 (ddd, *J* = 11.0, 7.6, 5.2 Hz, 1H), 2.89 (ddd, *J* = 9.0, 6.9, 4.9 Hz, 1H), 2.89 (ddd, *J* = 9.0, 6.9, 4.9 Hz, 1H), 2.26 – 2.12 (m, 2H), 1.98 – 1.88 (m, 1H), 1.83 (dddd, *J* = 14.5, 9.5, 5.8, 4.8 Hz, 1H), 1.63 – 1.48 (m, 2H). ^{13}C NMR (126 MHz, $CDCl_3$) δ 158.6, 68.0, 44.4, 42.0, 37.4, 29,6, 23.2, 19.4. HRMS-ESI (*m/z*) calculated for $C_8H_{12}ClNO_2$ $[M+H]^+$ 190.0629; found, 190.0627.

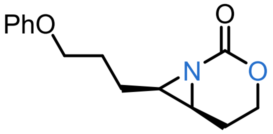

1i

Compound 1i: Following the aziridination reaction (step 5), on a 2.11 mmol scale. The product was purified by column chromatography using a Combiflash® RF (Teledyne ISCO), equipped with a 24 g silica gel disposable

flash column (0 – 5% MeOH/CH_2Cl_2, then 0% – 60% EtOAc in hexanes, 5% increments) to yield **1i** as a pale-yellow oil (328.7 mg, 1.33 mmol, 63%, >20:1 *dr*). R_f = 0.18 (50% EtOAc/hexanes). ^{1}H NMR (500 MHz, $CDCl_3$) δ 7.28 (d, *J* = 7.5 Hz, 2H), 6.94 (t, *J* = 7.3 Hz, 1H), 6.87 (d, *J* = 7.5 Hz, 2H), 4.43 – 4.28 (m, 2H), 4.04 (ddd, *J* = 9.1, 6.7, 4.6 Hz, 1H), 3.97 (ddd, *J* = 9.2, 6.7, 4.9 Hz, 1H), 2.89 (ddd, *J* = 8.9, 6.8, 4.8 Hz, 1H), 2.71 (dt, *J* = 8.3, 4.9 Hz, 1H), 2.19 – 2.07 (m, 2H), 2.02 – 1.88 (m, 2H), 1.58 – 1.46 (m, 2H). ^{13}C NMR (126 MHz, $CDCl_3$) δ 158.8, 158.7, 129.6, 121.0, 114.5, 68.0, 66.8, 42.5, 37.6, 26.5, 22.5, 19.3. HRMS-ESI (*m/z*) calculated for $C_{14}H_{17}NO_3$ $[M+H]^+$ 248.1281; found, 248.1278.

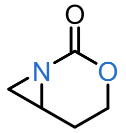

1j

Compound 1j:[14d] Following the aziridination reaction (step 5), on a 6.93 mmol scale. The product was purified by column chromatography using a Combiflash® RF (Teledyne ISCO), equipped with a 40 g silica gel disposable flash column (0 – 2.5% MeOH/CH_2Cl_2, then 0% – 70% EtOAc in hexanes, 5% increments) to yield **1j** as a pale-yellow solid (235.2 mg, 2.08 mmol, 30%). R_f = 0.77 (40% EtOAc/hexanes). ^{1}H NMR (500 MHz, $CDCl_3$) δ 4.45 (ddd, *J* = 12.4, 10.6, 2.0 Hz, 1H), 4.33 (ddd, *J* = 10.6, 4.1, 1.9 Hz, 1H), 2.81 (ddt, *J* = 8.4, 6.0, 4.0 Hz, 1H), 2.54 (d, *J* = 4.4 Hz, 1H), 2.43 (ddt, *J* = 14.7, 6.1, 2.0 Hz, 1H), 2.07 (d, *J* = 3.7 Hz, 1H), 1.36 (dddd, *J* = 14.7, 12.6, 8.7, 4.1 Hz, 1H). ^{13}C NMR (126 MHz, $CDCl_3$) δ 161.0, 68.2, 35.3, 34.0, 25.5. HRMS-ESI (*m/z*) calculated for $C_5H_7NO_2$ $[M+Na]^+$ 136.0369; found, 136.0369.

6. General procedures for two-carbon ring expansion

6.2. General procedure A

In a nitrogen-filled dry box, a flame-dried 4-dram vial containing a magnetic stir bar was charged sequentially with the bicyclic aziridine **1** (0.5 mmol, 1 equiv.), the Kobayashi aryne precursor **2** (0.5 mmol, 1 equiv.), tetrabutylammonium triflate (0.5 mmol, 1 equiv.), dry THF (0.5 M), and CsF (0.5 mmol, 1 equiv.) (*NOTE: It is imperative to use freshly dried CsF to achieve reproducible results*). The vial was then sealed using a Teflon-lined cap, the top part was wrapped in parafilm and stirred at room temperature for 8 h. The resulting mixture was diluted in dichloromethane, filtered over a pad of Celite, and concentrated under reduced pressure. The crude product was purified by column chromatography using a Combiflash® RF (Teledyne ISCO), equipped with a 24 g silica gel disposable flash column (gradient method, EtOAc in hexanes, 2-10% increments).

6.3. General procedure B

In a nitrogen-filled dry box, a flame-dried 4-dram vial containing a magnetic stir bar was charged sequentially with the bicyclic aziridine **1** (0.5 mmol, 1 equiv.), the Kobayashi aryne precursor **2** (0.5 mmol, 1 equiv.), dry MeCN (0.5 M), and CsF (1.5 mmol, 3 equiv.) (*NOTE: It is imperative to use freshly dried CsF to achieve reproducible results*). The vial was then sealed using a Teflon-lined cap, the top part was wrapped in parafilm and stirred at room temperature for 2 h. The resulting mixture was diluted in dichloromethane, filtered over a pad of Celite, and concentrated under reduced pressure. The crude product was purified by column chromatography using a Combiflash® RF (Teledyne ISCO), equipped with a 24 g silica gel disposable flash column (gradient method, EtOAc in hexanes, 2-10% increments).

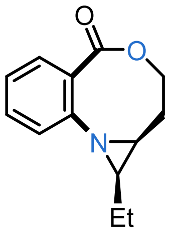

3a

Compound 3a: Following the general procedure A, on a 0.5 mmol scale. The product was purified by column chromatography using a Combiflash® RF (Teledyne ISCO), equipped with a 24 g silica gel disposable flash column (0% – 15% EtOAc in hexanes, 2% increments) to yield **3a** as a white solid (84.7 mg, 0.39 mmol, 78%, >20:1 *dr*). R_f = 0.66 (30% EtOAc/hexanes). ^{1}H NMR (500 MHz, $CDCl_3$) δ 7.57 (dd, *J* = 7.7, 1.5 Hz, 1H), 7.35 (ddd, *J* = 8.0, 7.4, 1.5 Hz, 1H), 6.97 (td, *J* = 7.5, 1.1 Hz, 1H), 6.91 (dd, *J* = 8.1, 1.1 Hz, 1H), 4.94 – 4.86 (m, 1H), 4.47 (dd, *J* = 11.6, 6.4 Hz, 1H), 2.23 (dt, *J* = 8.2, 5.5 Hz, 1H), 2.08 – 2.00 (m, 1H), 1.96 – 1.88 (m, 2H), 1.69 (dddd, J = 14.9, 12.9, 6.9, 5.3 Hz, 1H), 1.64 – 1.54 (m, 1H), 1.18 (t, *J* = 7.4 Hz, 3H). ^{13}C NMR (126 MHz, $CDCl_3$) δ 174.3, 154.8, 133.1, 130.5, 124.5, 121.8, 119.5, 65.7, 43.9, 40.8, 27.8, 22.2, 12.4. HRMS-ESI (*m/z*) calculated for $C_{13}H_{15}NO_2$ $[M+H]^+$ 218.1176; found, 218.1175.

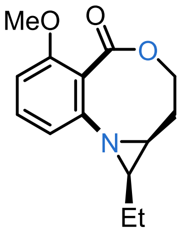

3b

Compound 3b: Following the general procedure A, on a 0.5 mmol scale. The product was purified by column chromatography using a Combiflash® RF (Teledyne ISCO), equipped with a 24 g silica gel disposable flash column (0% – 40% EtOAc in hexanes, 5% increments) to yield **3b** as a white solid (84.1 mg, 0.34 mmol, 68%,

>20:1 *dr*). R_f = 0.22 (40% EtOAc/hexanes). ^{1}H NMR (500 MHz, $CDCl_3$) δ 7.23 (t, *J* = 8.2 Hz, 1H), 6.51 (dd, *J* = 8.2, 3.7 Hz, 2H), 5.10 (td, *J* = 11.7, 5.0 Hz, 1H), 4.33 (dd, *J* = 11.3, 7.3 Hz, 1H), 3.84 (s, 3H), 2.23 (dt, *J* = 8.0, 5.7 Hz, 1H), 2.05 (dddd, *J* = 17.9, 13.3, 5.6, 1.8 Hz, 2H), 1.84 (dtd, *J* = 13.3, 11.6, 7.3 Hz, 1H), 1.65 (ddd, *J* = 14.8, 7.4, 5.5 Hz, 1H), 1.61 – 1.50 (m, 1H), 1.15 (t, *J* = 7.4 Hz, 3H). ^{13}C NMR (126 MHz, $CDCl_3$) δ 171.3, 159.1, 154.8, 132.7, 113.71, 111.8, 104.9, 63.9, 56.2, 44.1, 39.0, 27.8, 22.1, 12.4. HRMS-ESI (*m/z*) calculated for $C_{14}H_{17}NO_3$ $[M+H]^+$ 248.1281; found, 248.1279.

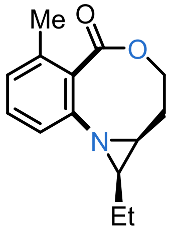

3c

Compound 3c: Following the general procedure A, on a 0.5 mmol scale. The product was purified by column chromatography using a Combiflash® RF (Teledyne ISCO), equipped with a 24 g silica gel disposable flash column (0% – 20% EtOAc in hexanes, 2% increments) to yield **3c** as a white solid (53.2 mg, 0.23 mmol, 46%, >20:1 *dr*). R_f = 0.53 (30% EtOAc/hexanes). ^{1}H NMR (500 MHz, $CDCl_3$) δ 7.17 (t, *J* = 7.8 Hz, 1H), 6.79 (d, *J* = 7.6 Hz, 1H), 6.73 (d, *J* = 8.0 Hz, 1H), 5.09 (td, *J* = 11.6, 5.0 Hz, 1H), 4.36 (dd, *J* = 11.3, 7.3 Hz, 1H), 2.41 (s, 3H), 2.21 (dt, *J* = 8.0, 5.6 Hz, 1H), 2.03 (m, 2H), 1.86 (dtd, *J* = 13.3, 11.8, 7.3 Hz, 1H), 1.72 – 1.50 (m, 2H), 1.16 (t, *J* = 7.4 Hz, 3H). ^{13}C NMR (126 MHz, $CDCl_3$) δ 173.6, 153.8, 139.3, 131.6, 124.1, 123.8, 116.9, 64.1, 44.0, 39.1, 27.8, 22.1, 19.7, 12.4. HRMS-ESI (*m/z*) calculated for $C_{14}H_{17}NO_2$ $[M+H]^+$ 232.1332; found, 232.1331.

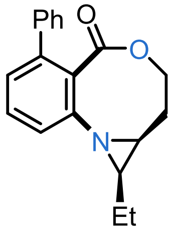

3d

Compound 3d: Following the general procedure A, on a 0.5 mmol scale. The product was purified by column chromatography using a Combiflash® RF (Teledyne ISCO), equipped with a 24 g silica gel disposable flash column (0% – 30% EtOAc in hexanes, 2% increments) to yield **3d** as an off-white solid (99.7 mg, 0.34 mmol, 68%, >20:1 *dr*). R_f = 0.66 (30% EtOAc/hexanes). ^{1}H NMR (500 MHz, $CDCl_3$) δ 7.41 – 7.29 (m, 6H), 6.99 (d, *J* = 7.6 Hz, 1H), 6.87 (d, *J* = 8.0 Hz, 1H), 5.07 (td, *J* = 11.6, 5.1 Hz, 1H), 4.37 (dd, *J* = 11.3, 7.2 Hz, 1H), 2.33 (q, *J* = 6.3 Hz, 1H), 2.07 (dddd, *J* = 29.5, 13.3, 5.5, 1.8 Hz, 1H), 2.07 (dddd, *J* = 29.5, 13.3, 5.5, 1.8 Hz, 2H), 1.90 (dddd, *J* = 13.3, 11.9, 11.5, 7.4 Hz, 1H), 1.69 – 1.51 (m, 1H), 1.45 (dp, *J* = 14.7, 6.9 Hz, 2H), 0.97 (t, *J* = 7.3 Hz,

3H). ^{13}C NMR (126 MHz, $CDCl_3$) δ 173.6, 153.3, 143.0, 140.2, 131.5, 128.7, 128.6, 127.8, 123.9, 123.6, 117.9, 64.1, 42.7, 38.9, 28.6, 22.8, 14.2. HRMS-ESI (*m/z*) calculated for $C_{19}H_{19}NO_2$ $[M+H]^+$ 294.1489; found, 294.1483.

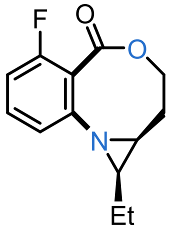

3e

Compound 3e: Following the general procedure A, on a 0.5 mmol scale. The product was purified by column chromatography using a Combiflash® RF (Teledyne ISCO), equipped with a 24 g silica gel disposable flash column (0% – 40% EtOAc in hexanes, 5% increments) to yield **3e** as a white solid (72.9 mg, 0.31 mmol, 62%, >20:1 *dr*). $R_f = 0.73$ (50% EtOAc/hexanes). ^1H NMR (500 MHz, $CDCl_3$) δ 7.26 (dt, $J = 8.2, 6.0$ Hz, 1H), 6.67 (td, $J = 8.4, 1.1$ Hz, 2H), 5.04 (td, $J = 11.8, 4.8$ Hz, 1H), 4.41 (dd, $J = 11.4, 7.1$ Hz, 1H), 2.28 (dt, $J = 8.1, 5.7$ Hz, 1H), 2.08 – 2.01 (m, 2H), 1.87 (dtd, $J = 13.5, 11.9, 7.2$ Hz, 1H), 1.73 – 1.52 (m, 2H), 1.16 (t, $J = 7.4$ Hz, 3H). ^{13}C NMR (126 MHz, $CDCl_3$) δ 169.4, 161.43 (d, $J = 255.3$ Hz), 155.53 (d, $J = 4.3$ Hz), 133.23 (d, $J = 10.4$ Hz), 114.86 (d, $J = 3.2$ Hz), 113.26 (d, $J = 15.7$ Hz), 109.18 (d, $J = 21.3$ Hz), 65.1, 44.5, 39.8, 27.7, 22.0, 12.4. ^{19}F NMR (376 MHz, $CDCl_3$) δ -114.4. HRMS-ESI (*m/z*) calculated for $C_{13}H_{14}FNO_2$ $[M+H]^+$ 236.1081; found, 236.1077.

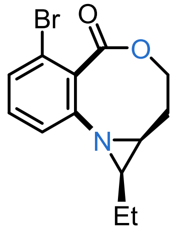

3f

Compound 3f: Following the general procedure A, on a 0.5 mmol scale. The product was purified by column chromatography using a Combiflash® RF (Teledyne ISCO), equipped with a 24 g silica gel disposable flash column (0% – 30% EtOAc in hexanes, 2% increments) to yield **3f** as a white solid (68.1 mg, 0.23 mmol, 45%, >20:1 *dr*). $R_f = 0.44$ (30% EtOAc/hexanes). ^1H NMR (500 MHz, $CDCl_3$) δ 7.19 – 7.09 (m, 2H), 6.83 (dd, $J = 7.8, 1.0$ Hz, 1H), 5.14 (td, $J = 11.6, 5.2$ Hz, 1H), 4.40 (dd, $J = 11.3, 7.4$ Hz, 1H), 2.29 (dt, $J = 8.0, 5.8$ Hz, 1H), 2.09 (dddd, $J = 24.5, 13.5, 5.6, 1.8$ Hz, 2H), 1.85 (dtd, $J = 13.3, 11.6, 7.4$ Hz, 1H), 1.66 (dqd, $J = 14.9, 7.5, 5.4$ Hz, 1H), 1.62 – 1.52 (m, 1H), 1.16 (t, $J = 7.4$ Hz, 3H). ^{13}C NMR (126 MHz, $CDCl_3$) δ 171.4, 154.7, 132.4, 126.5, 125.4, 121.6, 118.2, 64.4, 44.6, 39.1, 27.7, 22.0, 12.4. HRMS-ESI (*m/z*) calculated for $C_{13}H_{14}BrNO_2$ $[M+H]^+$ 296.0281; found, 296.0277.

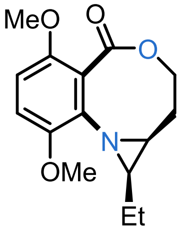

3g

Compound 3g: Following the general procedure A over 24 h, on a 0.5 mmol scale. The product was purified by column chromatography using a Combiflash® RF (Teledyne ISCO), equipped with a 24 g silica gel disposable flash column (0% – 70% EtOAc in hexanes, 5% increments) to yield **3g** as a white solid (12.5 mg, 0.05 mmol, 9%, >20:1 *dr*). R_f = 0.44 (50% EtOAc/hexanes). ^{1}H NMR (500 MHz, $CDCl_3$) δ 6.81 (d, *J* = 8.9 Hz, 1H), 6.42 (d, *J* = 8.9 Hz, 1H), 5.12 (td, *J* = 11.7, 5.0 Hz, 1H), 4.30 (dd, *J* = 11.3, 7.3 Hz, 1H), 3.79 (s, 3H), 3.78 (s, 3H), 2.36 (dt, *J* = 7.4, 5.9 Hz, 1H), 2.07 – 1.98 (m, 2H), 1.95 – 1.78 (m, 2H), 1.51 (dp, *J* = 14.9, 7.5 Hz, 1H), 1.09 (t, *J* = 7.5 Hz, 3H). ^{13}C NMR (126 MHz, $CDCl_3$) δ 171.4, 152.6, 145.7, 143.4, 114.9, 114.6, 104.0, 63.3, 56.5, 56.3, 44.2, 39.4, 27.6, 22.5, 11.4. HRMS-ESI (*m/z*) calculated for $C_{15}H_{19}NO_4$ $[M+H]^+$ 278.1387; found, 278.1386.

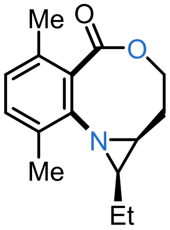

3h

Compound 3g: Following the general procedure A over 24 h, on a 0.5 mmol scale. The product was purified by column chromatography using a Combiflash® RF (Teledyne ISCO), equipped with a 24 g silica gel disposable flash column (0% – 30% EtOAc in hexanes, 2% increments) to yield **3h** as a white solid (8.6 mg, 0.04 mmol, 7%, >20:1 *dr*). R_f = 0.33 (30% EtOAc/hexanes). ^{1}H NMR (500 MHz, $CDCl_3$) δ 7.03 (d, *J* = 7.7 Hz, 1H), 6.70 (d, *J* = 7.7 Hz, 1H), 5.16 (td, *J* = 11.6, 5.2 Hz, 1H), 4.32 (dd, *J* = 11.2, 7.5 Hz, 1H), 2.36 (s, 3H), 2.29 – 2.22 (m, 4H), 2.19 – 2.04 (m, 3H), 1.85 (dtd, *J* = 13.1, 11.7, 7.5 Hz, 1H), 1.41 (ddq, J = 14.8, 9.7, 7.6 Hz, 1H), 1.04 (t, *J* = 7.6 Hz, 3H). ^{13}C NMR (126 MHz, $CDCl_3$) δ 174.1, 150.9, 136.5, 133.1, 126.4, 123.9, 123.6, 63.6, 43.3, 39.5, 27.3, 22.3, 19.5, 18.5, 11.3. HRMS-ESI (*m/z*) calculated for $C_{15}H_{19}NO_2$ $[M+H]^+$ 246.1489; found, 246.1484.

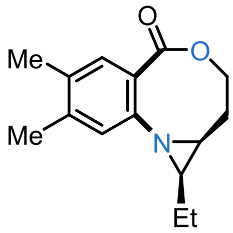

3i

Compound 3i: Following the general procedure A, on a 0.5 mmol scale. The product was purified by column chromatography using a Combiflash® RF (Teledyne ISCO), equipped with a 24 g silica gel disposable flash column (0% – 30% EtOAc in hexanes, 2% increments) to yield **3i** as a white solid (79.7 mg, 0.36 mmol, 65%, >20:1 *dr*). R_f = 0.47 (30% EtOAc/hexanes). ^{1}H NMR (500 MHz, $CDCl_3$) δ 7.35 (s, 1H), 6.67 (s, 1H), 4.84 (td, *J* = 11.6, 4.7 Hz, 1H), 4.46 (dd, *J* = 11.4, 6.6 Hz, 1H), 2.23 (s, 3H), 2.20 – 2.13 (m, 4H), 2.01 (ddd, *J* = 13.0, 4.6, 1.5 Hz, 1H), 1.90 (dtd, *J* = 12.7, 11.4, 6.6 Hz, 1H), 1.82 (ddd, *J* = 11.2, 6.0, 1.5 Hz, 1H), 1.71 – 1.53 (m, 2H), 1.18 (t, *J* = 7.4 Hz, 3H). ^{13}C NMR (126 MHz, $CDCl_3$) δ 174.5, 153.2, 142.6, 131.3, 130.2, 121.9, 120.5, 65.8. 43.9, 41.2, 27.9, 22.3, 20.3, 19.0, 12.4. HRMS-ESI (*m/z*) calculated for $C_{15}H_{19}NO_2$ $[M+H]^+$ 246.1488; found, 246.1486.

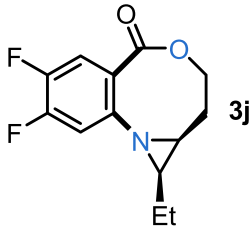

3j

Compound 3j: Following the general procedure A, on a 0.5 mmol scale. The product was purified by column chromatography using a Combiflash® RF (Teledyne ISCO), equipped with a 24 g silica gel disposable flash column (0% – 40% EtOAc in hexanes, 2% increments) to yield **3j** as a white solid (86.1 mg, 0.34 mmol, 67%, >20:1 *dr*). R_f = 0.53 (30% EtOAc/hexanes). ^{1}H NMR (500 MHz, $CDCl_3$) δ 7.40 (t, *J* = 9.2 Hz, 1H), 6.69 (dd, *J* = 11.0, 6.7 Hz, 1H), 4.91 – 4.79 (m, 1H), 4.52 – 4.45 (m, 1H), 2.22 (dt, *J* = 7.8, 5.6 Hz, 1H), 2.04 (dd, *J* = 11.6, 4.7 Hz, 1H), 1.90 (pd, *J* = 11.6, 6.2 Hz, 2H), 1.68 (ddd, *J* = 13.6, 7.3, 5.5 Hz, 1H), 1.57 (dp, *J* = 14.8, 7.5 Hz, 1H), 1.17 (t, *J* = 7.4 Hz, 3H). ^{13}C NMR (126 MHz, $CDCl_3$) δ 172.1 (d, J = 2.0 Hz), 153.0 (dd, J = 255.3, 13.8 Hz), 152.4 (dd, J = 8.2, 2.5 Hz), 145.5 (dd, J = 244.2, 13.3 Hz), 120.3 (dd, J = 5.1, 3.3 Hz), 119.0 (dd, J = 19.1, 2.4 Hz), 108.3 (d, J = 19.4 Hz), 66.1, 44.4, 41.7, 27.6, 21.9, 12.3. ^{19}F NMR (376 MHz, $CDCl_3$) δ -129.04 (d, *J* = 21.1 Hz), -145.35 (d, *J* = 21.8 Hz). HRMS-ESI (*m/z*) calculated for $C_{13}H_{13}F_2NO_2$ $[M+H]^+$ 254.0987; found, 254.0984.

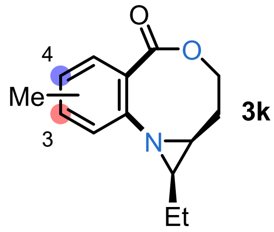

3k

Compound 3k: Following the general procedure A, on a 0.5 mmol scale. The inseparable mixture of **3k-4-Me** and **3k-3-Me** was purified by column chromatography using a Combiflash® RF (Teledyne ISCO), equipped with a 24 g silica gel disposable flash column (0% – 30% EtOAc in hexanes, 2% increments) to yield the mixture **3k**

(**3k-4-Me** + **3k-3-Me**) as a white solid (71.7 mg, 0.31 mmol, 61%, >20:1 *dr*, 1:1.3 rr, 4-Me:3-Me). R_f = 0.62 (30% EtOAc/hexanes). ^{1}H NMR (500 MHz, $CDCl_3$) (**major regioisomer, 3k-3-Me**) δ 7.47 (d, *J* = 7.8 Hz, 1H), 6.78 (d, *J* = 7.8 Hz, 1H), 6.70 (s, 1H), 4.93 – 4.80 (m, 1H)*, 4.51 – 4.43 (m, 1H)*, 2.32 (s, 3H), 2.23 – 2.14 (m, 1H)*, 2.06 – 1.98 (m, 1H)*, 1.95 – 1.83 (m, 2H)*, 1.73 – 1.52 (m, 2H)*, 1.21 – 1.14 (m, 3H)*. ^{1}H NMR (500 MHz, $CDCl_3$) (**minor regioisomer, 3k-4-Me**) δ 7.38 (d, *J* = 1.4 Hz, 1H), 7.15 (dd, *J* = 8.1, 1.6 Hz, 1H), 6.81 (d, *J* = 8.2 Hz, 1H), 4.93 – 4.80 (m, 1H)*, 4.51 – 4.43 (m, 1H)*, 2.28 (s, 3H), 2.23 – 2.14 (m, 1H)*, 2.06 – 1.98 (m, 1H)*, 1.95 – 1.83 (m, 2H)*, 1.73 – 1.52 (m, 2H)*, 1.21 – 1.14 (m, 3H)*. ^{13}C NMR (126 MHz, $CDCl_3$) (**major regioisomer, 3k-3-Me**) δ 174.3, 155.1, 144.0, 130.6, 122.8, 121.9, 119.9, 65.8*, 43.8, 41.0*, 27.9*, 22.2*, 21.9, 12.4*. ^{13}C NMR (126 MHz, $CDCl_3$) (**minor regioisomer, 3k-4-Me**) δ 174.5, 152.7, 133.9, 131.3, 130.7, 124.3, 119.4, 65.8*, 44.0, 41.0*, 27.9*, 22.2*, 20.6, 12.4*. HRMS-ESI (*m/z*) calculated for $C_{14}H_{17}NO_2$ $[M+H]^+$ 232.1332; found, 232.1330. *peaks overlapped.

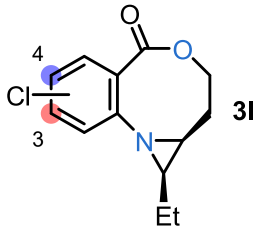

3l

Compound 3l: Following the general procedure, on a 0.5 mmol scale. The inseparable mixture of **3l-4-Cl** and **3l-3-Cl** was purified by column chromatography using a Combiflash® RF (Teledyne ISCO), equipped with a 24 g silica gel disposable flash column (0% – 30% EtOAc in hexanes, 2% increments) to yield the mixture **3l** (**3l-4-Cl** + **3l-3-Cl**) as a white solid (70.5 mg, 0.28 mmol, 56%, >20:1 *dr*, 2.7:1 rr, 4-Cl:3-Cl). R_f = 0.60 (30% EtOAc/hexanes). ^{1}H NMR (500 MHz, $CDCl_3$) (**major regioisomer, 3l-4-Cl**) δ 7.54 (d, *J* = 2.4 Hz, 1H), 7.29 (dd, *J* = 8.6, 2.4 Hz, 1H), 6.85 (d, *J* = 8.6 Hz, 1H), 4.93 – 4.83 (m, 1H)*, 4.52 – 4.44 (m, 1H)*, 2.30 – 2.18 (m, 1H)*, 2.09 – 2.00 (m, 1H)*, 1.97 – 1.84 (m, 2H)*, 1.74 – 1.63 (m, 1H)*, 1.64 – 1.52 (m, 1H)*, 1.21 – 1.13 (m, 3H)*. ^{1}H NMR (500 MHz, $CDCl_3$) (**minor regioisomer, 3l-3-Cl**) δ 7.50 (d, *J* = 8.2 Hz, 1H), 6.94 (dd, *J* = 8.2, 2.0 Hz, 1H), 6.89 (d, *J* = 1.9 Hz, 1H), 4.93 – 4.83 (m, 1H)*, 4.52 – 4.44 (m, 1H)*, 2.30 – 2.18 (m, 1H)*, 2.09 – 2.00 (m, 1H)*, 1.97 – 1.84 (m, 2H)*, 1.74 – 1.63 (m, 1H)*, 1.64 – 1.52 (m, 1H)*, 1.21 – 1.13 (m, 3H)*. ^{13}C NMR (126 MHz, $CDCl_3$) (**major regioisomer, 3l-4-Cl**) δ 172.9, 153.4, 133.0, 130.0, 126.8, 125.7, 120.9, 66.0*, 44.2*, 41.0, 27.7, 22.1, 12.4*. ^{13}C NMR (126 MHz, $CDCl_3$) (**minor regioisomer, 3l-3-Cl**) δ 173.3, 156.0, 138.8, 131.7, 123.0, 122.1, 119.6, 66.0*, 44.2*, 41.1, 27.6, 22.0, 12.4*. HRMS-ESI (*m/z*) calculated for $C_{13}H_{14}ClNO_2$ $[M+H]^+$ 252.0786; found, 252.0781. *peaks overlaped

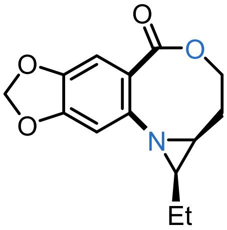

3m

Compound 3m: Following the general procedure A, on a 0.5 mmol scale. The product was purified by column chromatography using a Combiflash® RF (Teledyne ISCO), equipped with a 24 g silica gel disposable flash column (0% – 40% EtOAc in hexanes, 5% increments) to yield **3m** as a white solid (95.4 mg, 0.37 mmol, 73%, >20:1 *dr*). R_f = 0.49 (30% EtOAc/hexanes). ^{1}H NMR (500 MHz, $CDCl_3$) δ 7.03 (s, 1H), 6.41 (s, 1H), 5.97 (d, *J* = 1.2 Hz, 1H), 5.93 (d, *J* = 1.2 Hz, 1H), 4.80 (td, *J* = 11.4, 4.5 Hz, 1H), 4.45 (dd, *J* = 11.5, 6.2 Hz, 1H), 2.14 (dt, *J* = 7.9, 5.3 Hz, 1H), 2.01 (dd, *J* = 11.3, 4.8 Hz, 1H), 1.94 – 1.81 (m, 2H), 1.73 – 1.61 (m, 1H), 1.55 (dp, *J* = 15.1, 7.5 Hz, 1H), 1.16 (t, *J* = 7.4 Hz, 3H). ^{13}C NMR (126 MHz, $CDCl_3$) δ 173.5, 152.4, 151.6, 142.7, 116.7, 109.5, 101.8, 100.5, 66.6, 43.9, 42.2, 27.7, 22.1, 12.4. HRMS-ESI (*m/z*) calculated for $C_{14}H_{15}NO_4$ $[M+H]^+$ 262.1074; found, 262.1069.

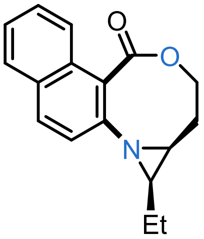

3n

Compound 3n: Following the general procedure A, on a 0.5 mmol scale. The product was purified by column chromatography using a Combiflash® RF (Teledyne ISCO), equipped with a 24 g silica gel disposable flash column (0% – 30% EtOAc in hexanes, 2% increments) to yield **3n** as a pale-yellow solid (72.2 mg, 0.27 mmol, 54%, >20:1 *dr*). R_f = 0.64 (30% EtOAc/hexanes). ^{1}H NMR (500 MHz, $CDCl_3$) δ 8.16 (d, *J* = 8.6 Hz, 1H), 7.79 (d, *J* = 8.8 Hz, 1H), 7.73 (d, *J* = 8.2 Hz, 1H), 7.51 (ddd, *J* = 8.4, 6.8, 1.4 Hz, 1H), 7.35 (ddd, *J* = 8.0, 6.8, 1.1 Hz, 1H), 7.08 (d, *J* = 8.8 Hz, 1H), 5.14 (td, *J* = 11.7, 4.9 Hz, 1H), 4.39 (dd, *J* = 11.2, 7.2 Hz, 1H), 2.33 (dt, *J* = 7.9, 5.8 Hz, 1H), 2.12 (dddd, *J* = 26.0, 13.2, 5.5, 1.8 Hz, 2H), 1.92 (dtd, *J* = 13.1, 11.9, 7.2 Hz, 1H), 1.77 – 1.57 (m, 2H), 1.21 (t, *J* = 7.4 Hz, 3H). ^{13}C NMR (126 MHz, $CDCl_3$) δ 173.2, 152.9, 133.0, 132.1, 129.6, 128.3, 128.1, 124.3, 124.1, 119.8, 117.4, 63.5, 43.3, 40.1, 27.4, 22.2, 12.4. HRMS-ESI (*m/z*) calculated for $C_{17}H_{17}NO_2$ $[M+H]^+$ 268.1332; found, 268.1331.

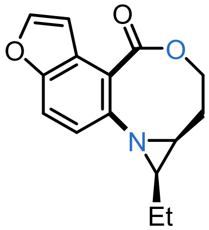

3o

Compound 3o: Following the general procedure A, on a 0.5 mmol scale. The product was purified by column chromatography using a Combiflash® RF (Teledyne ISCO), equipped with a 24 g silica gel disposable flash column (0% – 30% EtOAc in hexanes, 2% increments) to yield **3o** as a pale-yellow solid (90.1 mg, 0.35 mmol, 70%, >20:1 *dr*). R_f = 0.33 (30% EtOAc/hexanes). ^{1}H NMR (500 MHz, $CDCl_3$) δ 7.65 (d, *J* = 2.2 Hz, 1H), 7.46 (d, *J* = 8.7 Hz, 1H), 7.03 – 7.00 (m, 1H), 6.86 (d, *J* = 8.7 Hz, 1H), 4.90 (td, *J* = 11.5, 4.5 Hz, 1H), 4.53 – 4.46 (m, 1H), 2.22 (dt, *J* = 7.8, 5.3 Hz, 1H), 2.06 (dt, *J* = 11.8, 4.2 Hz, 1H), 2.01 – 1.88 (m, 2H), 1.76 – 1.55 (m, 2H), 1.20 (t, *J* = 7.4 Hz, 3H). ^{13}C NMR (126 MHz, $CDCl_3$) δ 172.8, 152.0, 150.7, 147.3, 128.6, 116.4, 115.7, 115.3, 106.5, 65.3, 43.8, 41.8, 27.7, 22.2, 12.4. HRMS-ESI (*m/z*) calculated for $C_{15}H_{15}NO_3$ $[M+H]^+$ 258.1125; found, 258.1121.

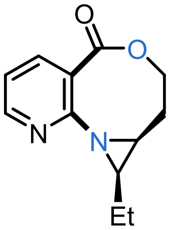

3p

Compound 3p: Following the general procedure B, on a 0.5 mmol scale. The product was purified by column chromatography using a Combiflash® RF (Teledyne ISCO), equipped with a 24 g silica gel disposable flash column (0% – 70% EtOAc in hexanes, 5% increments) to yield **3p** as a pale-brown solid (48.0 mg, 0.22 mmol, 44%, >20:1 *dr*). R_f = 0.24 (40% EtOAc/hexanes). ^{1}H NMR (500 MHz, $CDCl_3$) δ 8.36 (dd, *J* = 4.9, 1.9 Hz, 1H), 7.87 (dd, *J* = 7.5, 1.9 Hz, 1H), 6.93 (dd, *J* = 7.5, 4.9 Hz, 1H), 4.89 (td, *J* = 11.7, 4.6 Hz, 1H), 4.53 (dd, *J* = 11.4, 6.7 Hz, 1H), 2.60 (q, *J* = 6.6 Hz, 1H), 2.05 (dtd, *J* = 12.7, 4.5, 1.7 Hz, 2H), 1.93 (dtd, *J* = 13.7, 11.9, 6.9 Hz, 1H), 1.84 (dp, *J* = 14.2, 7.4 Hz, 1H), 1.60 (dp, *J* = 14.6, 7.4 Hz, 1H), 1.15 (t, *J* = 7.5 Hz, 3H). ^{13}C NMR (126 MHz, $CDCl_3$) δ 172.9, 164.2, 152.1, 139.0, 119.9, 117.5, 66.5, 42.7, 40.4, 27.1, 22.1, 11.8. HRMS-ESI (*m/z*) calculated for $C_{12}H_{14}N_2O_2$ $[M+H]^+$ 219.1128; found, 219.1126.

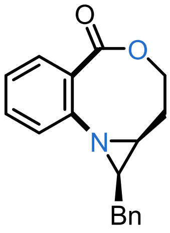

3q

Compound 3q: Following the general procedure A, on a 0.5 mmol scale. The product was purified by column chromatography using a Combiflash® RF (Teledyne ISCO), equipped with a 24 g silica gel disposable flash column (0% – 40% EtOAc in hexanes, 2% increments) to yield **3q** as a colorless oil (71.2 mg, 0.26 mmol, 51%, >20:1 *dr*). R_f = 0.60 (30% EtOAc/hexanes). ^{1}H NMR (500 MHz, $CDCl_3$) δ 7.55 (dd, J = 7.7, 1.6 Hz, 1H), 7.39 – 7.32 (m, 4H), 7.31 – 7.26 (m, 1H), 7.24 – 7.18 (m, 1H), 6.92 (td, J = 7.5, 1.1 Hz, 1H), 6.48 (dd, J = 8.1, 1.1 Hz, 1H), 4.91 (td, J = 11.4, 5.1 Hz, 1H), 4.54 – 4.48 (m, 1H), 2.99 – 2.84 (m, 2H), 2.48 (dt, J = 7.8, 5.7 Hz, 1H), 2.15 – 2.01 (m, 2H), 1.97 (ddd, J = 10.8, 6.0, 2.2 Hz, 1H). ^{13}C NMR (126 MHz, $CDCl_3$) δ 174.2, 154.5, 139.1, 133.1, 130.4, 129.0, 128.9, 126.8, 124.3, 121.8, 119.4, 65.7, 43.8, 40.9, 35.6, 28.1. HRMS-ESI (*m/z*) calculated for $C_{18}H_{17}NO_2$ $[M+H]^+$ 280.1332; found, 280.1330.

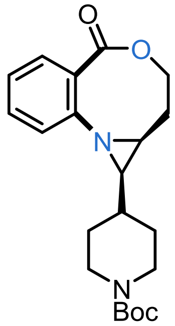

3r

Compound 3r: Following the general procedure A, on a 0.5 mmol scale. The product was purified by column chromatography using a Combiflash® RF (Teledyne ISCO), equipped with a 24 g silica gel disposable flash column (0% – 70% EtOAc in hexanes, 5% increments) to yield **3r** as a white solid (156.4 mg, 0.42 mmol, 83%, >20:1 *dr*). R_f = 0.31 (30% EtOAc/hexanes). ^{1}H NMR (500 MHz, $CDCl_3$) δ 7.58 (dd, J = 7.6, 1.6 Hz, 1H), 7.35 (td, J = 7.8, 1.6 Hz, 1H), 6.99 (td, J = 7.8, 1.6 Hz, 1H), 6.85 (d, J = 8.1 Hz, 1H), 4.91 (td, J = 11.8, 5.0 Hz, 1H), 4.48 (dd, J = 11.4, 6.3 Hz, 1H), 4.21 (bs, 2H), 2.84 – 2.64 (m, 2H), 2.12 – 1.88 (m, 5H), 1.71 – 1.61 (m, 1H), 1.48 (s, 9H), 1.55 – 1.31 (m, 3H). ^{13}C NMR (126 MHz, $CDCl_3$) δ 174.1, 155.0, 154.4, 133.2, 130.6, 124.5, 122.5, 119.3, 79.7, 65.6, 46.9, 40.6, 36.3, 31.3, 29.3, 28.6, 27.9. HRMS-ESI (*m/z*) calculated for $C_{21}H_{28}N_2O_4$ $[M+H]^+$ 373.2122; found, 373.2116.

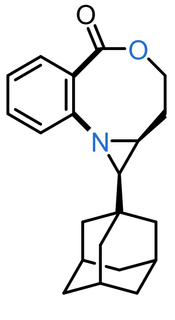

3s

Compound 3s: Following the general procedure A, on a 0.5 mmol scale. The product was purified by column chromatography using a Combiflash® RF (Teledyne ISCO), equipped with a 24 g silica gel disposable flash column (0% – 30% EtOAc in hexanes, 2% increments) to yield **3s** as a white solid (93.8 mg, 0.29 mmol, 57%, >20:1 *dr*). R_f = 0.62 (30% EtOAc/hexanes). ^{1}H NMR (500 MHz, $CDCl_3$) δ 7.56 (dd, *J* = 8.0, 1.6 Hz, 1H), 7.35 (td, *J* = 8.1, 1.6 Hz, 1H), 7.00 – 6.94 (m, 2H), 4.79 (ddd, *J* = 12.2, 11.2, 4.3 Hz, 1H), 4.44 (dd, *J* = 11.2, 6.7 Hz, 1H), 2.35 – 2.22 (m, 1H), 2.11 – 2.02 (m, 4H), 1.88 – 1.66 (m, 14H). ^{13}C NMR (126 MHz, $CDCl_3$) δ 174.3, 156.2, 133.1, 130.5, 124.5, 121.9, 120.4, 66.6, 53.5, 41.9, 41.5, 37.0, 33.8, 29.5, 28.5. HRMS-ESI (*m/z*) calculated for $C_{21}H_{25}NO_2$ $[M+H]^+$ 324.1958; found, 324.1956.

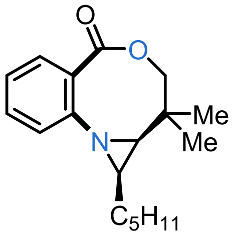

3t

Compound 3t: Following the general procedure A, on a 0.5 mmol scale. The product was purified by column chromatography using a Combiflash® RF (Teledyne ISCO), equipped with a 24 g silica gel disposable flash column (0% – 30% EtOAc in hexanes, 2% increments) to yield **3t** as a white solid (97.7 mg, 0.34 mmol, 68%, >20:1 *dr*). R_f = 0.64 (30% EtOAc/hexanes). ^{1}H NMR (500 MHz, $CDCl_3$) δ 7.59 (dd, *J* = 8.2, 1.5 Hz, 1H), 7.35 (td, *J* = 7.9, 1.6 Hz, 1H), 6.99 – 6.93 (m, 2H), 4.44 (d, *J* = 11.0 Hz, 1H), 3.92 (d, *J* = 10.9 Hz, 1H), 2.12 (ddd, *J* = 7.9, 6.2, 5.1 Hz, 1H), 1.84 – 1.61 (m, 4H), 1.60 – 1.50 (m, 1H), 1.46 – 1.33 (m, 4H), 1.14 (s, 3H), 1.05 (s, 3H), 0.94 (t, *J* = 7.1 Hz, 3H). ^{13}C NMR (126 MHz, $CDCl_3$) δ 174.0, 156.1, 133.2, 130.8, 123.9, 121.7, 120.5, 79.6, 49.2, 44.4, 36.3, 32.0, 29.5, 28.8, 23.3, 23.0, 22.7, 14.2. HRMS-ESI (*m/z*) calculated for $C_{18}H_{25}NO_2$ $[M+H]^+$ 288.1958; found, 288.1954.

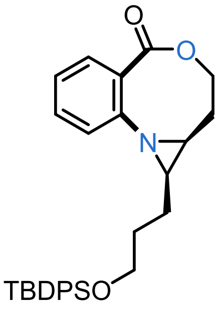

3u

Compound 3u: Following the general procedure A, on a 0.5 mmol scale. The product was purified by column chromatography using a Combiflash® RF (Teledyne ISCO), equipped with a 24 g silica gel disposable flash column (0% – 20% EtOAc in hexanes, 2% increments) to yield **3u** as a white solid (187.0 mg, 0.39 mmol, 77%,

>20:1 *dr*). R_f = 0.76 (30% EtOAc/hexanes). ^{1}H NMR (500 MHz, $CDCl_3$) δ 7.67 (dd, *J* = 7.8, 1.2 Hz, 4H), 7.57 (dd, *J* = 7.7, 1.3 Hz, 1H), 7.45 – 7.35 (m, 6H), 7.32 (td, *J* = 7.7, 1.4 Hz, 1H), 6.97 (t, *J* = 7.5 Hz, 1H), 6.86 (d, *J* = 8.1 Hz, 1H), 4.89 (td, *J* = 11.5, 5.1 Hz, 1H), 4.46 (dd, *J* = 11.5, 5.9 Hz, 1H), 3.83 – 3.71 (m, 2H), 2.26 (dt, *J* = 7.3, 5.3 Hz, 1H), 1.99 (dd, *J* = 11.3, 4.8 Hz, 1H), 1.94 – 1.73 (m, 5H), 1.70 – 1.59 (m, 1H), 1.06 (s, 9H). ^{13}C NMR (126 MHz, $CDCl_3$) δ 147.3, 154.8, 135.7, 135.7, 134.0, 134.0, 133.1, 130.5, 129.8, 127.8, 124.5, 121.8, 119.5, 65.8, 63.6, 42.3, 40.7, 31.1, 27.8, 27.0, 25.5, 19.4. HRMS-ESI (*m/z*) calculated for $C_{30}H_{35}NO_3Si$ $[M+H]^+$ 486.2459; found, 486.2455.

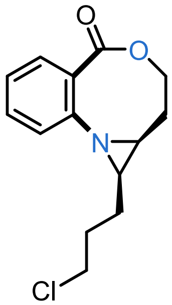

3v

Compound 3v: Following the general procedure A, on a 0.5 mmol scale. The product was purified by column chromatography using a Combiflash® RF (Teledyne ISCO), equipped with a 24 g silica gel disposable flash column (0% – 30% EtOAc in hexanes, 2% increments) to yield **3v** as a white solid (77.1 mg, 0.29 mmol, 57%, >20:1 *dr*). R_f = 0.49 (30% EtOAc/hexanes). ^{1}H NMR (500 MHz, $CDCl_3$) δ 7.58 (dd, *J* = 7.7, 1.4 Hz, 1H), 7.36 (td, *J* = 7.7, 1.5 Hz, 1H), 6.99 (td, *J* = 7.5, 1.1 Hz, 1H), 4.91 (td, *J* = 11.8, 4.9 Hz, 1H), 4.49 (dd, *J* = 11.3, 6.4 Hz, 1H), 3.72 – 3.58 (m, 2H), 2.30 (dt, *J* = 8.0, 5.5 Hz, 1H), 2.19 – 2.00 (m, 3H), 1.99 – 1.84 (m, 3H), 1.74 – 1.65 (m, 1H). ^{13}C NMR (126 MHz, $CDCl_3$) δ 174.1, 154.5, 133.2, 130.6, 124.6, 122.1, 119.3, 65.7, 44.6, 41.7, 40.7, 31.2, 27.9, 26.5. HRMS-ESI (*m/z*) calculated for $C_{14}H_{16}ClNO_2$ $[M+H]^+$ 266.0942; found, 266.0941.

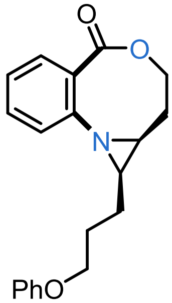

3x

Compound 3x: Following the general procedure A, on a 0.5 mmol scale. The product was purified by column chromatography using a Combiflash® RF (Teledyne ISCO), equipped with a 24 g silica gel disposable flash column (0% – 30% EtOAc in hexanes, 2% increments) to yield **3x** as a colorless oil (98.6 mg, 0.31 mmol, 61%, >20:1 *dr*). R_f = 0.56 (30% EtOAc/hexanes). ^{1}H NMR (500 MHz, $CDCl_3$) δ 7.58 (dd, *J* = 7.7, 1.6 Hz, 1H), 7.35

(td, J = 7.8, 1.6 Hz, 1H), 7.29 (td, J = 7.5, 1.9 Hz, 2H), 7.02 – 6.88 (m, 5H), 4.90 (td, J = 11.7, 5.0 Hz, 1H), 4.48 (dd, J = 11.3, 6.4 Hz, 1H), 4.11 (dt, J = 9.2, 6.0 Hz, 1H), 4.04 (dt, J = 9.2, 6.4 Hz, 1H), 2.38 (dt, J = 7.9, 5.4 Hz, 1H), 2.18 – 2.00 (m, 3H), 2.00 – 1.85 (m, 3H), 1.75 (dtd, J = 14.1, 8.6, 5.9 Hz, 1H). ^{13}C NMR (126 MHz, $CDCl_3$) δ 174.2, 159.0, 154.7, 133.2, 130.6, 129.7, 124.6, 122.0, 120.9, 119.5, 114.6, 67.5, 65.8, 42.2, 40.8, 28.0, 27.8, 26.0. HRMS-ESI (*m/z*) calculated for $C_{20}H_{21}NO_3$ $[M+H]^+$ 324.1594; found, 324.1590.

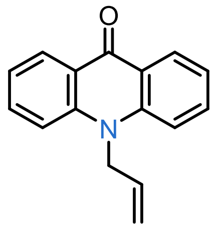

7

Compound 7:[17] Following the general procedure A, on a 0.5 mmol scale. The product was purified by column chromatography using a Combiflash® RF (Teledyne ISCO), equipped with a 24 g silica gel disposable flash column (0% – 50% EtOAc in hexanes, 5% increments) to yield **7** as a pale-yellow solid (41.2 mg, 0.18 mmol, 35%, >20:1 *dr*). R_f = 0.36 (33% EtOAc/hexanes). ^{1}H NMR (500 MHz, $CDCl_3$) δ 8.57 (dd, J = 8.0, 1.7 Hz, 2H), 7.70 (ddd, J = 8.7, 6.9, 1.7 Hz, 2H), 7.42 (d, J = 8.7 Hz, 2H), 7.30 (t, J = 7.5 Hz, 2H), 6.14 (ddt, J = 17.2, 10.6, 3.8 Hz, 1H), 5.32 (dt, J = 10.6, 2.1 Hz, 1H), 5.13 (dt, J = 17.3, 2.2 Hz, 1H), 4.98 (dt, J = 4.0, 2.1 Hz, 1H). ^{13}C NMR (126 MHz, $CDCl_3$) δ 178.4, 142.4, 134.0, 130.8, 127.9, 122.7, 121.7, 117.6, 115.2, 49.5. HRMS-ESI (*m/z*) calculated for $C_{16}H_{13}NO$ $[M+H]^+$ 236.1070; found, 236.1068.

7. Gram-scale reaction procedure

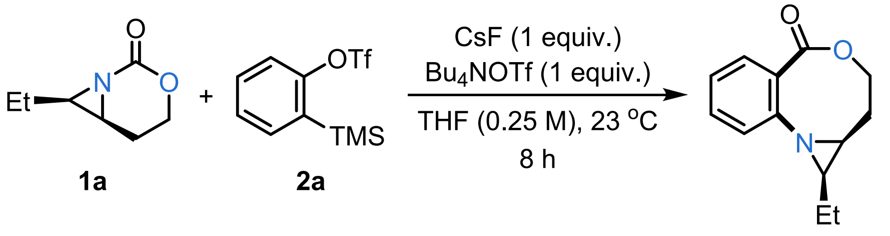

In a nitrogen-filled dry box, a flame-dried round-bottom flask (50 mL) containing a magnetic stir bar was charged sequentially with the bicyclic aziridine **1a** (7.5 mmol, 1 equiv., 1.06 g), the Kobayashi aryne precursor **2a** (7.5 mmol, 1 equiv., 2.24 g), tetrabutylammonium triflate (7.5 mmol, 1 equiv., 2.94 g), dry THF (0.25 M), and CsF (7.5 mmol, 1 equiv., 1.14 g) (*NOTE: It is imperative to use freshly dried CsF to achieve reproducible results*). The flask was then sealed using a Teflon cap, the top part was wrapped in parafilm, and stirred at room temperature for 8 h. The resulting mixture was diluted in dichloromethane, filtered over a pad of Celite, and

concentrated under reduced pressure. The crude product was purified by column chromatography using a Combiflash® RF (Teledyne ISCO), equipped with a 120 g silica gel disposable flash column (0% – 15% EtOAc in hexanes, 2% increments) to yield **3a** as a white solid (1.21 g, 5.55 mmol, 74%, >20:1 *dr*). R_f = 0.66 (30% EtOAc/hexanes).

8. Telescoped intramolecular aziridination/two-carbon ring expansion procedure

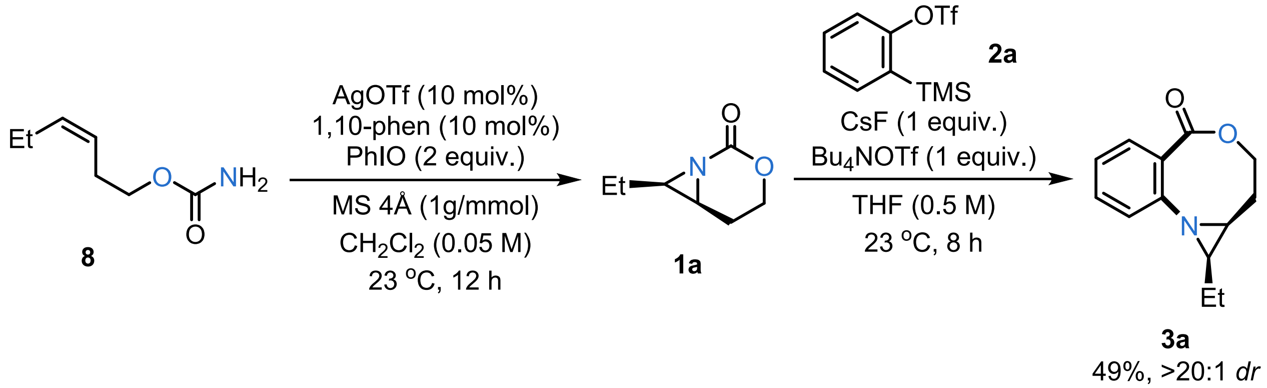

A cooled, flame-dried round bottom flask was charged with $AgClO_4$ or AgOTf (10 mol%) and 1,10-phenanthroline ligand **L2** (10 mol%). Dry CH_2Cl_2 (0.05 M) was added to the flask and the mixture was stirred vigorously for 20 min. After 20 min of pre-stirring, powdered 4Å molecular sieves (1g of sieves /mmol of substrate) were added (*NOTE: It is not necessary to use rigorously dried molecular sieves*). After 5 min, the homoallylic carbamate **8** (0.5 mmol, 1 equiv., 71.6 mg) and recently prepared iodosobenzene (2 equiv.) were added. The reaction was then capped, covered in aluminum foil and allowed to stir at room temperature. After 12 h, the reaction mixture was filtered over Celite and the filter bed was washed with CH_2Cl_2 (50 mL). The filtrate was concentrated under reduced pressure, and the crude reaction was used in the next step without purification. In a nitrogen-filled dry box, a flame-dried 4-dram vial containing a magnetic stir bar was charged sequentially with the crude bicyclic aziridine **1a**, the Kobayashi aryne precursor **2a** (0.5 mmol, 1 equiv., 149.2 mg), tetrabutylammonium triflate (0.5 mmol, 1 equiv., 195.8 mg), dry THF (0.5 M), and CsF (0.5 mmol, 1 equiv., 76.0 mg) (*NOTE: It is imperative to use freshly dried CsF to achieve reproducible results*). The vial was then sealed using a Teflon-lined cap, the top part was wrapped in parafilm and stirred at room temperature for 8 h. The resulting mixture was diluted in dichloromethane, filtered over a pad of Celite, and concentrated under reduced pressure. The crude product was purified by column chromatography using a Combiflash® RF (Teledyne ISCO), equipped with a 24 g silica gel disposable flash column (0% – 15% EtOAc in hexanes, 2% increments) to yield **3a** as a white solid (53.2 mg, 0.24 mmol, 49%, >20:1 *dr*). R_f = 0.66 (30% EtOAc/hexanes).

9. Stereochemical retention experiment procedure

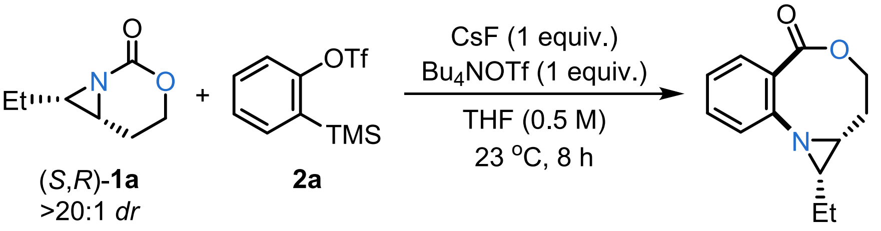

(*S*,*R*)-**1a**
>20:1 *dr*
79:21 *er*

2a

(*S*,*R*)-**3a**
80%, >20:1 *dr*
79:21 *er*

In a nitrogen-filled dry box, a flame-dried 4-dram vial containing a magnetic stir bar was charged sequentially with the bicyclic aziridine (*S*,*R*)-**1a** (79:21 *er*) (0.5 mmol, 1 equiv., 70.6 mg), the Kobayashi aryne precursor **2a** (0.5 mmol, 1 equiv., 149.2 mg), tetrabutylammonium triflate (0.5 mmol, 1 equiv., 195.8 mg), dry THF (0.5 M), and CsF (0.5 mmol, 1 equiv., 76.0 mg) (*NOTE: It is imperative to use freshly dried CsF to achieve reproducible results*). The vial was then sealed using a Teflon-lined cap, the top part was wrapped in parafilm, and stirred at room temperature for 8 h. The resulting mixture was diluted in dichloromethane, filtered over a pad of Celite, and concentrated under reduced pressure. The crude product was purified by column chromatography using a Combiflash® RF (Teledyne ISCO), equipped with a 24 g silica gel disposable flash column (0% – 15% EtOAc in hexanes, 2% increments) to yield (*S*,*R*)-**3a** as a white solid (86.9 mg, 0.40 mmol, 80%, 79:21 *er*, >20:1 *dr*). R_f = 0.66 (30% EtOAc/hexanes).

<u>HPLC conditions</u>:[18] Shimadzu LC-20AB instrument equipped with a CHIRALPAK® AD-H column (4.6 mm diameter x 25 mm length, particle size 5 mm). Flow rate: 0.7 ml min^{-1}; Oven temperature: 40 °C; Wavelength: 254 nm; Solvent: 5% to 25% *i*-PrOH/hexanes gradient over 14 min, then 25% *i*-PrOH/hexanes over 11 min, then 25% to 5% *i*-PrOH/hexanes gradient over 3 min, then 5% *i*-PrOH/hexanes over 2 min.

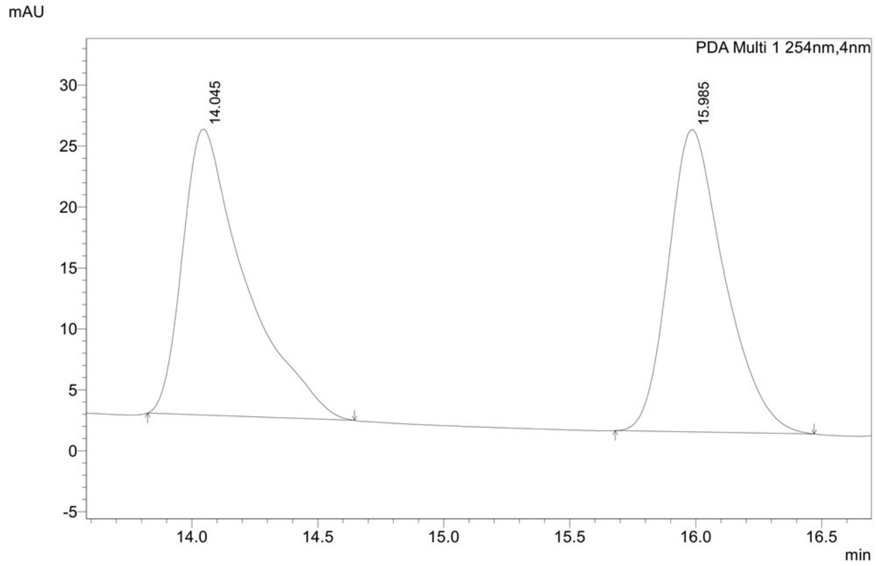

Peak #	Retention time (min)	Area	Area %
1	14.045	407030	51.308
2	15.985	386271	48.692
**Total**		793301	100.000

Figure S3 HPLC chromatogram of racemic bicyclic aziridine **1a**.

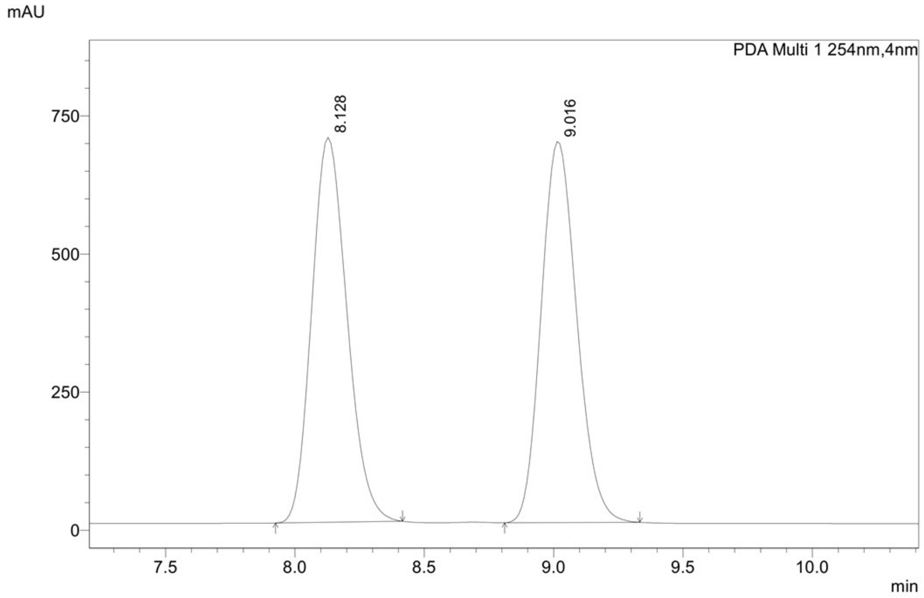

Peak #	Retention time (min)	Area	Area %
1	8.128	6599139	49.972
2	9.016	6606518	50.028
**Total**		13205657	100.000

Figure S4 HPLC chromatogram of racemic oxazocine **3a**.

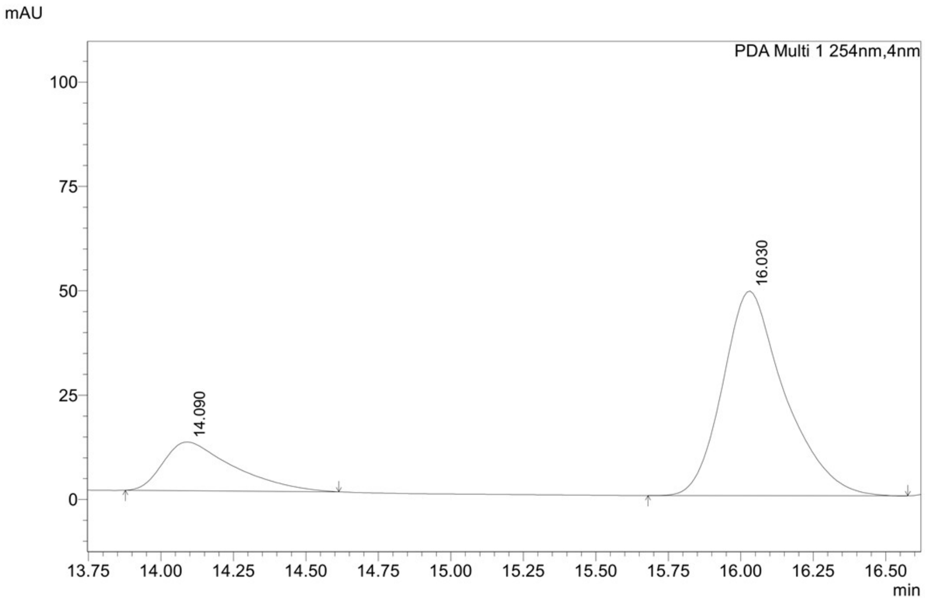

Peak #	Retention time (min)	Area	Area %
1	14.090	197945	21.276
2	16.030	732434	78.724
**Total**		930378	100.000

Figure S5 HPLC chromatogram of scalemic bicyclic aziridine (*S*,*R*)-**1a**.

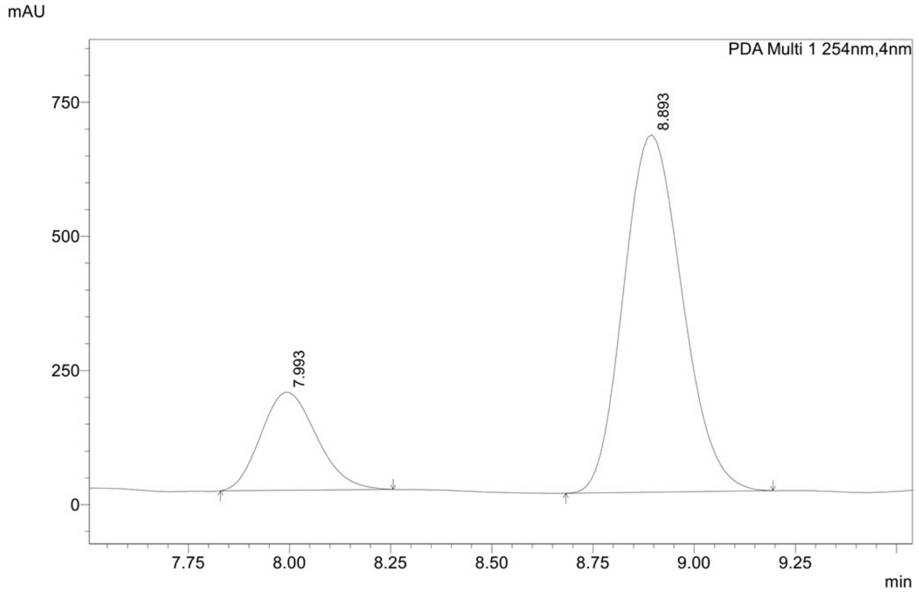

Peak #	Retention time (min)	Area	Area %
1	7.993	1755225	20.714
2	8.893	6718553	79.286
**Total**		8473779	100.000

Figure S5 HPLC chromatogram of scalemic oxazocine (*S*,*R*)-**3a**.

10. Synthetic applications procedures and characterization

10.1 Reduction of 3a with $LiAlH_4$[19]

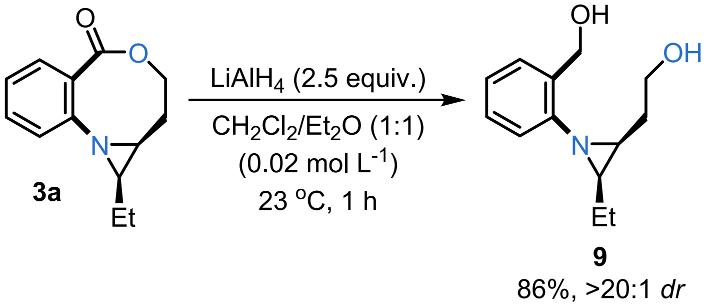

In a flame-dried two-neck round bottom flask under N_2 atmosphere, the oxazocine **3a** (1 equiv., 0.25 mmol, 54.3 mg) was dissolved in a mixture of dry CH_2Cl_2/Et_2O (1:1, 0.02 M). The reaction mixture was cooled to 0 °C, and $LiAlH_4$ (2.5 equiv.) was added portion-wise over 30 min. After, the ice water bath was removed, and the reaction was allowed to warm up to room temperature and stir until the TLC indicated completion (30 min). Once completed, the reaction was quenched with saturated aqueous NH_4Cl solution (5 mL) and extracted with Et_2O (3 × 10 mL). The combined organic layer was washed with brine (3 × 10 mL) and dried over Na_2SO_4. After the solids were filtered off, the solvent was removed under reduced pressure and the residue was purified by column chromatography using a Combiflash® RF (Teledyne ISCO), equipped with a 24 g silica gel disposable flash column (0% – 10% methanol in dichloromethane, 1% increments) to yield **9** as a colorless oil (47.6 mg, 0.22 mmol, 86%, >20:1 *dr*). $R_f = 0.44$ (10% MeOH/hexanes). ^1H NMR (500 MHz, $CDCl_3$) δ 7.24 (d, J = 7.4 Hz, 1H), 7.17 (t, J = 7.7 Hz, 1H), 6.97 (t, J = 7.4 Hz, 1H), 6.89 (d, J = 7.9 Hz, 1H), 4.81 (d, J = 12.8 Hz, 1H), 4.66 (d, J = 12.8 Hz, 1H), 3.89 – 3.78 (m, 2H), 3.60 (bs, 1H), 2.75 (bs, 1H), 2.40 (q, J = 6.5 Hz, 1H), 2.13 (q, J = 6.7 Hz, 1H), 1.94 – 1.80 (m, 3H), 1.54 (dp, J = 14.8, 7.5 Hz, 1H), 1.08 (t, J = 7.5 Hz, 3H). ^{13}C NMR (126 MHz, $CDCl_3$) δ 151.8, 133.7, 128.6, 128.5, 123.1, 119.5, 62.6, 61.3, 46.2, 41.8, 30.7, 21.7, 11.9. HRMS-ESI (*m/z*) calculated for $C_{13}H_{19}NO_2$ $[M+H]^+$ 222.1489; found, 222.1485.

10.2 Synthesis of the diazocine 10 from 9[20]

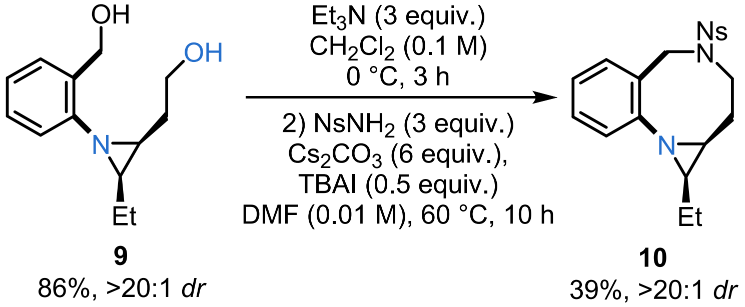

In a flame-dried two-neck round bottom flask under N_2 atmosphere, the diol **9** (1 equiv., 0.085 mmol, 18.8 mg) was dissolved in CH_2Cl_2 (0.1 M) at 0 °C and then MsCl (2.5 equiv., 0.212 mmol, 16.4 μL) and Et_3N (3 equiv., 0.255 mmol, 35.5 μL) were sequentially added dropwise. The reaction mixture was stirred at 0 °C for 3 h and then quenched by the addition of water (1 mL). The aqueous layer was extracted with CH_2Cl_2 (3 x 10 ml), and the combined organic layers were dried over Na_2SO_4, filtered, and concentrated under reduced pressure to afford the dimesylate. The crude dimesylate was then dissolved in DMF (0.01 M), and to this solution were added $NsNH_2$ (3 equiv., 0.255 mmol, 51.5 mg), Cs_2CO_3 (6 equiv., 0.510 mmol, 166.2 mg), and tetrabutylammonium iodide (0.5 equiv., 0.0425 mmol, 15.7 mg). The reaction mixture was stirred at 60 °C for 10 h and then quenched by the addition of water (1 mL). The aqueous layer was extracted with EtOAc (3 x 10 mL), and the combined organic layers were washed with brine (3 x 10 mL) and saturated aqueous LiCl (3 x 10 mL), dried over Na_2SO_4, filtered, and concentrated under reduced pressure. The crude product was purified by silica gel column chromatography (0-50% EtOAc/hexanes gradient) to afford **10** (12.9 mg, 0.033 mmol, 39%, >20:1 *dr*) as a yellow solid. $R_f = 0.25$ (25% EtOAc/hexanes). ^1H NMR (500 MHz, $CDCl_3$) δ 8.13 (d, $J = 8.7$ Hz, 2H), 7.75 (d, $J = 8.7$ Hz, 2H), 7.21 – 7.15 (m, 2H), 6.95 (t, $J = 7.4$ Hz, 1H), 6.57 (d, $J = 7.8$ Hz, 1H), 4.66 (d, $J = 16.1$ Hz, 1H), 4.49 (d, $J = 16.1$ Hz, 1H), 4.15 – 4.02 (m, 1H), 3.73 – 3.60 (m, 1H), 2.28 (q, $J = 6.2$ Hz, 1H), 2.06 – 1.96 (m, 1H), 1.90 (ddd, $J = 10.0$, 6.2, 3.5 Hz, 1H), 1.52 – 1.39 (m, 2H), 1.38 – 1.27 (m, 1H), 0.99 (t, $J = 7.4$ Hz, 3H). ^{13}C NMR (126 MHz, $CDCl_3$) δ 152.0, 149.7, 146.1, 130.2, 129.3, 128.8, 128.6, 123.7, 122.3, 117.7, 56.3, 52.1, 44.9, 42.5, 26.2, 21.8, 12.2. HRMS-ESI (*m/z*) calculated for $C_{19}H_{21}N_3O_4S$ $[M+H]^+$ 388.1326; found, 388.1315.

10.3 Synthesis of 11 via ring-opening using $Sc(OTf)_3$ and $TMSN_3$[21]

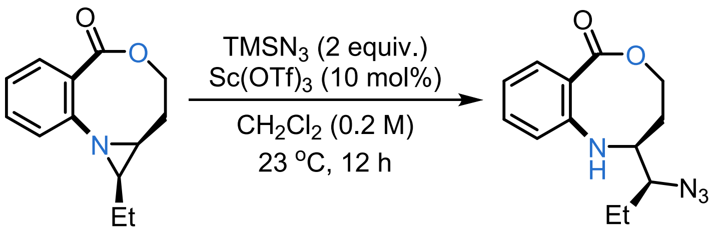

3a **11**
46% (>20:1 *dr*)

In a flame-dried two-neck round bottom flask equipped with a magnetic stir bar and under N_2 atmosphere, $Sc(OTf)_3$ (10 mol%, 0.01 mmol, 4.92 mg) was added to a solution of the oxazocine **3a** (1 equiv., 0.1 mmol, 21.7 mg) in CH_2Cl_2 (0.2 M). Then, $TMSN_3$ (2.0 equiv., 0.2 mmol, 26.3 μL) was added, and the reaction mixture was stirred at room temperature for 12 h. After this period, the solution was concentrated under reduced pressure and purified by silica gel column chromatography (0-25% EtOAc/hexanes gradient) to afford **11** (12.0 mg, 0.046 mmol, 46%, >20:1 *dr*) as a colorless semisolid. $R_f = 0.20$ (25% EtOAc/hexanes). ^1H NMR (500 MHz, $CDCl_3$) δ

7.44 (d, J = 7.9 Hz, 1H), 7.21 (td, J = 7.8, 1.2 Hz, 1H), 6.71 (t, J = 7.5 Hz, 1H), 6.58 (d, J = 8.3 Hz, 1H), 4.46 (td, J = 12.3, 2.7 Hz, 1H), 4.37 (dd, J = 12.2, 5.6 Hz, 1H), 4.22 (d, J = 8.4 Hz, 1H), 3.54 (ddt, J = 11.1, 8.3, 2.5 Hz, 1H), 3.48 (td, J = 7.1, 2.2 Hz, 1H), 2.13 (tdd, J = 13.0, 5.8, 2.6 Hz, 1H), 2.00 – 1.91 (m, 1H), 1.85 – 1.65 (m, 2H), 1.01 (t, J = 7.5 Hz, 3H). ^{13}C NMR (126 MHz, $CDCl_3$) δ 172.8, 146.3, 134.2, 132.5, 117.6, 116.6, 112.3, 67.6, 65.5, 50.7, 35.7, 26.0, 10.9. HRMS-ESI (m/z) calculated for $C_{13}H_{16}N_4O_2$ [M-H]$^-$ 259.1200; found, 259.1197.

10.4 Synthesis of 12 via ring-opening using NaI and Amberlyst-15[18]

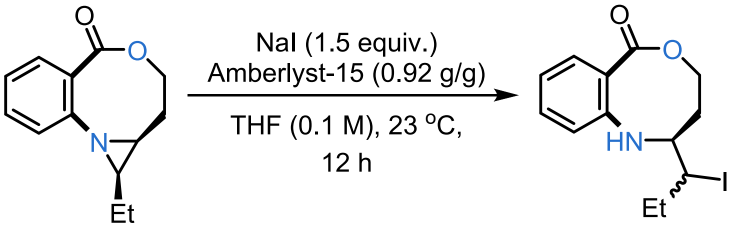

3a

12
35% (2:1 *dr*)

In a flame-dried two-neck round bottom flask equipped with a magnetic stir bar and under N_2 atmosphere, NaI (1.5 equiv., 0.15 mmol, 22.5 mg) was added to a solution of **3a** (1 equiv., 0.1 mmol, 21.7 mg) and Amberlyst-15 (0.92 g/g of **3a**, 20 mg) in THF (0.1 M). The reaction mixture was stirred at room temperature for 12 h, filtrated, and then concentrated under reduced pressure. The crude product was purified by silica gel column chromatography (0-20% EtOAc/hexanes gradient) to afford **12** (12.1 mg, 0.035 mmol, 35%, 2:1 *dr*) as a yellow solid. R_f = 0.20 (25% EtOAc/hexanes). *The mixture of diastereomers provided an almost fully overlapped ^{1}H NMR spectrum, except for two signals at around 6.7–6.6 ppm and 1.1–1.0 ppm, which allowed us to determine the diastereomeric ratio. The inherent complex pattern of overlapping signals prevented the identification of relevant coupling constants of the major and minor diastereomers. The data described below is from the apparent spectrum and does not specify the signals for each diastereomer. ^{1}H NMR (500 MHz, $CDCl_3$) δ 7.46 (d, J = 7.9 Hz, 1H), 7.25 – 7.20 (m, 1H), 6.76 – 6.70 (m, 1H), 6.65 (d, J = 8.3 Hz, 1H) (**major diastereomer**), 6.61 (d, J = 8.3 Hz, 1H) (**minor diastereomer**), 4.53 – 4.44 (m, 1H), 4.41 – 4.23 (m, 2H), 4.21 (ddd, J = 8.5, 5.3, 2.5 Hz, 1H), 2.93 – 2.82 (m, 1H), 2.26 (tdd, J = 12.7, 5.6, 2.7 Hz, 1H) (**minor diastereomer**), 2.20 – 2.05 (m, 2H), 1.91 – 1.77 (m, 1H), 1.09 (t, J = 7.2 Hz, 3H) (**minor diastereomer**), 1.02 (t, J = 7.2 Hz, 3H) (**major diastereomer**). ^{13}C NMR (126 MHz, $CDCl_3$) δ 172.7*, 145.8, 145.4, 134.2, 134.2, 132.5*, 117.9, 117.9, 116.7, 116.7, 112.8*, 65.2, 65.0, 52.2, 51.9, 48.3, 47.1, 39.3, 34.6, 32.9, 31.7, 14.8, 14.6. *peaks overlapped. HRMS-ESI (m/z) calculated for $C_{13}H_{16}INO_2$ [M+H]$^+$ 346.0299; found, 346.0287.

11. Crystal structure analysis of 3a

11.1 Data collection

A colorless crystal with approximate dimensions 0.16 × 0.11 × 0.04 mm^3 was selected under oil under ambient conditions and attached to the tip of a MiTeGen MicroMount©. The crystal was mounted in a stream of cold nitrogen at 100(1) K and centered in the X-ray beam by using a video camera. The crystal evaluation and data collection were performed on a Bruker D8 VENTURE PhotonIII four-circle diffractometer with Cu Kα (λ = 1.54178 Å) radiation and the detector to crystal distance of 50 mm and 70 mm.[19] The initial cell constants were obtained from a 180° φ scan conducted at a 2θ = 50° angle with the exposure time of 1 second per frame. The reflections were successfully indexed by an automated indexing routine built in the APEX3 program. The final cell constants were calculated from a set of 7705 strong reflections from the actual data collection. The data were collected by using the full sphere data collection routine to survey the reciprocal space to the extent of a full sphere to a resolution of 0.79 Å. A total of 9886 data were harvested by collecting 38 sets of frames with 0.6° scans in ω and φ with an exposure time 2–11 sec per frame. These highly redundant datasets were corrected for Lorentz and polarization effects. The absorption correction was based on fitting a function to the empirical transmission surface as sampled by multiple equivalent measurements.[20]

11.2 Structure Solution and Refinement

The systematic absences in the diffraction data were consistent for the space groups $P\bar{1}$ and $P1$. The E-statistics strongly suggested the centrosymmetric space group $P\bar{1}$ that yielded chemically reasonable and computationally stable results of refinement.[21–29] A successful solution by intrinsic phasing provided most non-hydrogen atoms from the E-map. The remaining non-hydrogen atoms were located with an alternating series of least-squares cycles and difference Fourier maps. The atomic structure factors were determined by DFT calculations, using the cc-pvTZ hybrid functional and the R2SCAN basis set, in the NoSpherA2 extension of the olex2.refine program.[26–28] All atoms were refined with anisotropic displacement coefficients. The compound crystallizes as a racemate. The enantiomer shown in Figure S6 (C8 – *R*, C11 – *S*) was chosen arbitrarily. All examined crystals were non-merohedrally twinned. The crystal chosen for the single-crystal X-ray diffraction experiment was a two-component non-merohedral twin with a 9.54(8) % second component contribution. The twin components are related by a 179.8° rotation about c*. The final least squares refinement of 281 parameters

against 2328 data resulted in residuals R (based on F^2 for $I \geq 2\sigma$) and wR (based on F^2 for all data) of 0.0216 and 0.0578, respectively. The final difference Fourier map was featureless.

Summary

Crystal Data for $C_{13}H_{15}NO_2$ (M =217.270 g/mol): triclinic, space group P-1 (no. 2), a = 8.3492(5) Å, b = 8.3954(5) Å, c = 9.4254(6) Å, α = 108.818(4)°, β = 112.424(4)°, γ = 97.373(5)°, V = 553.71(7) Å^3, Z = 2, T = 100 K, μ(Cu Kα) = 0.708 mm^{-1}, $Dcalc$ = 1.303 g/cm^3, 2328 reflections measured (11.1° ≤ 2Θ ≤ 155.68°), 2328 unique (R_{int} = ?, R_{sigma} = 0.0163) which were used in all calculations. The final R_1 was 0.0216 (I>=2u(I)) and wR_2 was 0.0578 (all data).

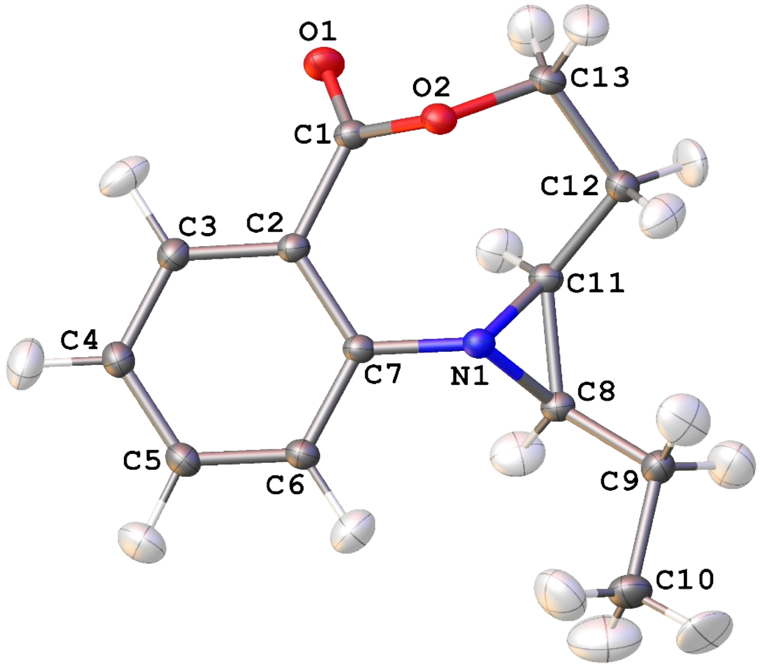

Figure S6. A molecular drawing of the asymmetric unit in **3a** shown with 50% probability ellipsoids. The compound crystallizes as a racemate. The enantiomer shown here (C8 – R, C11 – S) was chosen arbitrarily.

Table S2. Crystal data and structure refinement for **3a**.

Identification code	**3a**
Empirical formula	$C_{13}H_{15}NO_2$
Formula weight	217.270
Temperature/K	100
Crystal system	triclinic

Space group	P$\bar{1}$
a/Å	8.3492(5)
b/Å	8.3954(5)
c/Å	9.4254(6)
α/°	108.818(4)
β/°	112.424(4)
γ/°	97.373(5)
Volume/Å^3	553.71(7)
Z	2
ρ_{calc}g/cm^3	1.303
μ/mm^{-1}	0.708
F(000)	232.8
Crystal size/mm^3	0.16 × 0.11 × 0.04
Radiation	Cu Kα (λ = 1.54178)
2Θ range for data collection/°	11.1 to 155.68
Index ranges	-10 ≤ h ≤ 9, -10 ≤ k ≤ 10, 0 ≤ l ≤ 11
Reflections collected	2328
Independent reflections	2328 [R_{int} = ?, R_{sigma} = 0.0163]
Data/restraints/parameters	2328/0/281
Goodness-of-fit on F^2	1.277
Final R indexes [I>=2σ (I)]	R_1 = 0.0216, wR_2 = 0.0576
Final R indexes [all data]	R_1 = 0.0220, wR_2 = 0.0578
Largest diff. peak/hole / e Å^{-3}	0.10/-0.14

Table S3. Fractional Atomic Coordinates (×10^4) and Equivalent Isotropic Displacement Parameters (Å^2×10^3) for **3a**. U_{eq} is defined as 1/3 of the trace of the orthogonalised U_{IJ} tensor.

**Atom**	***x***	***Y***	***z***	**U(eq)**
O1	1809.8(7)	10579.0(7)	7371.7(6)	23.49(13)
O2	3719.2(6)	10003.6(6)	6234.1(6)	17.82(12)
N1	2754.6(7)	6485.9(7)	4684.9(7)	15.60(13)
C1	2749.7(9)	9688.5(9)	7034.9(8)	16.94(14)

Table S3. Fractional Atomic Coordinates (×10^4) and Equivalent Isotropic Displacement Parameters (Å^2×10^3) for **3a**. U_{eq} is defined as 1/3 of the trace of the orthogonalised U_{IJ} tensor.

**Atom**	***x***	***Y***	***z***	**U(eq)**
C2	2971.9(9)	8133.0(8)	7442.6(8)	15.99(14)
C3	3174.7(9)	8203.9(9)	9007.0(8)	18.37(15)
C4	3374.4(10)	6764.3(9)	9407.8(9)	20.09(15)
C5	3304.7(10)	5211.0(9)	8213.6(9)	20.13(15)
C6	3059.7(9)	5111.2(9)	6640.4(9)	18.24(15)
C7	2898.9(9)	6565.5(9)	6235.1(8)	15.29(14)
C8	1314.8(10)	5204.0(9)	3061.7(8)	17.25(14)
C9	1836.6(11)	4300.3(10)	1713.8(9)	21.20(15)
C10	2208.5(13)	2577.9(11)	1745.1(11)	27.50(17)
C11	1371.2(9)	7104.8(8)	3672.7(8)	16.64(14)
C12	2070.2(10)	8507.4(9)	3206.5(9)	18.73(15)
C13	2772.3(10)	10281.1(9)	4721.1(9)	19.38(15)

Table S4. Anisotropic Displacement Parameters (Å^2×10^3) for **3a**. The Anisotropic displacement factor exponent takes the form: $-2\pi^2[h^2a^{*2}U_{11}+2hka^*b^*U_{12}+\ldots]$.

**Atom**	$\mathbf{U_{11}}$	$\mathbf{U_{22}}$	$\mathbf{U_{33}}$	$\mathbf{U_{12}}$	$\mathbf{U_{13}}$	$\mathbf{U_{23}}$
O1	31.0(3)	20.4(2)	28.6(3)	14.3(2)	19.2(2)	11.7(2)
O2	20.1(2)	14.5(2)	20.6(2)	4.61(18)	10.60(19)	7.74(18)
N1	17.9(3)	13.6(3)	16.8(3)	5.3(2)	9.0(2)	6.2(2)
C1	20.1(3)	13.4(3)	18.6(3)	5.7(2)	10.1(2)	6.0(2)
C2	19.2(3)	13.0(3)	16.8(3)	4.6(2)	9.5(2)	5.7(2)
C3	22.1(3)	16.1(3)	17.0(3)	4.8(3)	9.8(3)	5.9(3)
H3	39(6)	23(5)	32(6)	7(5)	19(5)	-1(5)
C4	24.8(3)	19.5(3)	17.9(3)	6.1(3)	10.3(3)	9.1(3)
H4	48(7)	37(6)	22(6)	8(5)	18(5)	8(5)
C5	24.5(3)	18.2(3)	20.6(3)	7.0(3)	10.3(3)	10.7(3)
H5	55(7)	30(6)	39(6)	20(5)	22(6)	22(5)
C6	23.2(3)	14.6(3)	19.3(3)	6.8(3)	10.4(3)	8.2(3)

Table S4. Anisotropic Displacement Parameters (Å^2×10^3) for **3a**. The Anisotropic displacement factor exponent takes the form: $-2\pi^2[h^2a^{*2}U_{11}+2hka^*b^*U_{12}+\ldots]$.

**Atom**	**U_{11}**	**U_{22}**	**U_{33}**	**U_{12}**	**U_{13}**	**U_{23}**
H6	49(7)	28(6)	28(6)	11(5)	20(5)	5(5)
C7	17.9(3)	12.6(3)	16.9(3)	4.9(2)	9.0(2)	6.2(2)
C8	20.1(3)	13.8(3)	17.5(3)	4.9(3)	8.6(3)	5.8(2)
H8	22(5)	28(5)	42(6)	2(4)	14(5)	10(5)
C9	29.6(4)	18.6(3)	18.1(3)	10.4(3)	12.1(3)	7.7(3)
H9a	54(7)	40(7)	36(6)	27(6)	19(6)	20(5)
H9b	33(6)	44(7)	52(7)	20(5)	31(6)	24(6)
C10	37.7(5)	19.7(4)	31.4(4)	14.0(3)	19.7(4)	10.8(3)
H10a	108(11)	37(7)	60(8)	45(7)	63(8)	18(6)
H10b	52(8)	47(7)	48(7)	27(6)	24(6)	31(6)
H10c	48(8)	31(6)	74(9)	6(6)	26(7)	14(6)
C11	18.5(3)	14.3(3)	18.4(3)	5.7(2)	9.1(3)	6.9(2)
H11	21(5)	31(6)	35(6)	12(4)	19(5)	14(5)
C12	23.7(3)	16.3(3)	19.3(3)	7.0(3)	11.1(3)	8.6(3)
H12a	31(6)	27(6)	33(6)	2(5)	21(5)	6(5)
H12b	52(7)	37(6)	17(5)	17(5)	5(5)	16(5)
C13	25.1(4)	13.9(3)	22.1(3)	6.3(3)	12.0(3)	8.9(3)
H13a	35(6)	43(6)	36(6)	21(5)	19(5)	19(5)
H13b	47(7)	22(5)	30(6)	0(5)	15(5)	9(4)

Table S5. Bond Lengths for **3a**.

**Atom**	**Atom**	**Length/Å**	**Atom**	**Atom**	**Length/Å**
O1	C1	1.2096(9)	C3	C4	1.3891(9)
O2	C1	1.3546(8)	C4	C5	1.3980(10)
O2	C13	1.4541(8)	C5	C6	1.3898(10)
N1	C7	1.3980(8)	C6	C7	1.3995(9)
N1	C8	1.4651(8)	C8	C9	1.5049(10)
N1	C11	1.4654(8)	C8	C11	1.4992(9)

Table S5. Bond Lengths for **3a**.

**Atom**	**Atom**	**Length/Å**	**Atom**	**Atom**	**Length/Å**
C1	C2	1.4896(9)	C9	C10	1.5245(10)
C2	C3	1.3988(9)	C11	C12	1.5077(9)
C2	C7	1.4111(9)	C12	C13	1.5379(9)

Table S6. Bond Angles for **3a**.

**Atom**	**Atom**	**Atom**	**Angle/°**	**Atom**	**Atom**	**Atom**	**Angle/°**
C13	O2	C1	116.92(5)	C7	C6	C5	120.60(6)
C8	N1	C7	123.98(6)	C2	C7	N1	120.67(6)
C11	N1	C7	120.29(5)	C6	C7	N1	120.24(6)
C11	N1	C8	61.54(4)	C6	C7	C2	119.02(6)
O2	C1	O1	123.20(6)	C9	C8	N1	118.03(6)
C2	C1	O1	124.31(6)	C11	C8	N1	59.24(4)
C2	C1	O2	112.50(5)	C11	C8	C9	124.70(6)
C3	C2	C1	119.68(6)	C10	C9	C8	112.18(6)
C7	C2	C1	120.42(6)	C8	C11	N1	59.22(4)
C7	C2	C3	119.84(6)	C12	C11	N1	115.39(6)
C4	C3	C2	120.55(6)	C12	C11	C8	128.72(6)
C5	C4	C3	119.63(6)	C13	C12	C11	108.03(6)
C6	C5	C4	120.31(6)	C12	C13	O2	108.02(5)

Table S7. Torsion Angles for **3a**.

**A**	**B**	**C**	**D**	**Angle/°**	**A**	**B**	**C**	**D**	**Angle/°**
O1	C1	O2	C13	-48.70(8)	N1	C8	C11	C12	99.33(5)
O1	C1	C2	C3	-42.10(8)	N1	C11	C8	C9	-104.65(5)
O1	C1	C2	C7	135.09(7)	N1	C11	C12	C13	-80.44(6)
O2	C1	C2	C3	138.04(6)	C1	C2	C3	C4	179.68(6)
O2	C1	C2	C7	-44.77(7)	C1	C2	C7	C6	-178.15(6)

Table S7. Torsion Angles for **3a**.

A	B	C	D	Angle/°	A	B	C	D	Angle/°
O2	C13	C12	C11	40.34(6)	C2	C3	C4	C5	-2.38(8)
N1	C7	C2	C1	4.86(7)	C2	C7	C6	C5	-0.60(8)
N1	C7	C2	C3	-177.96(6)	C3	C4	C5	C6	0.80(8)
N1	C7	C6	C5	176.41(6)	C4	C5	C6	C7	0.68(8)
N1	C8	C9	C10	91.66(7)	C8	C11	C12	C13	-150.23(8)

Table S8. Hydrogen Atom Coordinates ($Å\times10^4$) and Isotropic Displacement Parameters ($Å^2\times10^3$) for **3a**.

**Atom**	***x***	***y***	***z***	**U(eq)**
H3	3219(14)	9413(12)	9922(12)	34(2)
H4	3575(15)	6841(13)	10637(12)	37(3)
H5	3401(16)	4058(13)	8482(13)	38(3)
H6	3017(15)	3918(13)	5718(13)	36(3)
H8	265(13)	4359(12)	3129(12)	32(2)
H9a	747(16)	4052(14)	489(13)	41(3)
H9b	3042(14)	5190(14)	1883(14)	37(3)
H10a	2590(20)	1969(15)	755(16)	58(4)
H10b	3347(16)	2814(14)	2932(15)	44(3)
H10c	1038(17)	1697(15)	1588(17)	54(3)
H11	333(12)	7372(12)	4081(12)	27(2)
H12a	3172(13)	8227(12)	2892(12)	31(2)
H12b	1020(15)	8576(13)	2130(11)	37(3)
H13a	1646(14)	10806(14)	4799(13)	35(2)
H13b	3772(15)	11224(12)	4692(12)	35(2)

12. Computational methods (Scheme 3)

All the calculations reported in this paper were performed with the Gaussian 16 suite of programs.[30] Electron correlation was partially taken into account using the hybrid functional usually denoted as B3LYP[31] in

conjunction with the D3 dispersion correction suggested by Grimme et al.[32] using the standard double-ζ quality def2-SVP[33] basis set for all atoms. The SMD continuum model was used to model the effects of the solvent. This level is denoted SMD(THF)-B3LYP-D3/def2-SVP. This DFT level has been selected to enable a direct comparison with our previous reports on the chemistry of strongly related systems.[14c–d,34] Geometries were fully optimized in solution without any geometry or symmetry constraints. Reactants, intermediates, and products were characterized by frequency calculations,[35] and have positive definite Hessian matrices. Transition structures (TS's) show only one negative eigenvalue in their diagonalized force constant matrices, and their associated eigenvectors were confirmed to correspond to the motion along the reaction coordinate under consideration using the Intrinsic Reaction Coordinate (IRC) method.[36] Energy refinements were performed through single-point calculations at the same DFT level but with the much larger triple-ζ basis set def2-TZVPP. The computed thermochemistry data were corrected following Grimme's quasi-harmonic (QHA) model for entropy[37] with a frequency cutoff value of 100.0 cm^{-1} and corrected to a 1 M concentration using the GoodVibes[38] program at 298.15 K. This level is denoted as SMD(THF)-B3LYP-D3/def2-TZVPP//SMD(THF)-B3LYP-D3/def2-SVP.

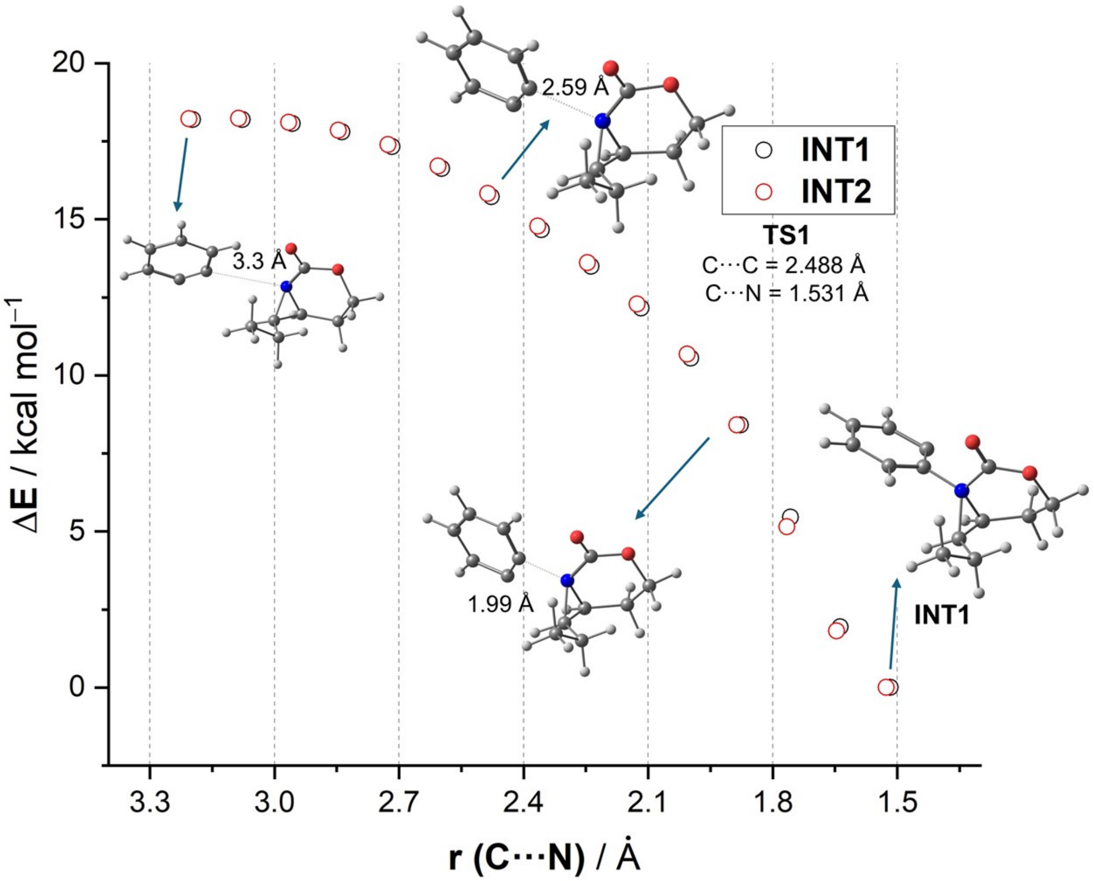

Figure S7. Relaxed scans calculations for the formation of **INT1** and **INT2** from **1a** and benzyne. Data computed at the SMD(THF)-B3LYP-D3/def2-SVP level.

Cartesian coordinates (in Å) and energies (in free energies, in a.u.) of all the stationary points discussed in the text. All calculations have been performed at the SMD(THF)-B3LYP-D3/def2-TZVPP//SMD(THF)-B3LYP-D3/def2-SVP level.

benzyne, G = -230.963167

C	0.626850000	-1.239993000	0.000037000
C	1.467172000	-0.133919000	-0.000085000
C	0.705432000	1.058998000	0.000030000
C	-0.704920000	1.059284000	0.000026000
C	-1.467150000	-0.133272000	-0.000074000
C	-0.627548000	-1.240032000	0.000061000
H	2.559276000	-0.130550000	-0.000013000
H	1.235609000	2.016508000	0.000069000
H	-1.234643000	2.017048000	0.000048000
H	-2.559260000	-0.129398000	-0.000073000

1a, G = -478.613426

N	0.037742000	0.134679000	-1.130946000
C	0.548008000	-1.212163000	-0.900344000
C	1.589893000	-1.401765000	0.188527000
C	1.760785000	-0.156874000	1.062016000
O	1.585242000	1.074797000	0.328550000
C	0.414318000	1.218717000	-0.343462000
C	-0.908555000	-0.909391000	-0.706133000
O	-0.192406000	2.262861000	-0.315331000
C	-2.813767000	0.106401000	0.599437000
C	-1.612388000	-0.842062000	0.630370000
H	0.793244000	-1.734722000	-1.833199000
H	1.355645000	-2.268090000	0.828331000
H	2.545238000	-1.620668000	-0.311940000

H	1.054138000	-0.152417000	1.907990000
H	2.774321000	-0.108330000	1.481423000
H	-1.571005000	-1.227535000	-1.522219000
H	-3.328153000	0.127032000	1.573369000
H	-2.495701000	1.132137000	0.356057000
H	-3.547146000	-0.208760000	-0.161386000
H	-1.942227000	-1.864510000	0.885381000
H	-0.915001000	-0.535333000	1.424593000

INT1, G = -709.58125

N	-0.230242000	-0.165535000	-0.100190000
C	-0.913938000	-0.860817000	-1.241440000
C	-1.972328000	-1.859533000	-0.866117000
C	-2.803624000	-1.433144000	0.322429000
O	-1.999336000	-0.842134000	1.378025000
C	-0.855295000	-0.199303000	1.246122000
C	-0.960732000	0.619212000	-1.195738000
O	-0.308037000	0.335047000	2.162836000
C	-1.578688000	2.892005000	-0.302278000
C	-2.090879000	1.558325000	-0.851621000
C	1.286897000	-0.185180000	-0.087898000
C	1.899827000	1.045969000	0.179262000
C	3.294803000	1.077461000	0.184994000
C	4.000004000	-0.109742000	-0.068989000
C	3.307374000	-1.302343000	-0.324027000
C	1.890324000	-1.407483000	-0.350087000
H	-0.148822000	-1.275448000	-1.903439000
H	-2.633359000	-2.041201000	-1.727520000
H	-1.445712000	-2.800367000	-0.643132000
H	-3.573244000	-0.697500000	0.051679000
H	-3.302462000	-2.288452000	0.795342000
H	-0.199851000	1.090331000	-1.826730000

H	-2.416996000	3.584406000	-0.131064000
H	-1.057877000	2.753521000	0.658590000
H	-0.878166000	3.372286000	-1.003881000
H	-2.646127000	1.718542000	-1.791506000
H	-2.806213000	1.116091000	-0.146808000
H	1.315485000	1.949519000	0.373799000
H	3.824550000	2.013378000	0.382924000
H	5.095973000	-0.095456000	-0.068609000
H	3.921039000	-2.196774000	-0.512878000

INT2, G = -709.580197

N	-0.231869000	-0.078170000	-0.134671000
C	-0.886867000	-0.350149000	-1.471395000
C	-1.900017000	-1.461693000	-1.491764000
C	-2.740438000	-1.519298000	-0.234646000
O	-1.954520000	-1.324771000	0.971132000
C	-0.871496000	-0.579468000	1.100402000
C	-0.986886000	1.006440000	-0.892889000
O	-0.389345000	-0.321867000	2.161910000
C	-1.662570000	2.799582000	0.731218000
C	-2.145670000	1.730685000	-0.252593000
C	1.291315000	0.010277000	-0.099989000
C	1.885252000	1.184131000	-0.531077000
C	3.301960000	1.105176000	-0.504008000
C	4.012447000	-0.032201000	-0.094971000
C	3.321305000	-1.184423000	0.311194000
C	1.926363000	-1.171691000	0.306400000
H	-0.112188000	-0.443252000	-2.239152000
H	-2.559861000	-1.342097000	-2.365046000
H	-1.348974000	-2.406266000	-1.624373000
H	-3.534387000	-0.759882000	-0.230047000
H	-3.210282000	-2.502486000	-0.107695000
H	-0.191772000	1.677042000	-1.257852000

H	-2.517518000	3.346187000	1.157934000
H	-1.097774000	2.348132000	1.561975000
H	-0.999184000	3.525946000	0.236848000
H	-2.704421000	2.197535000	-1.081471000
H	-2.852842000	1.055353000	0.247515000
H	3.900006000	1.974354000	-0.817886000
H	5.108244000	-0.031407000	-0.094242000
H	3.861677000	-2.081726000	0.624989000
H	1.365099000	-2.061344000	0.611557000

TSrot, G = -709.573639 (i = -52 cm-1)

N	-0.235768000	-0.150315000	-0.024027000
C	-0.866752000	-1.099636000	-1.009413000
C	-1.888526000	-2.060402000	-0.472701000
C	-2.739482000	-1.478778000	0.633503000
O	-1.958069000	-0.671594000	1.553015000
C	-0.974818000	0.145173000	1.219047000
C	-0.950562000	0.342858000	-1.298203000
O	-0.671405000	1.079101000	1.902663000
C	-1.647461000	2.743731000	-1.011594000
C	-2.113636000	1.298241000	-1.195562000
C	1.329378000	-0.135057000	-0.018987000
C	1.996559000	0.576379000	0.987093000
C	3.395633000	0.559590000	0.960758000
C	4.065361000	-0.132341000	-0.055224000
C	3.325624000	-0.797742000	-1.043600000
C	1.913007000	-0.829324000	-1.064324000
H	-0.012567000	-1.556287000	-1.542826000
H	-2.536341000	-2.403917000	-1.293943000
H	-1.338179000	-2.939045000	-0.101566000
H	-3.547655000	-0.839128000	0.249604000
H	-3.190212000	-2.259760000	1.258259000
H	-0.170660000	0.669584000	-1.998192000

H	-2.508218000	3.428449000	-0.969609000
H	-1.075124000	2.857657000	-0.077778000
H	-1.000259000	3.059650000	-1.845393000
H	-2.676113000	1.190600000	-2.138912000
H	-2.813044000	1.018756000	-0.394389000
H	1.479964000	1.132446000	1.765685000
H	3.953297000	1.095282000	1.734162000
H	5.160742000	-0.143436000	-0.076097000
H	3.894577000	-1.314855000	-1.830995000

TS1, G = -709.57115 (i = -131 cm-1)

N	-0.214583000	0.058930000	-0.210924000
C	-0.900938000	-0.194752000	-1.523737000
C	-1.798690000	-1.406282000	-1.598114000
C	-2.357049000	-1.844769000	-0.253591000
O	-1.356324000	-1.817391000	0.790806000
C	-0.683962000	-0.691416000	1.037823000
C	-1.121768000	1.096192000	-0.818359000
O	-0.660026000	-0.126440000	2.092064000
C	-2.040567000	2.663795000	0.927824000
C	-2.357141000	1.562327000	-0.084528000
C	1.263590000	0.094505000	-0.146981000
C	2.009374000	1.105842000	-0.754452000
C	3.402532000	1.009880000	-0.641004000
C	3.966782000	-0.064548000	0.060519000
C	3.145596000	-1.045033000	0.645155000
C	1.736968000	-1.007091000	0.560920000
H	-0.178160000	-0.134941000	-2.342236000
H	-2.630273000	-1.211829000	-2.293984000
H	-1.197264000	-2.223384000	-2.023927000
H	-3.209748000	-1.225111000	0.065716000
H	-2.699141000	-2.885943000	-0.297376000

H	-0.532474000	1.924767000	-1.221490000
H	-2.960934000	3.016394000	1.418406000
H	-1.357623000	2.291891000	1.706117000
H	-1.564907000	3.529107000	0.437822000
H	-3.049249000	1.937358000	-0.857840000
H	-2.874676000	0.729559000	0.410452000
H	1.556814000	1.940850000	-1.297137000
H	4.039877000	1.774326000	-1.093700000
H	5.056553000	-0.132909000	0.151161000
H	3.645734000	-1.863888000	1.182672000

3a, G = -709.686551

N	-0.834901000	-0.157903000	-0.093196000
C	-1.219902000	0.950893000	0.753700000
C	-1.527857000	2.255020000	0.047511000
C	-0.201091000	2.932766000	-0.326787000
O	0.668179000	1.929286000	-0.884846000
C	1.628649000	1.388559000	-0.095184000
C	-2.005403000	-0.335864000	0.736566000
O	2.415480000	2.057572000	0.535153000
C	-3.502545000	-1.958879000	-0.480050000
C	-3.339722000	-0.534106000	0.052189000
C	0.380580000	-0.824025000	-0.054992000
C	0.425205000	-2.231406000	-0.002802000
C	1.645944000	-2.903776000	0.005333000
C	2.855712000	-2.194351000	-0.022271000
C	2.824893000	-0.801587000	-0.059235000
C	1.607789000	-0.103222000	-0.100812000
H	-0.603618000	1.078486000	1.657857000
H	-2.115274000	2.056087000	-0.860631000
H	-2.112027000	2.932692000	0.691913000
H	-0.342740000	3.715359000	-1.086107000

H	0.286705000	3.390034000	0.549229000
H	-1.861856000	-0.961755000	1.631121000
H	-4.483473000	-2.096544000	-0.963103000
H	-2.724582000	-2.188227000	-1.226888000
H	-3.422510000	-2.703314000	0.330362000
H	-4.140368000	-0.306899000	0.777836000
H	-3.441766000	0.187111000	-0.773824000
H	-0.515360000	-2.785382000	0.035878000
H	1.654799000	-3.996653000	0.043902000
H	3.810219000	-2.725402000	-0.009240000
H	3.753363000	-0.225280000	-0.067374000

TS2, G = -709.568245 (i = -460 cm-1)

N	-0.168522000	-0.332989000	-0.121977000
C	-0.908164000	-0.107171000	-1.395253000
C	-2.002489000	-1.127181000	-1.619799000
C	-2.845804000	-1.356912000	-0.382710000
O	-2.068578000	-1.356016000	0.835480000
C	-0.807541000	-0.929816000	0.992042000
C	-1.080496000	1.253215000	-0.857397000
O	-0.253360000	-1.059519000	2.052731000
C	-1.577301000	2.805354000	1.056345000
C	-2.124625000	1.799115000	0.039506000
C	1.277931000	-0.120380000	-0.102977000
C	1.766720000	1.158796000	-0.381972000
C	3.173228000	1.257115000	-0.406716000
C	4.019551000	0.167299000	-0.149023000
C	3.470066000	-1.090866000	0.130185000
C	2.082242000	-1.246047000	0.146129000
H	-0.183354000	-0.118615000	-2.217947000
H	-2.645125000	-0.814087000	-2.456509000
H	-1.502074000	-2.061753000	-1.918038000

H	-3.625706000	-0.589790000	-0.269150000
H	-3.345470000	-2.335206000	-0.417268000
H	-0.261502000	1.932614000	-1.123778000
H	-2.400735000	3.251045000	1.634751000
H	-0.888322000	2.306652000	1.755640000
H	-1.023503000	3.616286000	0.558757000
H	-2.819628000	2.316316000	-0.657456000
H	-2.723556000	1.030056000	0.542358000
H	3.649113000	2.225171000	-0.621054000
H	5.107534000	0.294949000	-0.161408000
H	4.116400000	-1.949724000	0.331513000
H	1.641166000	-2.223839000	0.357582000

TS3, G = -709.565542 (i = -1503 cm-1)

N	-0.257383000	-0.192979000	-0.111065000
C	-0.913843000	-0.175126000	-1.483259000
C	-2.069679000	-1.131045000	-1.650062000
C	-2.944339000	-1.188402000	-0.417837000
O	-2.171906000	-1.269830000	0.808634000
C	-0.960378000	-0.778218000	1.021482000
C	-0.824792000	1.098876000	-0.759752000
O	-0.448383000	-0.803708000	2.104837000
C	-1.261966000	2.779568000	1.052286000
C	-1.880432000	1.880977000	-0.024085000
C	1.248185000	-0.170518000	-0.067089000
C	1.781762000	0.991793000	-0.595123000
C	3.185310000	1.051237000	-0.640802000
C	3.972836000	-0.014873000	-0.178320000
C	3.369207000	-1.173306000	0.333724000
C	1.974527000	-1.268883000	0.390395000
H	-0.135027000	-0.319831000	-2.247124000
H	-2.678883000	-0.816611000	-2.512349000

H	-1.664113000	-2.129310000	-1.883087000
H	-3.595041000	-0.307276000	-0.335442000
H	-3.579165000	-2.083751000	-0.408981000
H	0.491081000	1.563361000	-0.901440000
H	-2.036712000	3.372171000	1.564780000
H	-0.741708000	2.177801000	1.815472000
H	-0.526000000	3.475141000	0.619541000
H	-2.382958000	2.501480000	-0.786796000
H	-2.679281000	1.274204000	0.437528000
H	3.693674000	1.937634000	-1.041106000
H	5.065077000	0.049810000	-0.218000000
H	3.984486000	-2.004315000	0.689139000
H	1.490170000	-2.163828000	0.788209000

TS4, G = -709.567243 (i = -254 cm-1)

N	-0.064989000	-0.149037000	0.177159000
C	-0.744067000	-1.087787000	-0.760771000
C	-1.642901000	-2.076336000	-0.058143000
C	-2.538801000	-1.416799000	0.985173000
O	-2.027962000	-0.147129000	1.456975000
C	-0.775109000	0.306315000	1.315637000
C	-1.187731000	0.111505000	-1.447500000
O	-0.327116000	1.096191000	2.106799000
C	-2.496099000	2.258382000	-1.264548000
C	-2.518362000	0.744739000	-1.517126000
C	1.399372000	-0.066939000	0.029850000
C	1.975542000	-1.054500000	-0.772501000
C	3.376745000	-1.002746000	-0.884766000
C	4.140204000	0.027011000	-0.313805000
C	3.505197000	1.022256000	0.441823000
C	2.121728000	0.985482000	0.627457000
H	0.149657000	-1.579332000	-1.246708000

H	-2.255147000	-2.617755000	-0.794850000
H	-0.977598000	-2.811286000	0.419790000
H	-3.539836000	-1.194603000	0.592014000
H	-2.663580000	-2.056861000	1.871691000
H	-0.394472000	0.607307000	-2.020591000
H	-3.492008000	2.688381000	-1.447416000
H	-2.216078000	2.476561000	-0.222298000
H	-1.774836000	2.763403000	-1.926076000
H	-2.824353000	0.565443000	-2.571875000
H	-3.263470000	0.239749000	-0.891245000
H	3.911634000	-1.768614000	-1.464967000
H	5.224261000	0.066532000	-0.467079000
H	4.087263000	1.830004000	0.894693000
H	1.629807000	1.738333000	1.239926000

4a, G = -709.739041

N	0.727085000	-0.655906000	0.269070000
C	0.919228000	0.604404000	1.040045000
C	2.330893000	1.168196000	0.851985000
C	2.970759000	0.568174000	-0.385535000
O	2.936070000	-0.870041000	-0.311102000
C	1.719973000	-1.459693000	-0.229517000
C	-0.326831000	1.437740000	0.637814000
O	1.565914000	-2.606752000	-0.592758000
C	-1.363484000	3.130327000	-1.003353000
C	-0.141109000	2.291366000	-0.634552000
C	-0.660162000	-0.882557000	0.159026000
C	-1.340163000	0.321379000	0.434179000
C	-2.731035000	0.349993000	0.415430000
C	-3.438523000	-0.825108000	0.109651000
C	-2.748666000	-2.006952000	-0.180248000
C	-1.346345000	-2.057021000	-0.156037000

H	0.786511000	0.337485000	2.102874000
H	2.316864000	2.265803000	0.781352000
H	2.949167000	0.906682000	1.723941000
H	2.457360000	0.893247000	-1.306583000
H	4.032478000	0.836613000	-0.467777000
H	-0.609706000	2.100023000	1.471321000
H	-1.149168000	3.772485000	-1.872979000
H	-2.228907000	2.501422000	-1.264300000
H	-1.664683000	3.787201000	-0.169383000
H	0.719147000	2.963725000	-0.480813000
H	0.124990000	1.629511000	-1.476799000
H	-3.267019000	1.275908000	0.637819000
H	-4.531459000	-0.814884000	0.100103000
H	-3.307745000	-2.916144000	-0.418075000
H	-0.800501000	-2.974866000	-0.366632000

6a, G = -709.72441

N	0.081534000	0.704691000	-0.058269000
C	1.222749000	-0.120857000	-0.289991000
C	2.465814000	0.679724000	-0.564956000
C	2.496597000	1.876977000	0.362367000
O	1.321670000	2.677582000	0.167964000
C	0.113691000	2.091093000	0.070264000
C	1.144890000	-1.465828000	-0.270684000
O	-0.887931000	2.770478000	0.094143000
C	2.712210000	-3.172975000	0.747687000
C	2.272284000	-2.429624000	-0.522082000
C	-1.226211000	0.104698000	-0.021326000
C	-1.884811000	-0.198971000	-1.217371000
C	-3.145265000	-0.800555000	-1.180289000
C	-3.746190000	-1.096130000	0.049054000
C	-3.083182000	-0.790266000	1.242487000

C	-1.820168000	-0.191339000	1.208993000
H	-1.403297000	0.039248000	-2.168475000
H	3.369238000	0.074301000	-0.420863000
H	2.463558000	1.039020000	-1.609774000
H	2.549455000	1.562077000	1.419451000
H	3.347661000	2.538863000	0.152522000
H	0.180227000	-1.925858000	-0.037863000
H	3.493689000	-3.918045000	0.523206000
H	3.115571000	-2.471736000	1.497238000
H	1.865061000	-3.704357000	1.213320000
H	1.936935000	-3.174070000	-1.267243000
H	3.140830000	-1.924568000	-0.973099000
H	-3.662602000	-1.034932000	-2.114270000
H	-4.733274000	-1.564970000	0.076341000
H	-3.550257000	-1.018955000	2.203940000
H	-1.287881000	0.050982000	2.131679000

INT3, G = -709.60077

N	-0.223037000	-0.120174000	-0.116772000
C	-0.936030000	-0.559512000	-1.378065000
C	-1.922087000	-1.692949000	-1.164628000
C	-2.759615000	-1.508371000	0.079330000
O	-1.977296000	-1.050874000	1.211723000
C	-0.794377000	-0.450895000	1.175308000
C	-1.028712000	0.887049000	-1.133501000
O	-0.209309000	-0.162311000	2.184552000
C	-1.826051000	2.918308000	0.138817000
C	-2.214259000	1.588136000	-0.516576000
C	1.231681000	-0.055795000	-0.111870000
C	1.879225000	1.178240000	-0.083779000
C	3.276150000	1.213033000	-0.074933000
C	4.012181000	0.022306000	-0.103766000

C	3.349949000	-1.209638000	-0.133584000
C	1.952555000	-1.252028000	-0.134433000
H	-0.185460000	-0.855167000	-2.127058000
H	-2.586635000	-1.756193000	-2.040796000
H	-1.359504000	-2.640924000	-1.115878000
H	-3.563809000	-0.776836000	-0.075147000
H	-3.215453000	-2.450934000	0.412129000
H	1.284694000	2.092432000	-0.079248000
H	-2.712661000	3.457918000	0.513120000
H	-1.156584000	2.745625000	0.998632000
H	-1.294346000	3.571653000	-0.571523000
H	-2.888233000	1.784569000	-1.372131000
H	-2.829351000	1.024041000	0.211706000
H	3.792073000	2.176140000	-0.048396000
H	5.104785000	0.055120000	-0.103731000
H	3.920482000	-2.141494000	-0.156325000
H	1.420429000	-2.206558000	-0.148061000

TS5, G = -709.575555 (i = -764 cm-1)

N	-0.279498000	-0.004574000	-0.021591000
C	-1.035736000	-0.277512000	-1.691176000
C	-1.750320000	-1.582126000	-1.430403000
C	-2.666931000	-1.524193000	-0.227679000
O	-2.004321000	-1.245167000	1.027939000
C	-0.862483000	-0.588664000	1.176457000
C	-1.043618000	0.913548000	-0.876689000
O	-0.352536000	-0.451093000	2.256579000
C	-1.571988000	2.942683000	0.535135000
C	-2.123625000	1.770039000	-0.283761000
C	1.167045000	-0.010281000	-0.048646000
C	1.875099000	1.161843000	-0.326343000
C	3.272041000	1.128009000	-0.381158000

C	3.961297000	-0.066720000	-0.150777000
C	3.245627000	-1.235386000	0.137404000
C	1.850953000	-1.211147000	0.187059000
H	-0.462403000	-0.306056000	-2.639690000
H	-2.391733000	-1.768183000	-2.312318000
H	-1.050935000	-2.438931000	-1.386803000
H	-3.436546000	-0.751253000	-0.370080000
H	-3.170755000	-2.486517000	-0.064601000
H	1.320177000	2.083211000	-0.504063000
H	-2.384084000	3.532929000	0.992891000
H	-0.928000000	2.577574000	1.353022000
H	-0.963956000	3.615031000	-0.091931000
H	-2.733419000	2.151236000	-1.121680000
H	-2.836768000	1.212545000	0.367306000
H	3.823743000	2.046766000	-0.597210000
H	5.053361000	-0.088289000	-0.191651000
H	3.775946000	-2.173952000	0.318307000
H	1.292565000	-2.124561000	0.406954000

5a, G = -709.711847

N	-0.392058000	-0.235960000	0.230790000
C	-2.085184000	1.358082000	-0.387009000
C	-2.771909000	0.323214000	-1.232827000
C	-3.361328000	-0.788322000	-0.362084000
O	-2.442362000	-1.209646000	0.661030000
C	-1.131279000	-1.403389000	0.360561000
C	-0.961510000	1.079685000	0.298115000
O	-0.647284000	-2.511157000	0.298168000
C	0.691948000	3.056407000	0.431794000
C	-0.243552000	2.090811000	1.172134000
C	1.019771000	-0.351581000	-0.008777000
C	1.549486000	0.111735000	-1.219035000

C	2.925264000	0.032721000	-1.454126000
C	3.771602000	-0.515180000	-0.484452000
C	3.237885000	-0.982594000	0.722690000
C	1.864666000	-0.898904000	0.964153000
H	-2.542479000	2.343775000	-0.263057000
H	-3.578295000	0.760600000	-1.840544000
H	-2.045670000	-0.126697000	-1.934278000
H	-4.247180000	-0.433809000	0.184807000
H	-3.654540000	-1.660188000	-0.974118000
H	0.879092000	0.541073000	-1.967053000
H	1.108203000	3.800406000	1.130689000
H	1.534513000	2.530149000	-0.040011000
H	0.148447000	3.600864000	-0.357658000
H	-1.012681000	2.681974000	1.695460000
H	0.319392000	1.551769000	1.950950000
H	3.335322000	0.398322000	-2.399111000
H	4.847185000	-0.578334000	-0.668749000
H	3.895925000	-1.410289000	1.483556000
H	1.439173000	-1.257573000	1.903185000

13. References

1 W. L. F. Armarego, C. L. L. Chai, *Purification of Laboratory Chemicals*, 6th ed., Elsevier, Oxford, 2009.

2 H. E. Gottlieb, V. Kotlyar, and A. Nudelman, *J. Org. Chem.*, 1997, **62**, 7512-7515.

3 T. Matsumoto, T. Hosoya, M. Katsuki, and K. Suzuki, *Tetrahedron Lett.*, 1991, **32**, 6735–6736.

4 S. Kovácsa, Á. Csincsia, T. Z. Nagya, S. Boros, G. Timári, and Z. Novák, *Org. Lett.*, 2012, **14**, 2022–2025.

5 M. Wang, and Z. Huang, *Org. Biomol. Chem.*, 2016, **14**, 10185–10188.

6 H. Jiang, Y. Zhang, W. Xiong, J. Cen, L. Wang, R. Cheng, C. Qi, and W. Wu, *Org. Lett.*, 2019, **21**, 345–349.

7 J. George, J. S. Ward, and M. S. Sherburn, *Org. Lett.* 2019, 21, **18**, 7529–7533.

8 L. Wisson, G. Hanquet, F. Toulgoat, T. Billard, A. Panossian, and F. R. Leroux, *Eur. J. Org. Chem.*, 2024, **27**, e202400388.

9 T. K. Shah, J. M. Medina, and N. K. Garg, *J. Am. Chem. Soc.*, 2016, **138**, 4948–4954.

10 M. A. Walters and J. J. Shay, *Synth. Commun.*, 1997, **27**, 3573–3579.

11 Y. Jiang, W. Zhu, J. Huang, F. Luo, X. Chen, C. Fang, X. Chen, S. Liu, Y. Hu and S. Zhang, *Org. Chem. Front.*, 2024, **11**, 12–20.

12 K. Inoue, R. Nakura, K. Okano, and A. Mori, *Eur. J. Org. Chem.*, 2018, **2018**, 3343–3347.

13 M. Ramirez, E. R. Darzi, J. S. Donaldson, K. N. Houk, and N. K. Garg, *Angew. Chem. Int. Ed.*, 2021, **60**, 18201–18208.

14 (a) J. W. Rigoli, C. D. Weatherly, J. M. Alderson, B. T. Vo, and J. M. Schomaker, *J. Am. Chem. Soc.*, 2013, **135**, 17238–17241; (b) R. M. Ward, Y. Hu, N. P. Tu, and J. M. Schomaker, *ChemSusChem*, 2024, **17**, e202300964; (c) K. A. Nicastri, S. A. Zappia, J, C. Pratt, J. M. Duncan, I. A. Guzei, I. Fernández, and J. M. Schomaker, *ACS Catal.*, 2022, **12**, 1572–1580; (d) J. Eshon, K. A. Nicastri, S. C. Schmid, W. T. Raskopf, I. A. Guzei, I. Fernández, and J. M. Schomaker, *Nat. Commun.*, 2020, **11**, 1273.

15 (a) T. A. Trinh, Y. Fu, D. B. Hu, S. A. Zappia, I. A. Guzei, P. Liu, and J. M. Schomaker, *Chem. Commun.*, 2024, **60**, 224–227; (b) Y.Ohta, S. Yasuda, Y. Yokogawa, K. Kurokawa, C. Mukai, *Angew. Chem. Int. Ed.*, 2014, **54**, 1240–1244.

16 C. J. Hayes, P. W. Beavis, and L. A. Humphries, *Chem. Commun.*, 2006, **43**, 4501–4502.

17 Y-C. Lin, C-T. Chen, *Org. Lett.*, 2009, **11**, 4858–4861.

18 M. Ju, C. D. Weatherly, I. A. Guzei, and J. M. Schomaker, *Angew. Chem. Int. Ed.*, 2017, **56**, 9944–9948.

19 Bruker-AXS (2019). *APEX3*. Version 2019.11-0. Madison, Wisconsin, USA.

20 L. Krause, R, Herbst-Irmer, G. M. Sheldrick, and D. Stalke, *J. Appl. Cryst.*, 2015, **48**, 3–10.

21 G. M. Sheldrick, (2013b). *XPREP*. Version 2013/1. Georg-August-Universität Göttingen, Göttingen, Germany.

22 G. M. Sheldrick, (2013a). The *SHELX* homepage, http://shelx.uni-ac.gwdg.de/SHELX/.

23 G. M. Sheldrick, *Acta Cryst. A*, 2015a, **71**, 3–8.

24 G. M. Sheldrick, *Acta Cryst. C*, 2015b, **71**, 3–8.

25 O. V. Dolomanov, L. J. Bourhis, R. J. Gildea, J. A. K. Howard, and H. Puschmann, *J. Appl. Crystallogr.*, 2009, **42**, 339–341.

26 F. Kleemiss, O. V. Dolomanov, M. Bodensteiner, N. Peyerimhoff, L. Midgley, L. J. Bourhis, A. Genoni, L. A. Malaspina, D. Jayatilaka, J. L. Spencer, F. White, B. Grundkötter-Stock, S. Steinhauer, D. Lentz, H. Puschmann, and S. Grabowsky, *Chem. Sci.*, 2021, **12**, 1675–1692.

27 L.J. Bourhis, O.V. Dolomanov, R.J. Gildea, J.A.K. Howard, and H. Puschmann, *Acta Cryst. A*, 2015, **71**, 59–75.

28 F. Neese, *Wiley Interdiscip. Rev.: Comput. Mol. Sci.*, 2018, **8**, e1327.

29 I. A. Guzei, 2007-2022. Programs Gn. University of Wisconsin-Madison, Madison, Wisconsin, USA.

30 Gaussian 16, Revision E.01, M. J. Frisch, G. W. Trucks, H. B. Schlegel, G. E. Scuseria, M. A. Robb, J. R. Cheeseman, G. Scalmani, V. Barone, B. Mennucci, G. A. Petersson, H. Nakatsuji, M. Caricato, X. Li, H. P. Hratchian, A. F. Izmaylov, J. Bloino, G. Zheng, J. L. Sonnenberg, M. Hada, M. Ehara, K. Toyota, R. Fukuda, J. Hasegawa, M. Ishida, T. Nakajima, Y. Honda, O. Kitao, H. Nakai, T. Vreven, J. A., Jr. Montgomery, J. E. Peralta, F. Ogliaro, M. Bearpark, J. J. Heyd, E. Brothers, K. N. Kudin, V. N. Staroverov, R. Kobayashi, J. Normand, K. Raghavachari, A. Rendell, J. C. Burant, S. S. Iyengar, J. Tomasi, M. Cossi, N. Rega, J. M. Millam, M. Klene, J. E. Knox, J. B. Cross, V. Bakken, C. Adamo, J. Jaramillo, R. Gomperts, R. E. Stratmann, O. Yazyev, A. J. Austin, R. Cammi, C. Pomelli, J. W. Ochterski, R. L. Martin, K. Morokuma, V. G. Zakrzewski, G. A. Voth, P. Salvador, J. J. Dannenberg, S. Dapprich, A. D. Daniels, Ö. Farkas, J. B. Foresman, J. V. Ortiz, J. Cioslowski, D. J. Fox, Gaussian, Inc., Wallingford CT, 2009.

31 (a) A. D. Becke, *J. Chem. Phys*., 1993, **98**, 5648–5652; (b) C. Lee, W. Yang, R. G. Parr, *Phys. Rev. B*, 1998, **37**, 785–789; (c) S. H. Vosko, L. Wilk, M. Nusair, *Can. J. Phys*., 1980, **58**, 1200–1211.

32 S. Grimme, J. Antony, S. Ehrlich, H. Krieg, *J. Chem. Phys*., 2010, **132**, 154104.

33 F. Weigend, R. Ahlrichs, *Phys. Chem. Chem. Phys*., 2005, **7**, 3297–3305.

34 (a) S. C. Schmid, I. A. Guzei, I. Fernández, J. M. Schomaker, *ACS Catal*., 2018, **8**, 7907–7914; (b) H. J. Dequina, J. Eshon, S. C. Schmid, W. T. Raskopf, K. M. Sanders, I. Fernández, and J. M. Schomaker, *J. Org. Chem*. 2022, **87**, 10902–10907.

35 J. W. McIver, A. K. Komornicki, *J. Am. Chem. Soc.*, 1972, **94**, 2625–2633.

36 C. González, H. B. Schlegel, *J. Phys. Chem*., 1990, **94**, 5523

37 S. Grimme, *Chem. Eur. J.* **2012**, *18*, 9955–9964.

38 G. Luchini, J. V. Alegre-Requena, I. Funes-Ardoiz, R. S. Paton, *F1000 Research*, **2020**, *9*, 291.

14. NMR Spectra

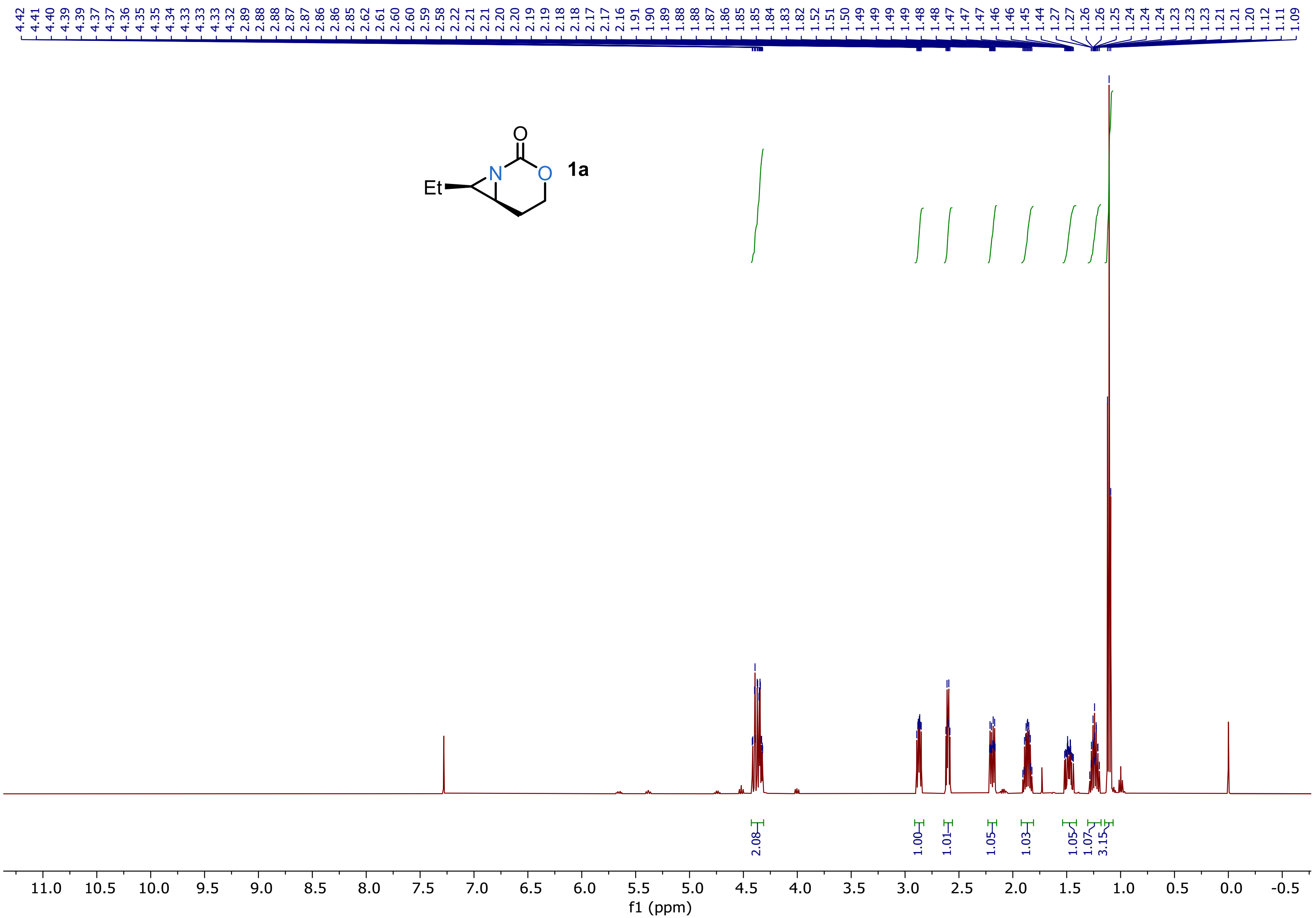

Figure S8. ^{1}H NMR (500 MHz, $CDCl_3$) spectrum of **1a**.

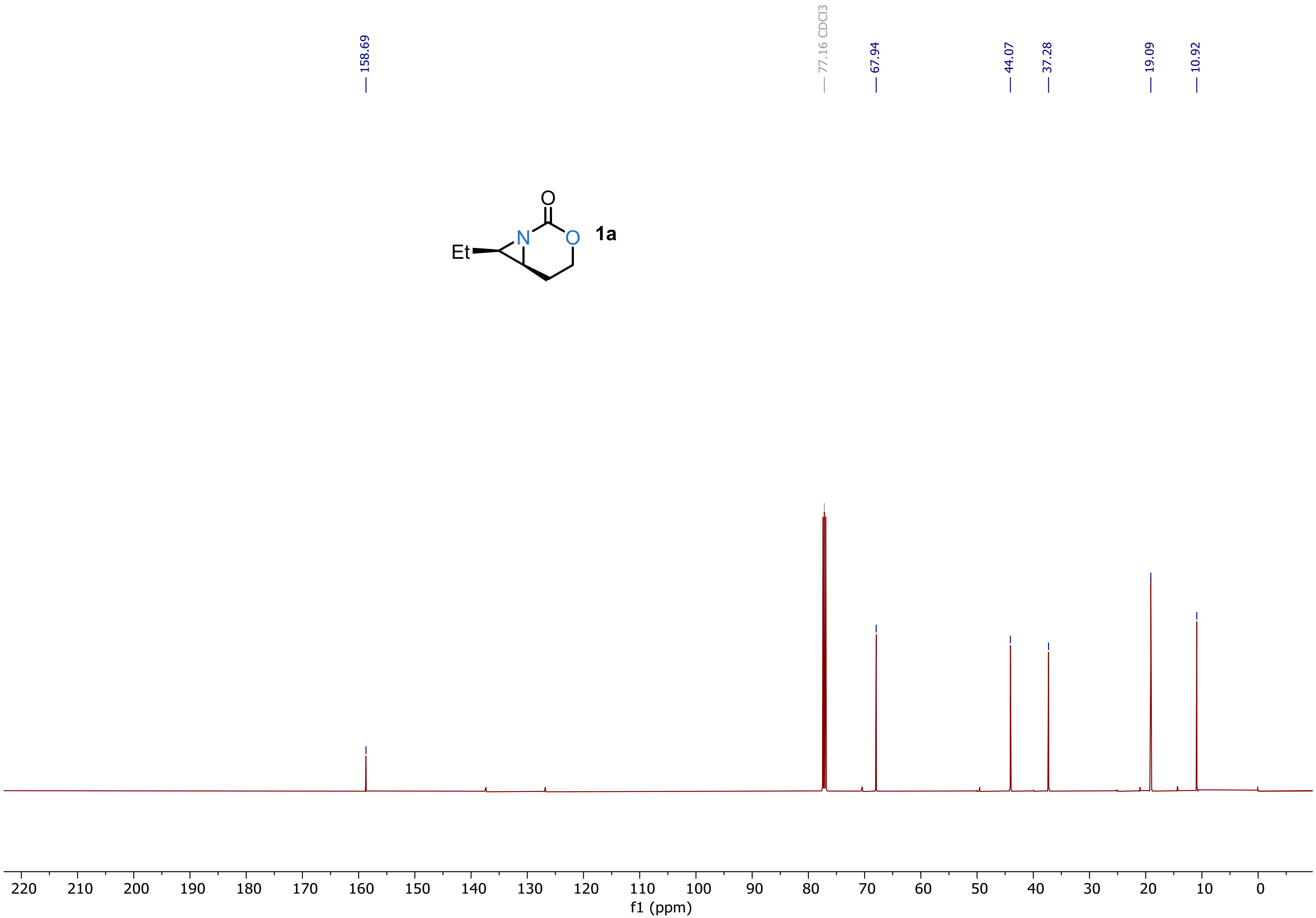

Figure S9. $^{13}C\{^{1}H\}$ NMR (126 MHz, $CDCl_3$) spectrum of **1a**.

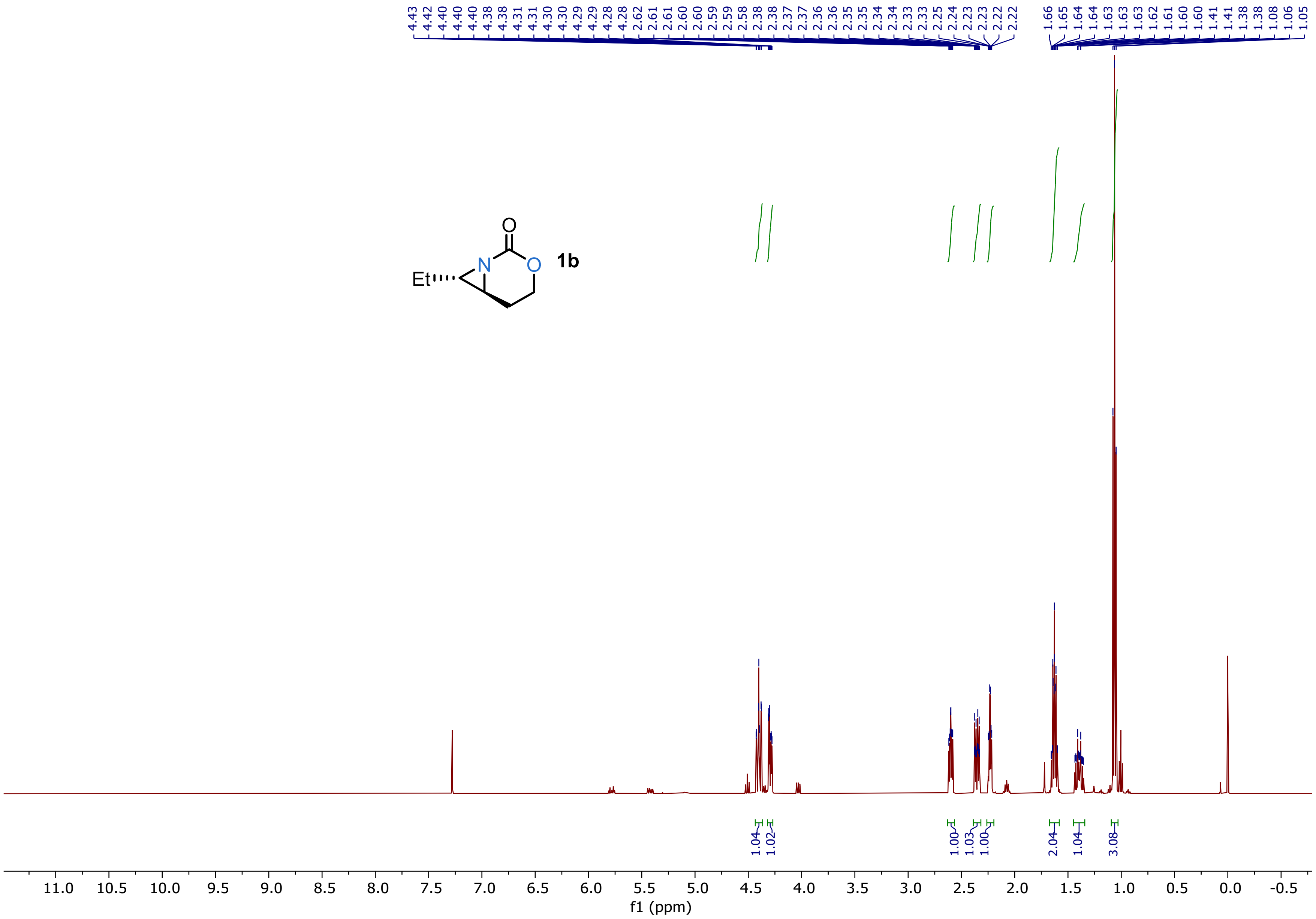

Figure S10. ^{1}H NMR (500 MHz, $CDCl_3$) spectrum of **1b**.

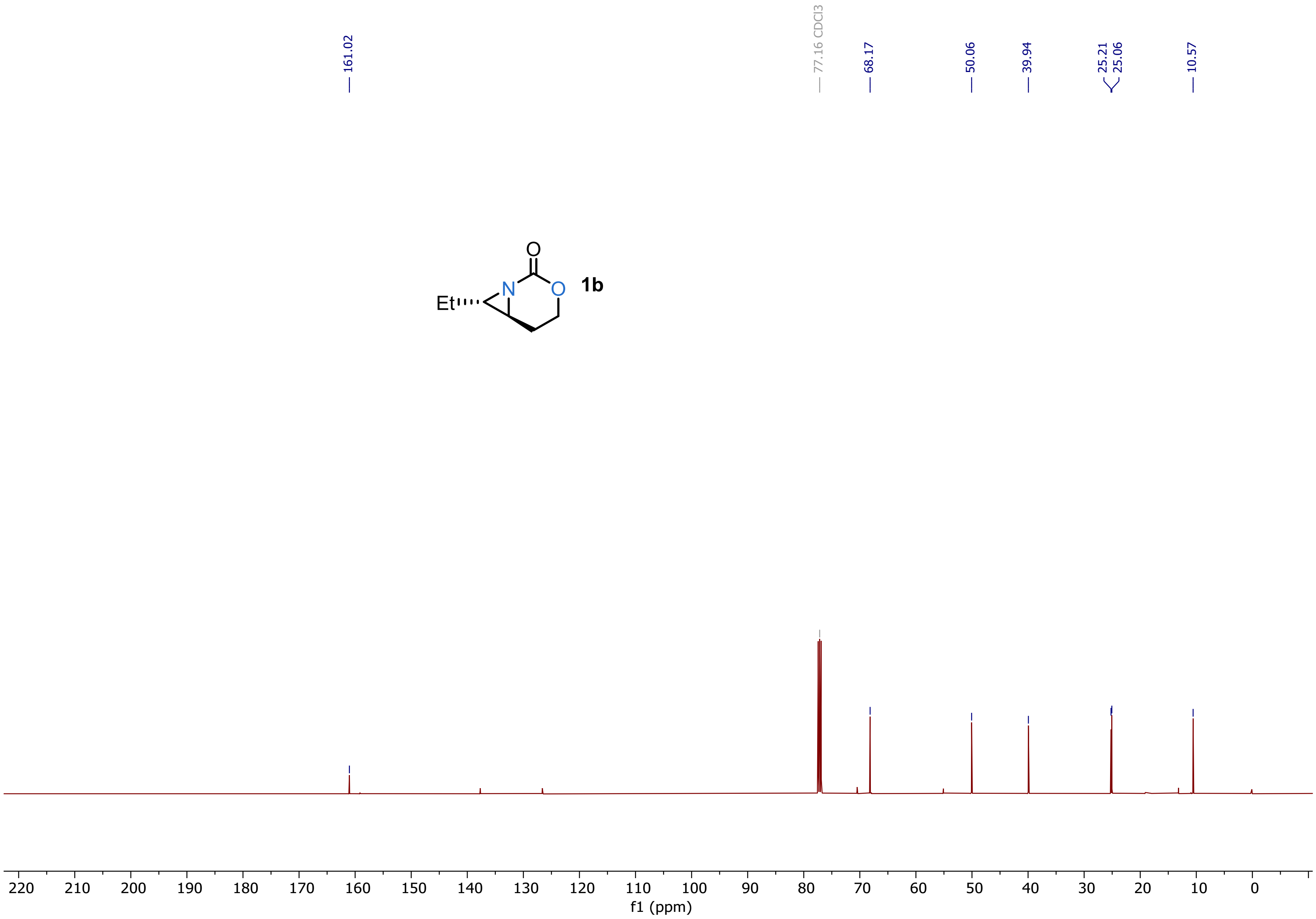

Figure S11. $^{13}C\{^{1}H\}$ NMR (126 MHz, $CDCl_3$) spectrum of **1b**.

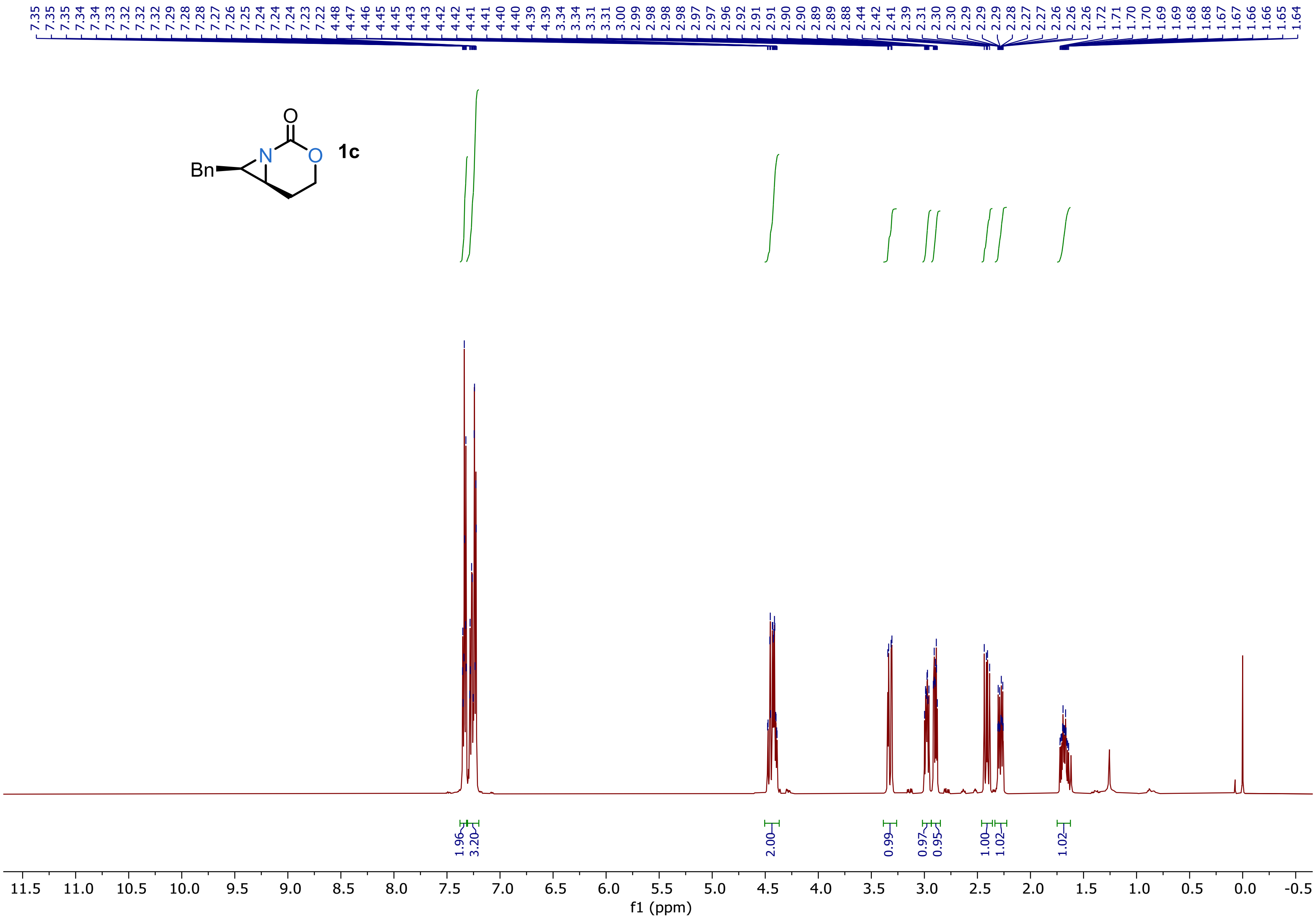

Figure S12. ^{1}H NMR (500 MHz, $CDCl_3$) spectrum of **1c**.

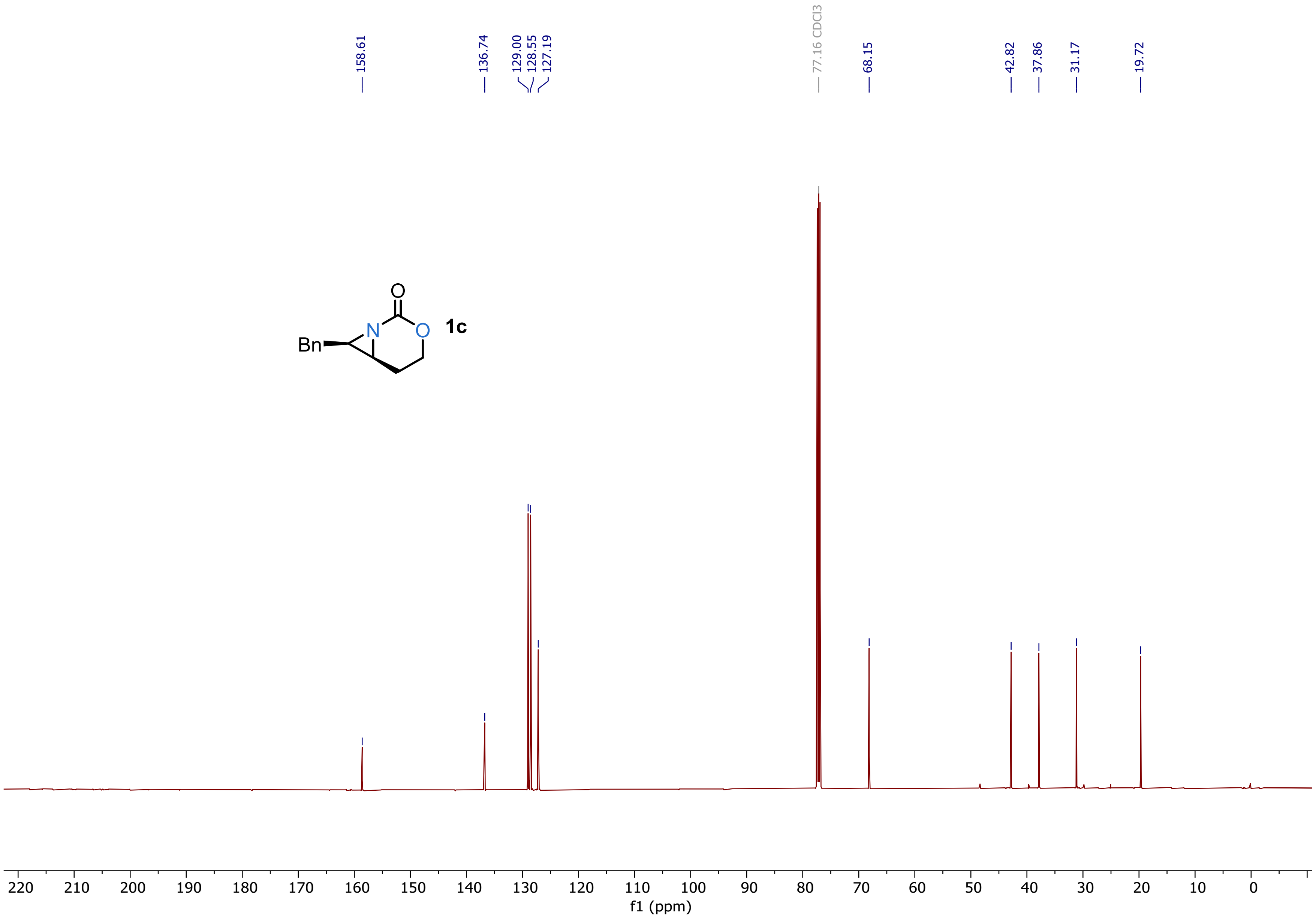

Figure S13. $^{13}C\{^1H\}$ NMR (126 MHz, $CDCl_3$) spectrum of **1c**.

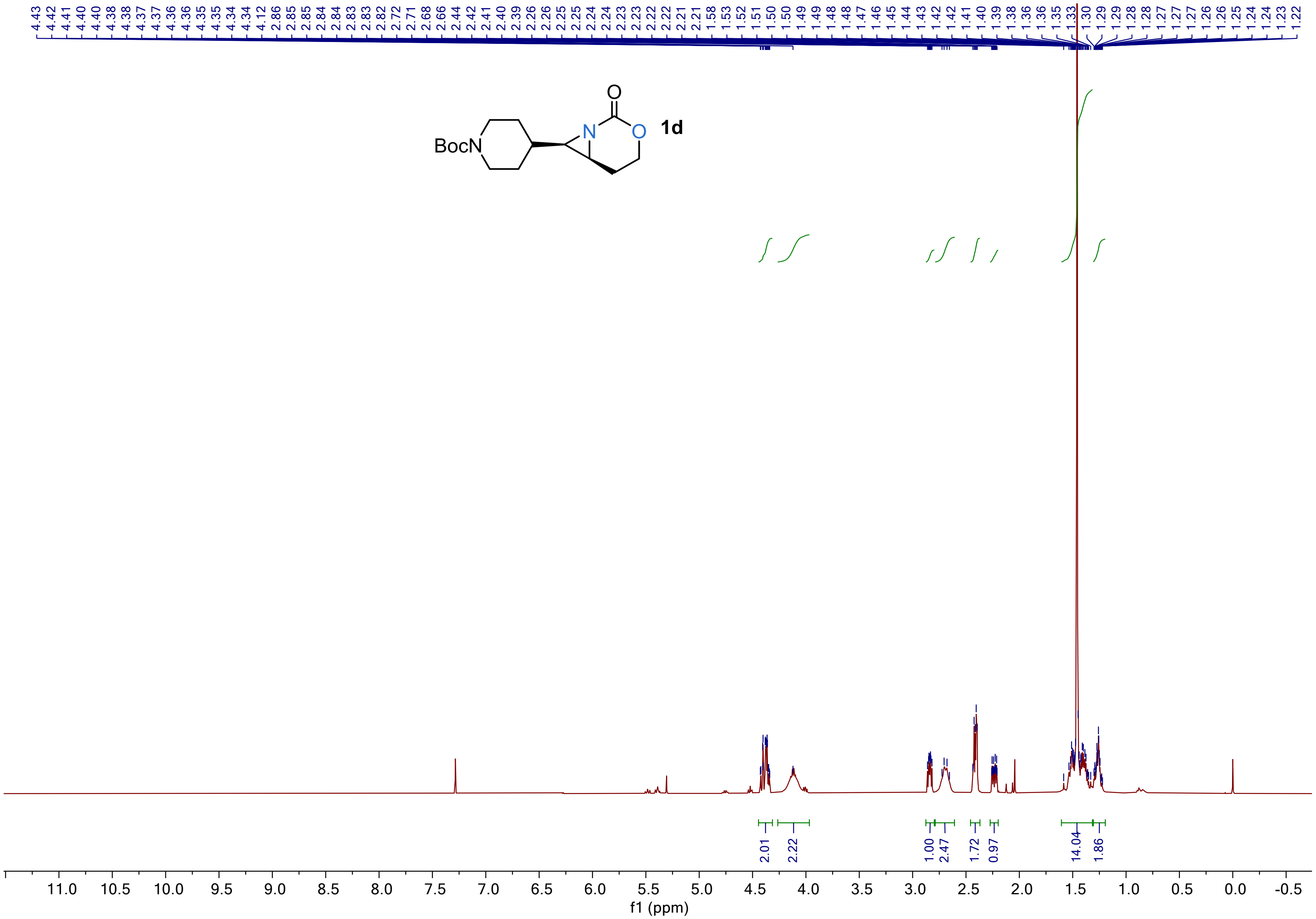

Figure S14. ^{1}H NMR (500 MHz, $CDCl_3$) spectrum of **1d**.

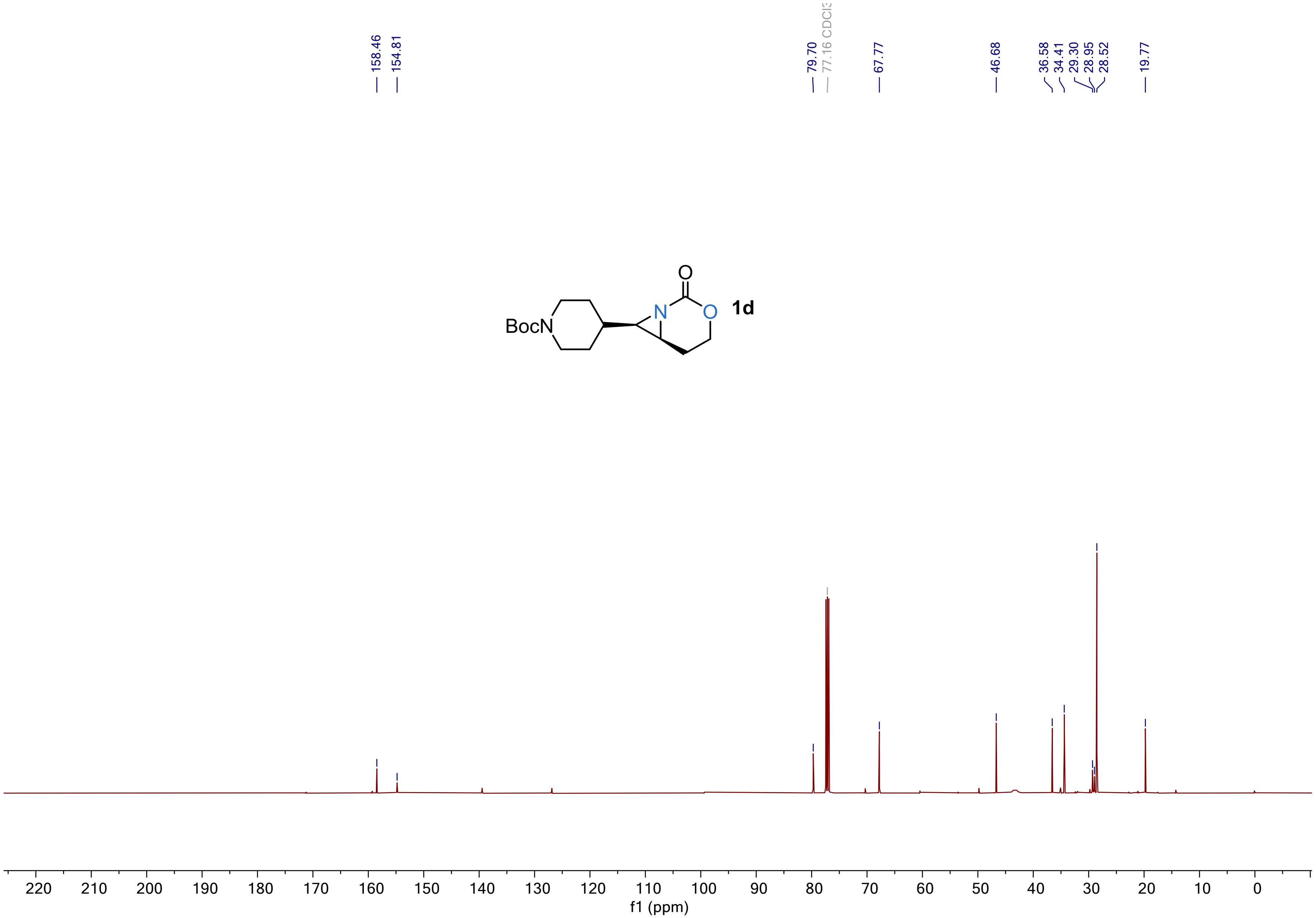

Figure S15. $^{13}C\{^{1}H\}$ NMR (126 MHz, $CDCl_3$) spectrum of **1d**.

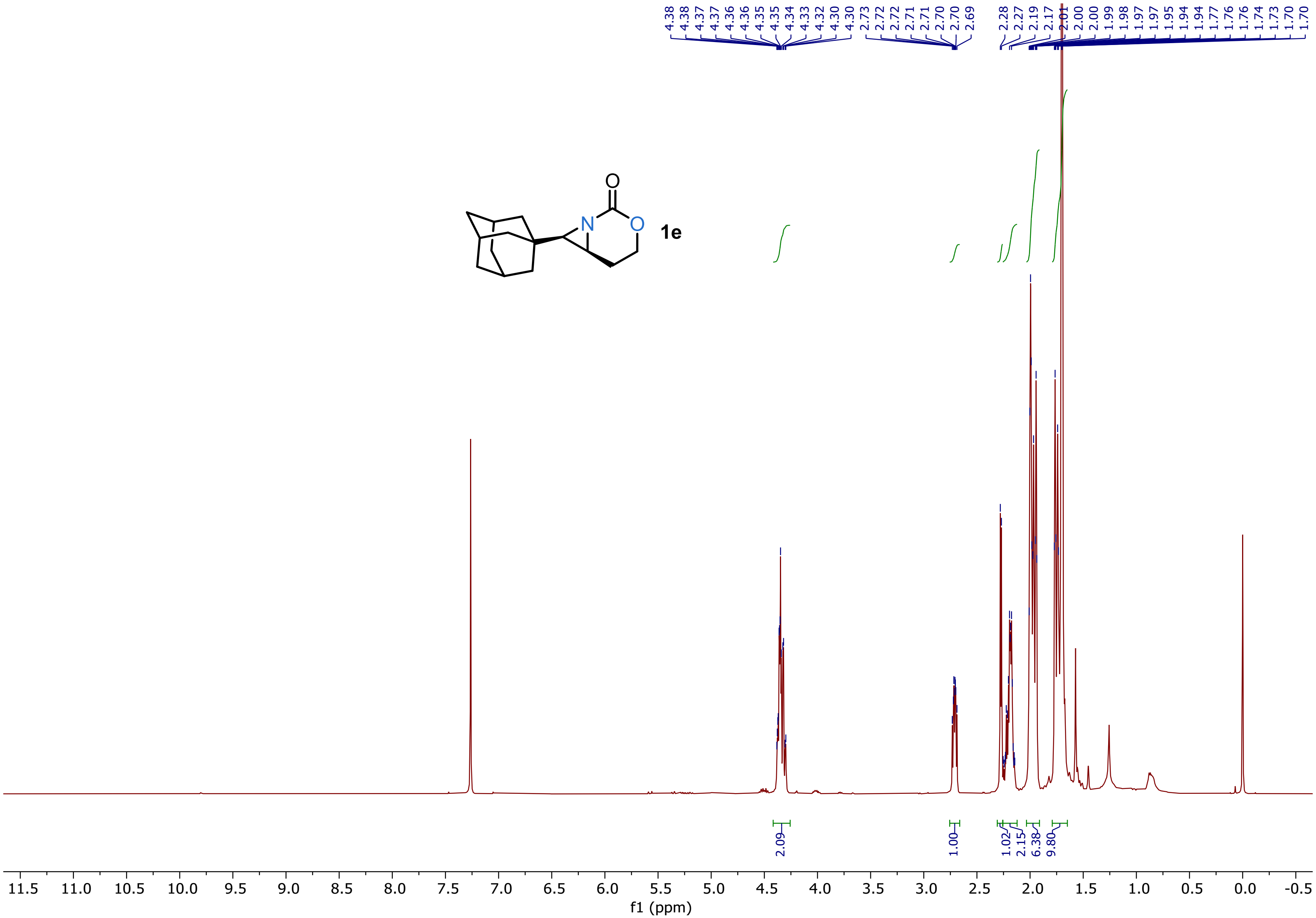

Figure S16. ¹H NMR (500 MHz, $CDCl_3$) spectrum of **1e**.

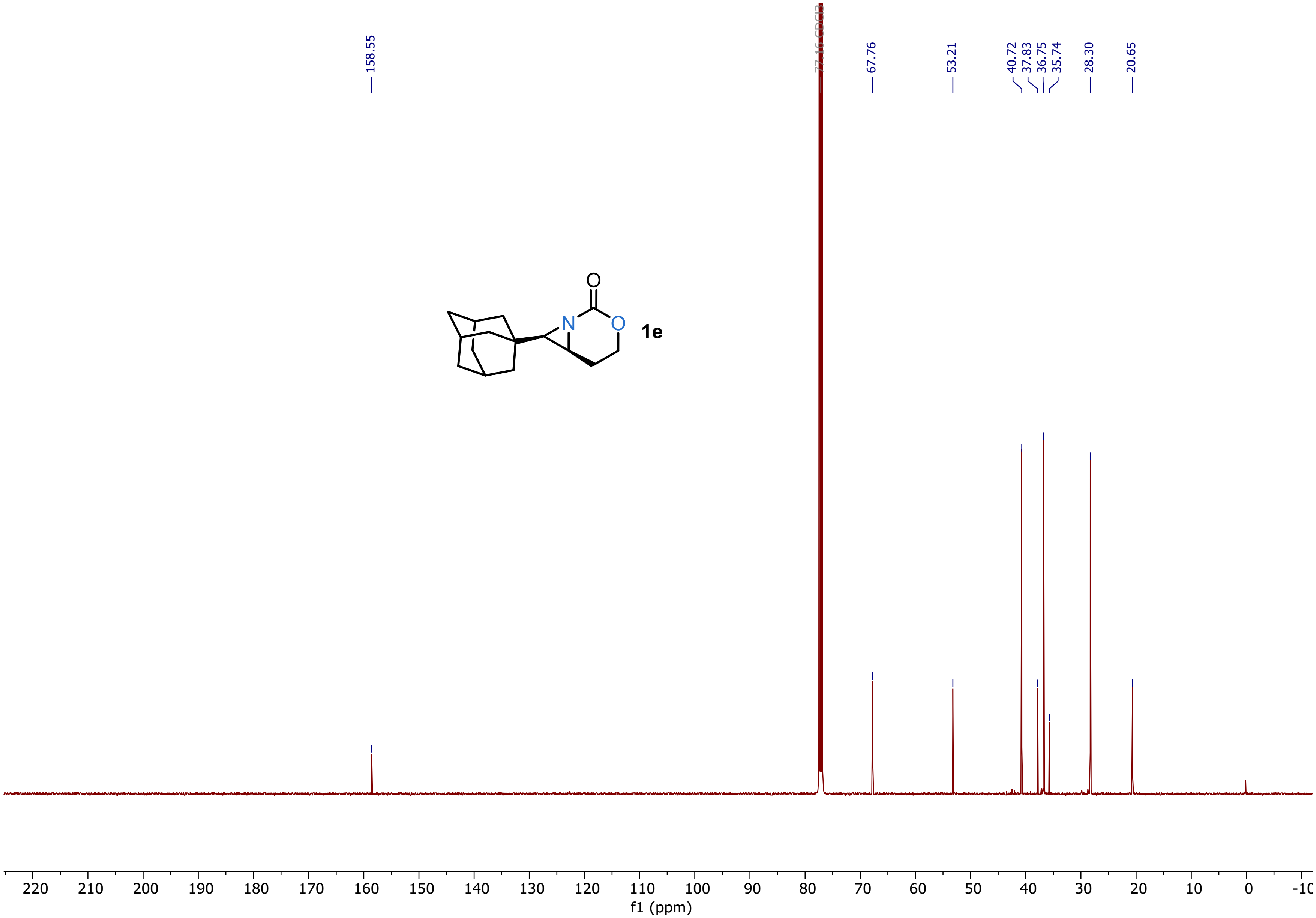

Figure S17. $^{13}C\{^1H\}$ NMR (126 MHz, $CDCl_3$) spectrum of **1e**.

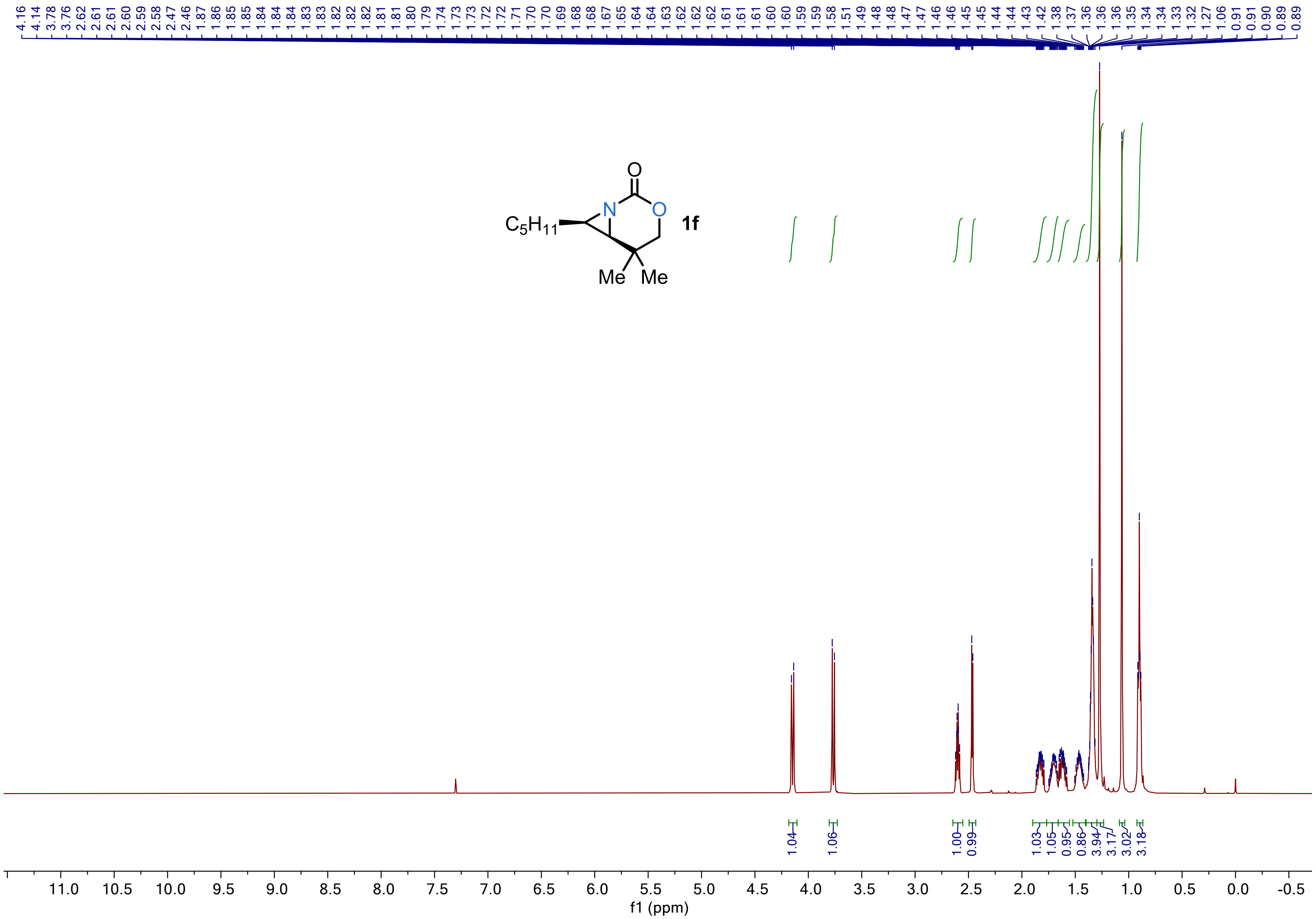

Figure S18. ^{1}H NMR (500 MHz, $CDCl_3$) spectrum of **1f**.

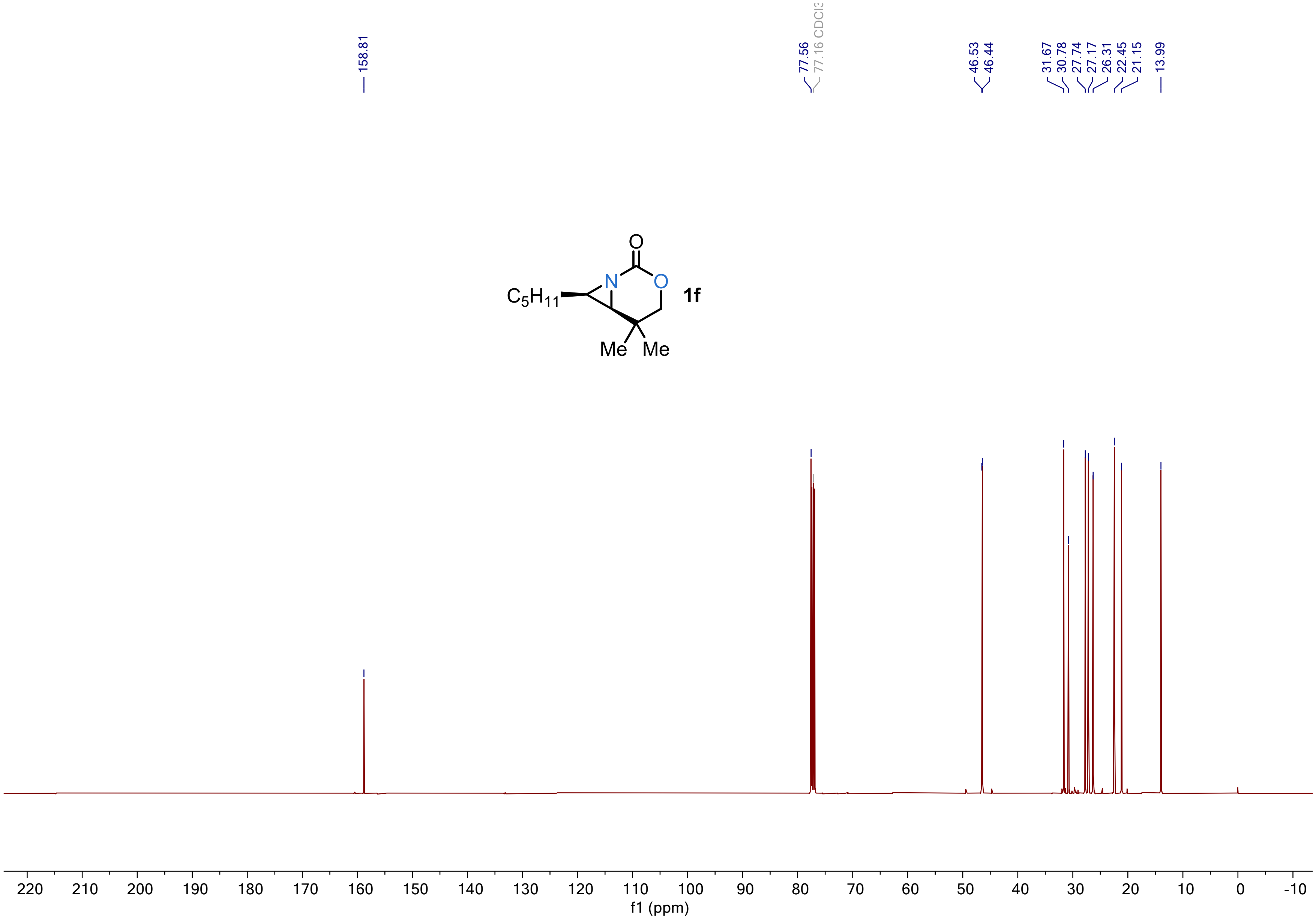

Figure S19. $^{13}C\{^{1}H\}$ NMR (126 MHz, $CDCl_3$) spectrum of **1f**.

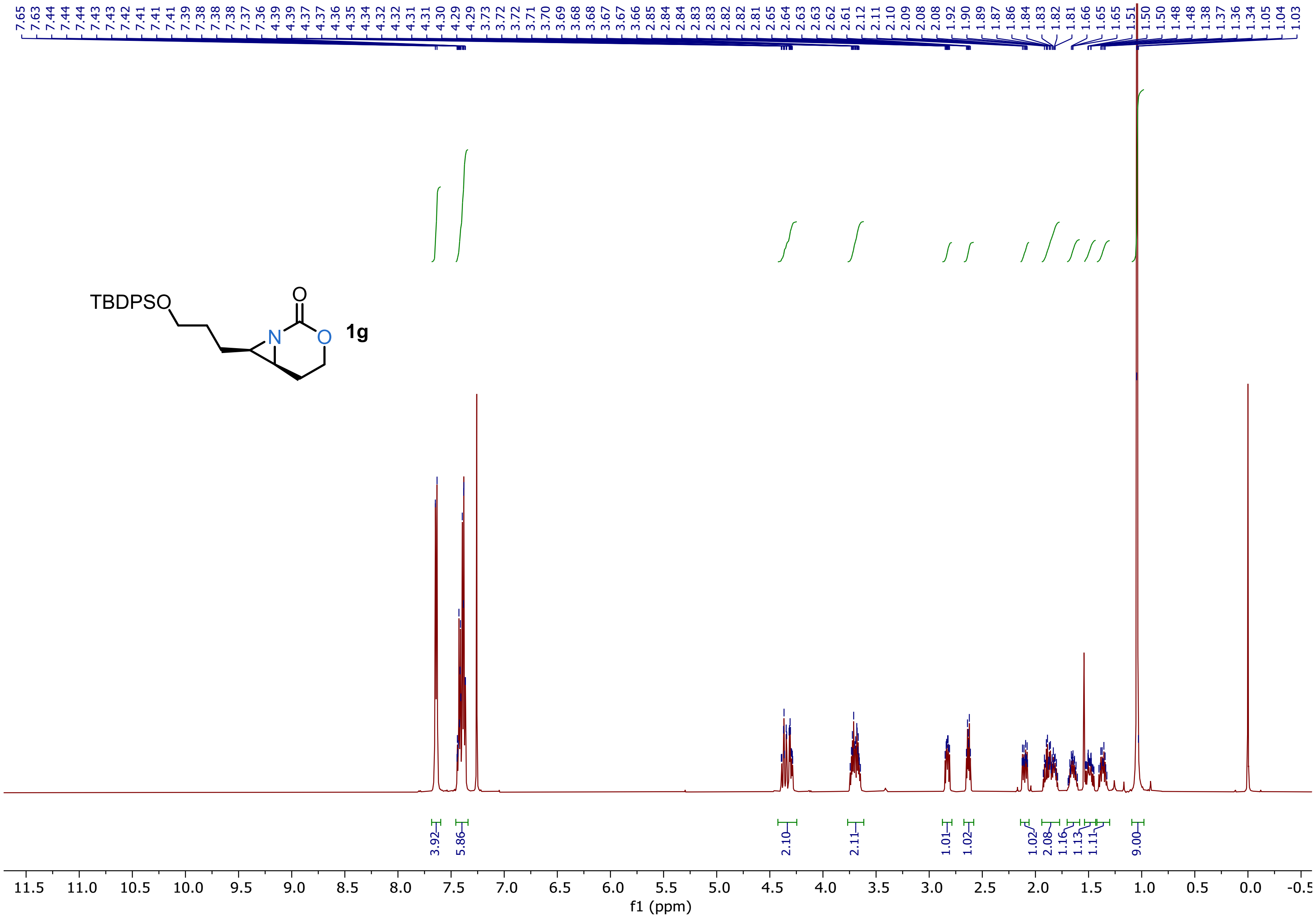

Figure S20. ^{1}H NMR (500 MHz, $CDCl_3$) spectrum of **1g**.

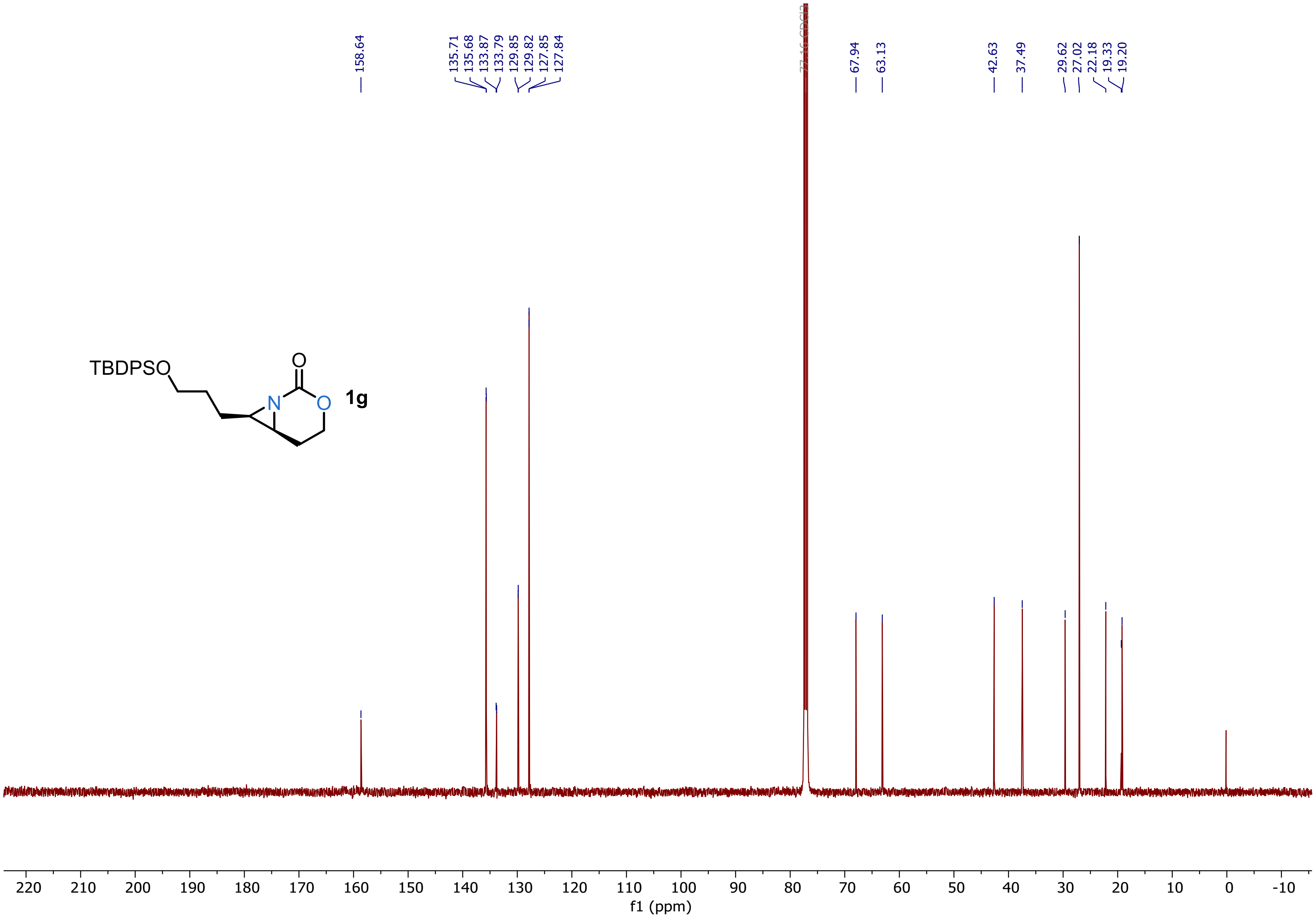

Figure S21. $^{13}C\{^1H\}$ NMR (126 MHz, $CDCl_3$) spectrum of **1g**.

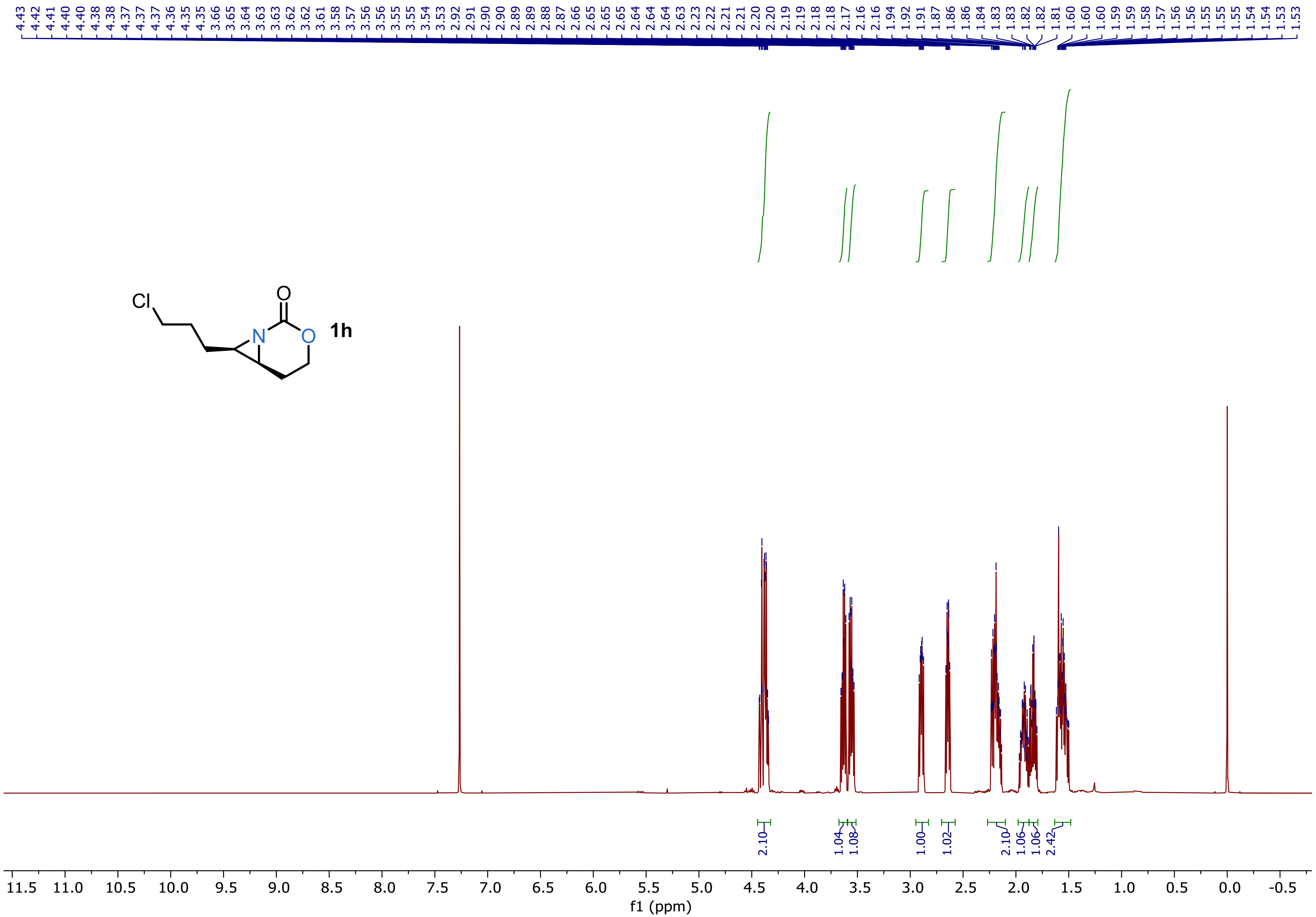

Figure S22. ^{1}H NMR (500 MHz, $CDCl_3$) spectrum of **1h**.

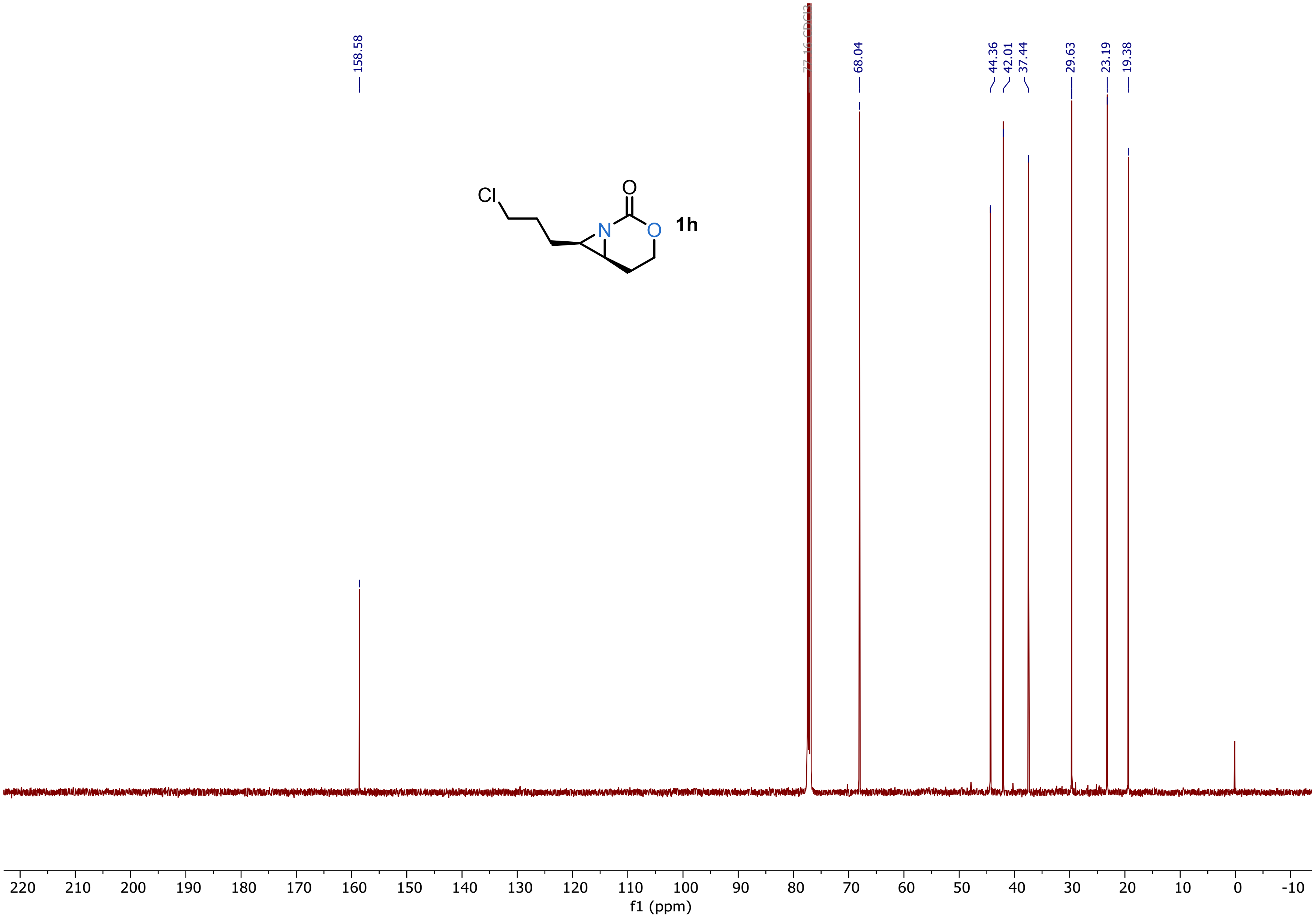

Figure S23. $^{13}C\{^{1}H\}$ NMR (126 MHz, $CDCl_3$) spectrum of **1h**.

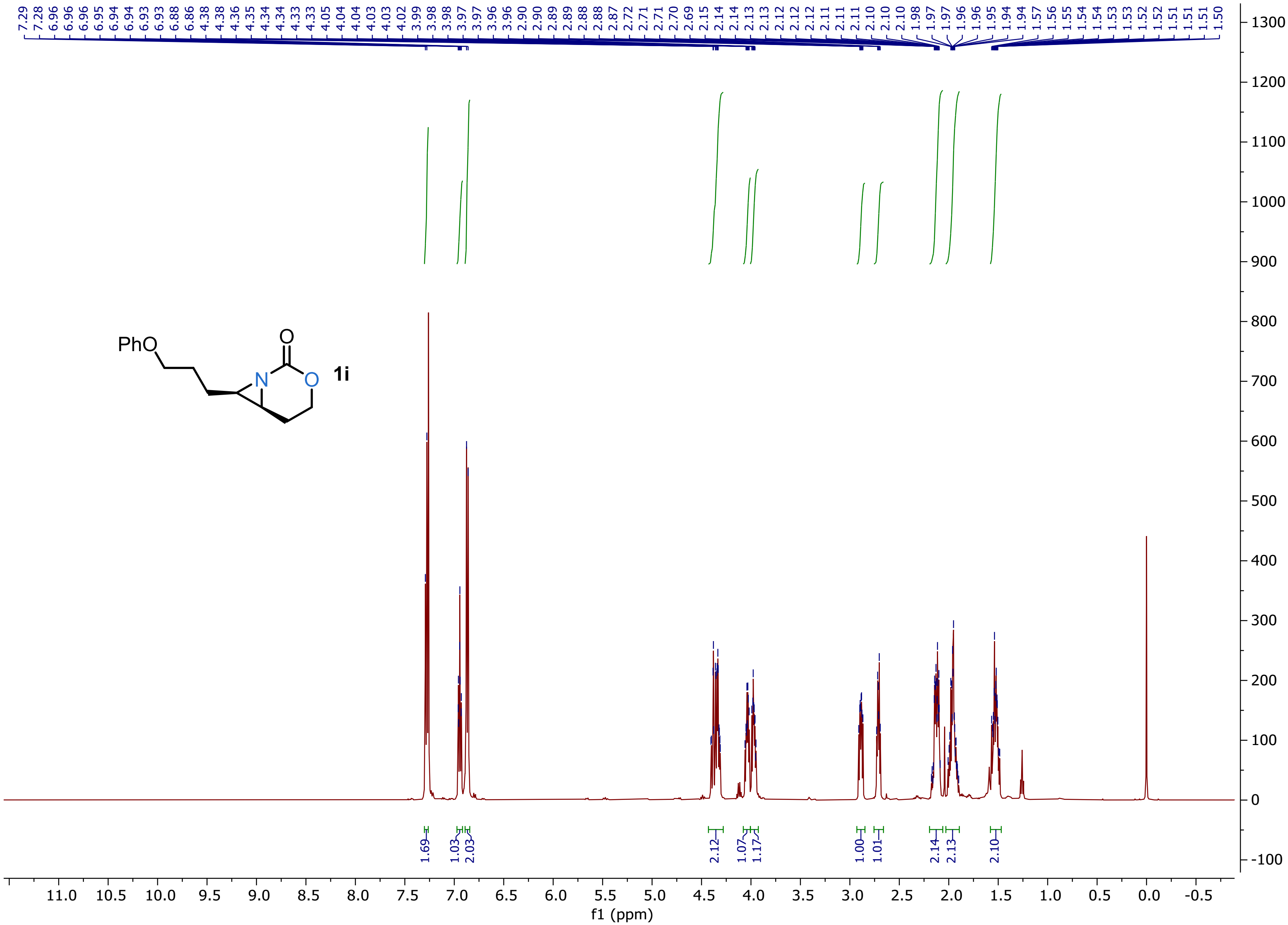

Figure S24. ^{1}H NMR (500 MHz, $CDCl_3$) spectrum of **1i**.

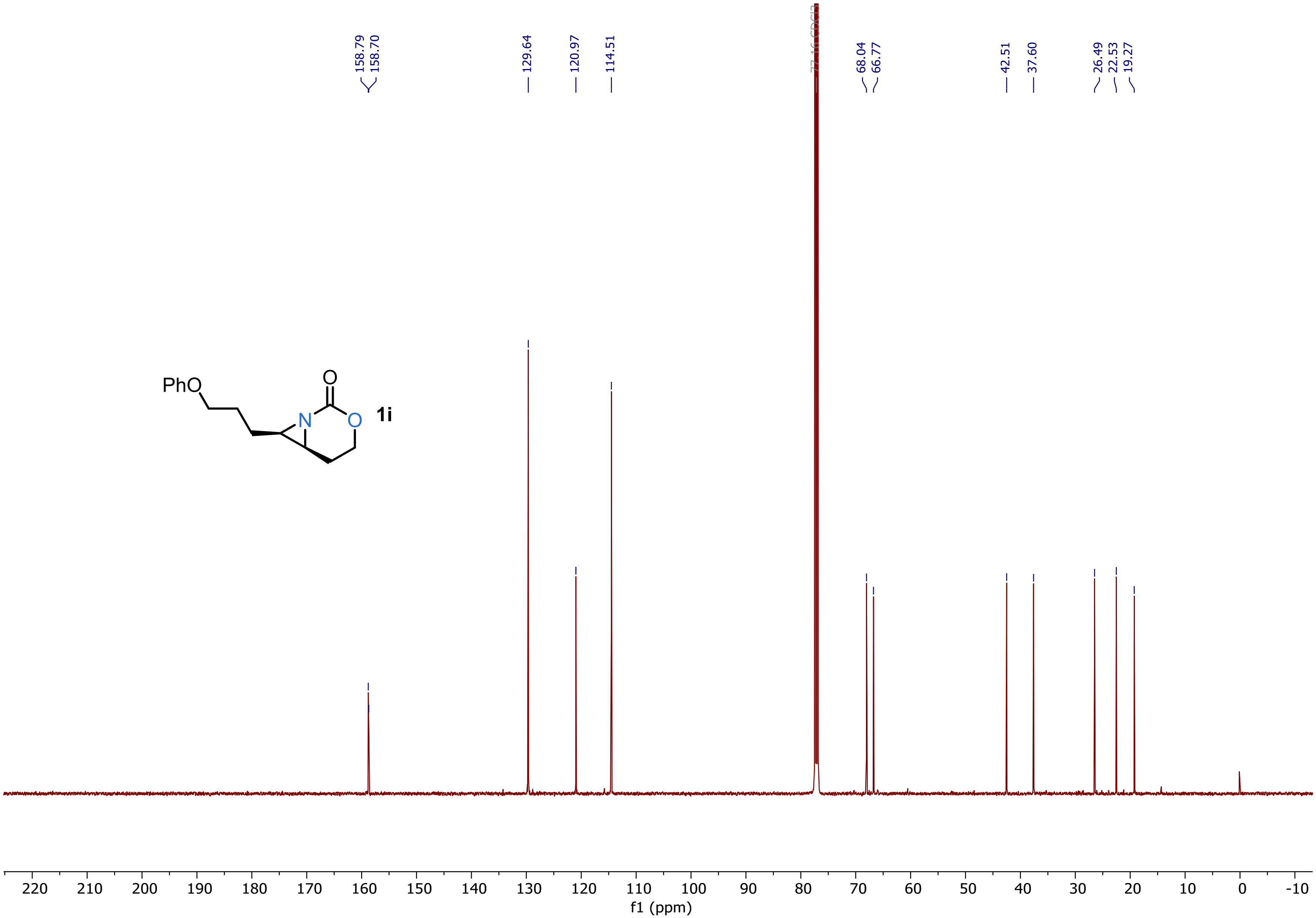

Figure S25. $^{13}C\{^{1}H\}$ NMR (126 MHz, $CDCl_3$) spectrum of **1i**.

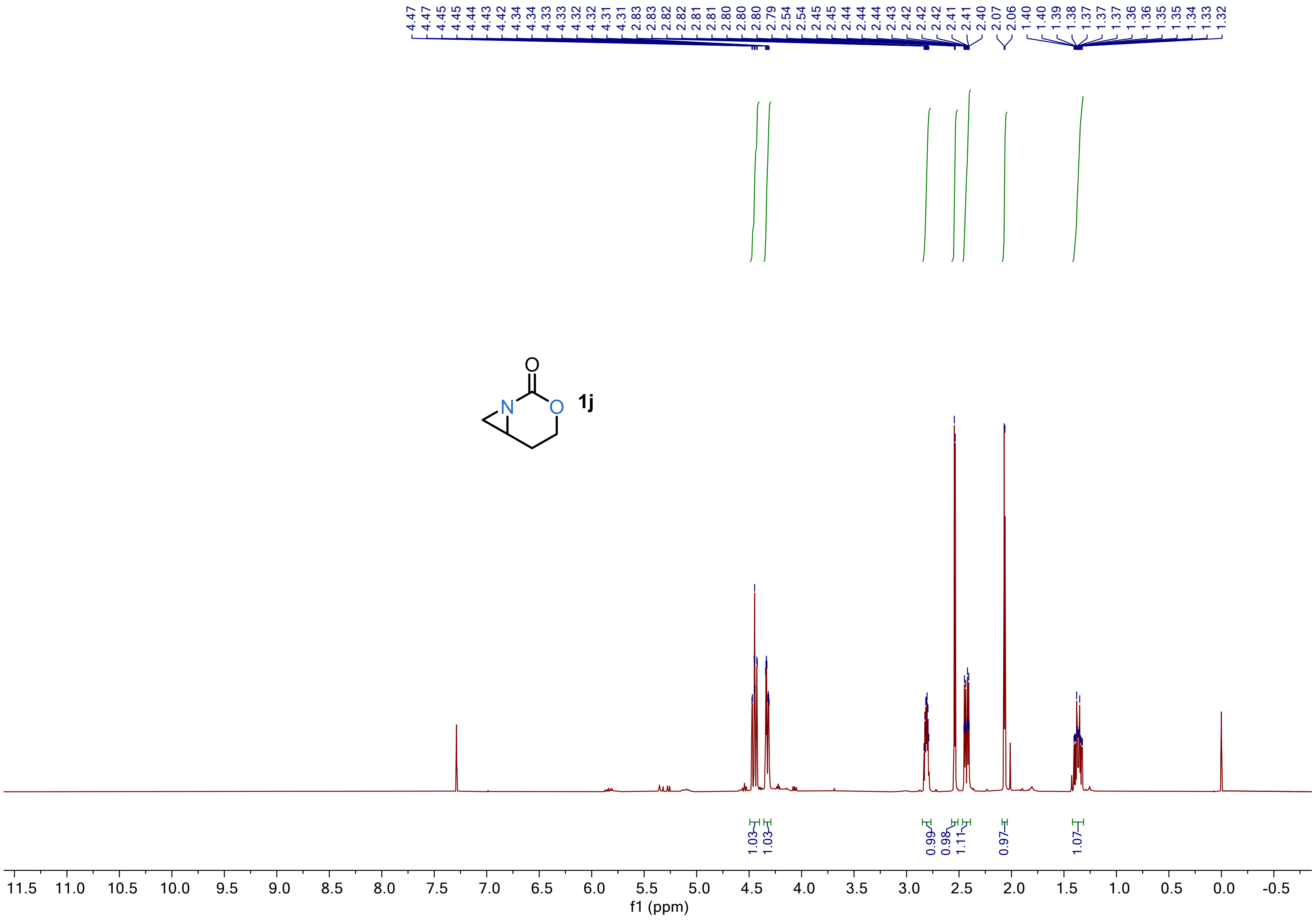

Figure S26. ^{1}H NMR (500 MHz, $CDCl_3$) spectrum of **1j**.

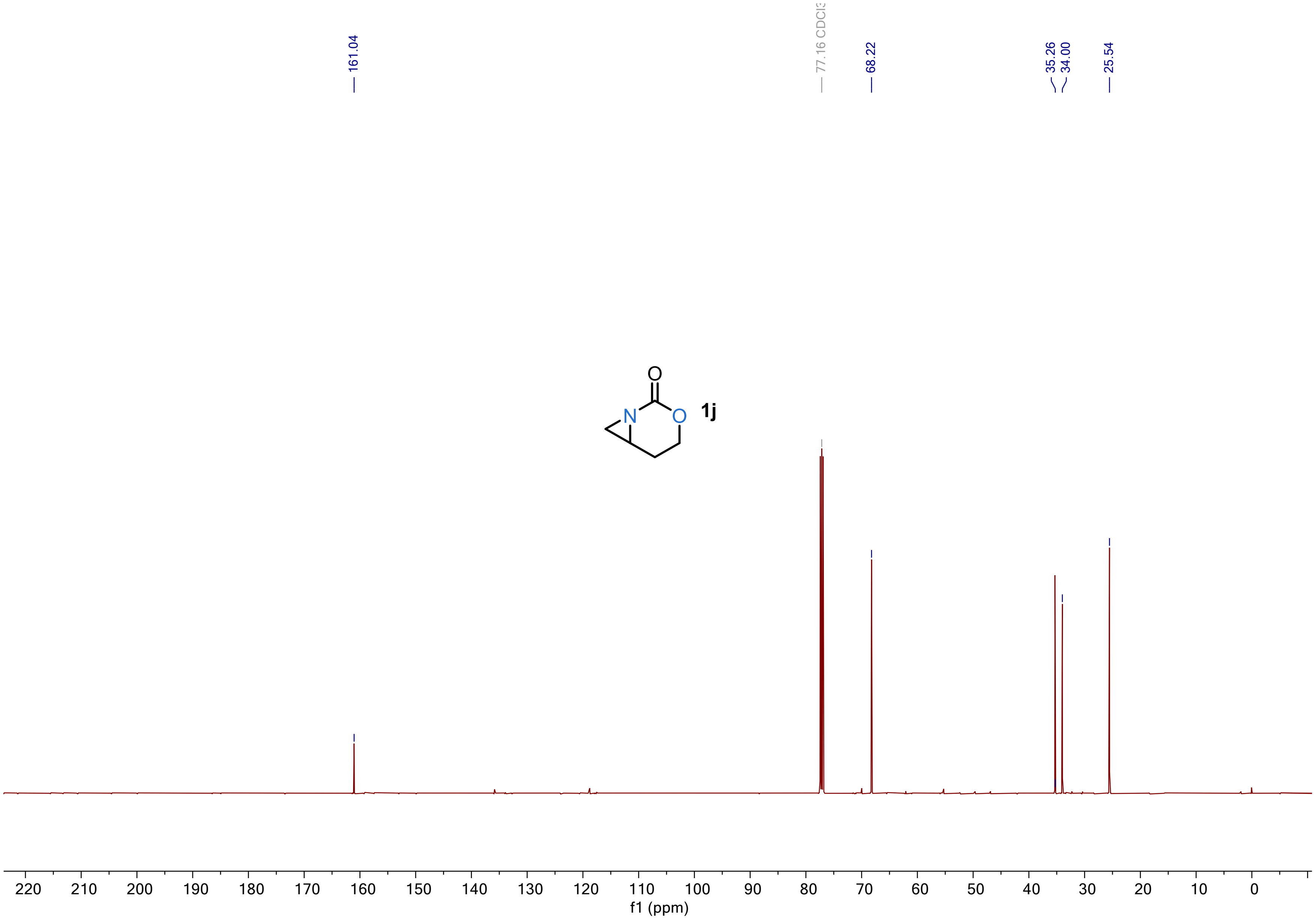

Figure S27. $^{13}C\{^{1}H\}$ NMR (126 MHz, $CDCl_3$) spectrum of **1j**.

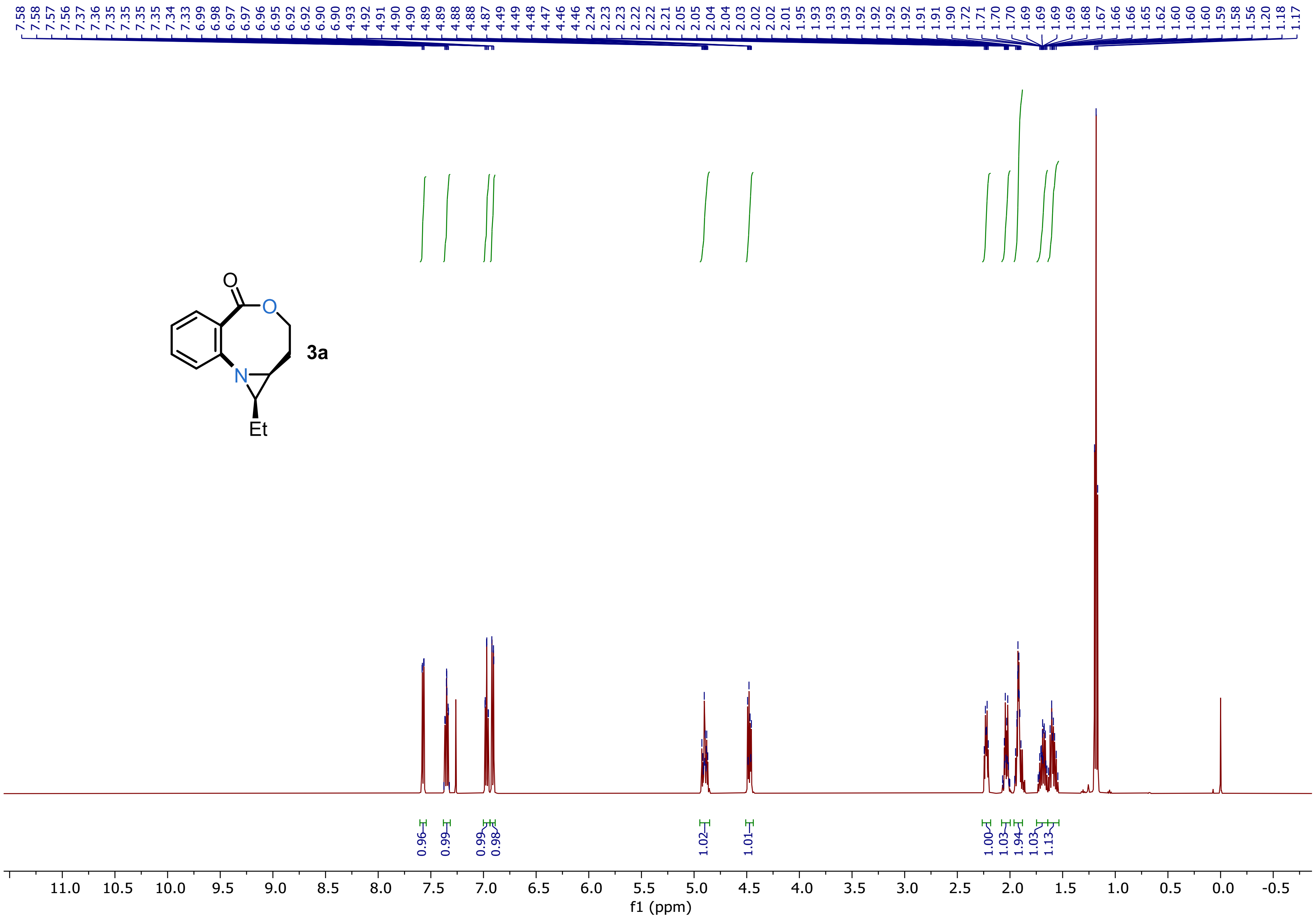

Figure S28. ^{1}H NMR (500 MHz, $CDCl_3$) spectrum of **3a**.

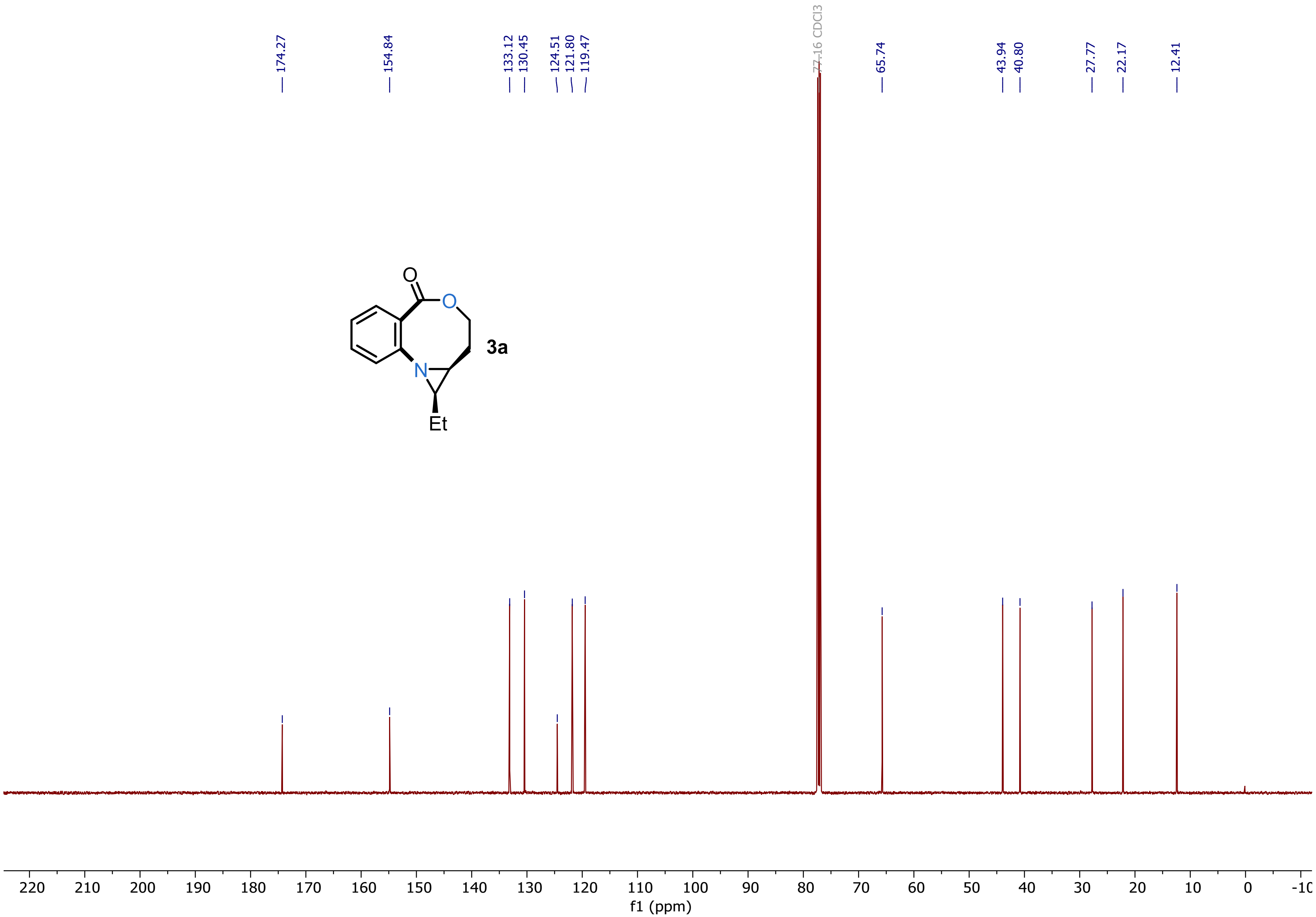

Figure S29. $^{13}C\{^{1}H\}$ NMR (126 MHz, $CDCl_3$) spectrum of **3a**.

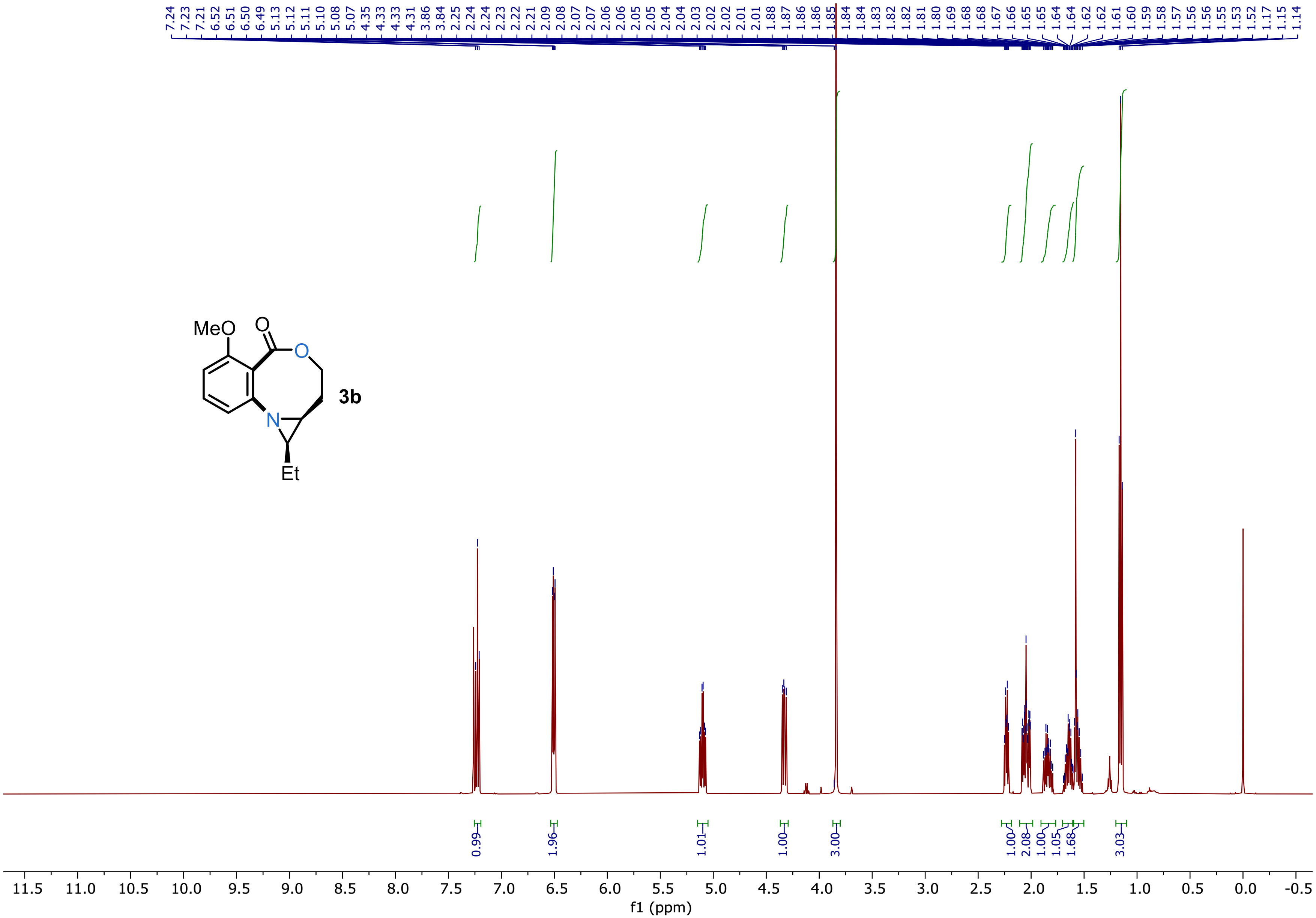

Figure S30. ^{1}H NMR (500 MHz, $CDCl_3$) spectrum of **3b**.

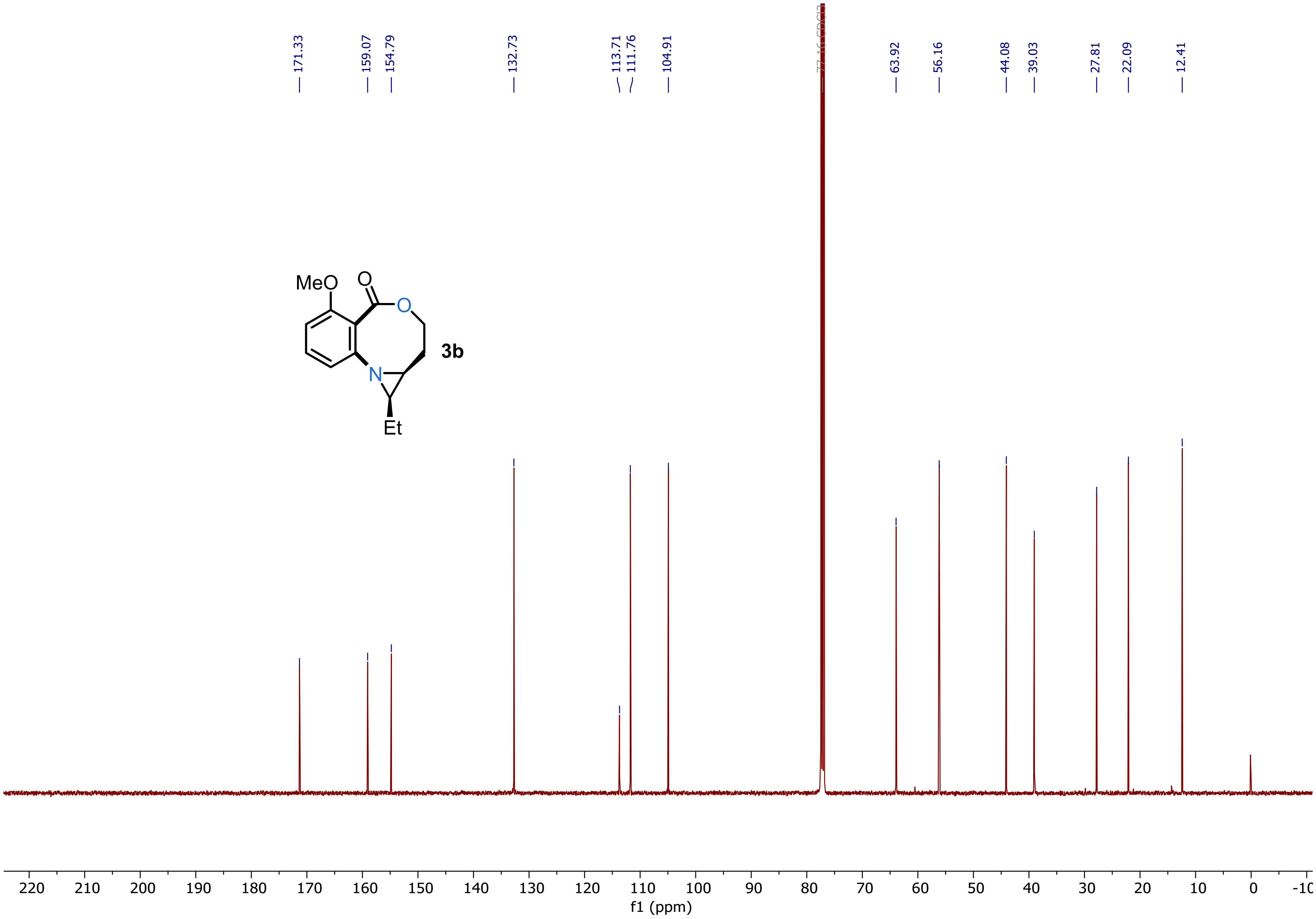

Figure S31. $^{13}C\{^{1}H\}$ NMR (126 MHz, $CDCl_3$) spectrum of **3b**.

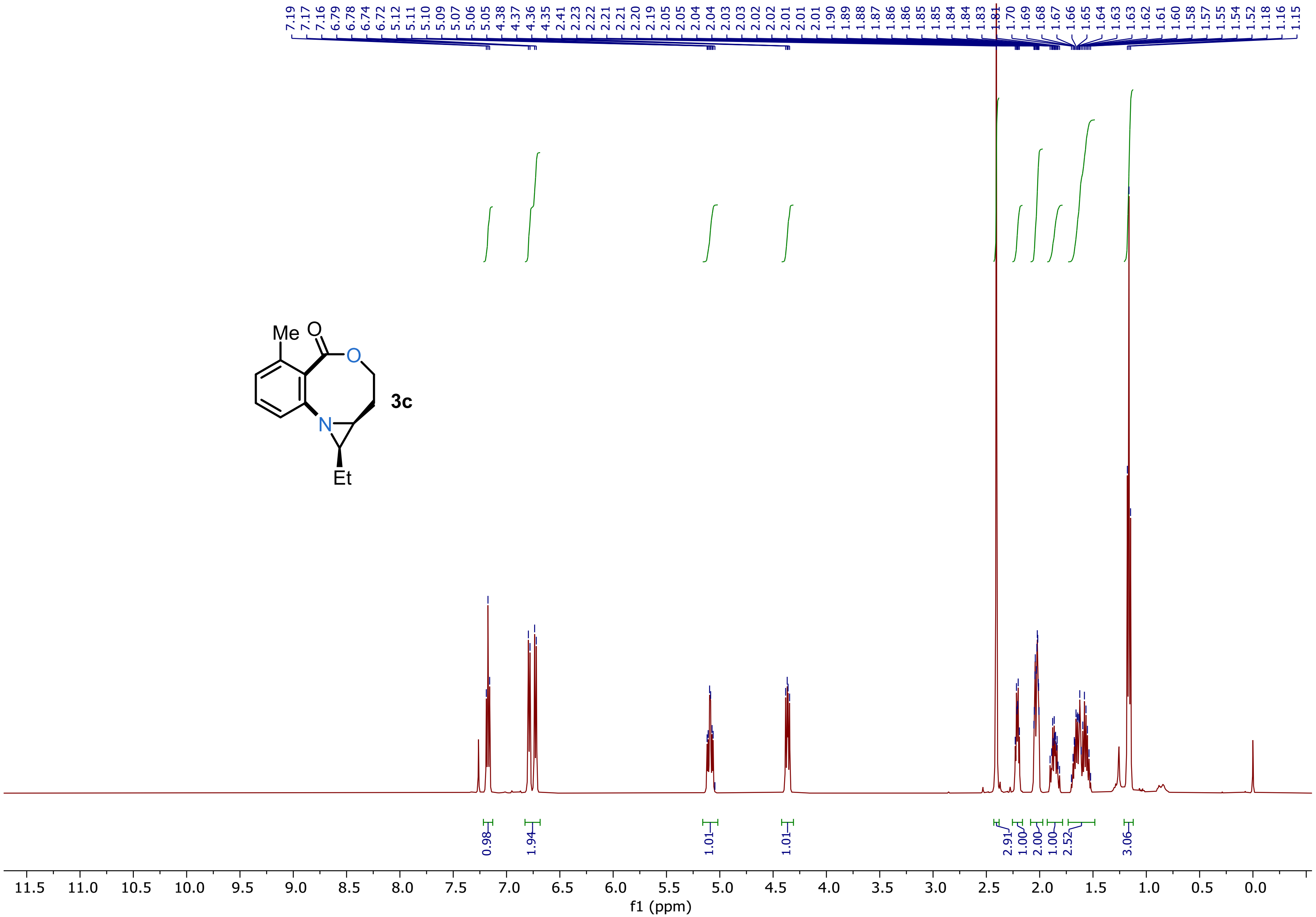

Figure S32. ¹H NMR (500 MHz, $CDCl_3$) spectrum of **3c**.

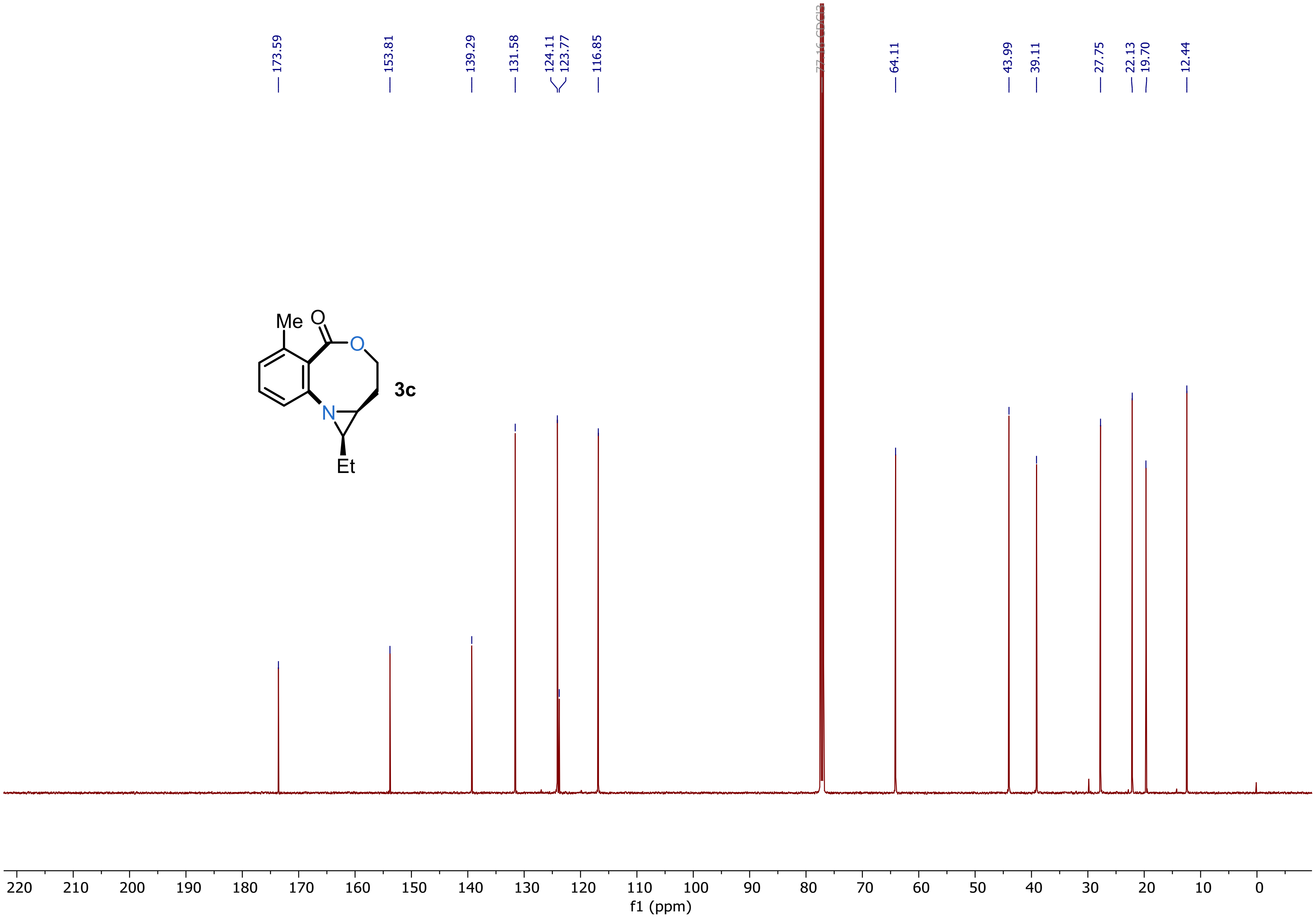

Figure S33. $^{13}C\{^1H\}$ NMR (126 MHz, $CDCl_3$) spectrum of **3c**.

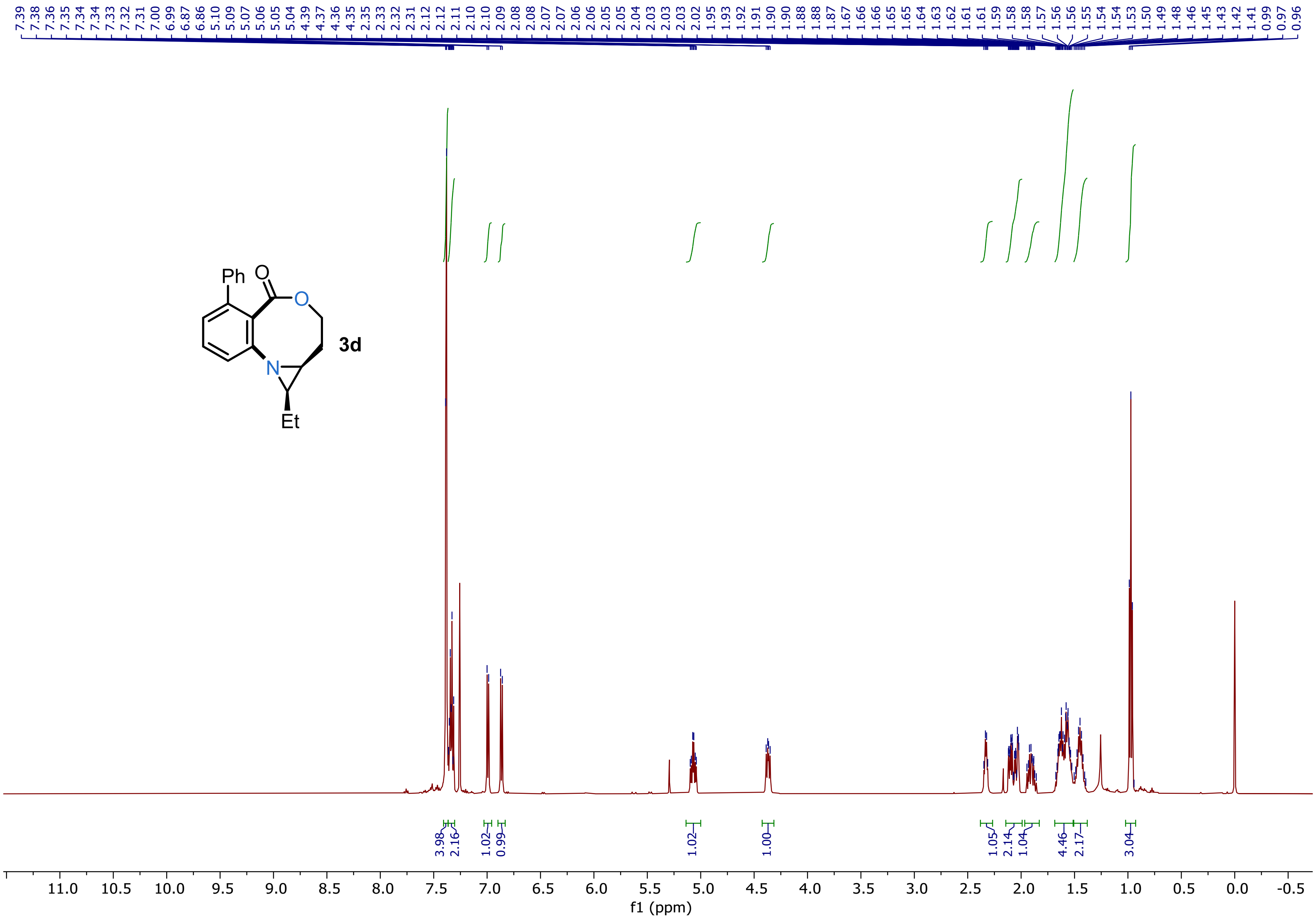

Figure S34. ^{1}H NMR (500 MHz, $CDCl_3$) spectrum of **3d**.

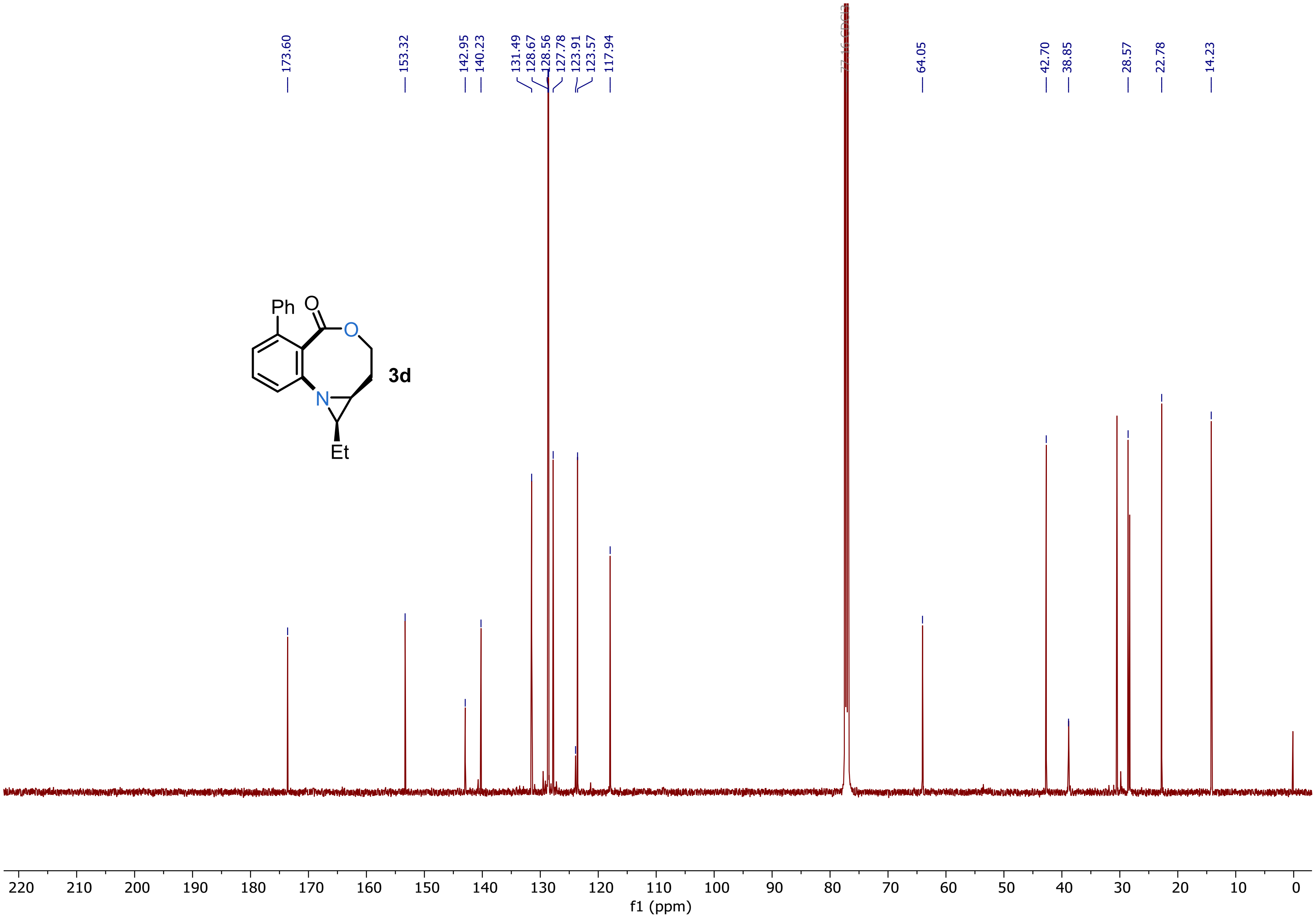

Figure S35. $^{13}C\{^{1}H\}$ NMR (126 MHz, $CDCl_3$) spectrum of **3d**.

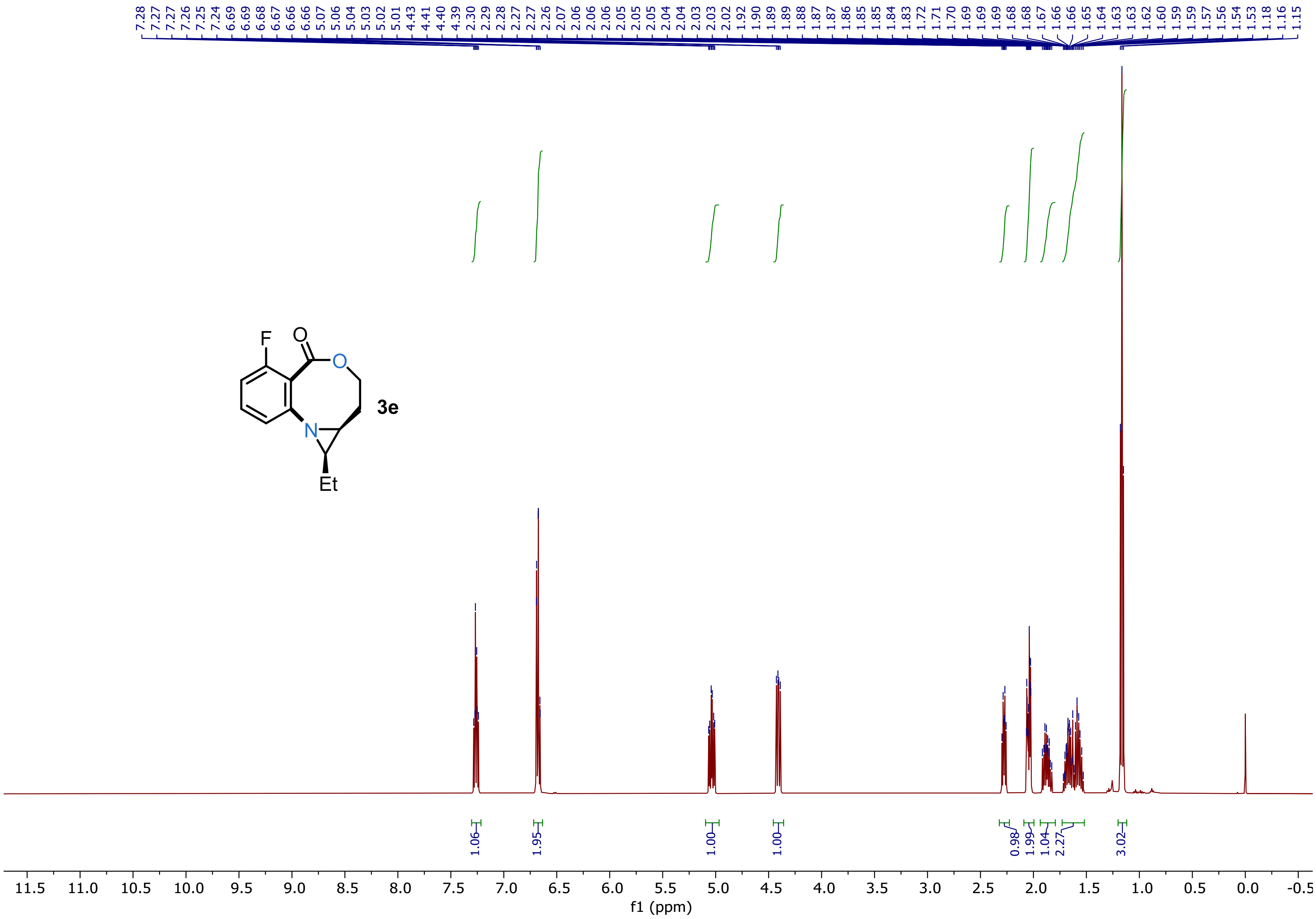

Figure S36. ^{1}H NMR (500 MHz, $CDCl_3$) spectrum of **3e**.

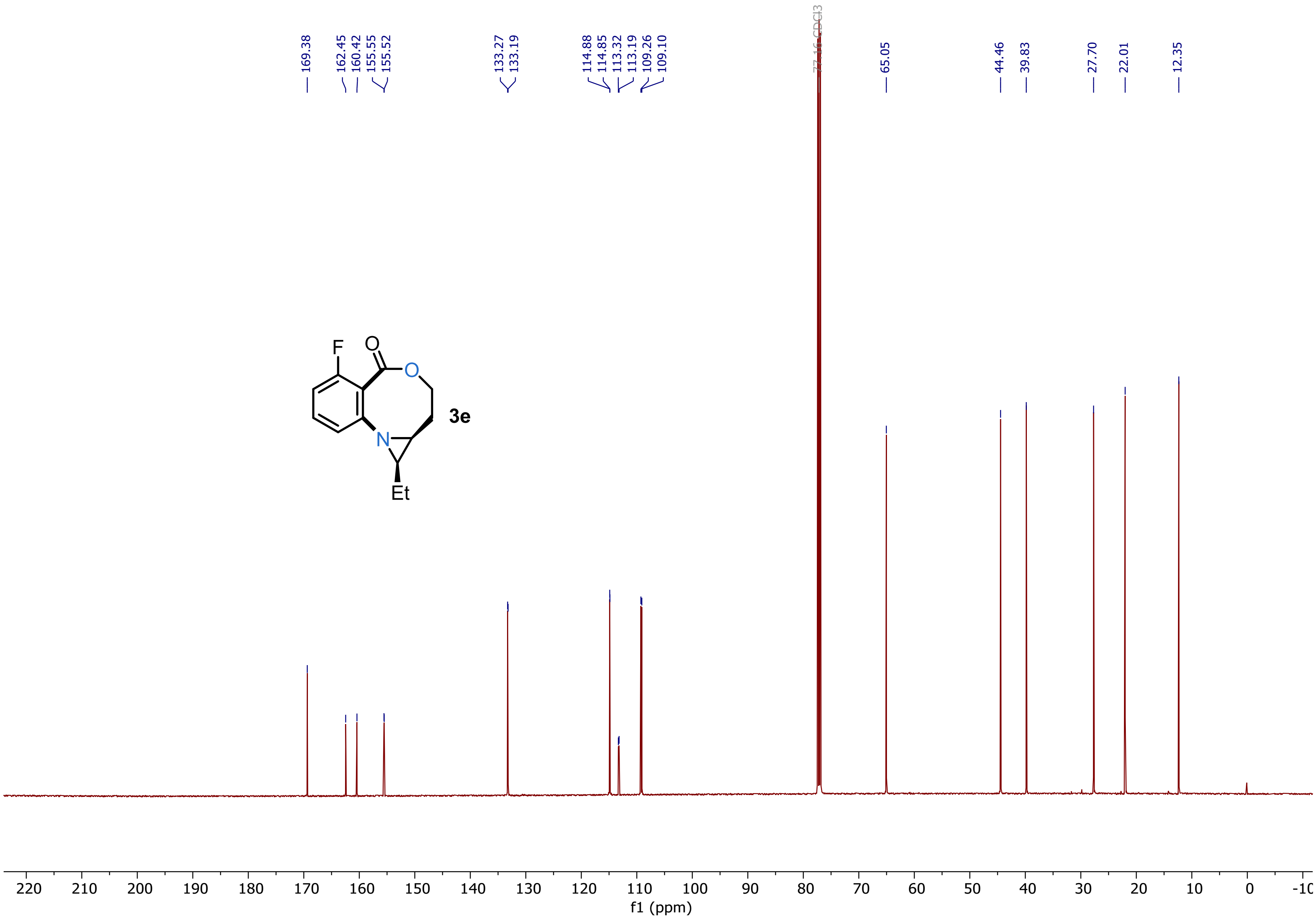

Figure S37. $^{13}C\{^{1}H\}$ NMR (126 MHz, $CDCl_3$) spectrum of **3e**.

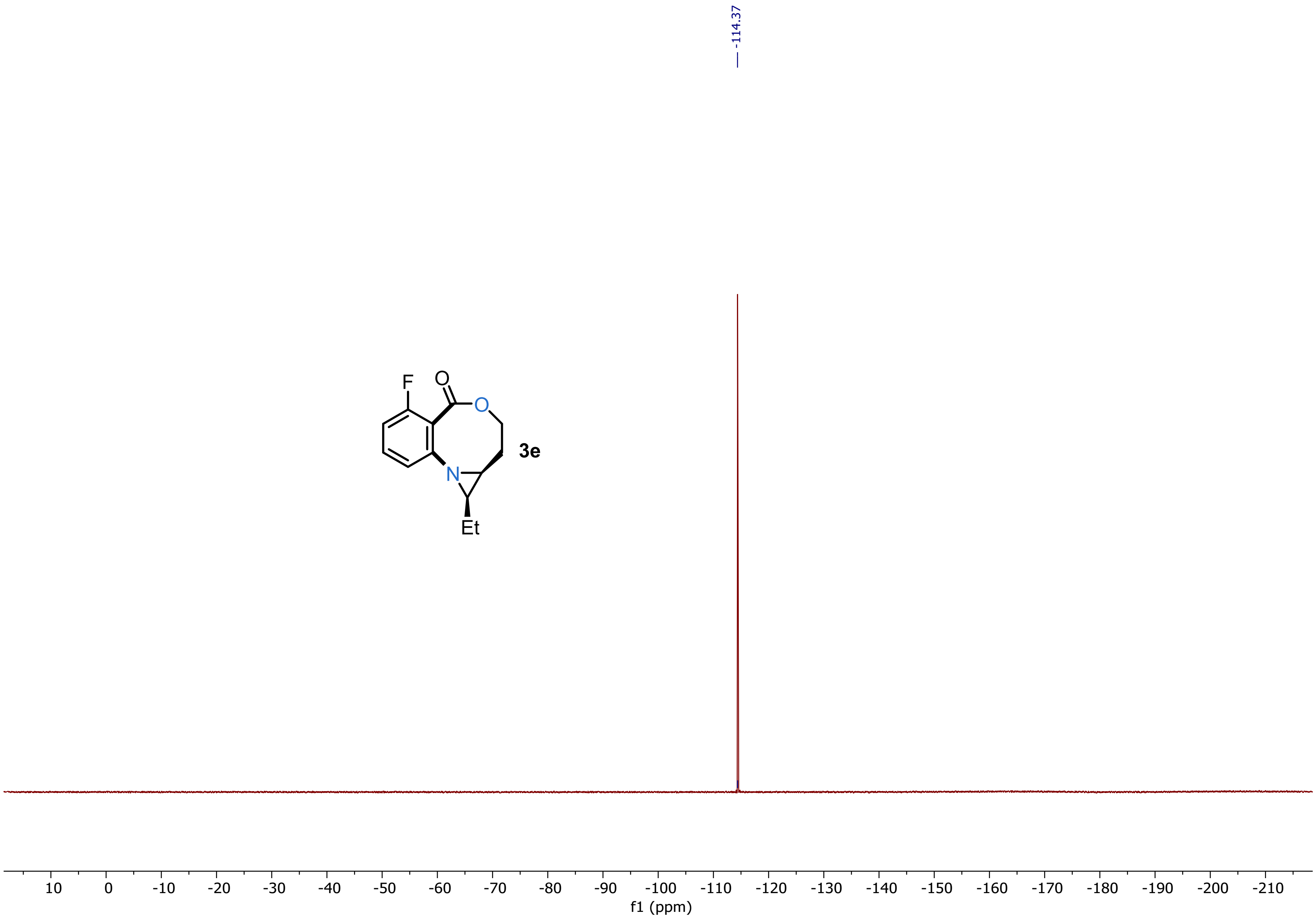

Figure S38. $^{19}F\{^{1}H\}$ NMR (376 MHz, $CDCl_3$) spectrum of **3e**.

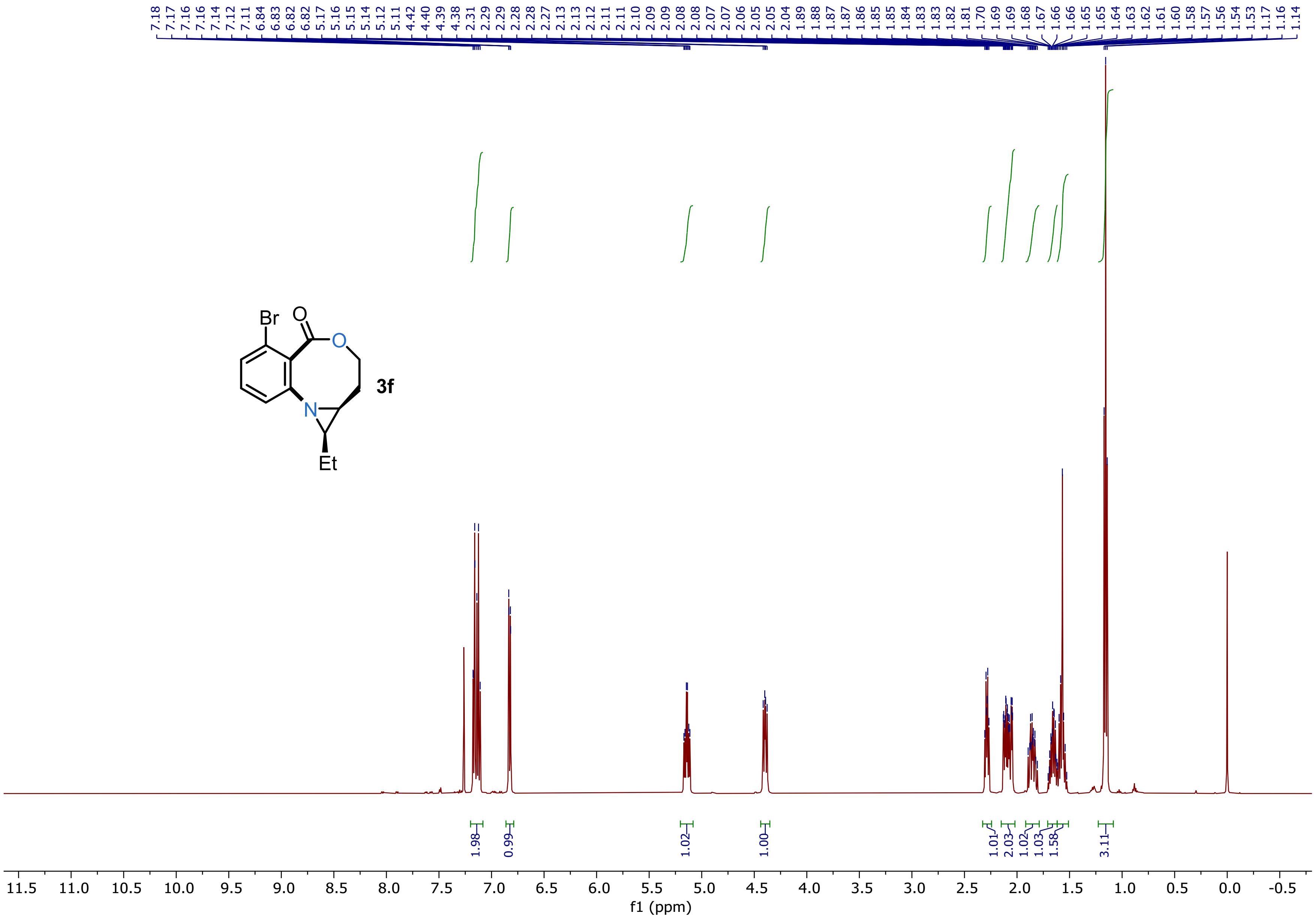

Figure S39. ^{1}H NMR (500 MHz, $CDCl_3$) spectrum of **3f**.

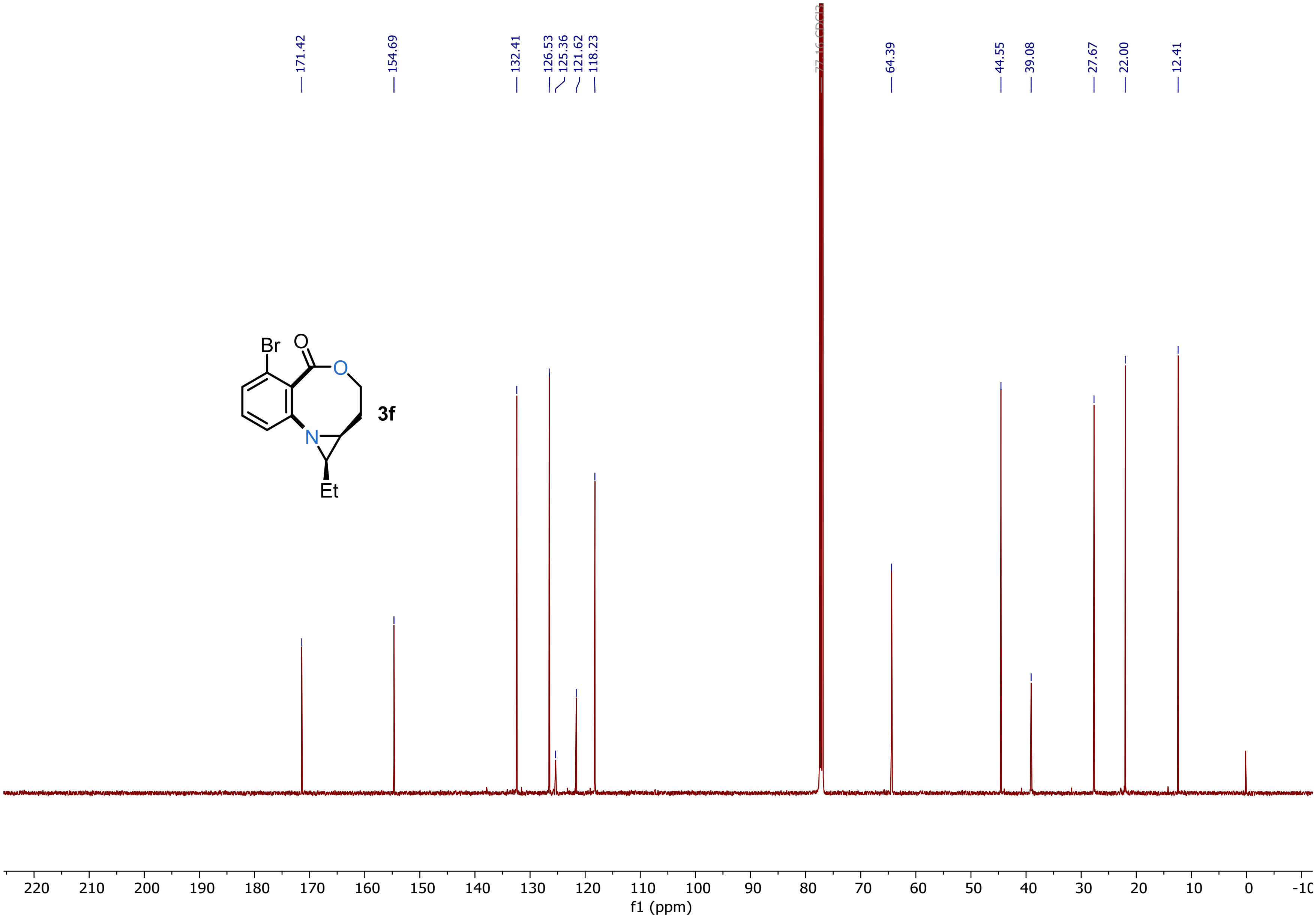

Figure S40. $^{13}C\{^1H\}$ NMR (126 MHz, $CDCl_3$) spectrum of **3f**.

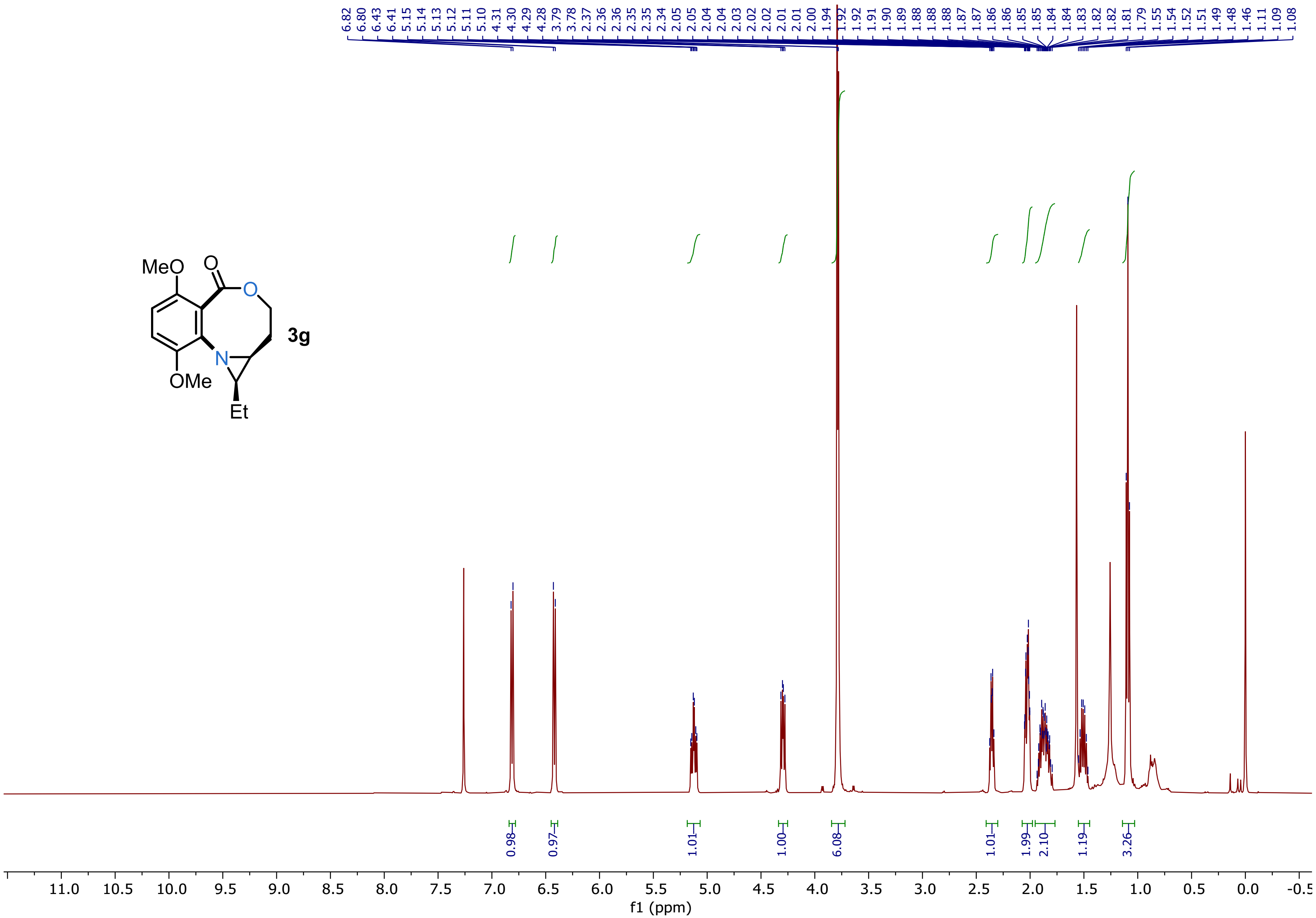

Figure S41. ^{1}H NMR (500 MHz, $CDCl_3$) spectrum of **3g**.

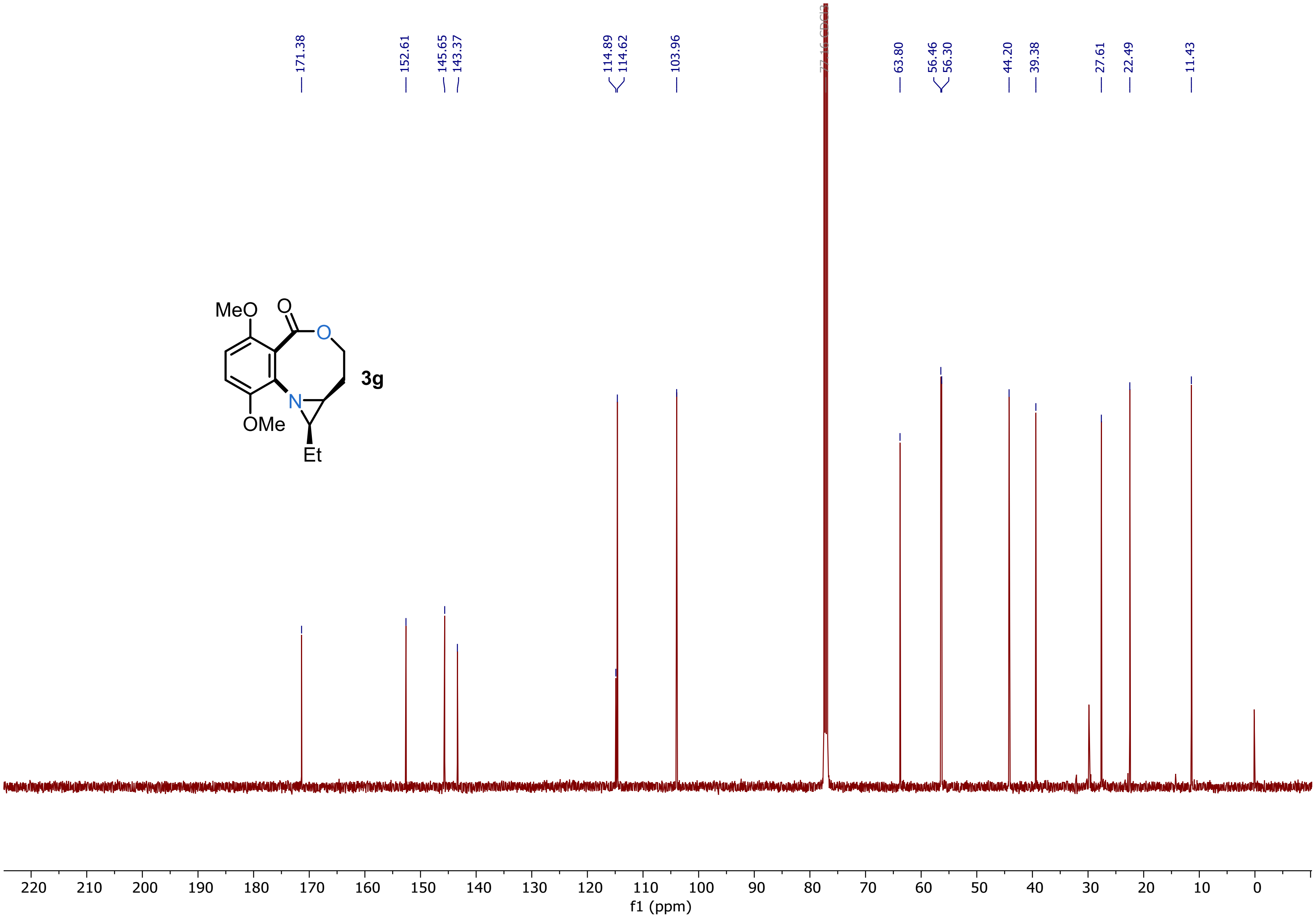

Figure S42. $^{13}C\{^1H\}$ NMR (126 MHz, $CDCl_3$) spectrum of **3g**.

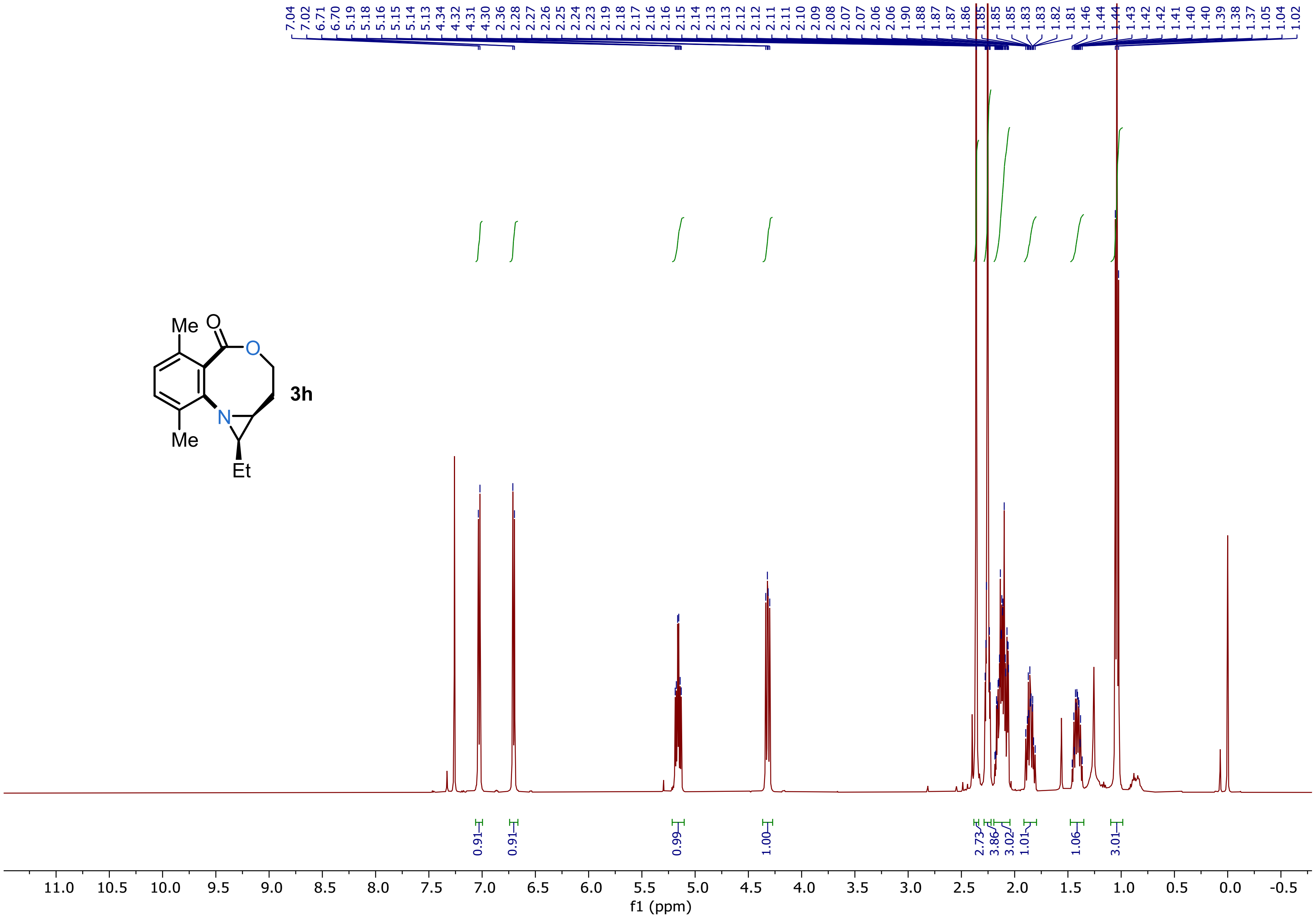

Figure S43. ^{1}H NMR (500 MHz, $CDCl_3$) spectrum of **3h**.

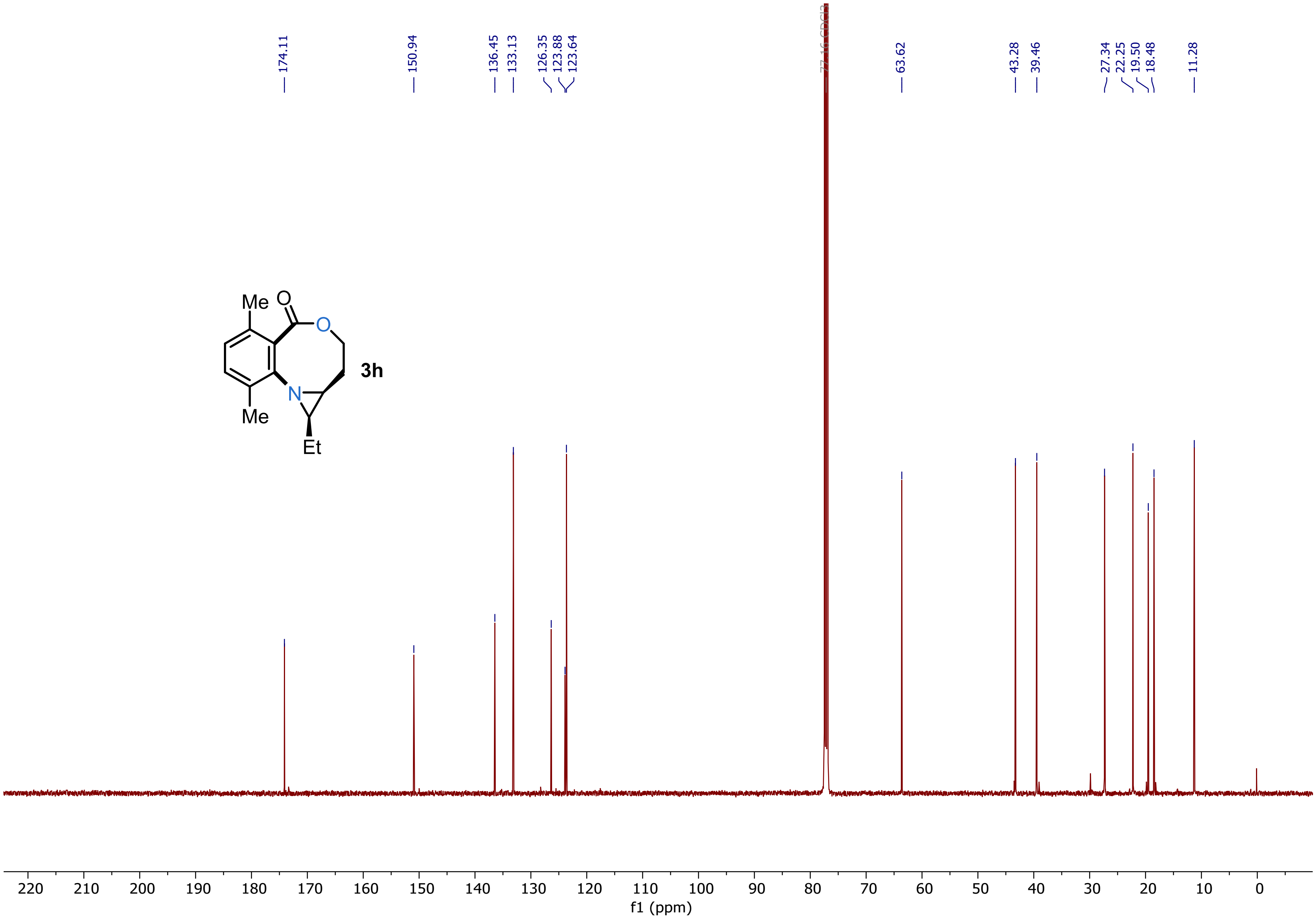

Figure S44. $^{13}C\{^{1}H\}$ NMR (126 MHz, $CDCl_3$) spectrum of **3h**.

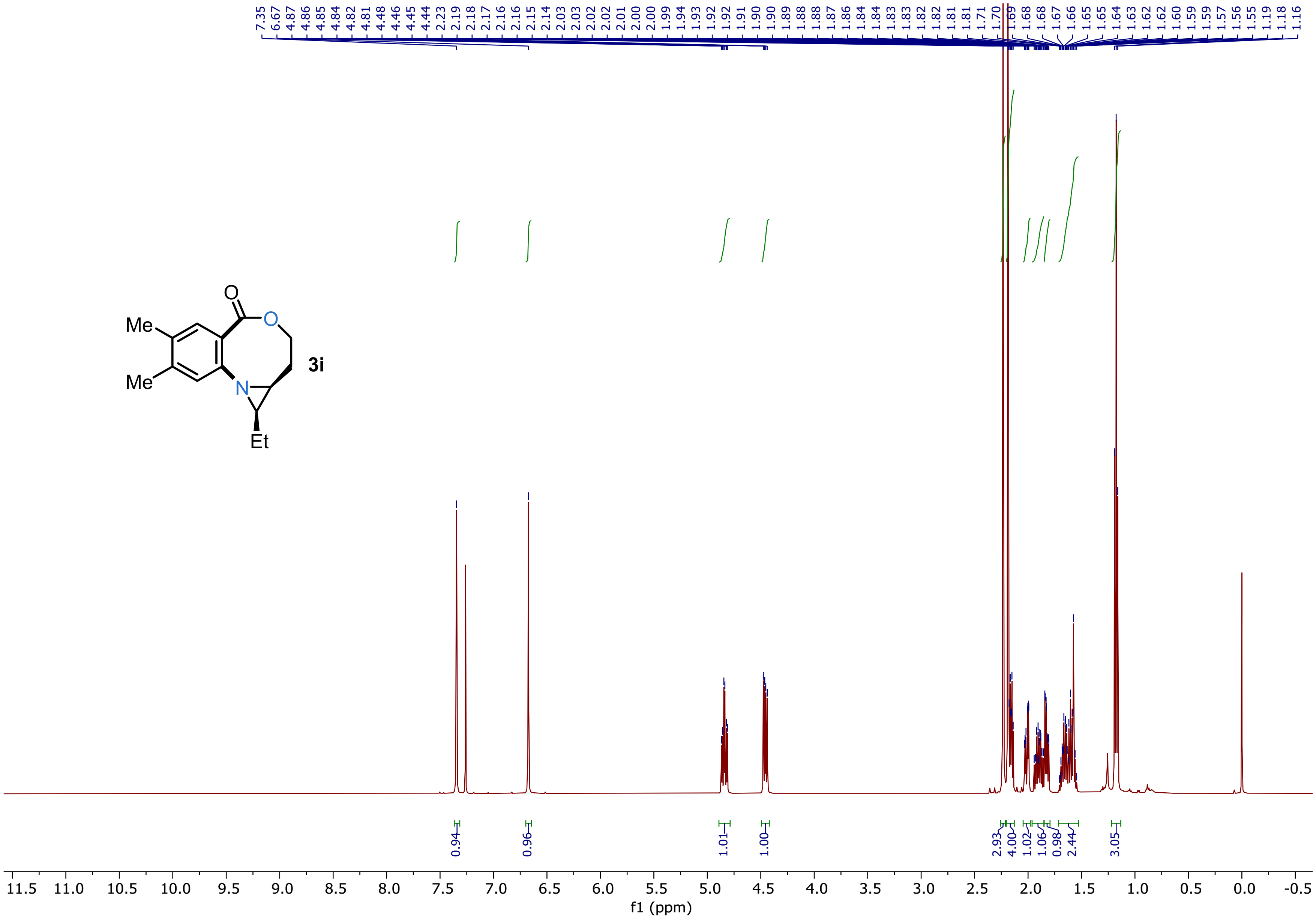

Figure S45. ^{1}H NMR (500 MHz, $CDCl_3$) spectrum of **3i**.

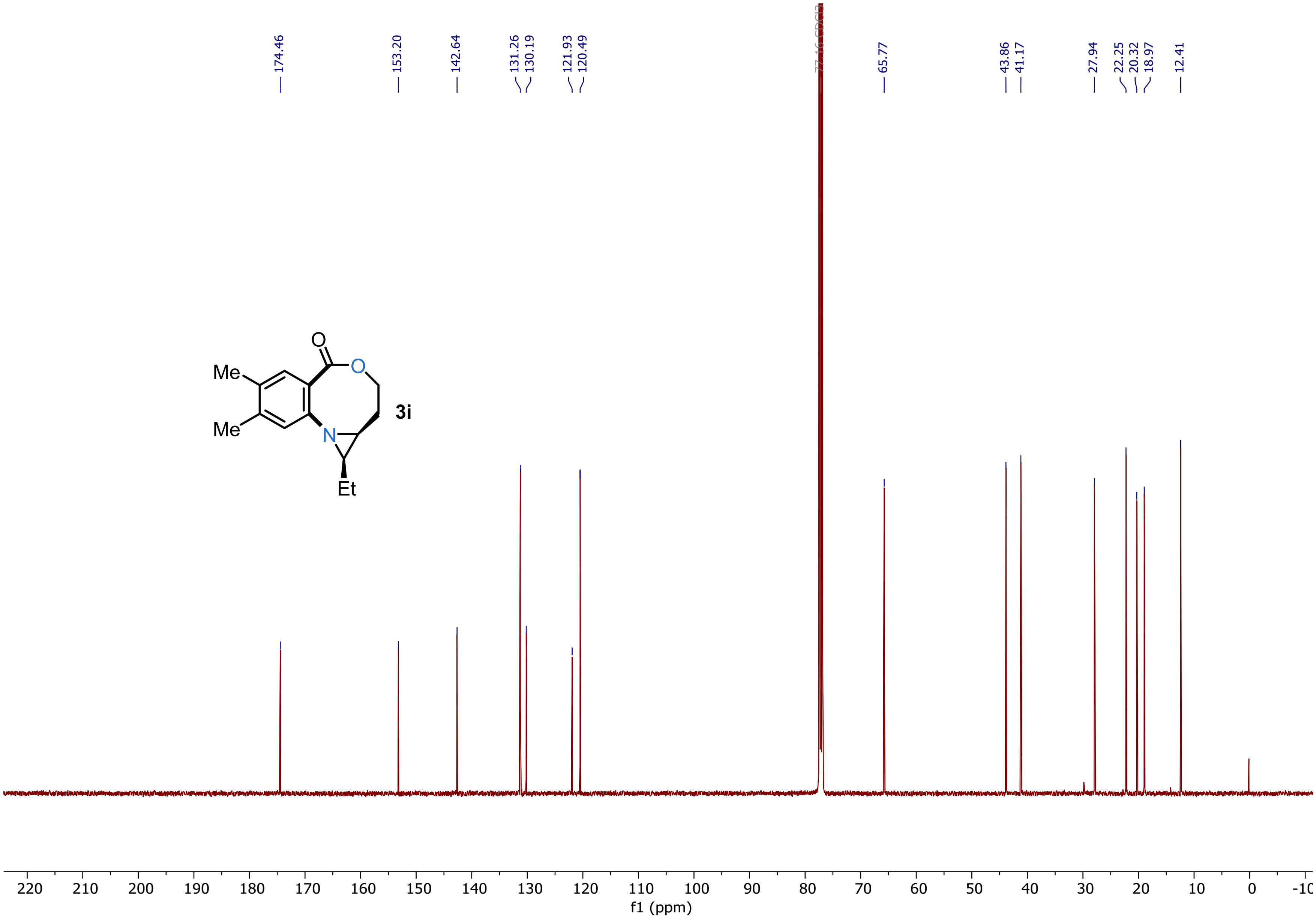

Figure S46. $^{13}C\{^{1}H\}$ NMR (126 MHz, $CDCl_3$) spectrum of **3i**.

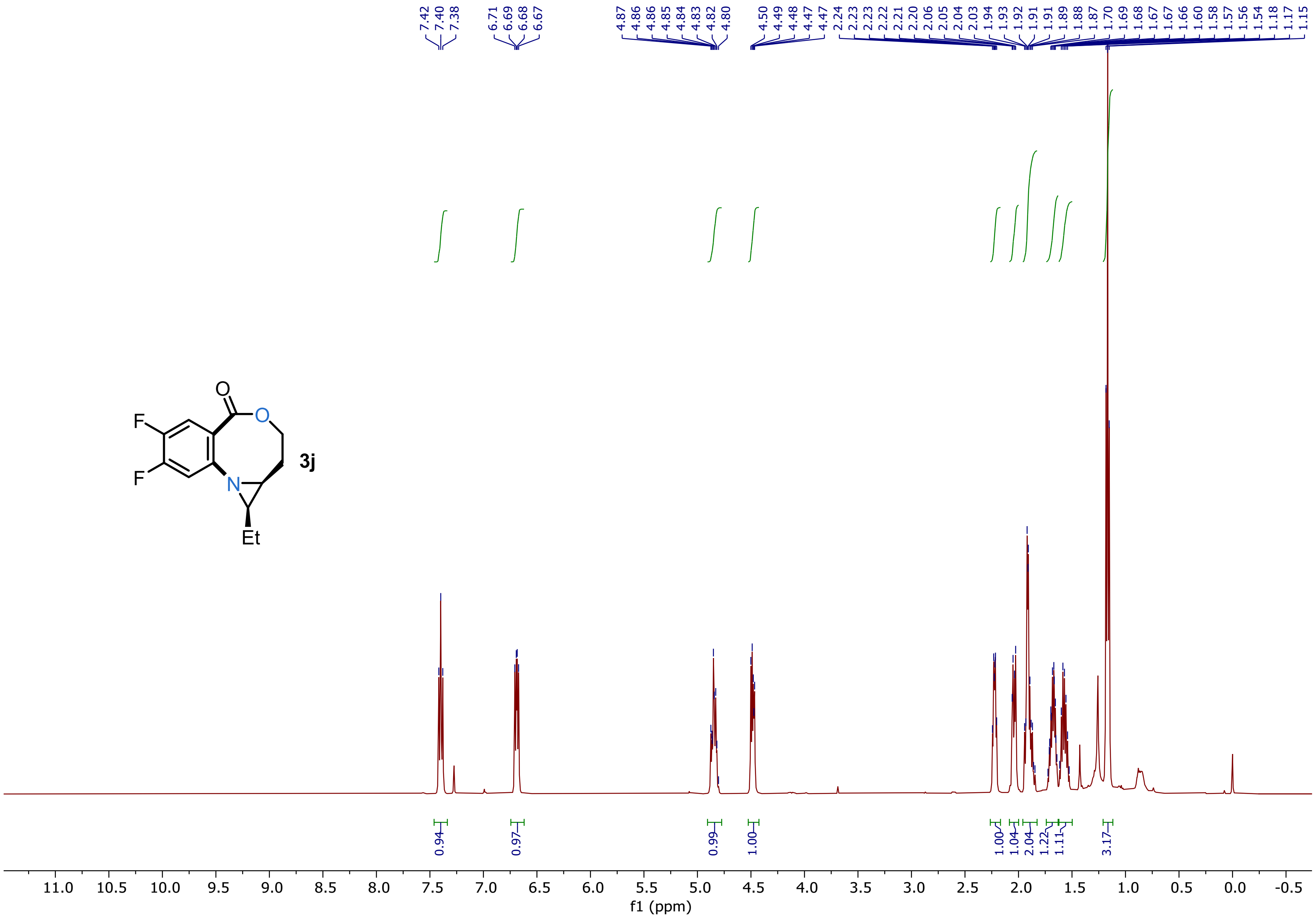

Figure S47. ^{1}H NMR (500 MHz, $CDCl_3$) spectrum of **3j**.

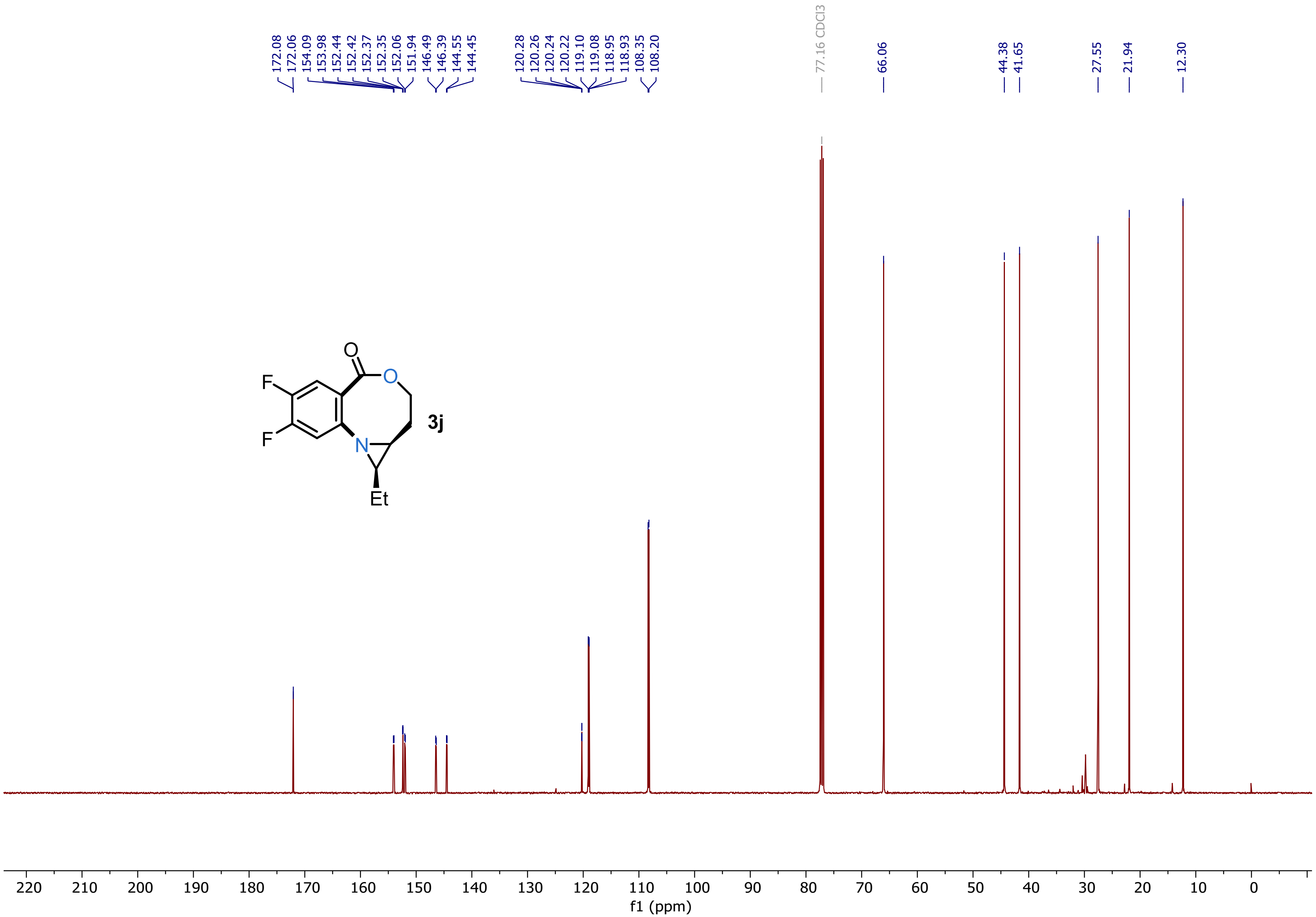

Figure S48. $^{13}C\{^{1}H\}$ NMR (126 MHz, $CDCl_3$) spectrum of **3j**.

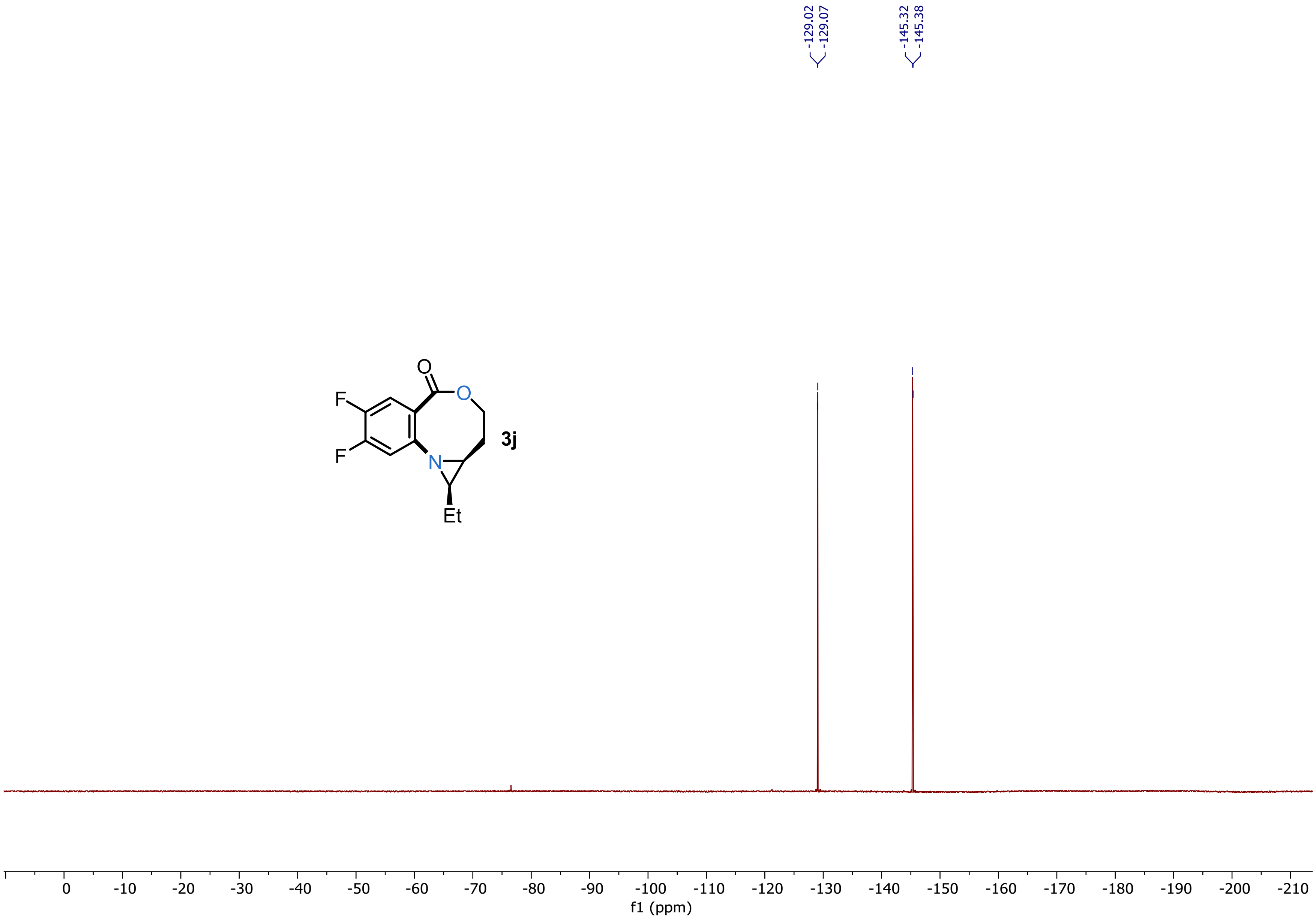

3j

Figure S49. $^{19}F\{^{1}H\}$ NMR (376 MHz, $CDCl_3$) spectrum of **3j**.

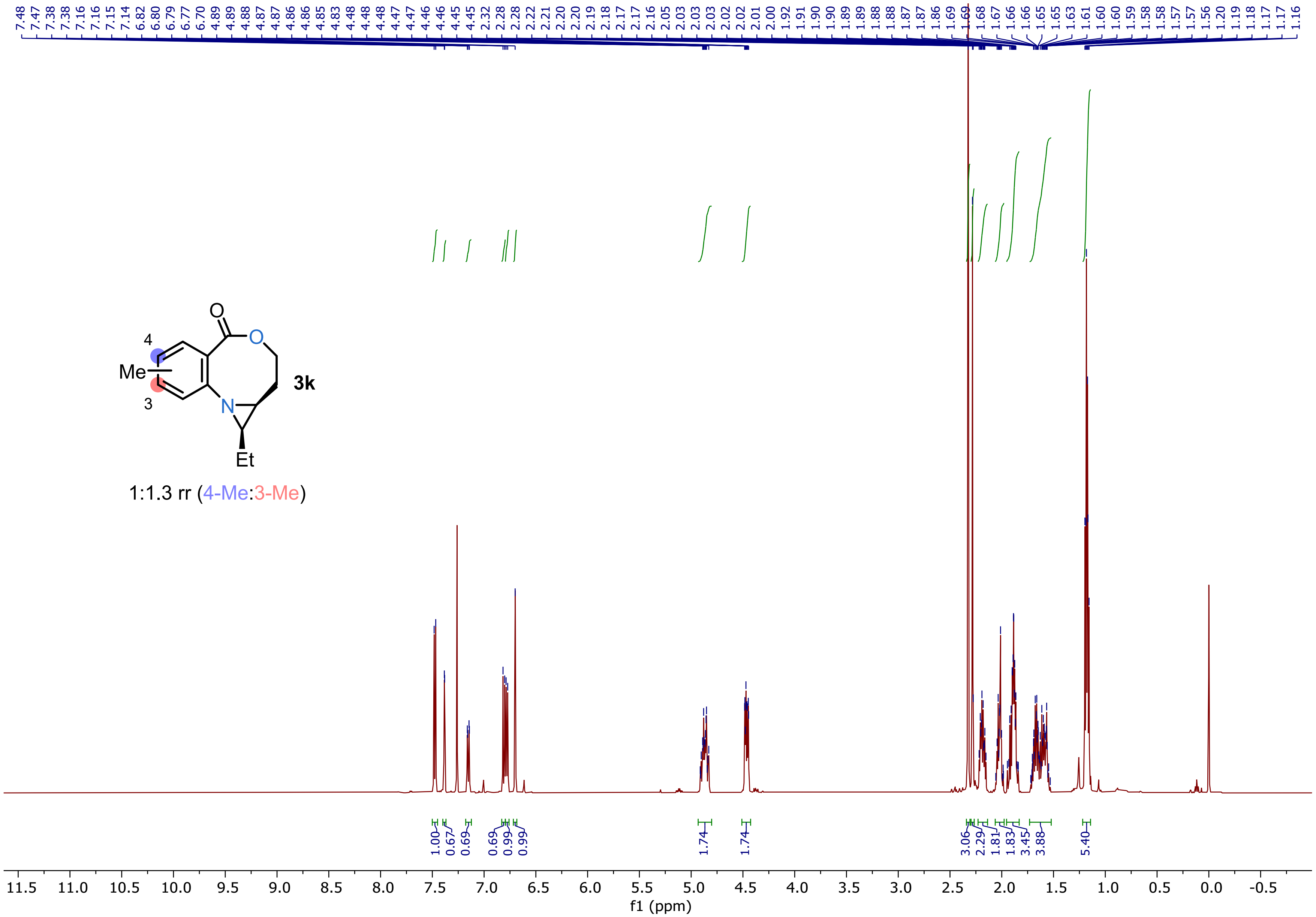

Figure S50. ^{1}H NMR (500 MHz, $CDCl_3$) spectrum of **3k**.

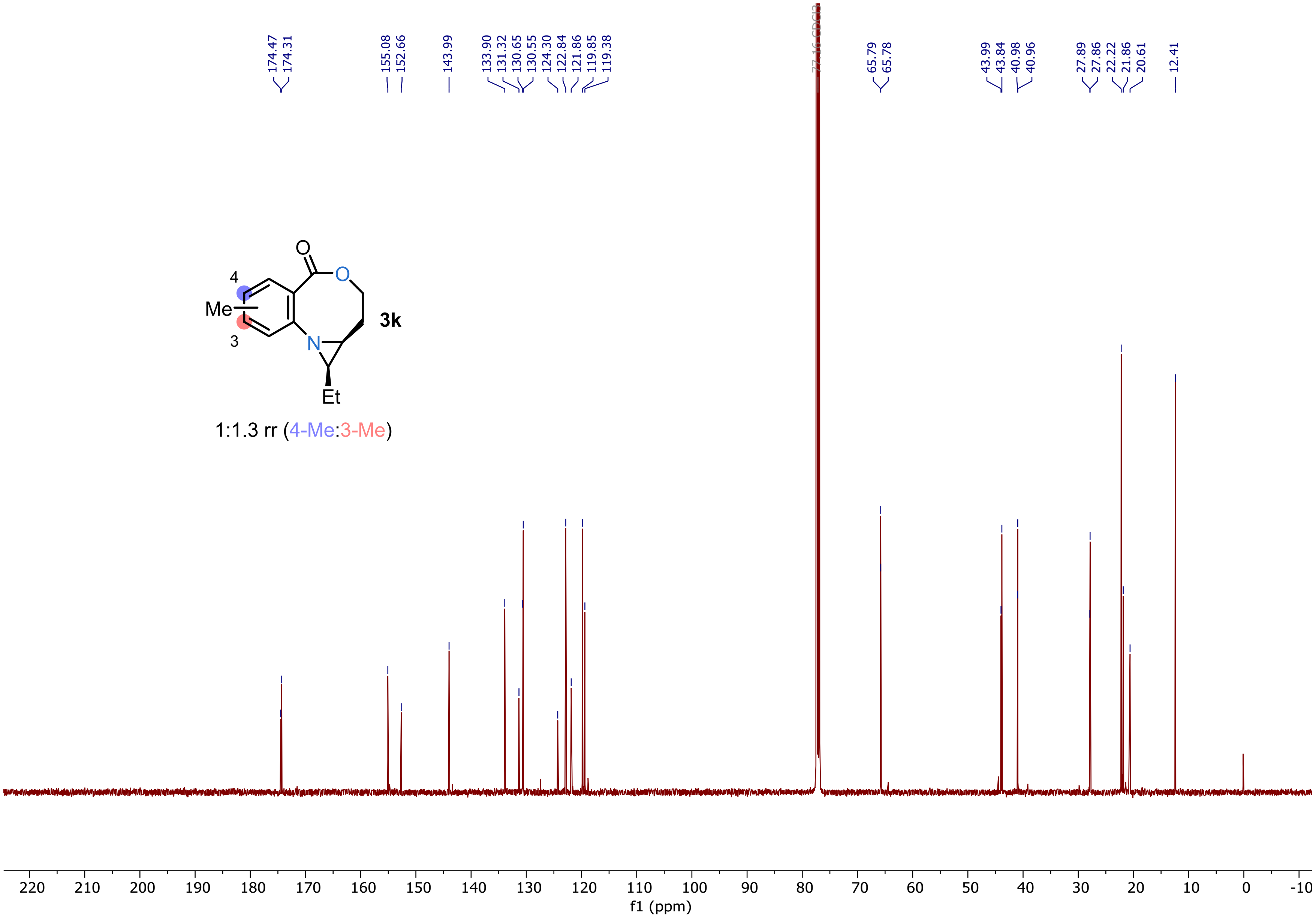

Figure S51. $^{13}C\{^1H\}$ NMR (126 MHz, $CDCl_3$) spectrum of **3k**.

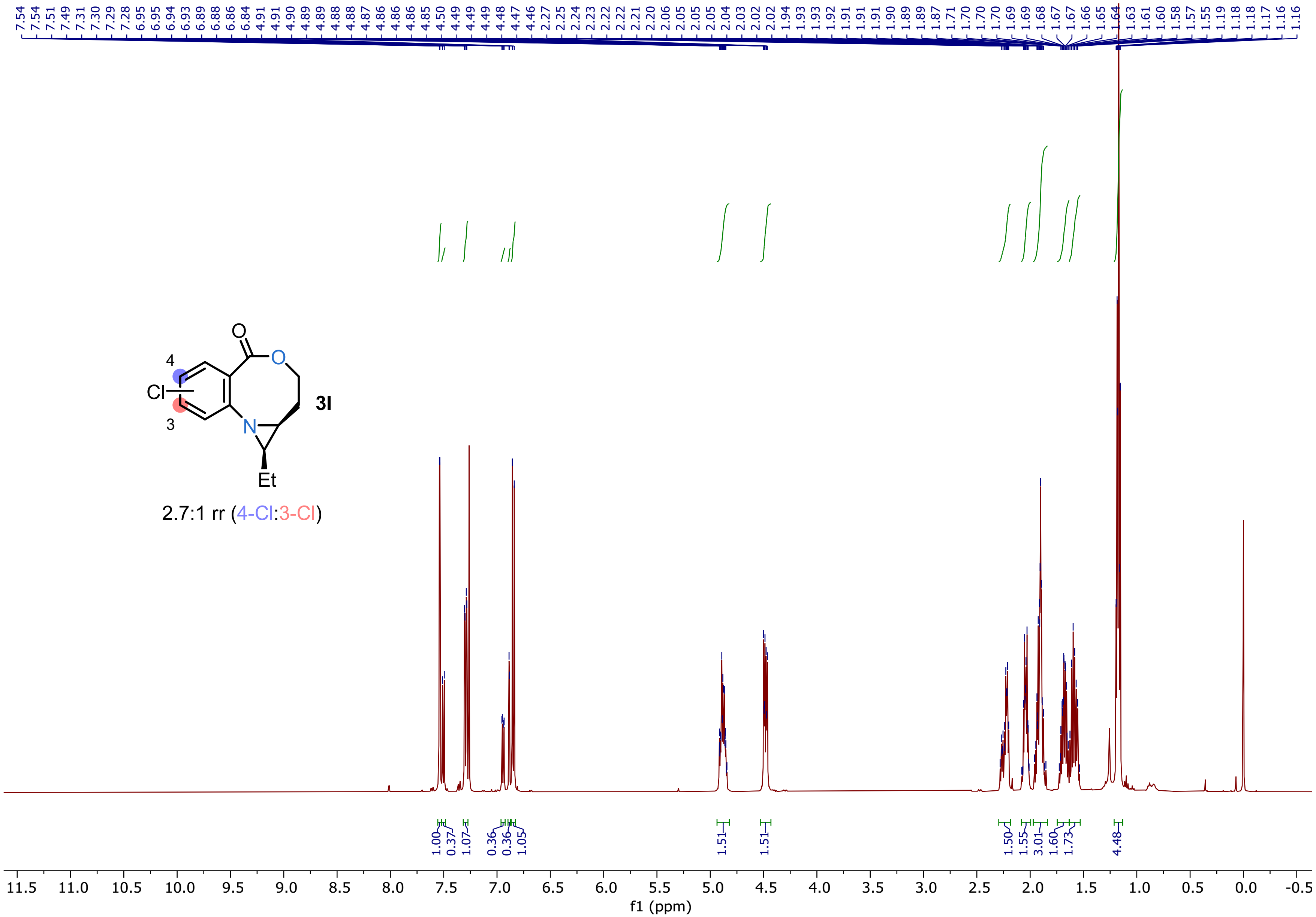

Figure S52. ^{1}H NMR (500 MHz, $CDCl_3$) spectrum of **3l**.

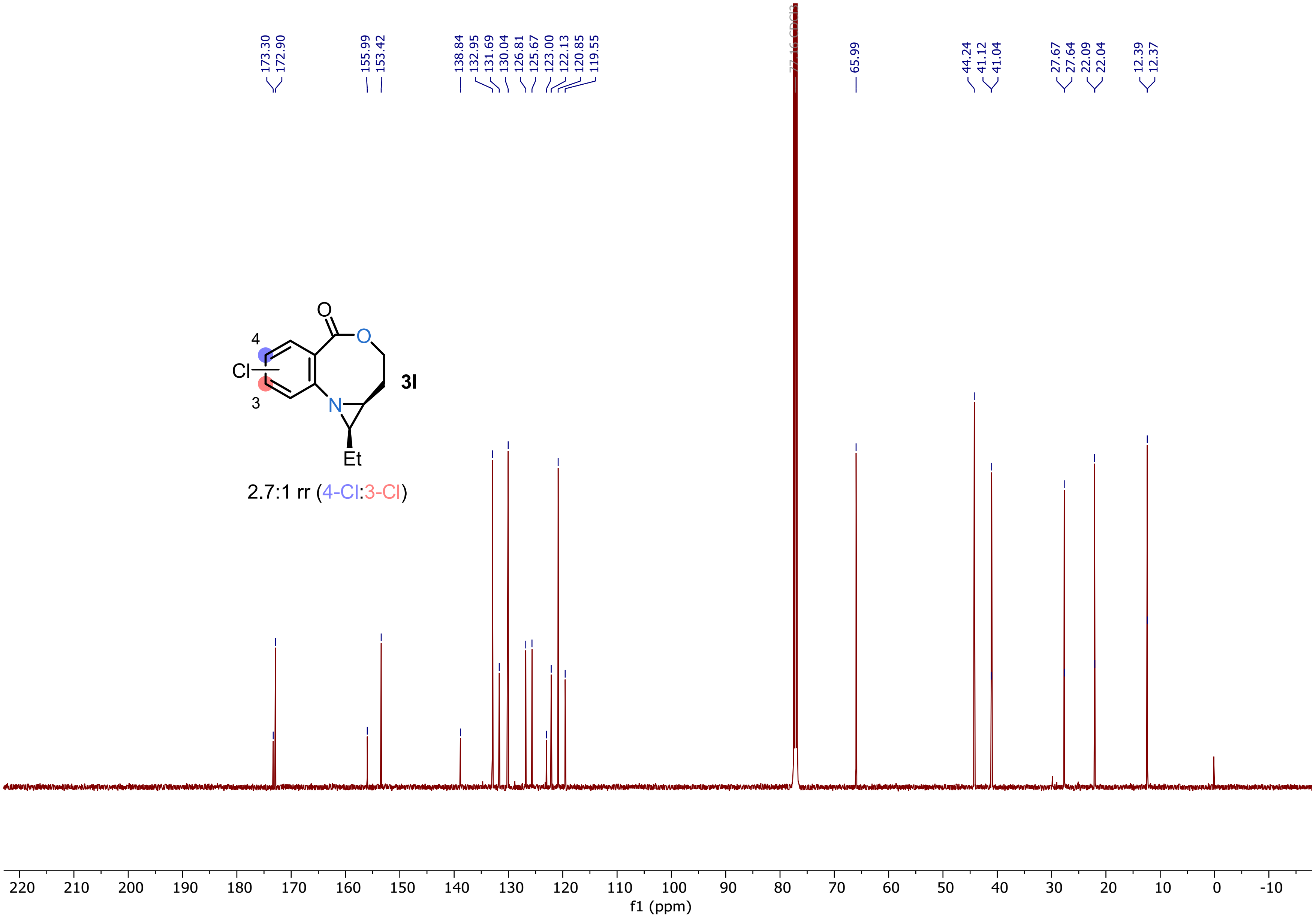

Figure S53. $^{13}C\{^{1}H\}$ NMR (126 MHz, $CDCl_3$) spectrum of **3l**.

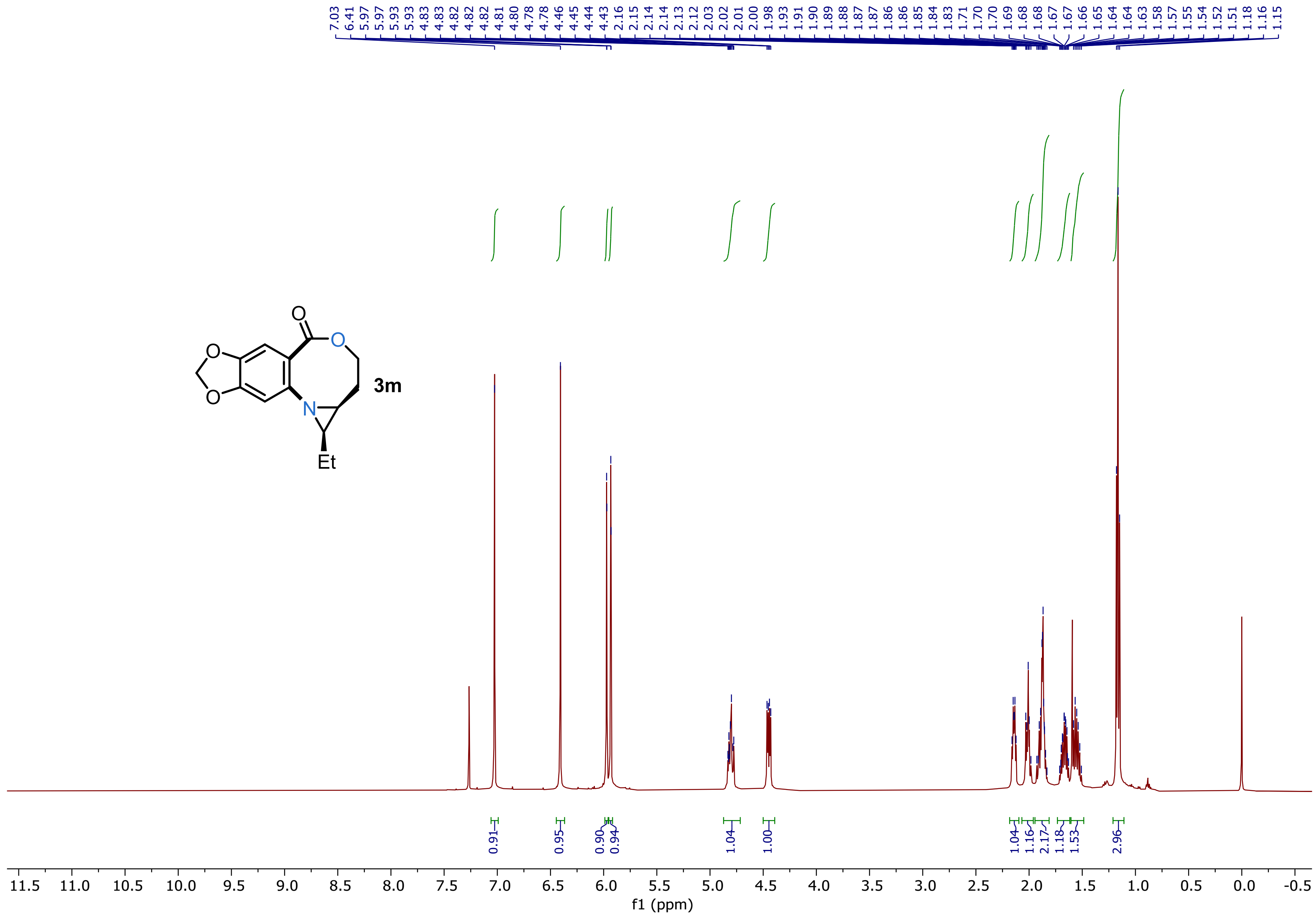

Figure S54. ^{1}H NMR (500 MHz, $CDCl_3$) spectrum of **3m**.

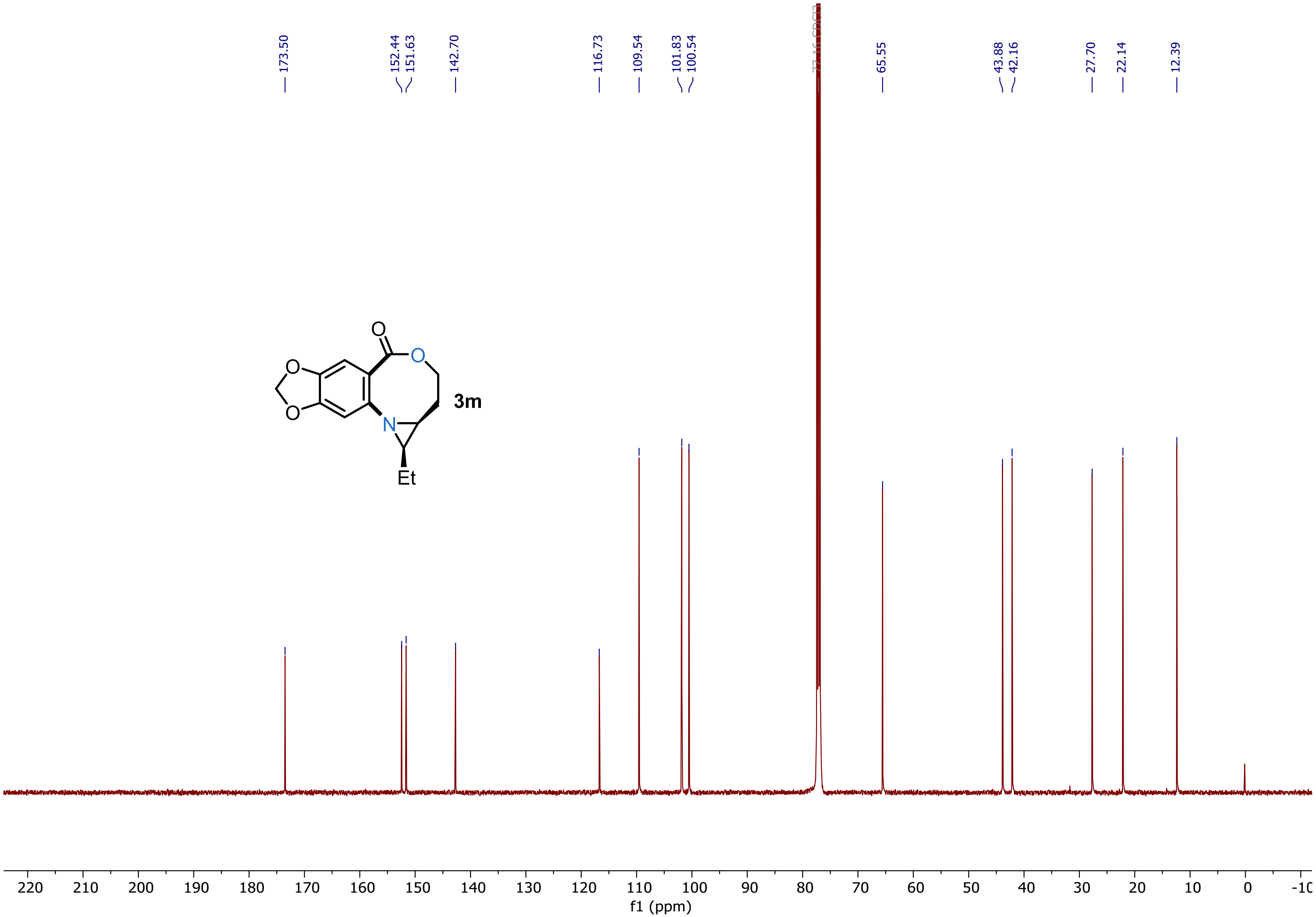

Figure S55. $^{13}C\{^1H\}$ NMR (126 MHz, $CDCl_3$) spectrum of **3m**.

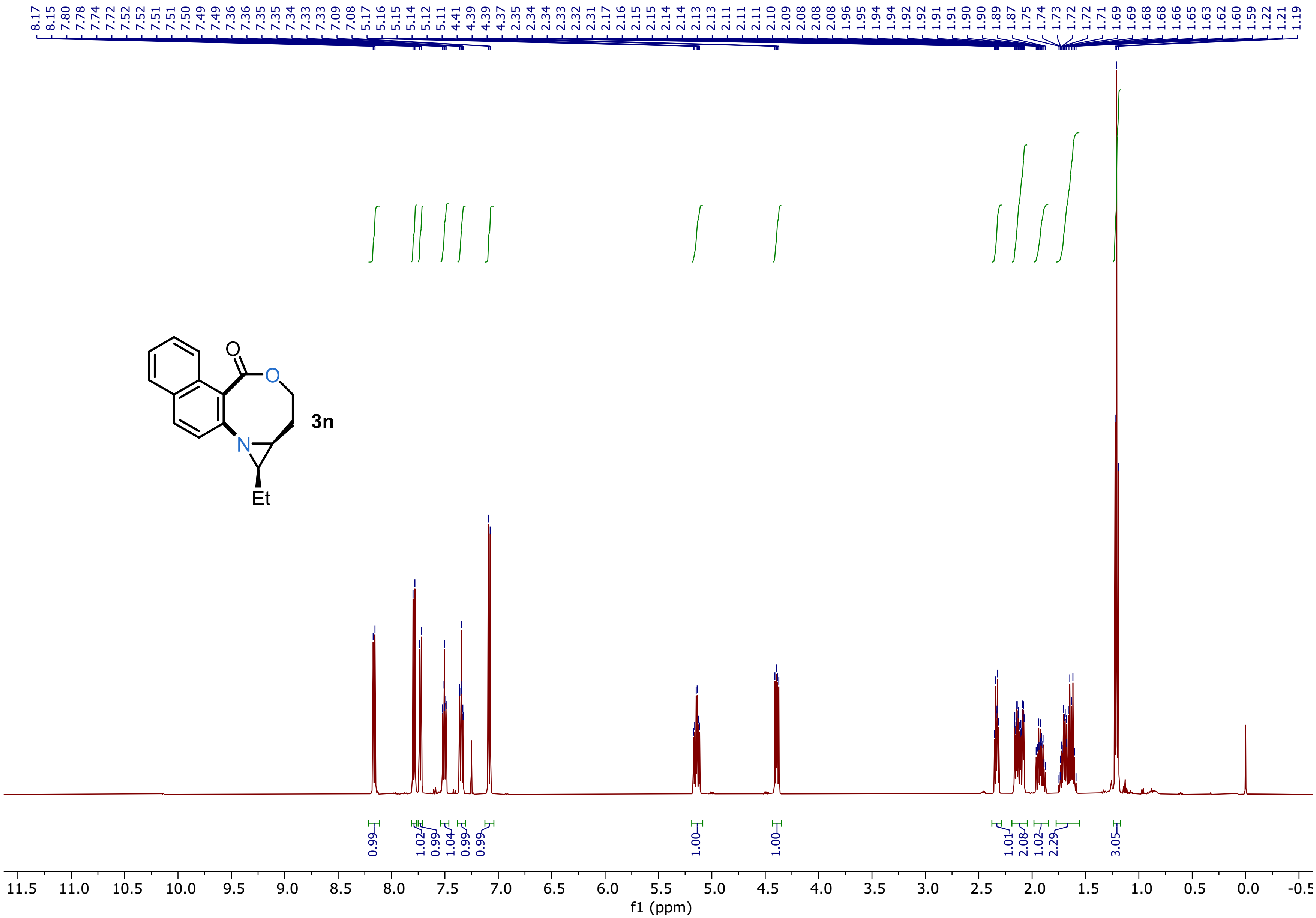

Figure S56. ^{1}H NMR (500 MHz, $CDCl_3$) spectrum of **3n**.

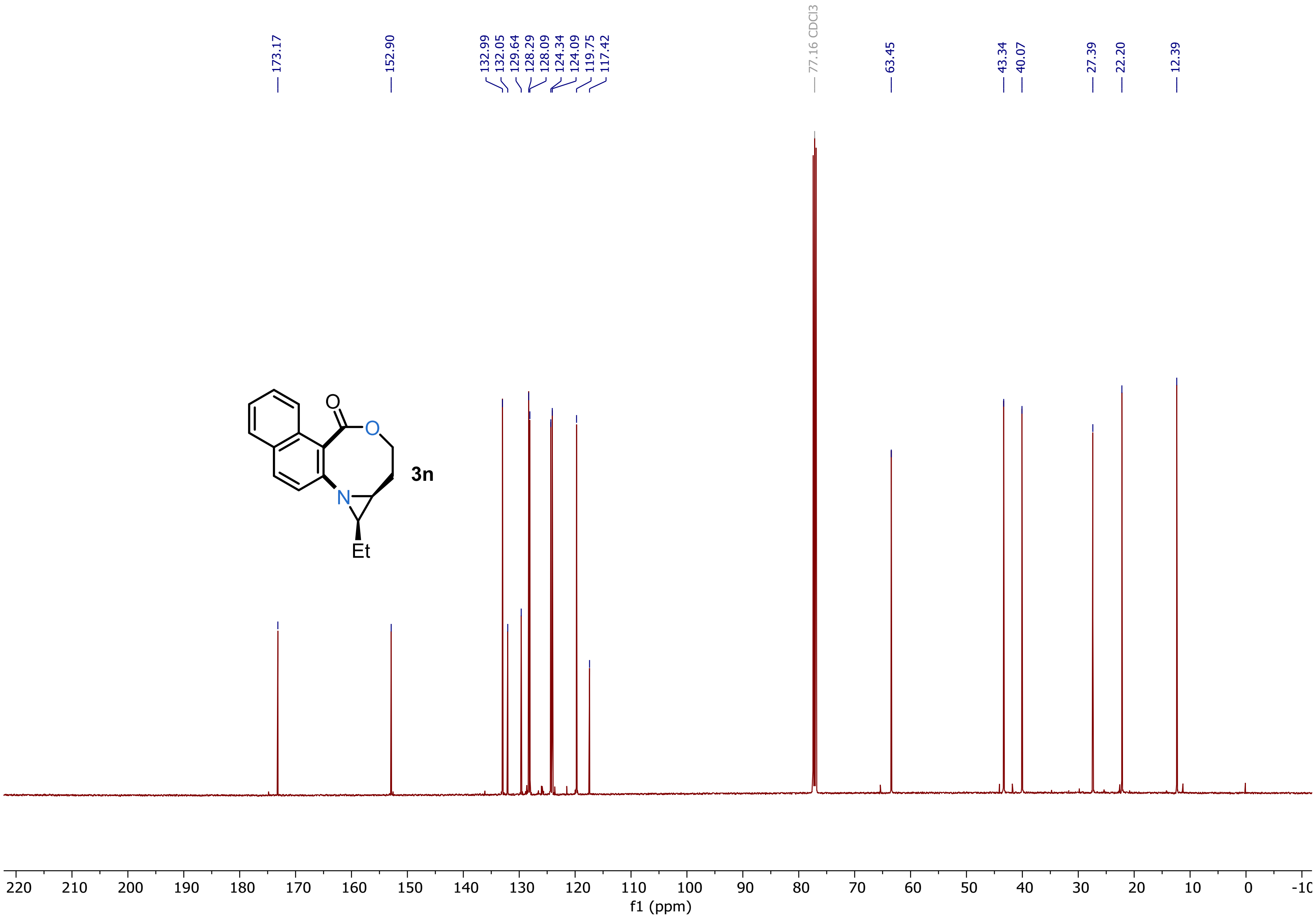

Figure S57. $^{13}C\{^{1}H\}$ NMR (126 MHz, $CDCl_3$) spectrum of **3n**.

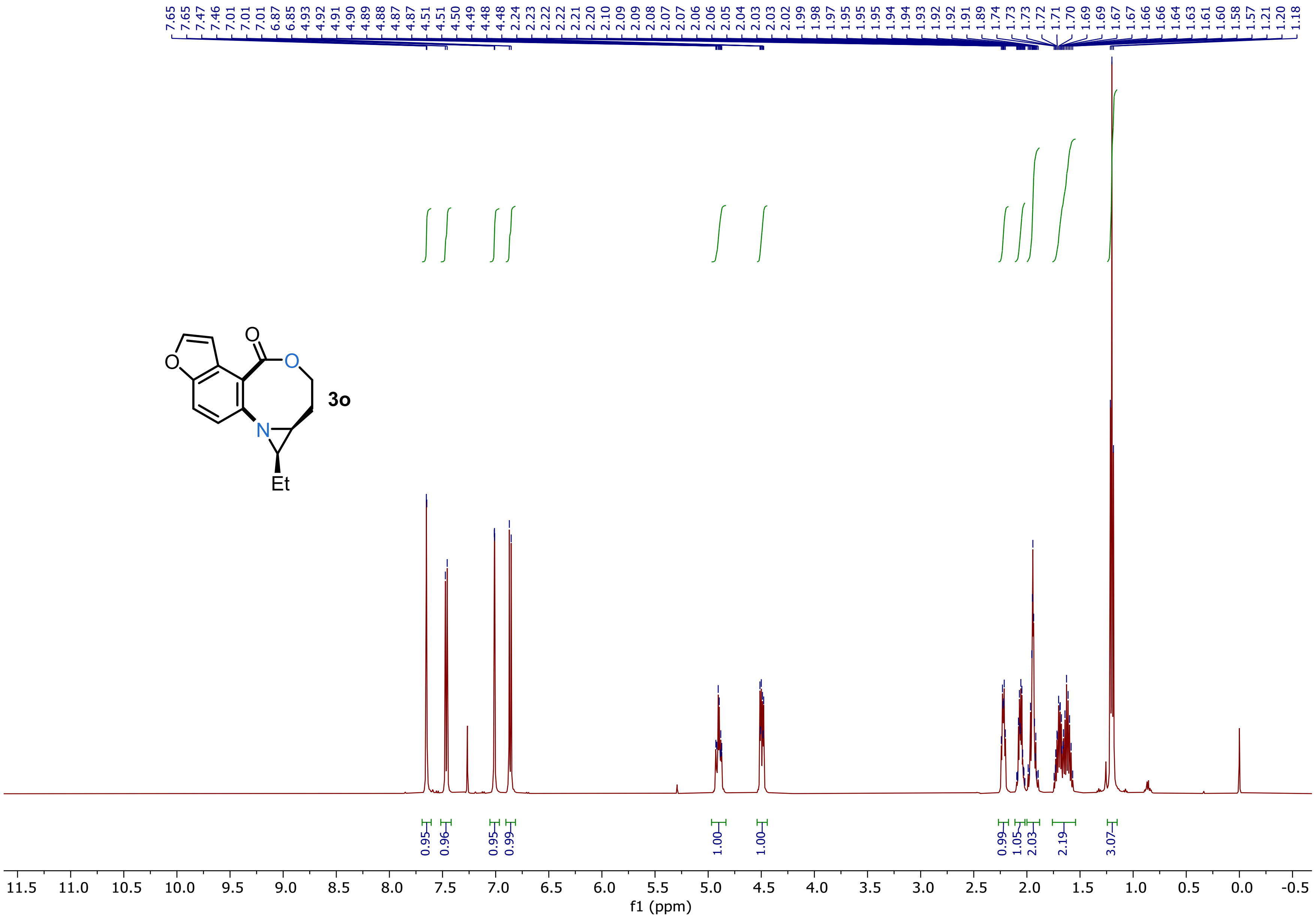

Figure S58. ^{1}H NMR (500 MHz, $CDCl_3$) spectrum of **3o**.

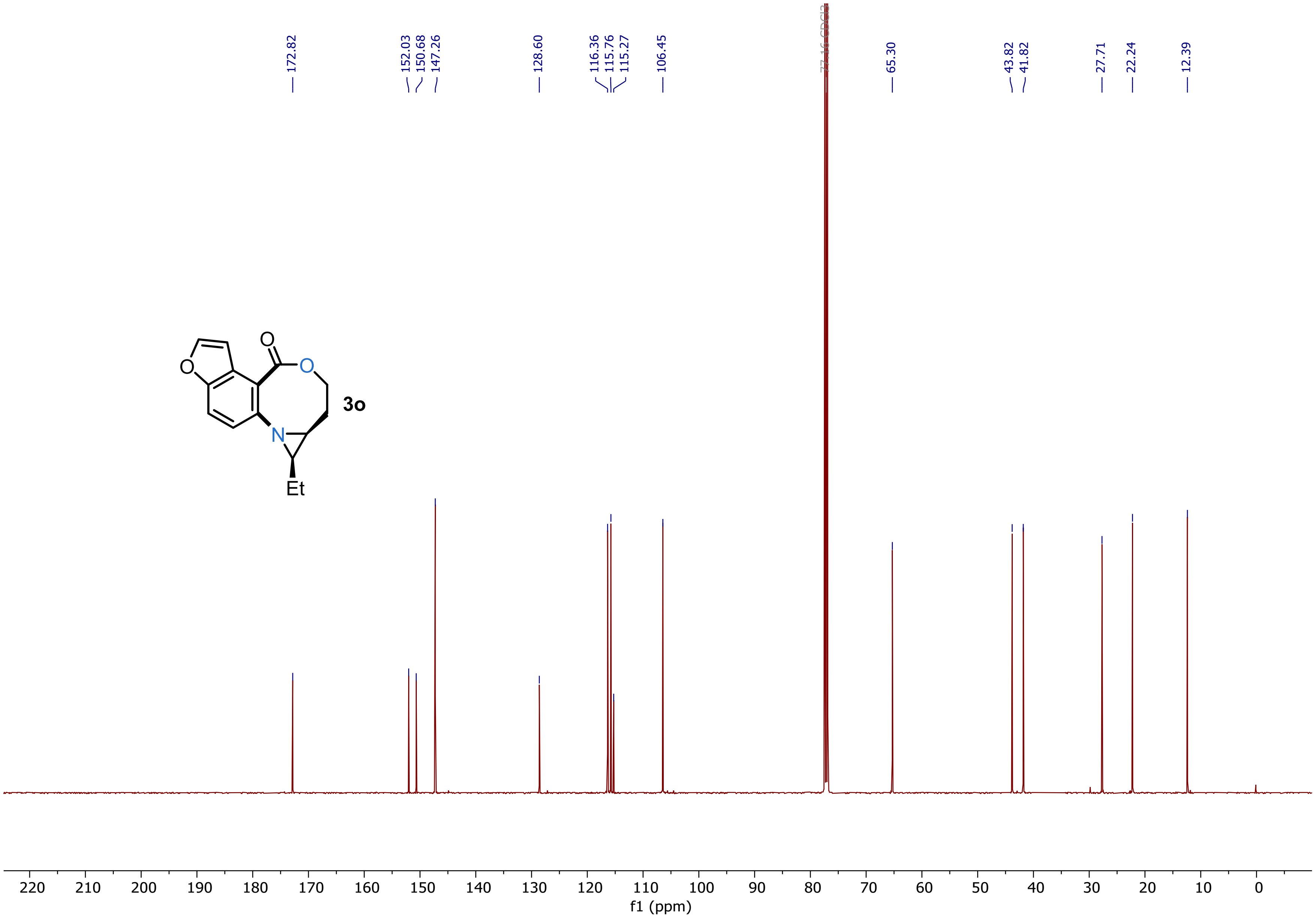

Figure S59. $^{13}C\{^{1}H\}$ NMR (126 MHz, $CDCl_3$) spectrum of **3o**.

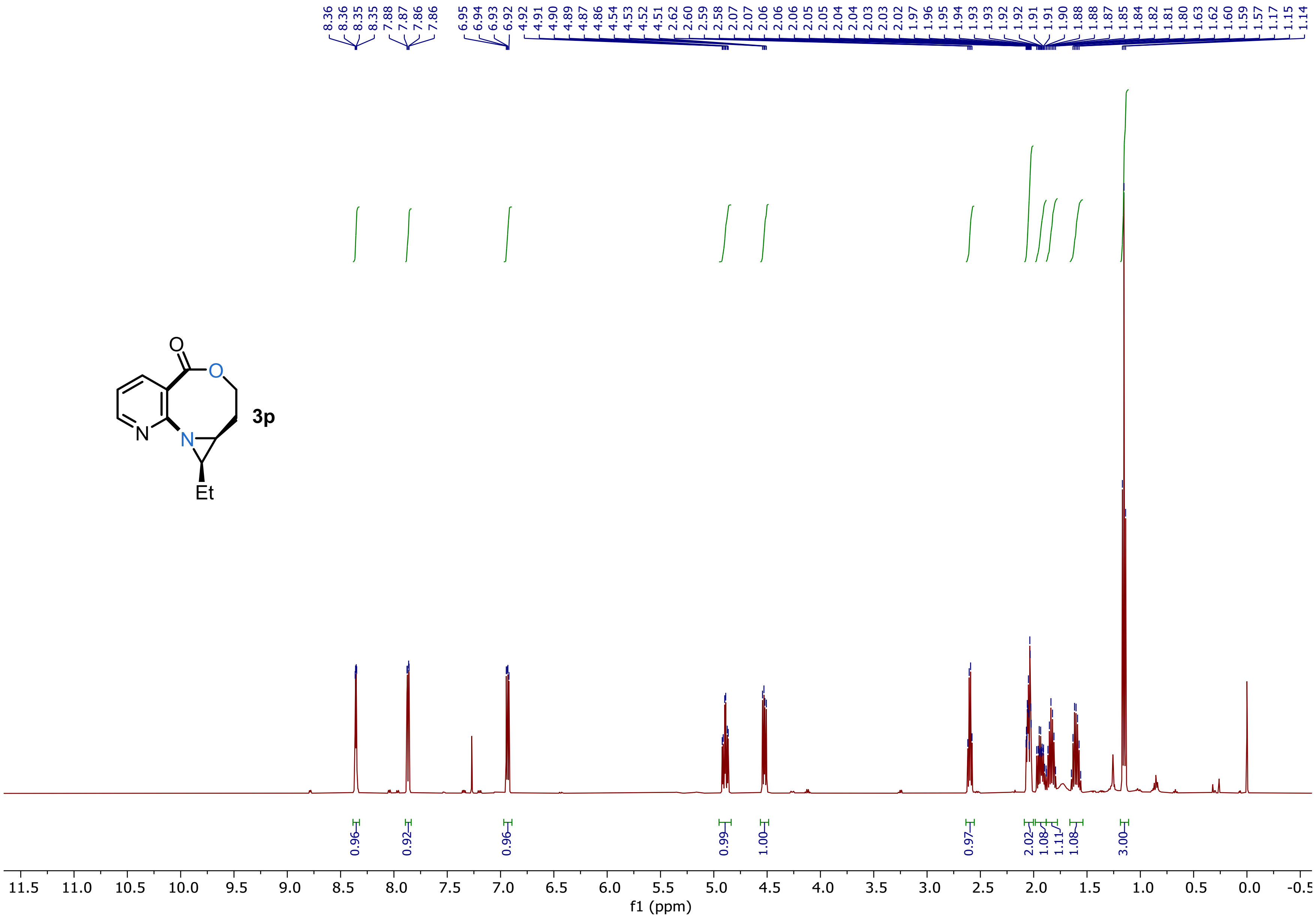

Figure S60. ^{1}H NMR (500 MHz, $CDCl_3$) spectrum of **3p**.

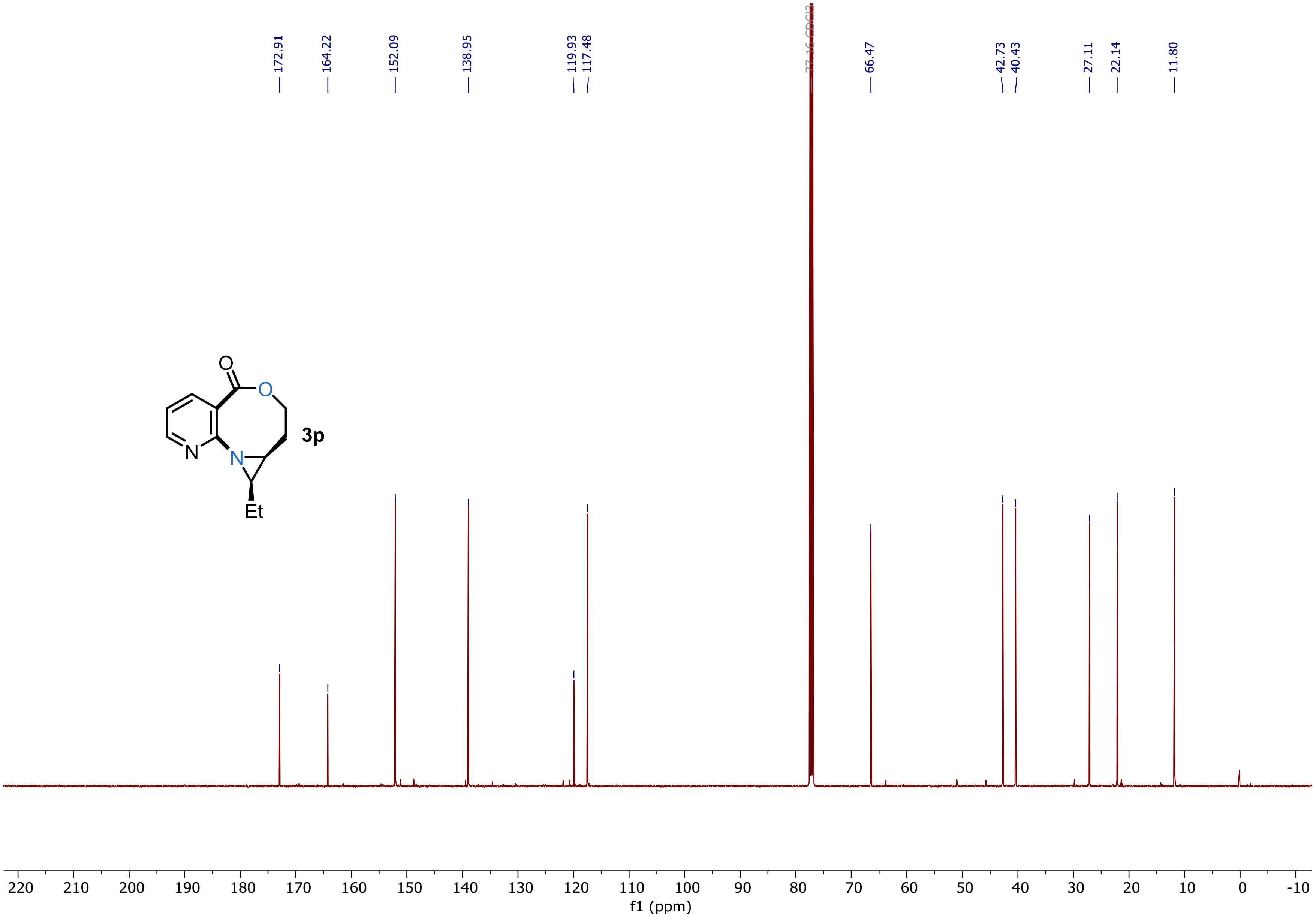

Figure S61. $^{13}C\{^{1}H\}$ NMR (126 MHz, $CDCl_3$) spectrum of **3p**.

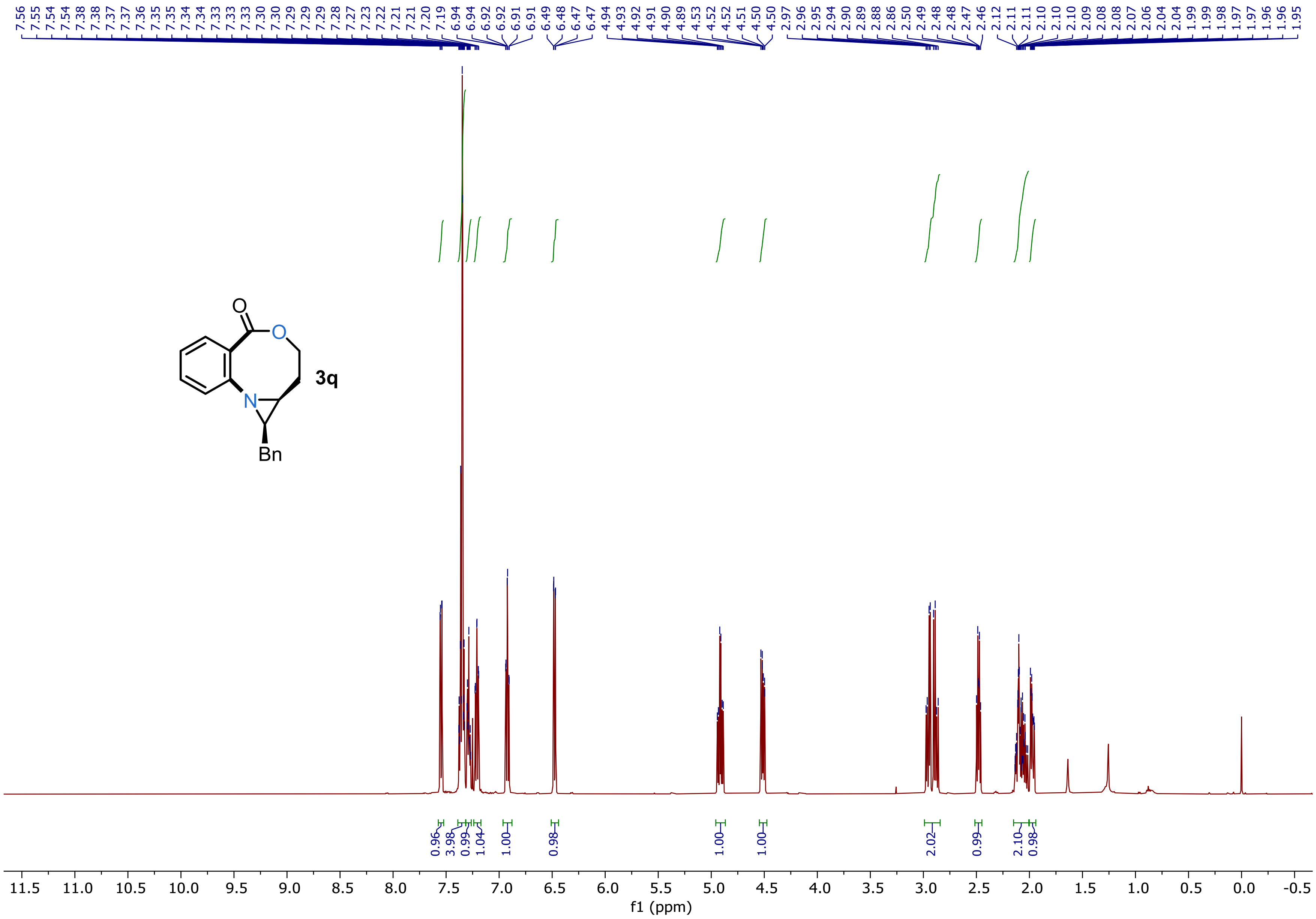

Figure S62. ^{1}H NMR (500 MHz, $CDCl_3$) spectrum of **3q**.

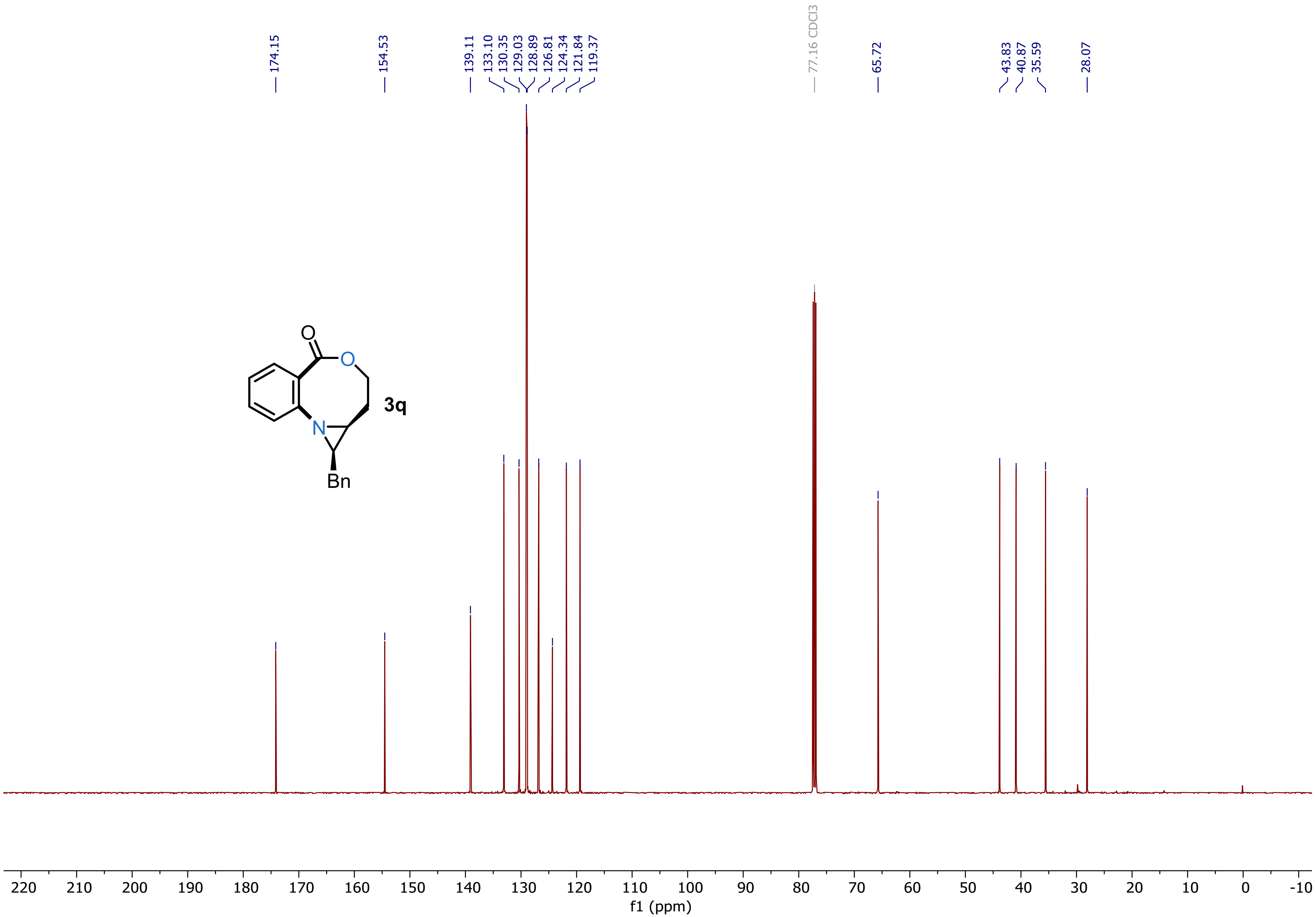

Figure S63. $^{13}C\{^{1}H\}$ NMR (126 MHz, $CDCl_3$) spectrum of **3q**.

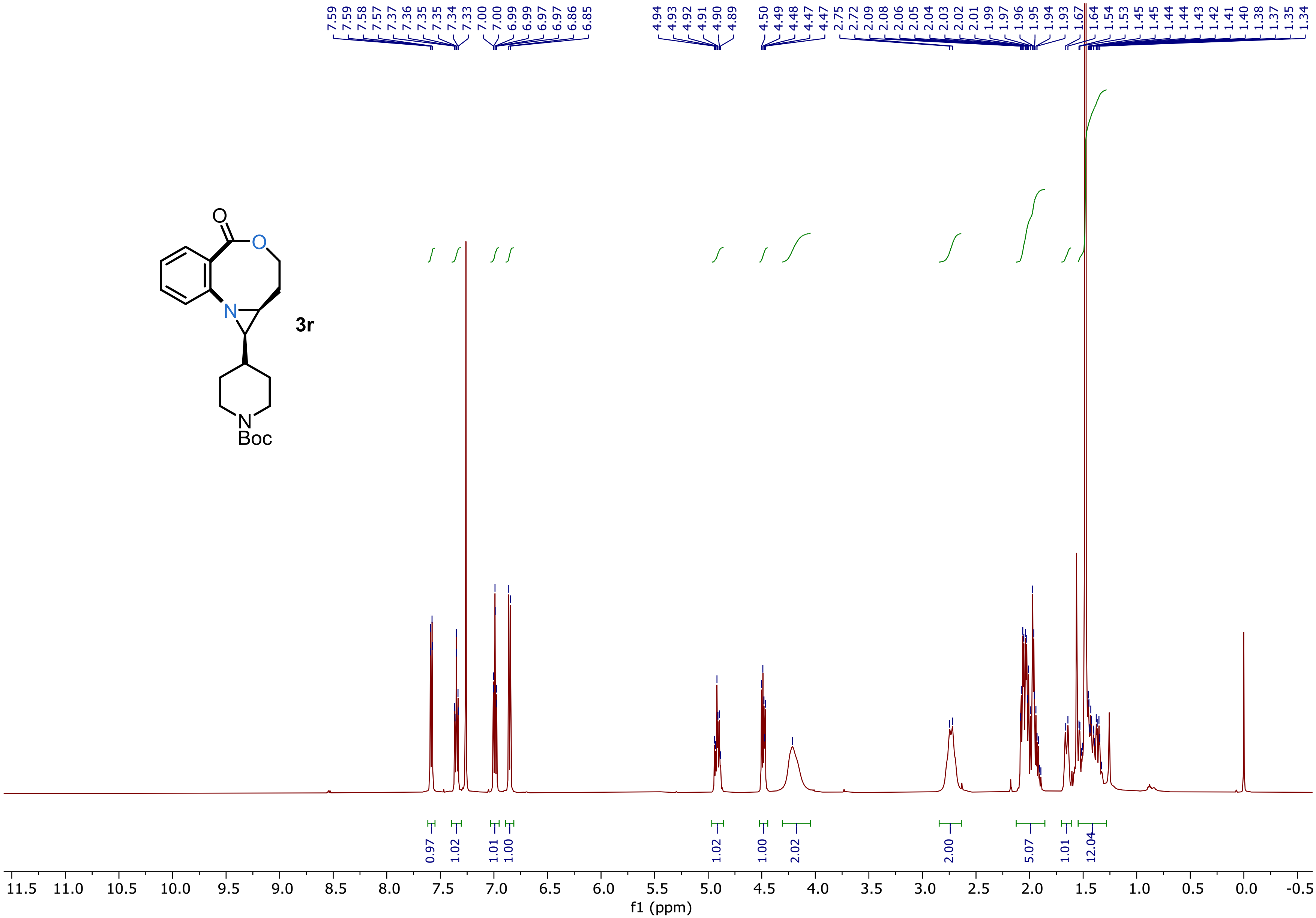

Figure S64. ^{1}H NMR (500 MHz, $CDCl_3$) spectrum of **3r**.

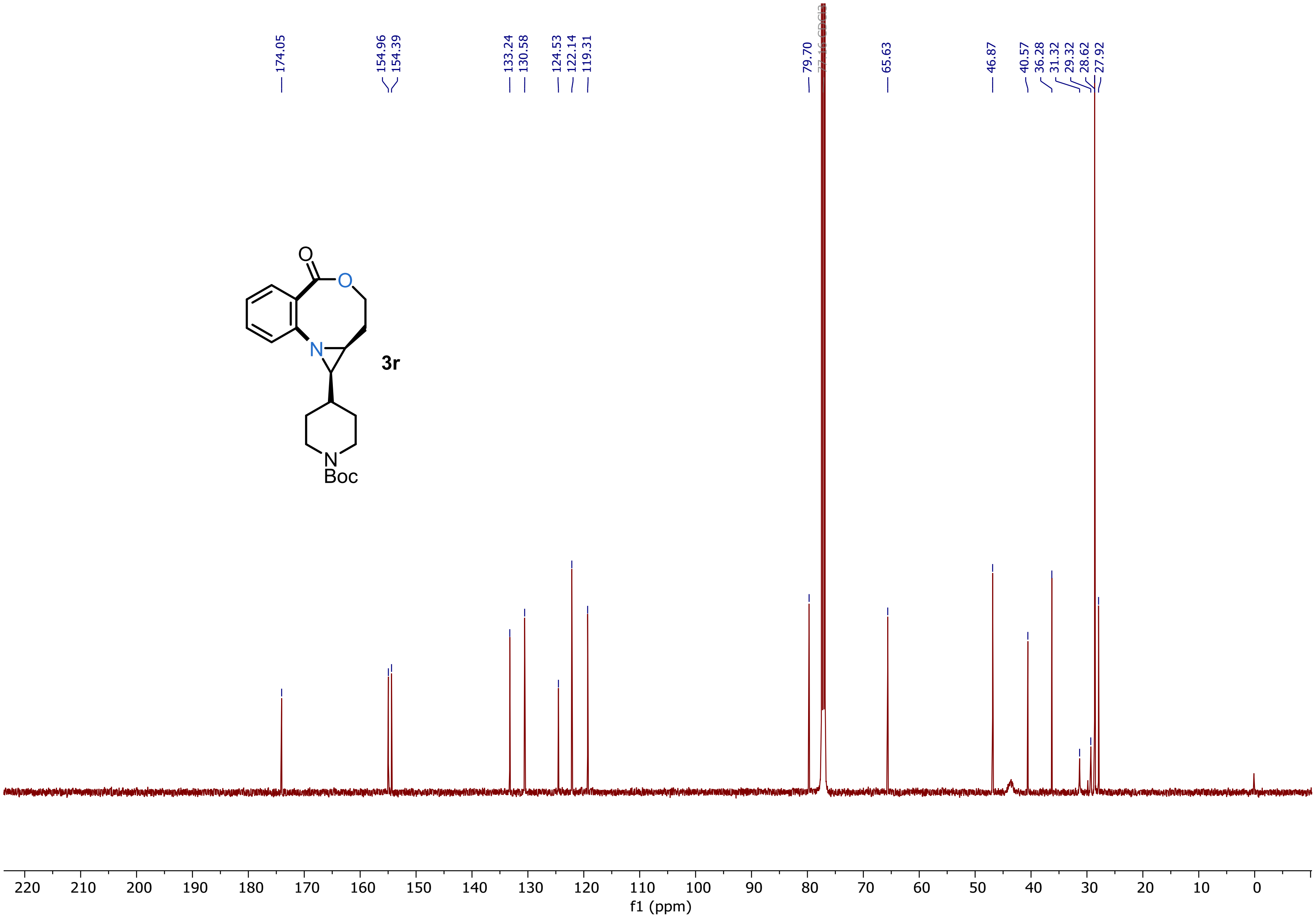

Figure S65. $^{13}C\{^{1}H\}$ NMR (126 MHz, $CDCl_3$) spectrum of **3r**.

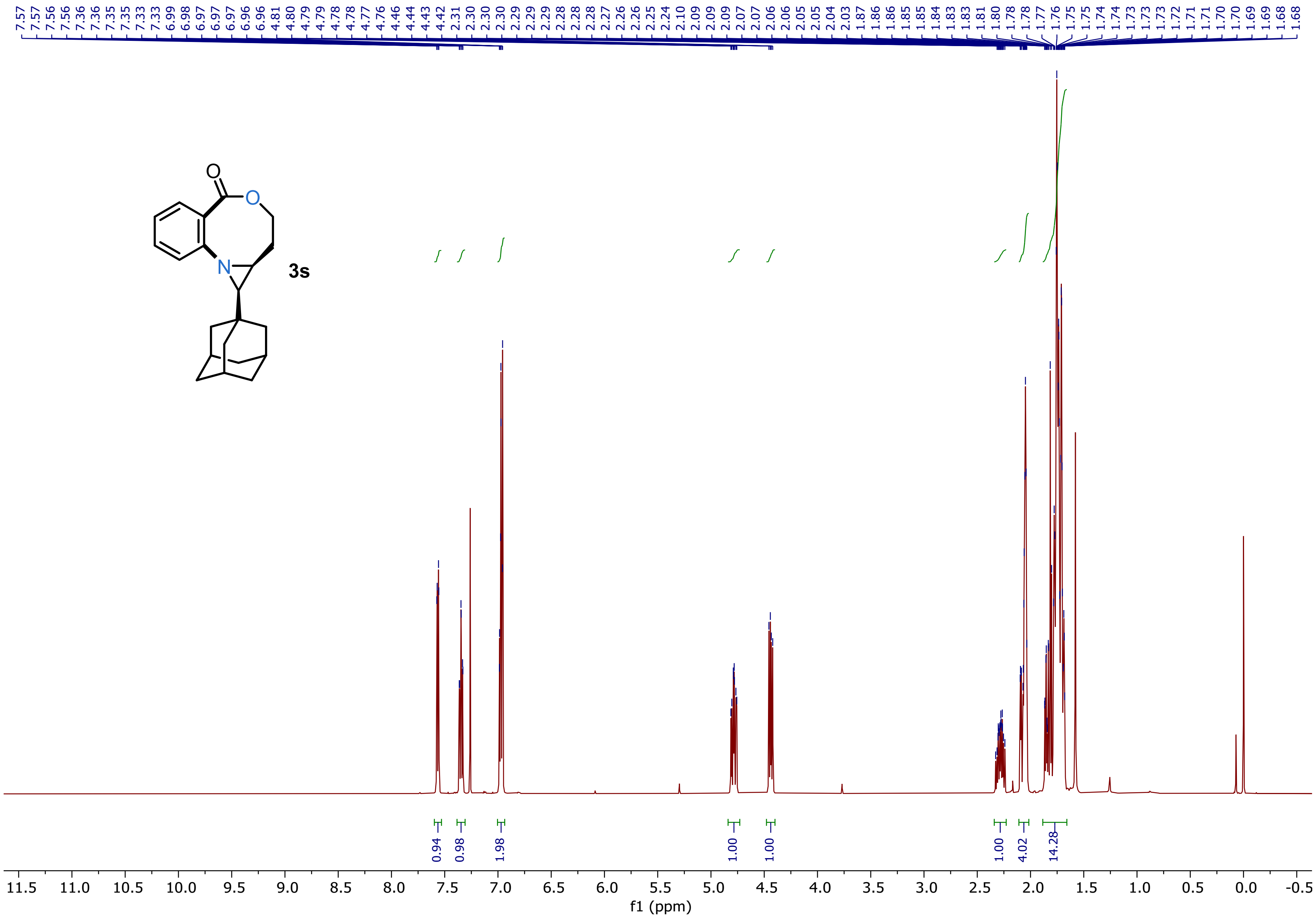

Figure S66. ^{1}H NMR (500 MHz, $CDCl_3$) spectrum of **3s**.

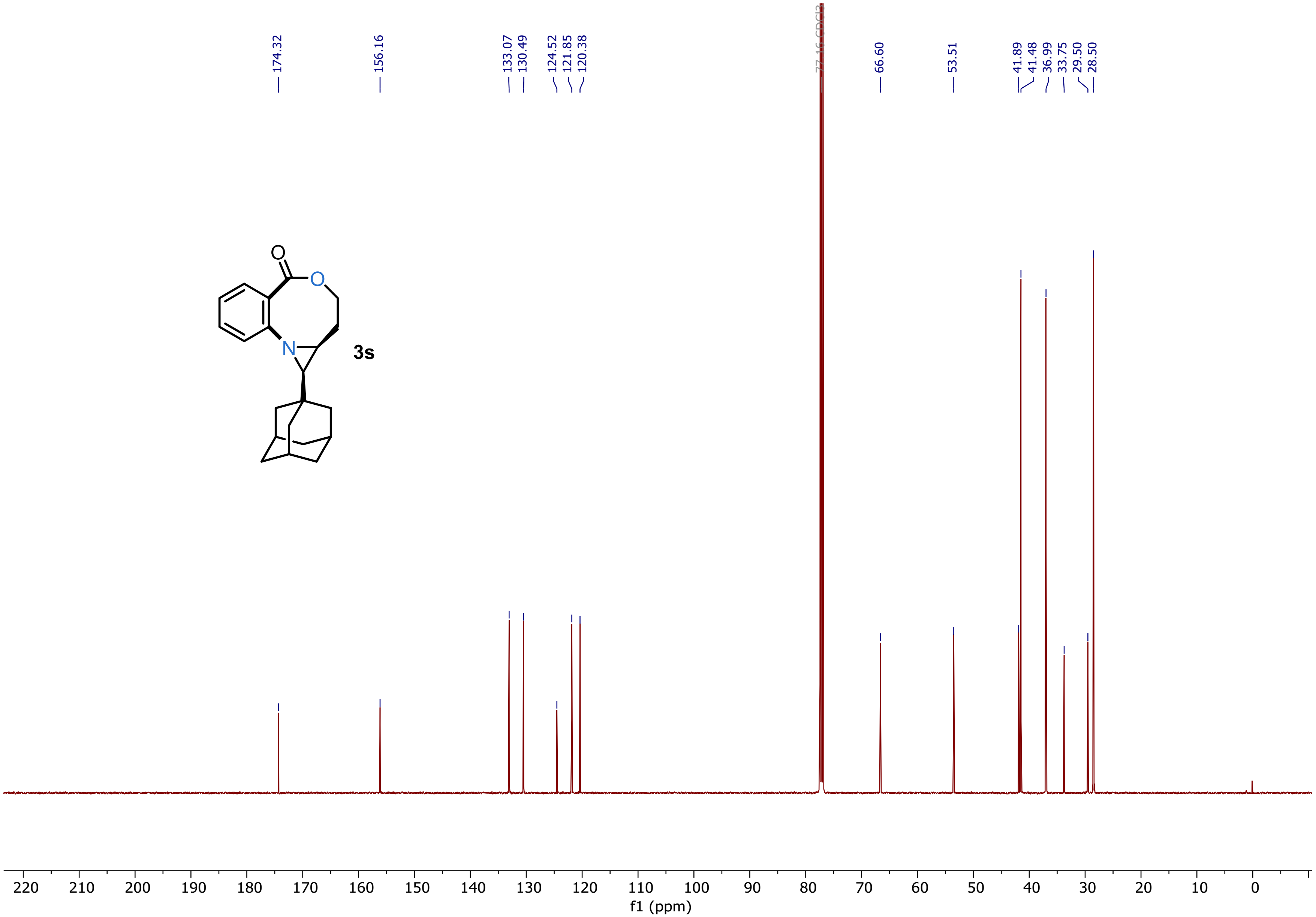

Figure S67. $^{13}C\{^1H\}$ NMR (126 MHz, $CDCl_3$) spectrum of **3s**.

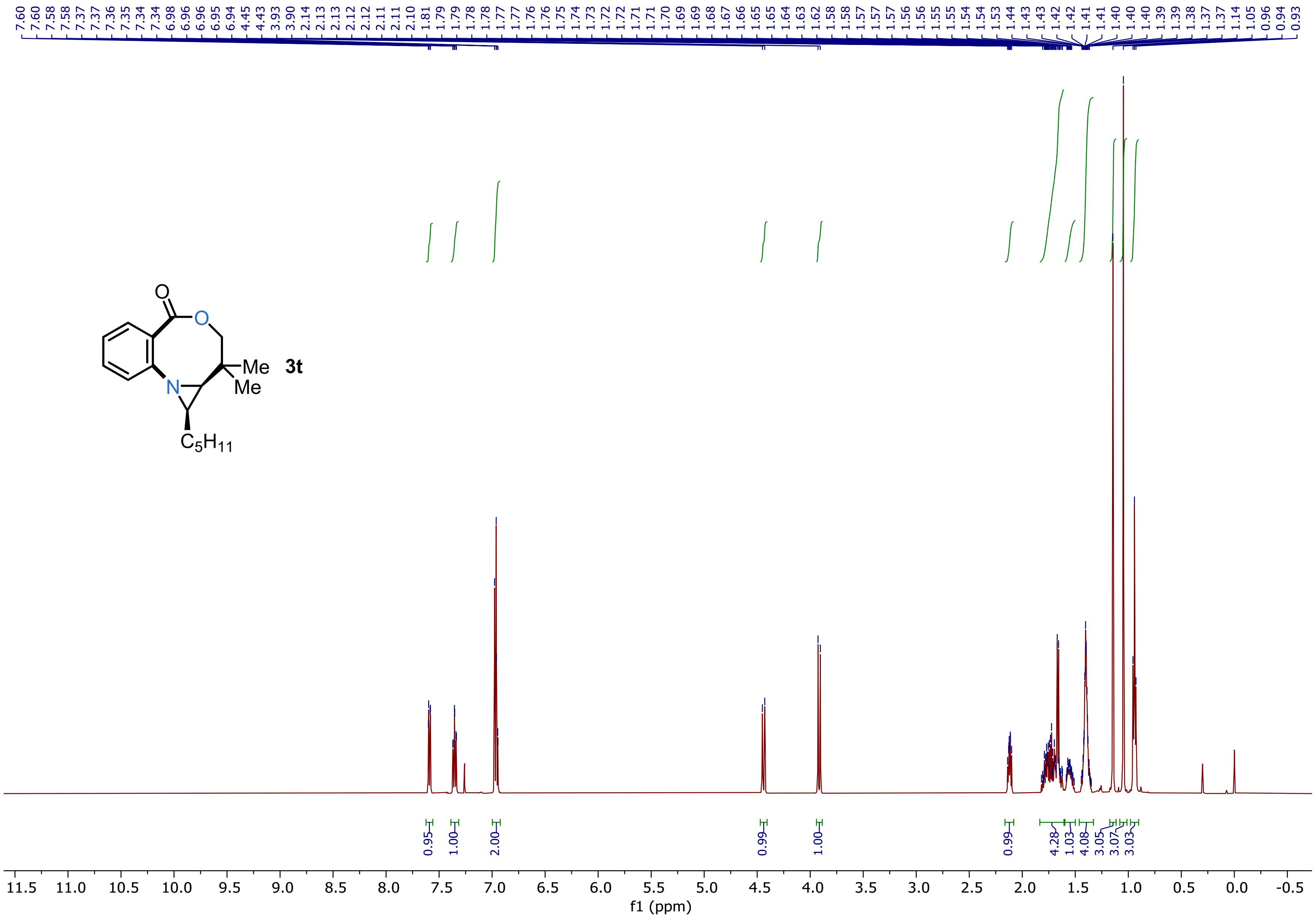

Figure S68. ^{1}H NMR (500 MHz, $CDCl_3$) spectrum of **3t**.

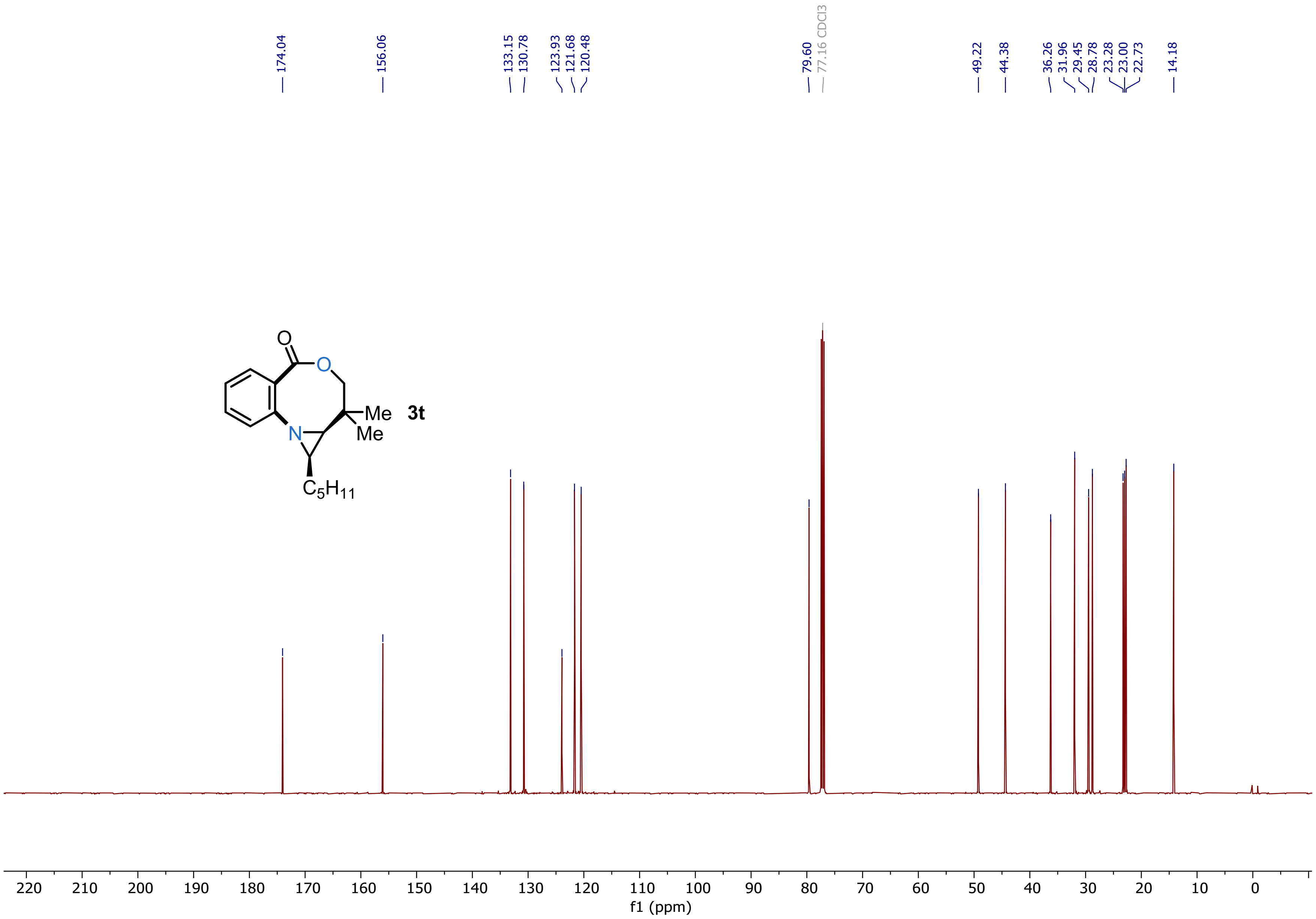

Figure S69. $^{13}C\{^{1}H\}$ NMR (126 MHz, $CDCl_3$) spectrum of **3t**.

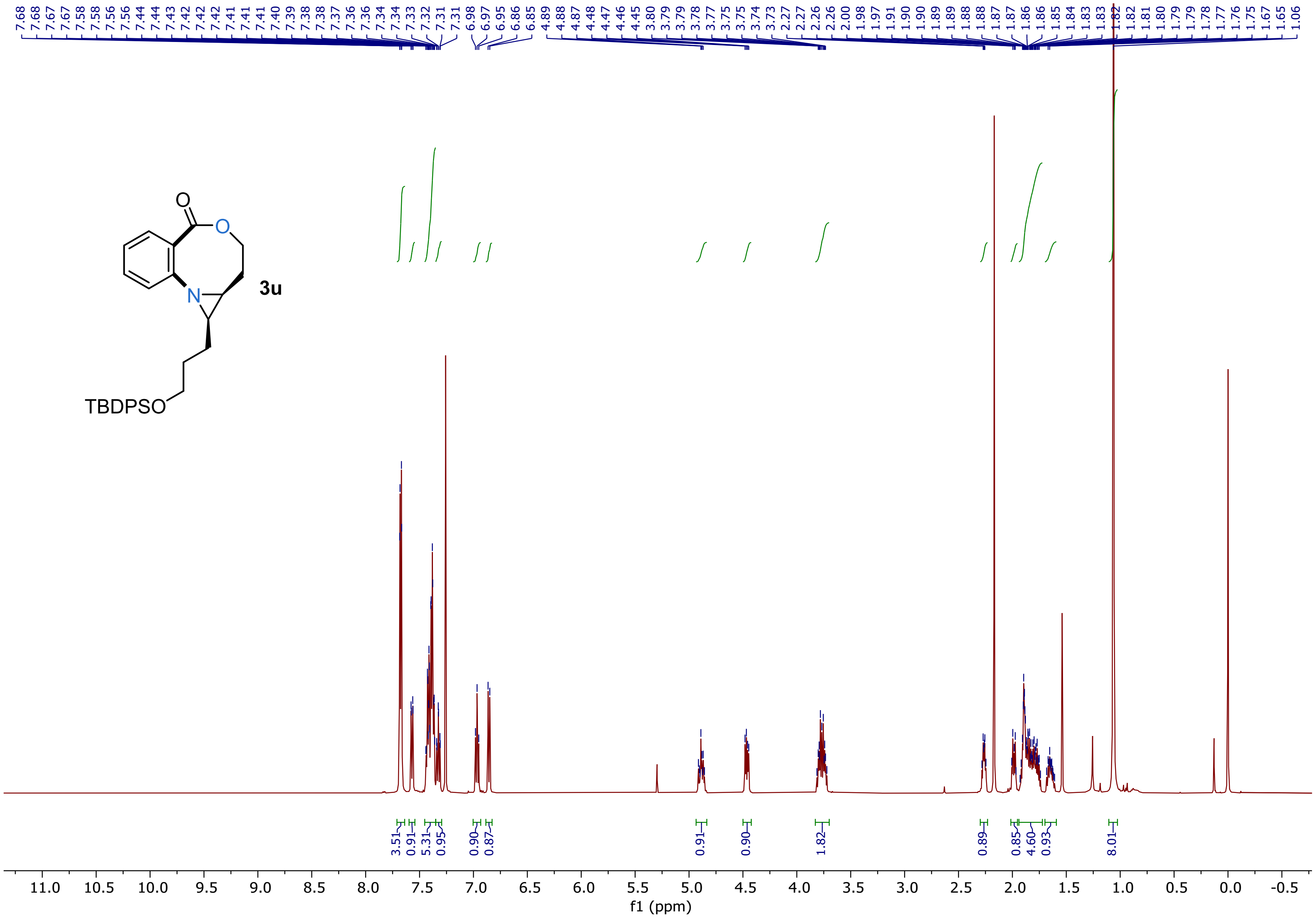

Figure S70. ^{1}H NMR (500 MHz, $CDCl_3$) spectrum of **3u**.

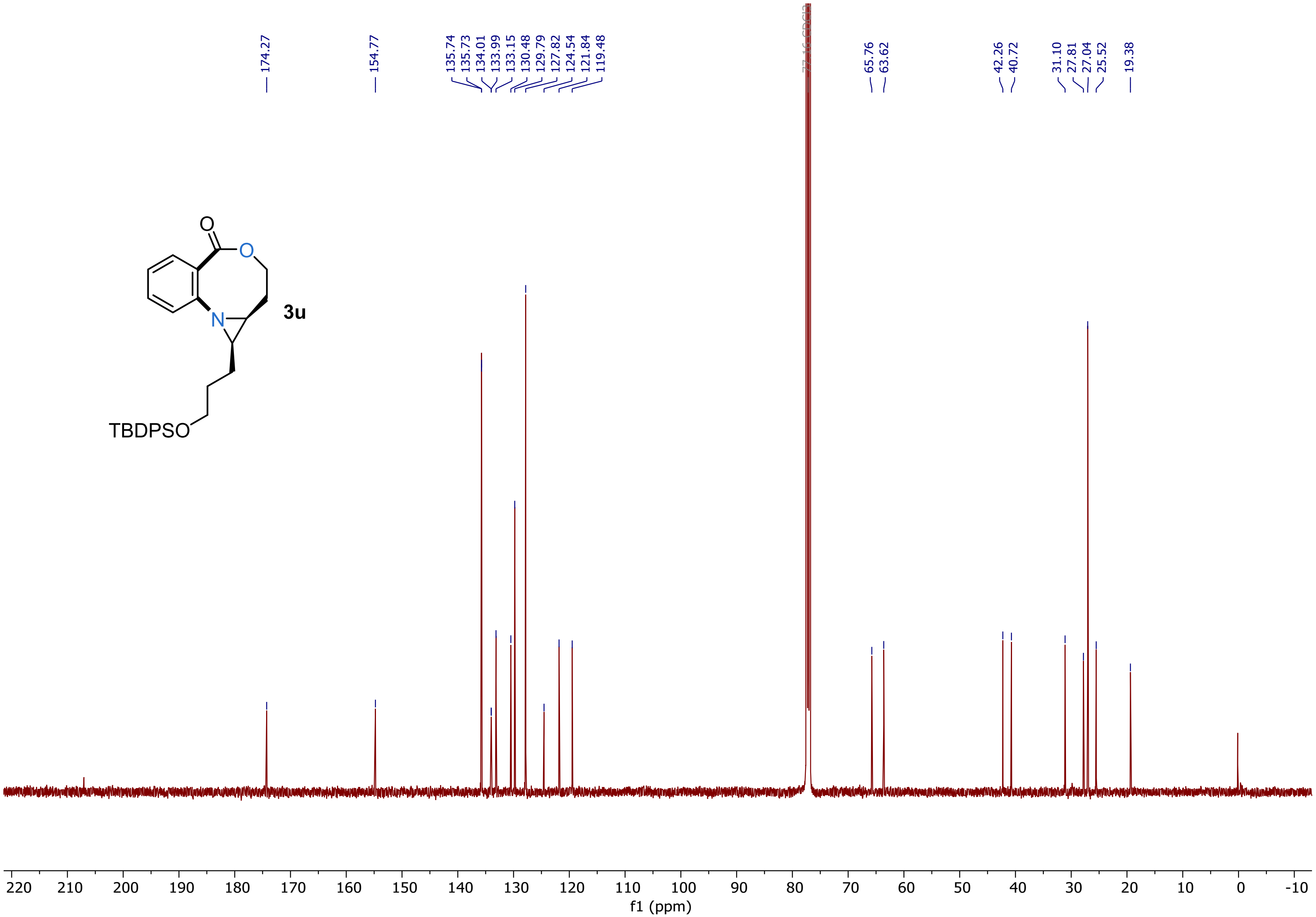

Figure S71. $^{13}C\{^1H\}$ NMR (126 MHz, $CDCl_3$) spectrum of **3u**.

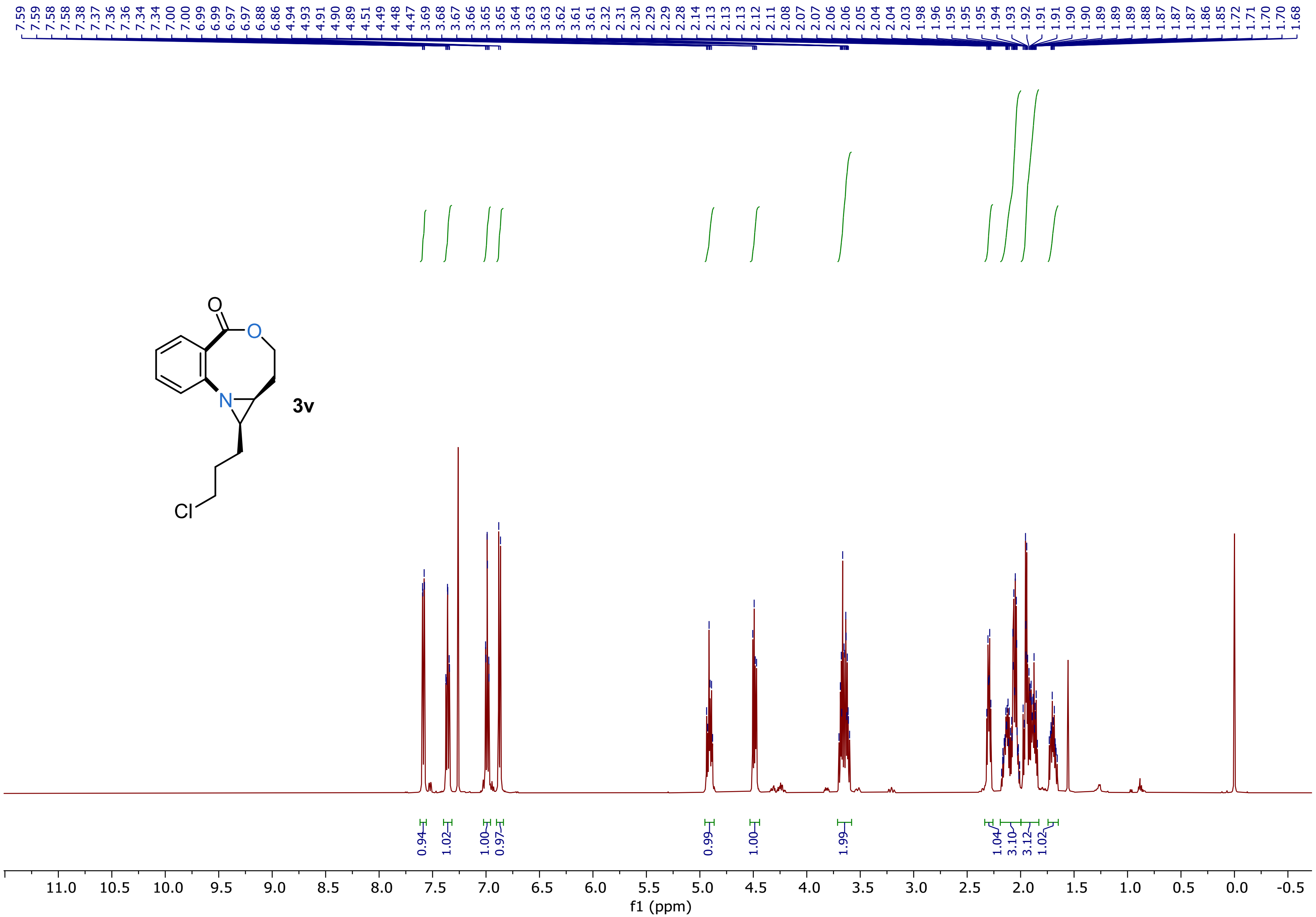

Figure S72. ^{1}H NMR (500 MHz, $CDCl_3$) spectrum of **3v**.

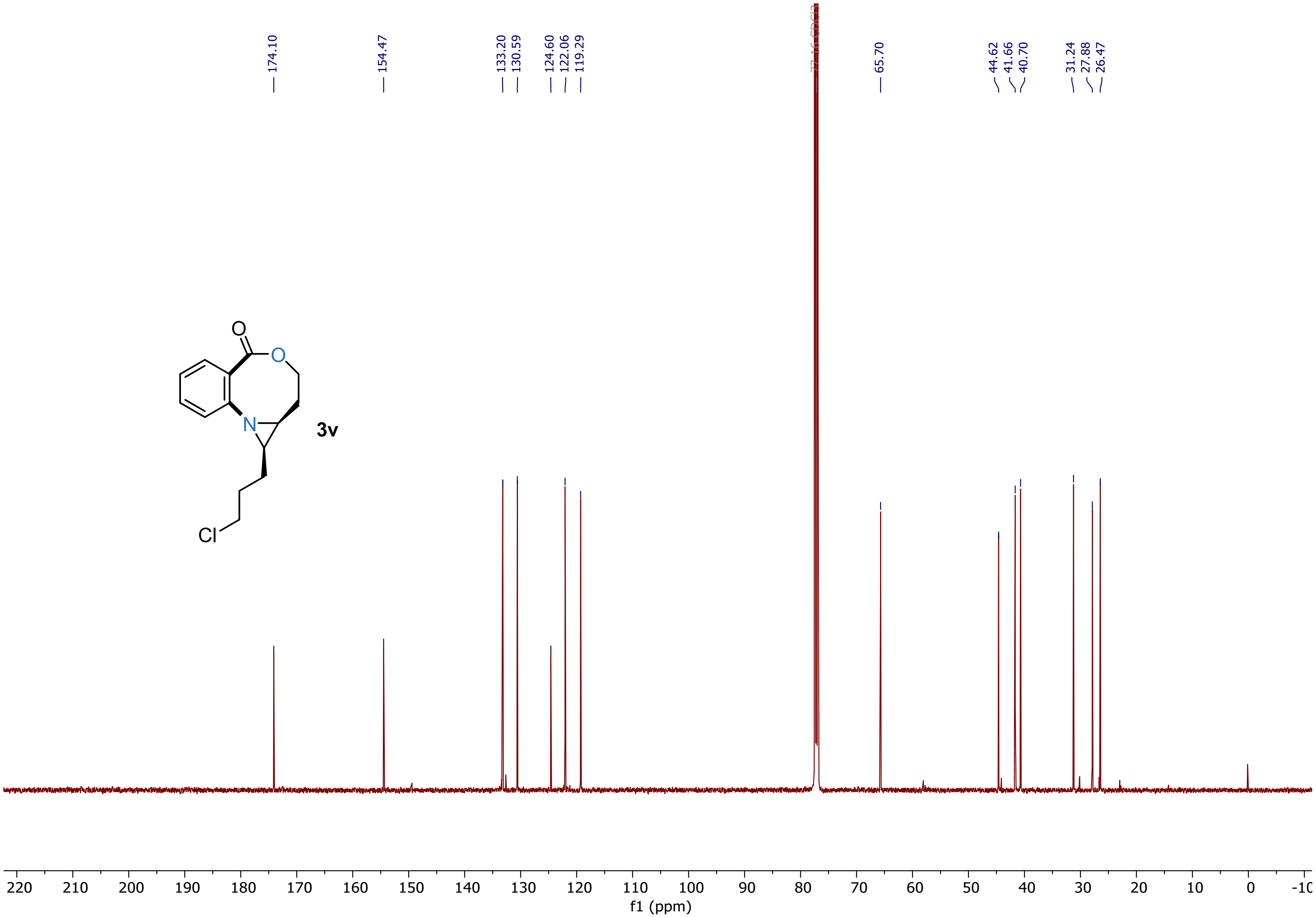

Figure S73. $^{13}C\{^{1}H\}$ NMR (126 MHz, $CDCl_3$) spectrum of **3v**.

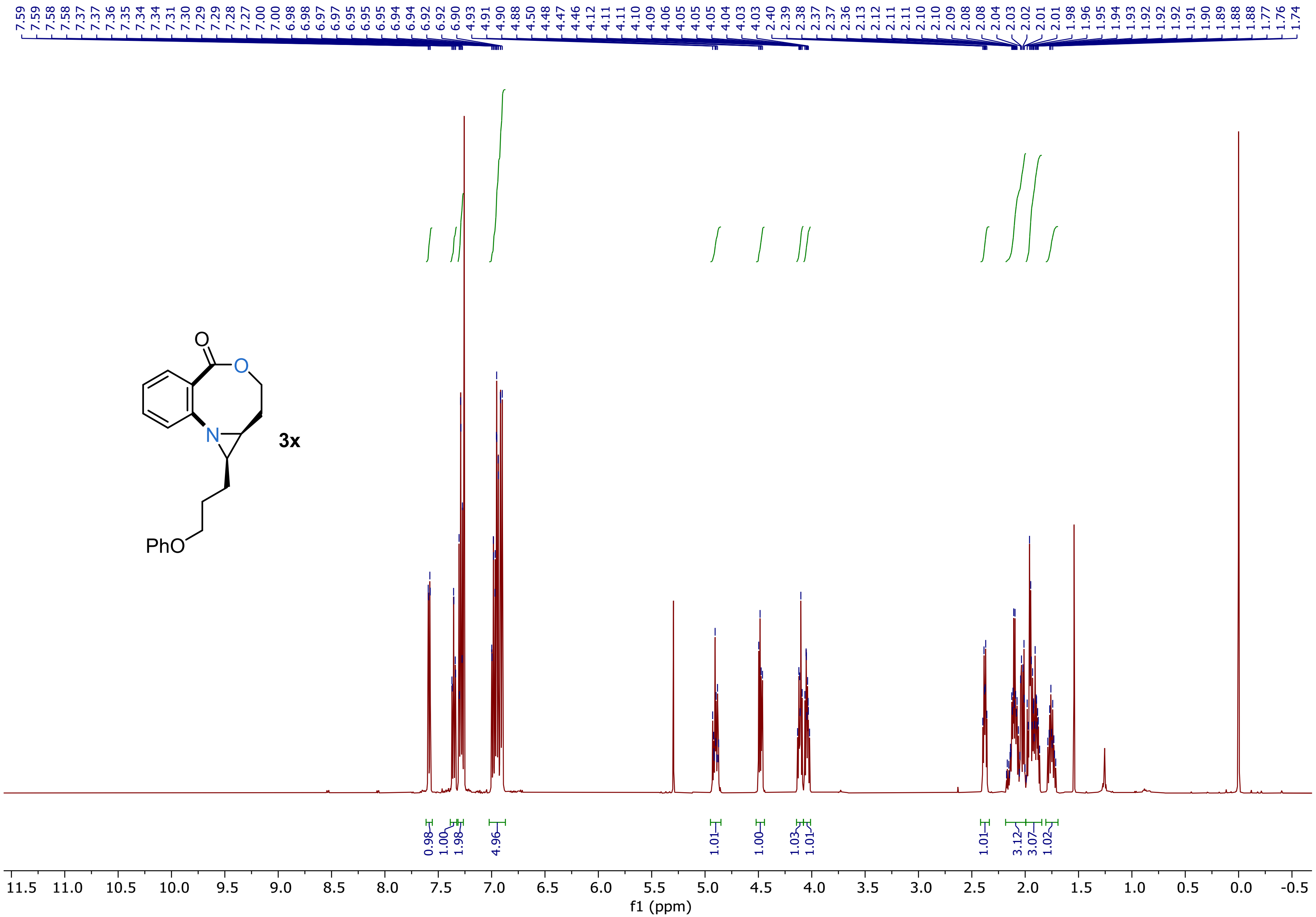

Figure S74. ^{1}H NMR (500 MHz, $CDCl_3$) spectrum of **3x**.

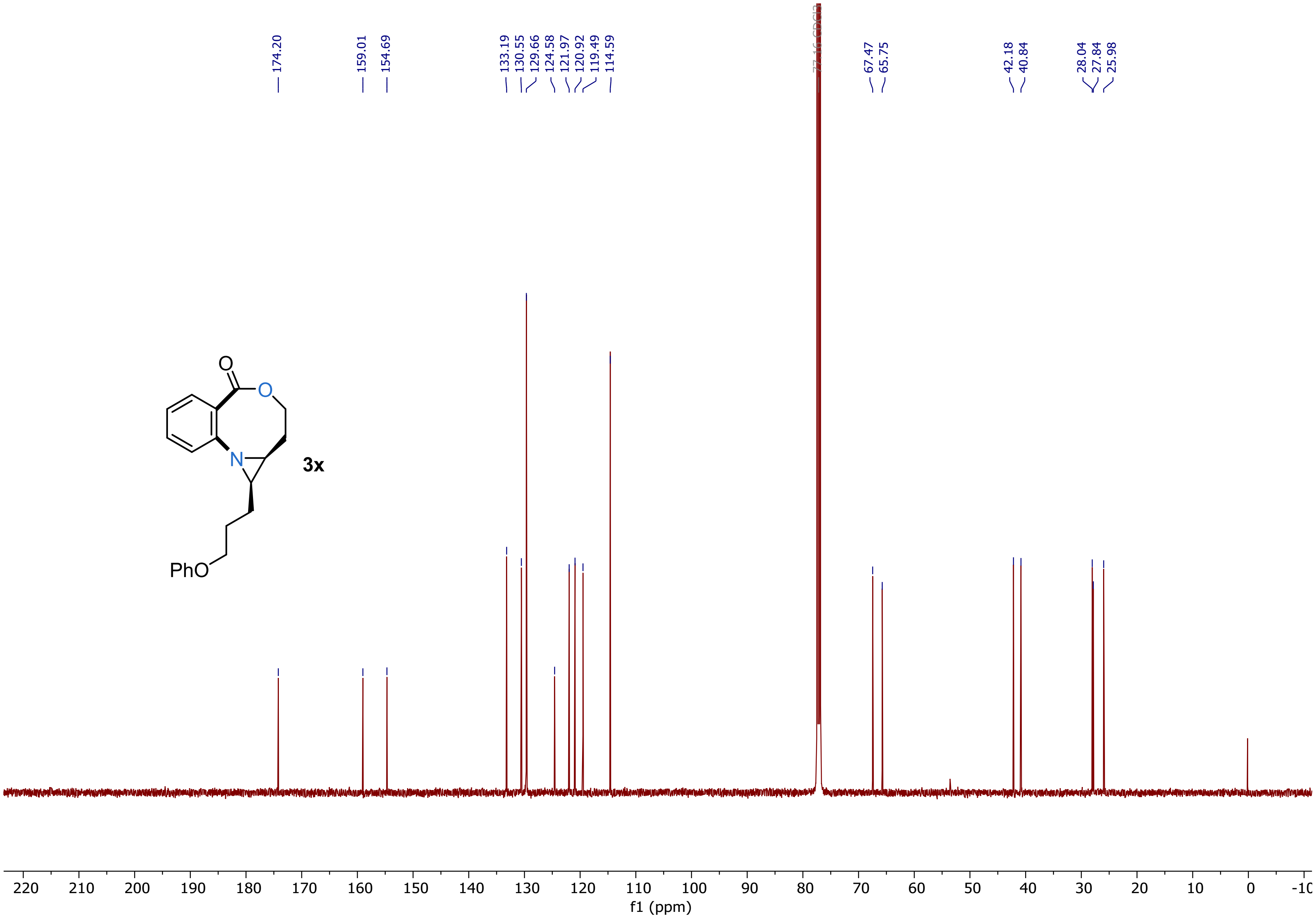

Figure S75. $^{13}C\{^{1}H\}$ NMR (126 MHz, $CDCl_3$) spectrum of **3x**.

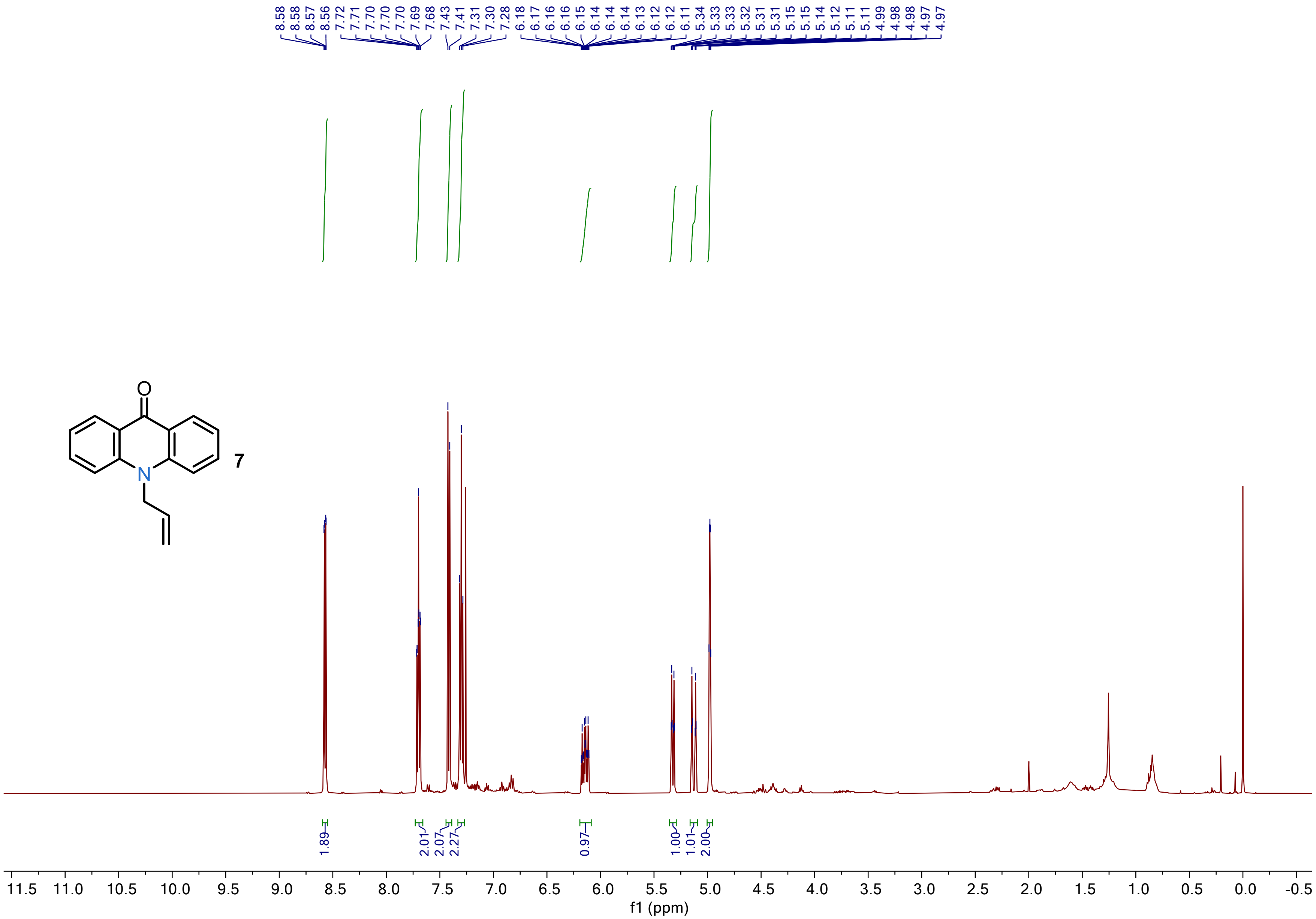

Figure S76. ^{1}H NMR (500 MHz, $CDCl_3$) spectrum of **7**.

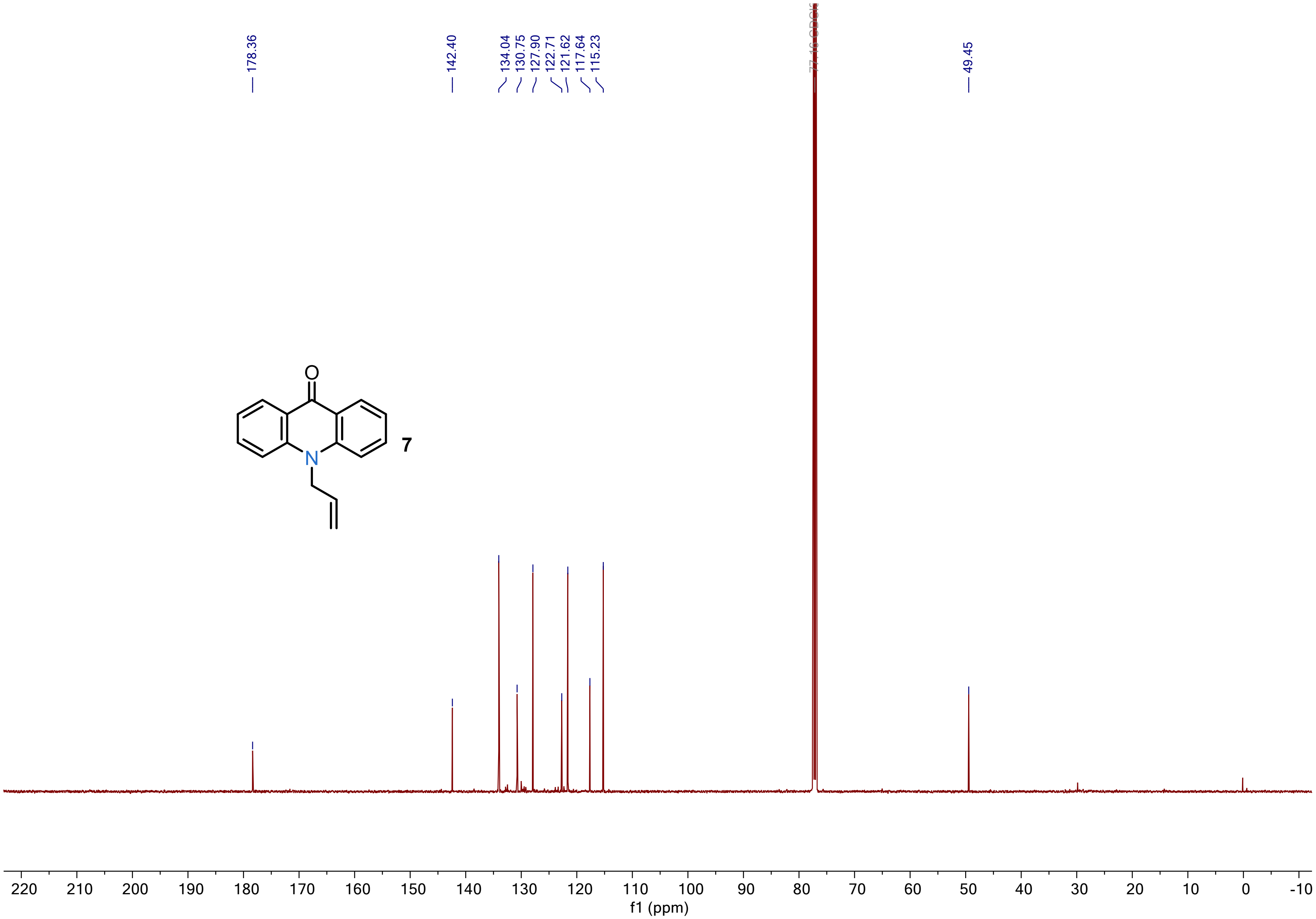

Figure S77. $^{13}C\{^{1}H\}$ NMR (126 MHz, $CDCl_3$) spectrum of **7**.

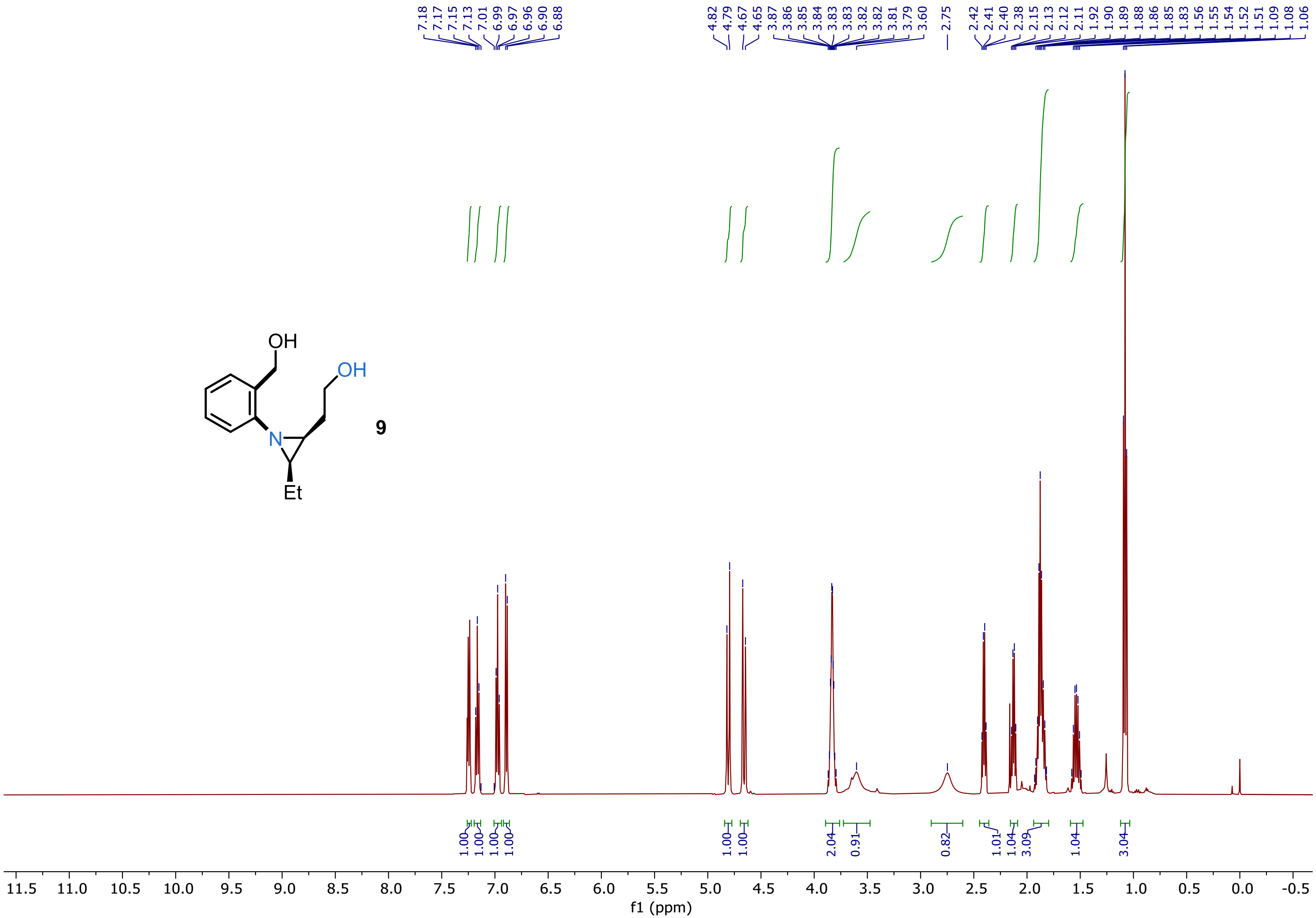

Figure S78. ^{1}H NMR (500 MHz, $CDCl_3$) spectrum of **9**.

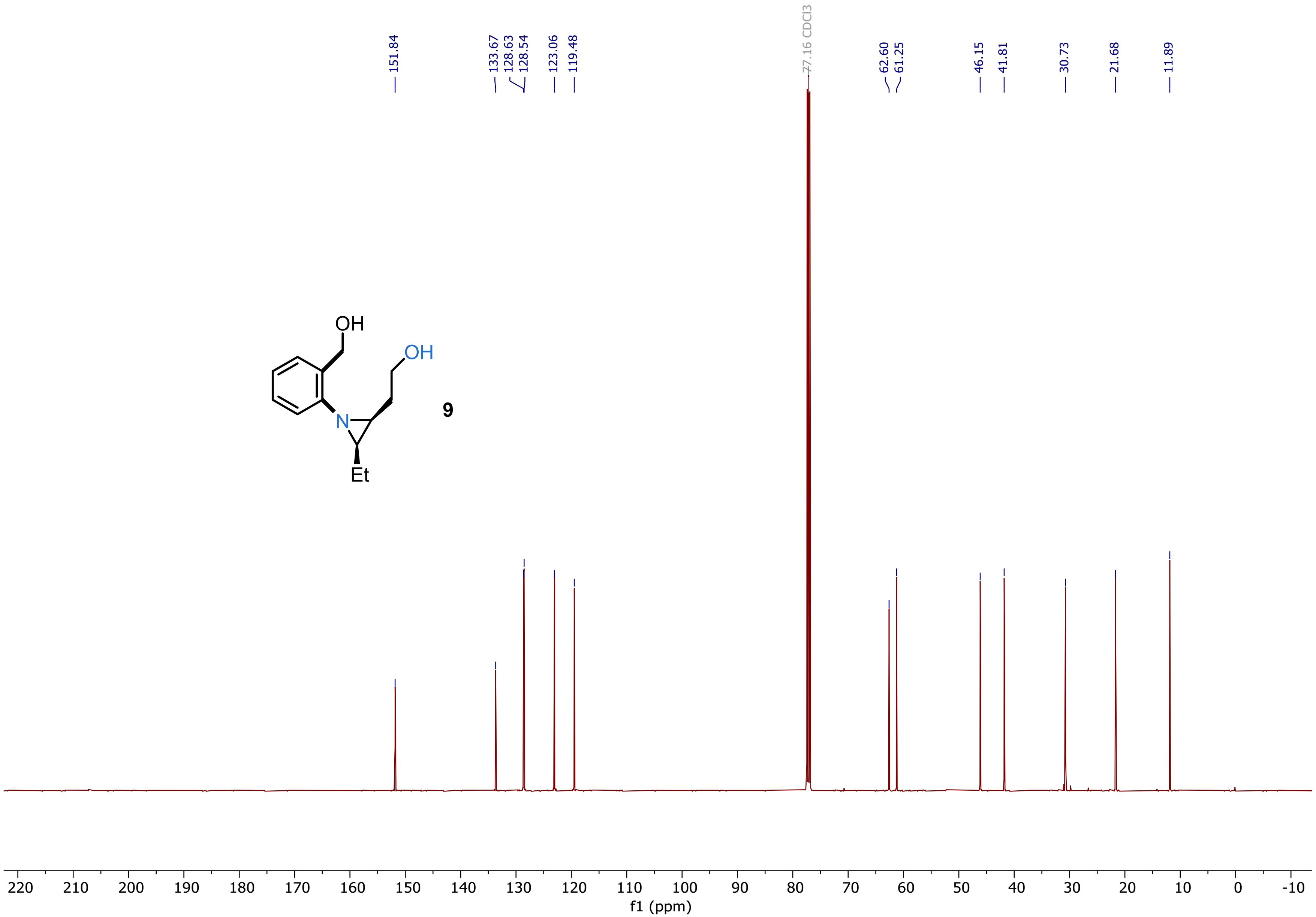

Figure S79. $^{13}C\{^1H\}$ NMR (126 MHz, $CDCl_3$) spectrum of **9**.

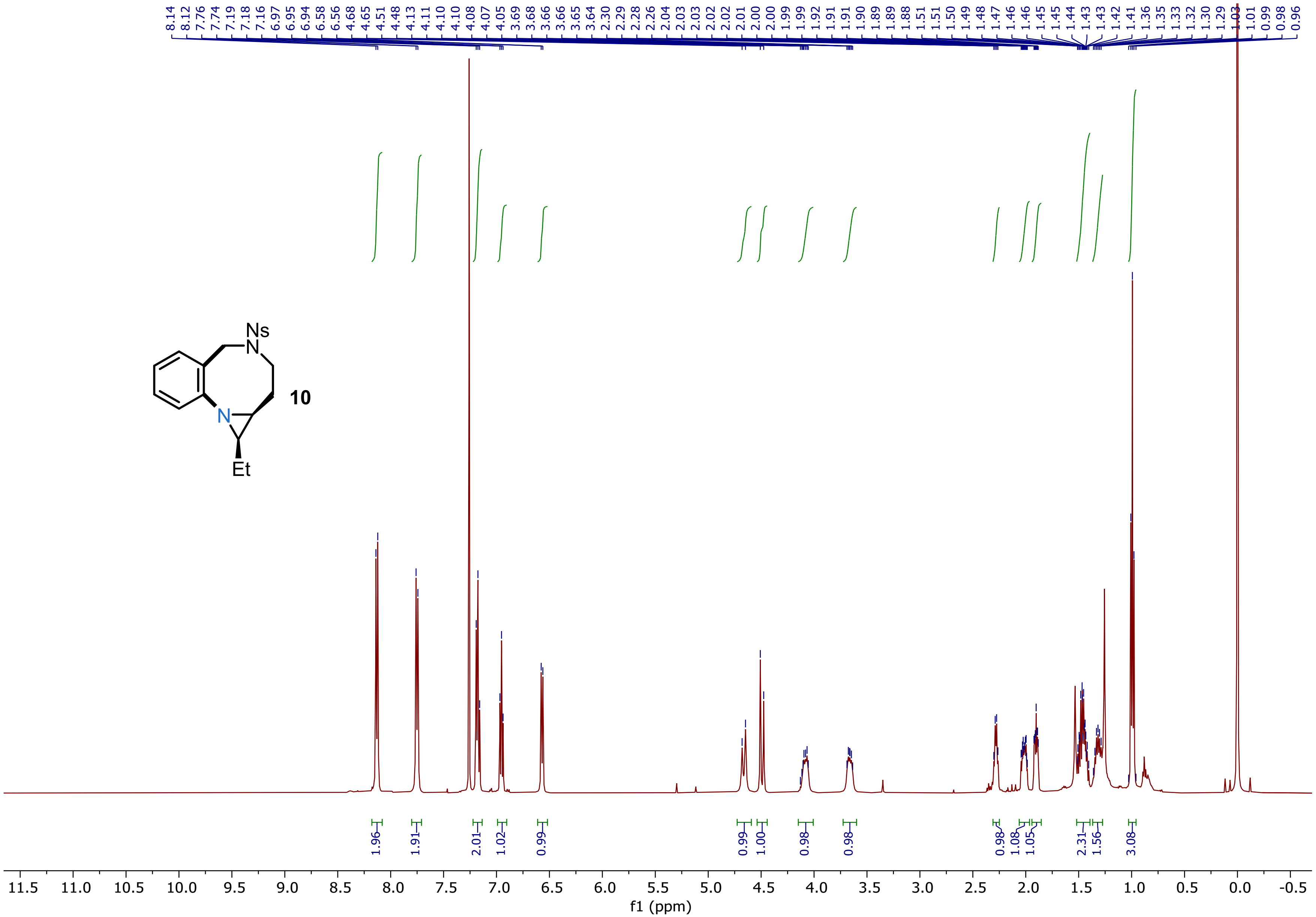

Figure S80. ^{1}H NMR (500 MHz, $CDCl_3$) spectrum of **10**.

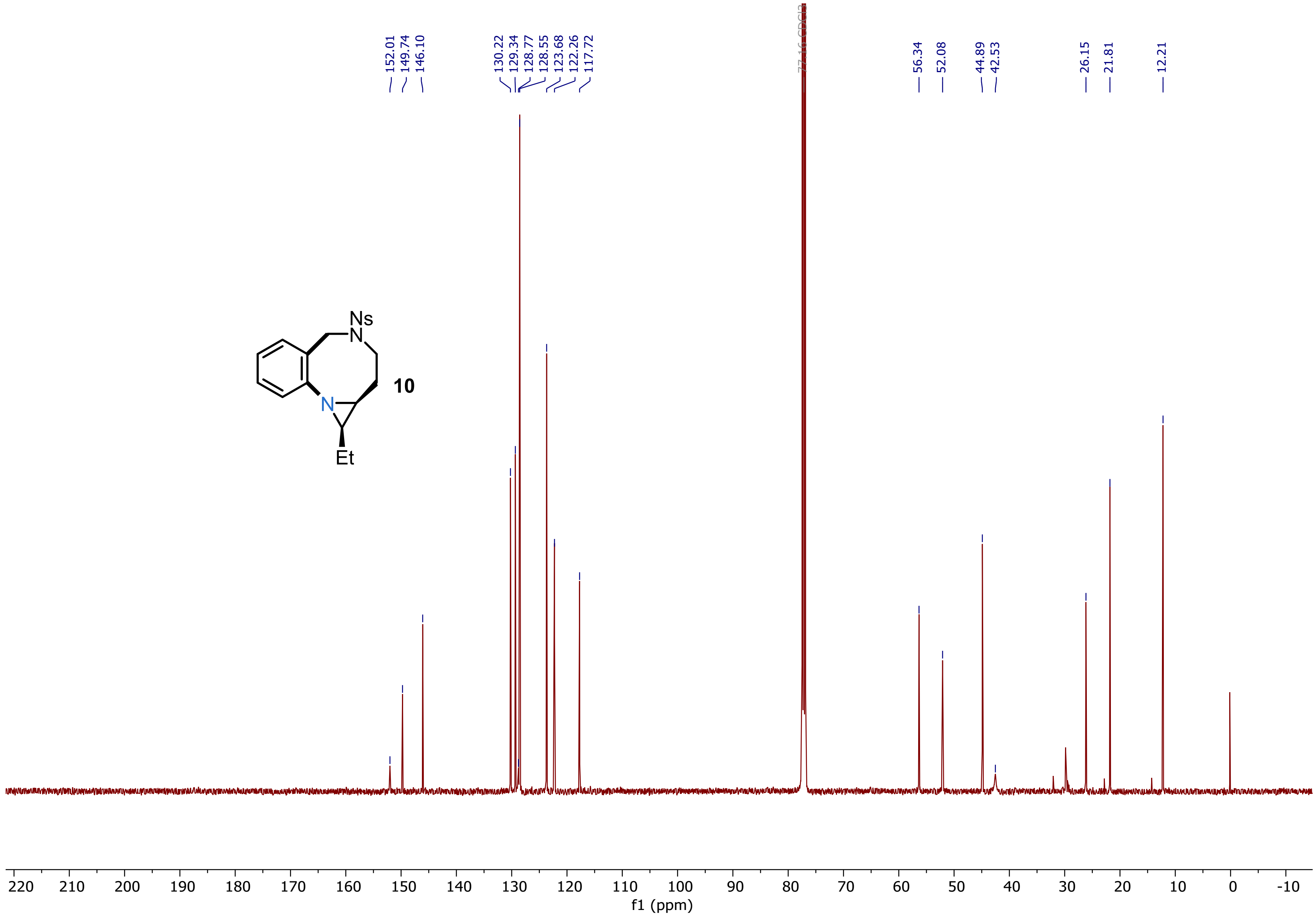

Figure S81. $^{13}C\{^{1}H\}$ NMR (126 MHz, $CDCl_3$) spectrum of **10**.

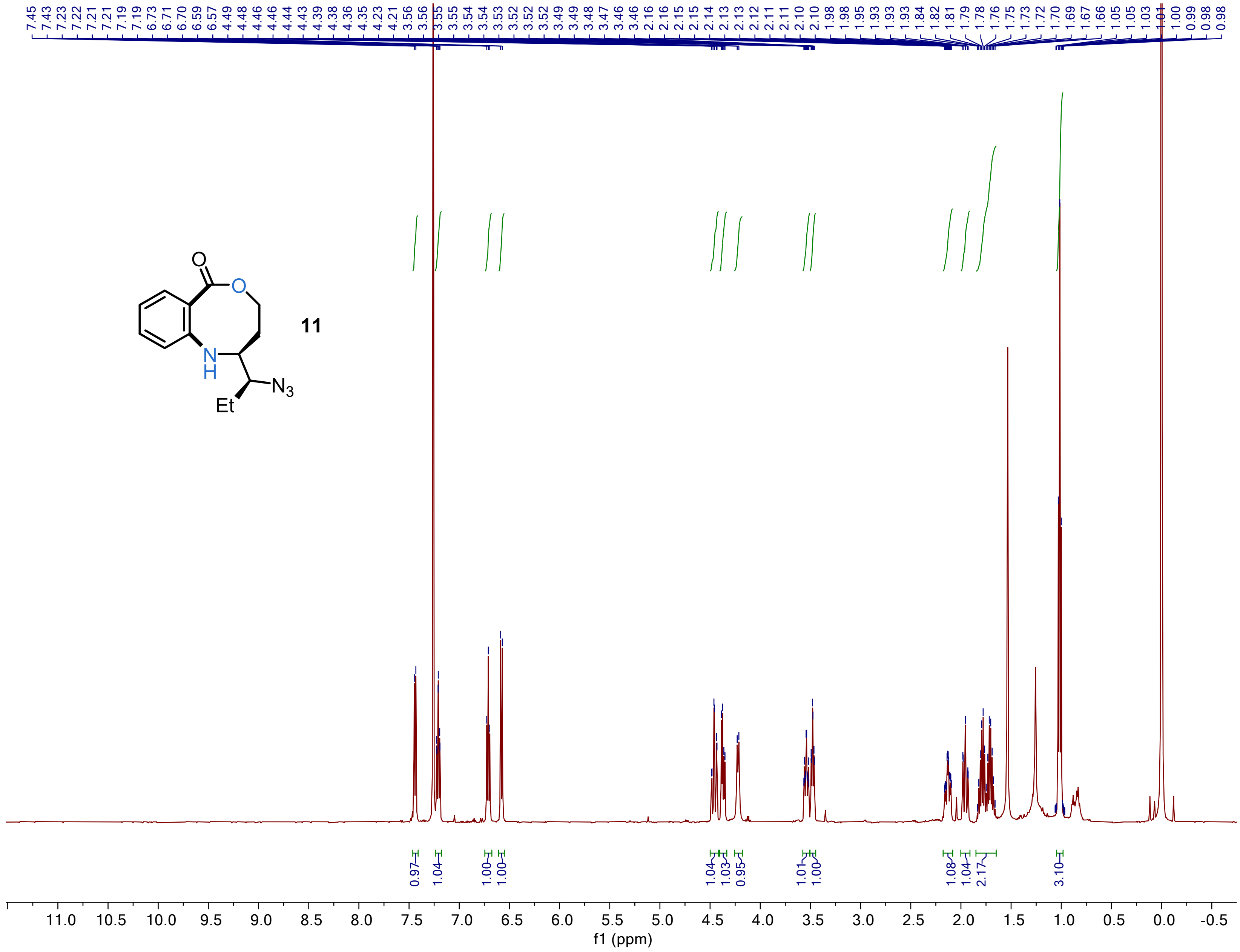

Figure S82. ^{1}H NMR (500 MHz, $CDCl_3$) spectrum of **11**.

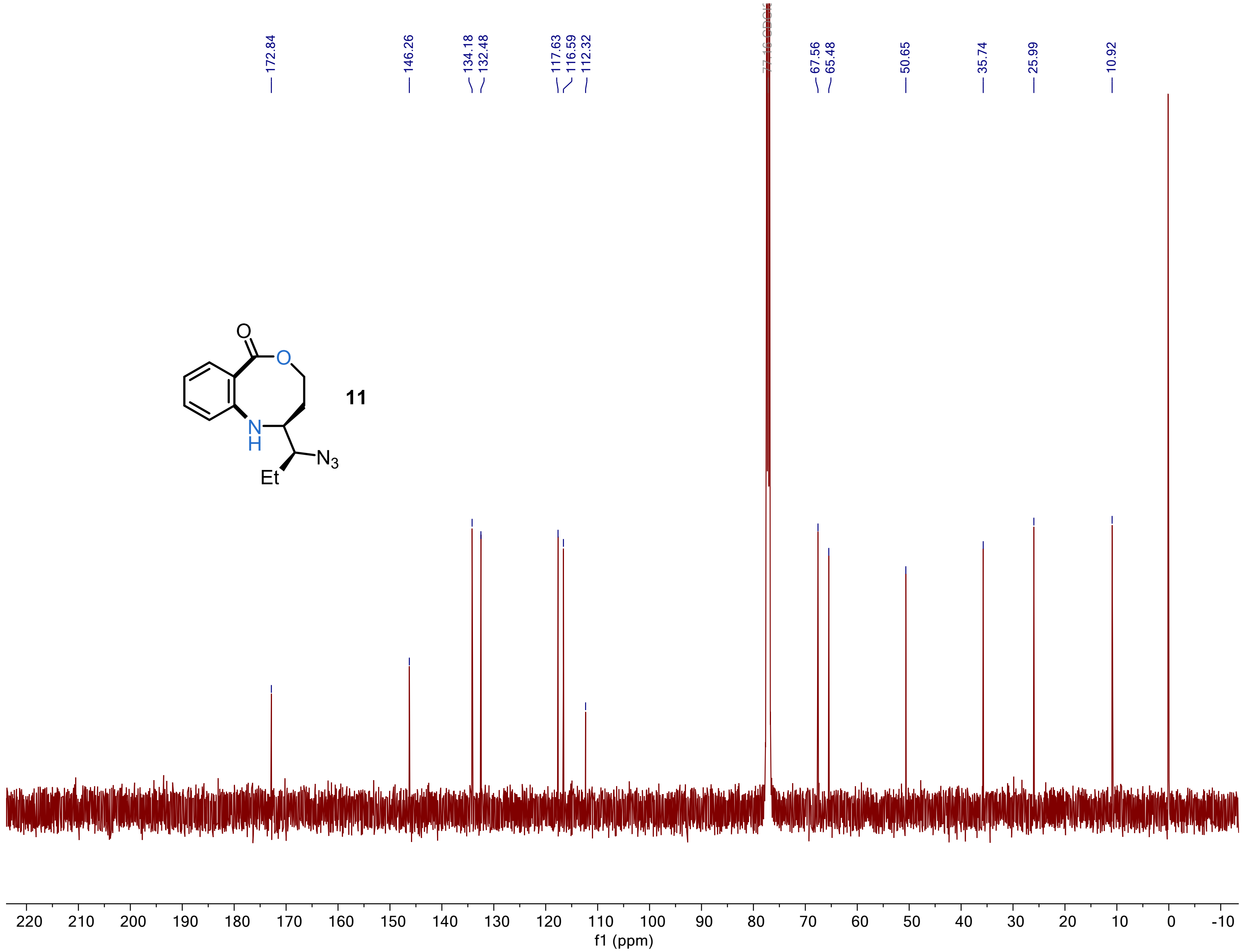

Figure S83. $^{13}C\{^{1}H\}$ NMR (126 MHz, $CDCl_3$) spectrum of **11**.

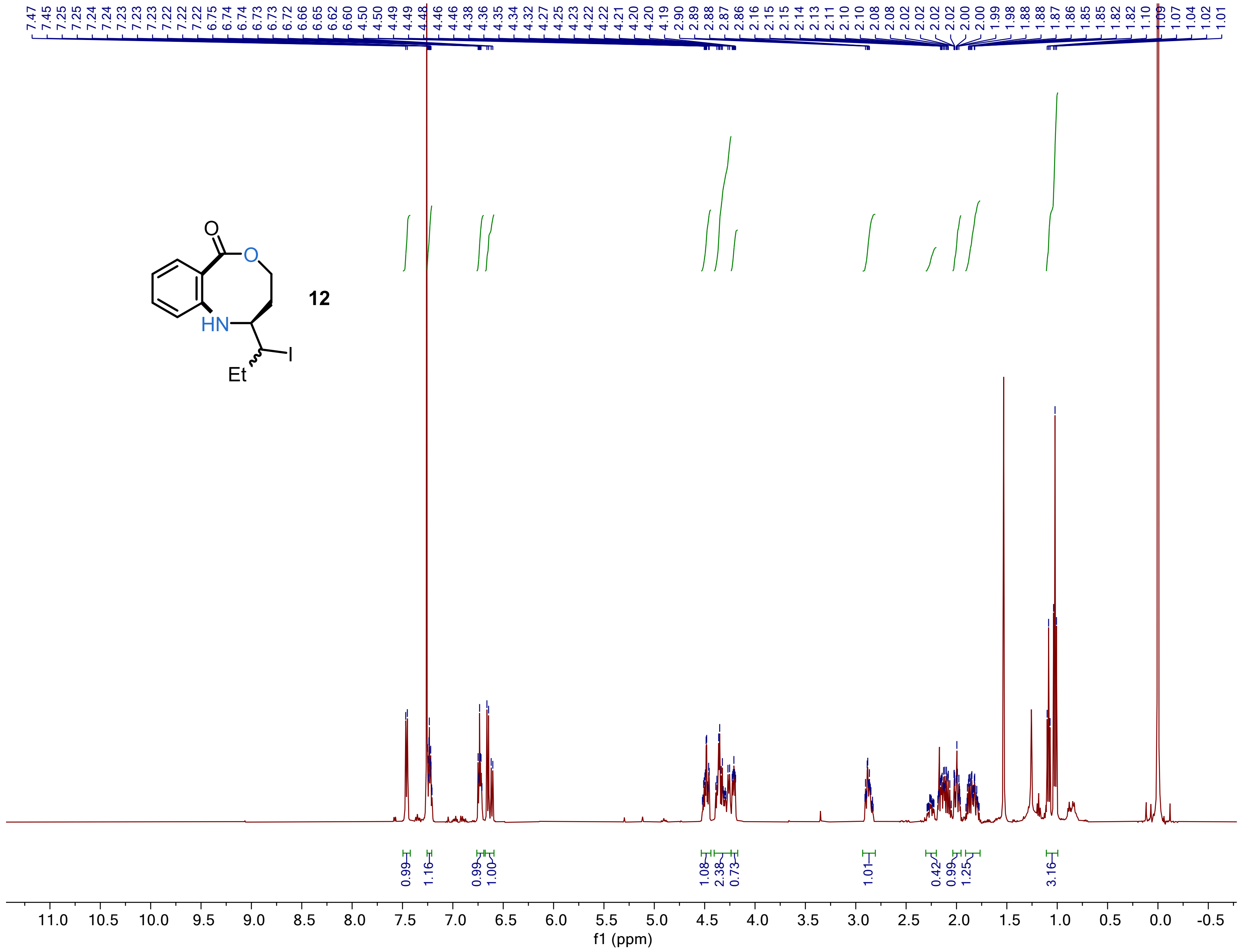

Figure S84. ^{1}H NMR (500 MHz, $CDCl_3$) spectrum of **12**.

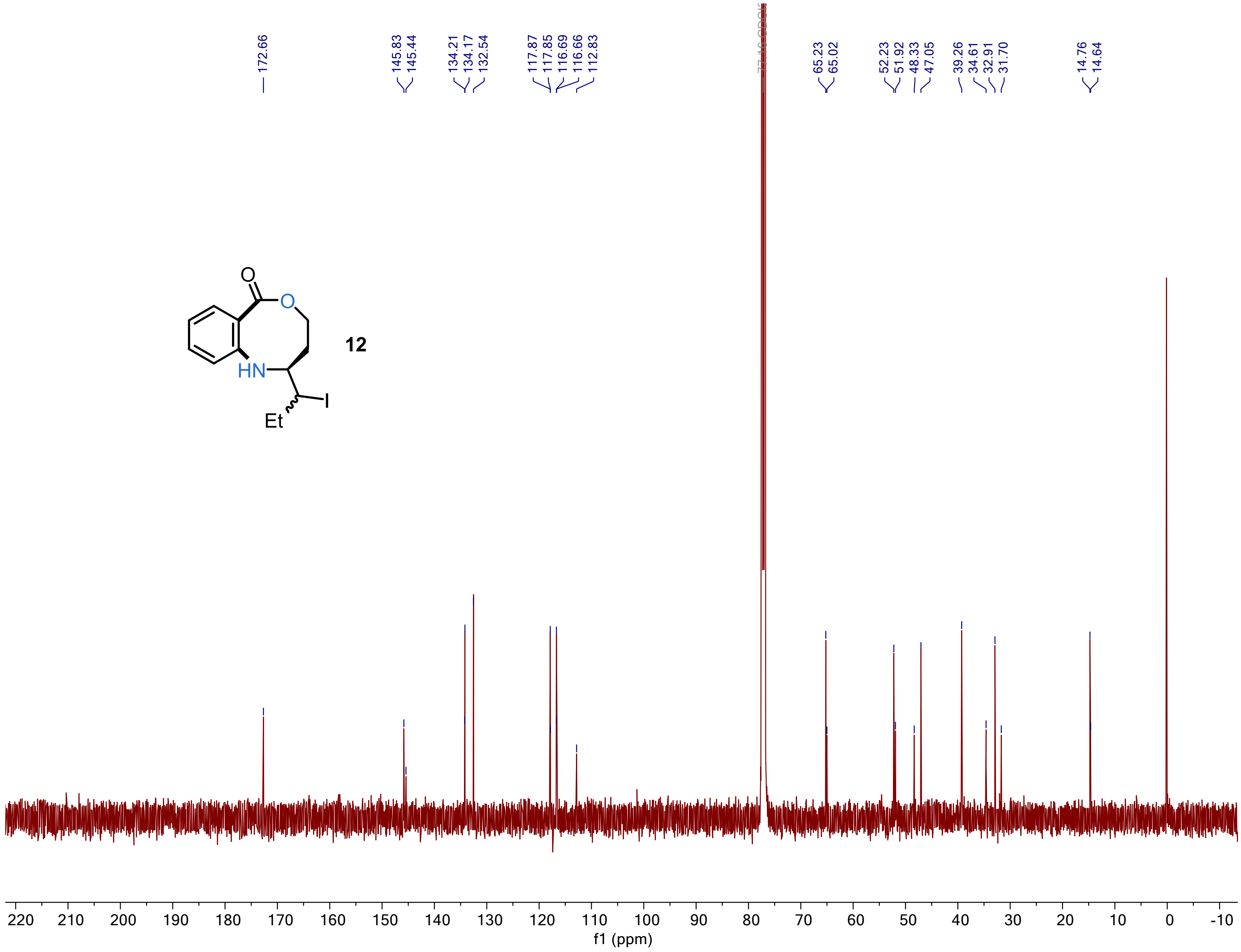

Figure S85. $^{13}C\{^{1}H\}$ NMR (126 MHz, $CDCl_3$) spectrum of **12**.
